# Deazaalloxazines – Flavin Derivatives That Provide Reductive Photoredox Catalysis with Inert Substrates

**DOI:** 10.1002/chem.202502897

**Published:** 2025-11-19

**Authors:** Ivana Weisheitelová, Naisargi Varma, Ludmila Šimková, Josef Chudoba, Tetiana Pavlovska, Iwona Gulaczyk, Gotard Burdziński, Jiří Ludvík, Marek Sikorski, Radek Cibulka

**Affiliations:** ^1^ Department of Organic Chemistry University of Chemistry and Technology, Prague Technická 5 Prague 16628 Czech Republic; ^2^ Faculty of Chemistry Adam Mickiewicz University Uniwersytetu Poznańskiego 8 Poznań 61‐614 Poland; ^3^ Department of Molecular Electrochemistry and Catalysis J. Heyrovský Institute of Physical Chemistry Czech Academy of Sciences Dolejškova 3 Prague 18223 Czech Republic; ^4^ Central Laboratories University of Chemistry and Technology, Prague Technická 5 Prague 16628 Czech Republic; ^5^ Faculty of Physics and Astronomy Adam Mickiewicz University Uniwersytetu Poznańskiego 2 Poznań 61–614 Poland

**Keywords:** cross‐coupling, cyclic voltammetry, deprotection, photoredox catalysis, reduction, spectroscopy

## Abstract

Reductive transformations of substances that are difficult to reduce continue to pose challenges for photoredox catalysis. Promising photoreduction catalysts include flavin and deazaflavin derivatives; however, even their reductive abilities are limited for the range of substrates considered “inert”. In this work, we present 5‐deazaalloxazines, a new group of deazaflavin analogues that are predisposed to catalyze reductions due to their low reduction potential (down to −1.65 V vs. SCE) even in the ground state. We studied three series of 5‐deazaalloxazines ([i] 5‐unsubstituted, [ii] 5‐aryldeazaalloxazines, and [iii] 5‐trifluoromethyl‐5‐deazaalloxazines) to determine their photophysical and electrochemical properties and their ability to participate in model photoreduction reactions. From 31 compounds, we selected 1,3‐dimethyl‐7,8‐dimethoxy‐5‐(*o*‐tolyl)‐5‐deazaalloxazine [**3a(*o*‐MePh)**], as it showed, among other things, the highest efficiency in photodehalogenation of *p*‐fluoroanisole and was photostable and absorbed in the visible light region, thereby allowing photoreactions using a 400 nm LED. Practical applicability was demonstrated in the C─P coupling reaction of electron‐rich aryl halides (including chloroanisoles and *p*‐fluoroanisole) with trimethyl phosphite, providing an arylation reaction to form dimethyl arylphosphonates, and in the release/deprotection of amines from the corresponding tosyl and triflylamides.

## Graphical abstract

7,8‐Dimethoxy‐5‐aryl‐5‐deazaalloxazines (**dAll**) are new class of photostable, visible‐light‐absorbing flavin derivatives which are strongly reducible upon excitation with 400 nm light. It was demonstrated on difficult‐to‐reduce aryl halides like bromo‐, chloro‐ and fluoroanisoles which were transformed to phosphonates by reductive cross‐coupling with trimethyl phosphite or on desulfonylation of sulfonamides to free amines by S─N bond cleavage.

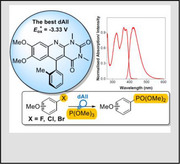

## 1 Introduction

**Figure 1:**
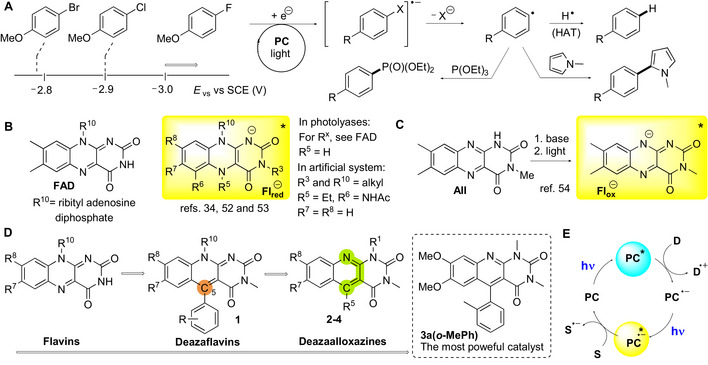
A) Reduction potentials of selected “inert” aryl halides and examples of their uses in arylations to form C─P and C─C bonds using a photocatalyst (PC); B) structure of an FAD cofactor and the structure of excited flavin species involved in photoreductions in both natural and artificial systems; C) formation and structure of an anion derived from oxidized flavin used in photoreductions; D) design of highly photoreductive flavin derivatives, including the structure of the most powerful catalyst developed within this work (for a summary of all tested derivatives, see Table 1); E) the conPET mechanism involved in photoreductions employing 5 deazaflavins **1** and 5‐deazaalloxazines **2** (D = sacrificial donor, S = substrate).

Photoredox catalysis has experienced great progress in recent years and now offers alternative procedures to traditional synthetic approaches and even pushes their boundaries.^[^ ^1^, ^2^, ^3^ ^]^ For reductive processes, photoredox catalysis has brought about methodologies that allow the reduction of “inert” substrates (i.e., those with very negative reduction potentials).^[^ ^4^, ^5^ ^]^ A typical example of difficult reductive processes is the generation of aryl radicals by the reduction/dehalogenation of aryl halides (Figure 1A), which are inexpensive and readily available precursors for radical arylation reactions that form C‐heteroatom or C─C bonds.^[^ ^6^, ^7^ ^]^ The reduction potentials of aryl bromides and aryl chlorides with electron‐donating substituents can reach values more negative than −2.7 V versus saturated calomel electrode (SCE). Typically, the reduction of these “inert” substrates has been achieved using light‐excited radicals formed within consecutive photoinduced electron transfer processes (conPET) processes^[^ ^8^, ^9^, ^10^, ^11^, ^12^, ^13^ ^]^ (i.e., by employing a radical generated by PET from a sacrificial reductant and its subsequent excitation with a second photon), or alternatively, by using an excited radical species generated electrochemically.^[^ ^14^, ^15^ ^]^ Recent results have shown that the reducing agent in some of these highly efficient systems is actually a solvated electron or anion formed from the intermediate radical species.^[^ ^16^, ^17^, ^18^, ^19^, ^20^, ^21^ ^]^ Very strong reducing agents can also be generated using approaches involving proton‐coupled electron transfer (PCET),^[^ ^22^, ^23^ ^]^ PET from an excited anion or dianion,^[^ ^24^, ^25^, ^26^, ^27^, ^28^, ^29^, ^30^, ^31^, ^32^ ^]^ or multiphoton electron transfer.^[^ ^33^ ^]^ The latter two processes even allow the reduction of aryl fluorides, which are the most difficult substrates to reduce (*E*_red_ < −3.0 V vs. SCE).

Well‐established photoredox catalysts include flavins—substances inspired by flavin cofactors.^[^ ^34^, ^35^ ^]^ Flavin derivatives were originally used in organocatalytic oxidations and photooxidations;^[^ ^36^, ^37^, ^38^, ^39^, ^40^, ^41^, ^42^, ^43^, ^44^, ^45^, ^46^, ^47^, ^48^, ^49^, ^50^, ^51^ ^]^ however, only a few procedures using flavins to enable photoreductions have been published recently. For example, Storch introduced a system that uses excited reduced 5‐alkylflavins **Fl_red_^‒^** that mimic the reductive system of FAD in photolyases (Figure 1B).^[^ ^52^, ^53^ ^]^ An excited flavin anion **Fl_ox_^‒^** generated from isomeric alloxazine **All** was also reported as a potent reductant (Figure 1C).^[^ ^54^ ^]^ Overall, 5‐aryl‐5‐deazaflavins **1** (Figure 1D) stand out in terms of their ability to reduce inert substrates. Systems with **1** use an excited radical anion within the conPET process (Figure 1E) and are capable of reducing both electron‐rich aryl bromides and aryl chlorides.^[^ ^55^, ^56^ ^]^ 5‐Deazaflavins have also been applied for the arylation of white phosphorus^[^ ^57^ ^]^ or triethyl phosphite.^[^ ^55^ ^]^ However, the systems with 5‐aryldeazaflavins still have only limited reductive ability, as they do not reduce electron‐rich aryl fluorides. Furthermore, some of 5‐aryldeazaflavin catalysts suffer from low photostability.

In the present study, we decided to further tune structure of 5‐deazaflavin derivatives to improve their capacity for reductive photoredox catalysis. Our idea was to switch to isomeric 5‐deazaalloxazines (**dAll**) (Figure 1D), as these are stronger reducing agents (by approximately 300 mV)^[^ ^58^ ^]^ in the ground state. The use of 5‐deazaalloxazines is almost unknown in photoredox catalysis, and they have been studied mostly as bioactive compounds thus far.^[^ ^59^, ^60^, ^61^, ^62^ ^]^ The only exception is our group's report of the application of two 5‐deaalloxazine derivatives for the reductive desulfonylation of amines and phenols.^[^ ^63^ ^]^ We have also studied an unsubstituted 5‐deazaalloxazine as a singlet oxygen photosensitizer.^[^ ^64^ ^]^ Here, we focused on studying the structure versus photocatalytic efficiency relationship for a large sample of 5‐deazaalloxazine derivatives **2**‒**4** and we developed a detailed map of their photophysical properties in order to find the structural rules influencing the catalytic ability and to predict the optimal structure(s) leading to the most potent catalyst. This extensive study has identified a superior catalyst, **3a(*o*‐MePh)**, which, unlike 5‐deazaflavins, is robust and stable and enables the reduction of the most difficult substrates, including aryl fluorides. We demonstrate its practical application in arylation of trimethyl phosphite and sulfonamide desulfonylations on selected challenging substrates.

## 2 Results and Discussion

### 2.1 Design and Synthesis

5‐Deazaalloxazines show promising characteristics for use in reductive catalysis. Their effectiveness can be assumed based on their ground state reduction potential, which is still 300 mV more negative than that of 5‐deazaflavins.^[^ ^58^ ^]^ However, the absorption maximum of 5‐deazaalloxazines at around 350 nm (representing the most hypsochromic shift among flavin derivatives)^[^ ^64^ ^]^ might be disadvantageous, as excitation with more energetic UV light can result in decreased selectivity of the reactions. Nevertheless, we assumed that we could use substitution to tune the spectral properties of the 5‐deazaalloxazines to make them also suitable for visible light photoredox catalysis.

The structure of the investigated 5‐deazaalloxazines **2**‒**4** was chosen after considering the effort required to study the influence of substitution on the photophysical properties. Here, we focused especially on a substitution at positions 5, 7, and 8. Keeping in mind our main goal to prepare the most efficient catalyst for photoreductions, we used substituents that are inert from the point of view of reduction processes (e.g., MeO, Me, and CF_3_). We were also inspired by the positive effect of the phenyl substituent in position 5 on the ability of isomeric 5‐deazaflavins **1** to participate in conPET photoreductions because of radical stabilization.^[^ ^55^, ^56^ ^]^ Therefore, we paid special attention to 5‐aryl‐5‐deazaalloxazines **3** and to the structure of the 5‐aryl group, including the possibility of its “replacement” with a heteroaryl unit (see Table 1 for the full list of the prepared 5‐deazaalloxazines). Note, we have included especially deazaalloxazines with 5‐phenyl group substituted in ortho position that maintain both rings in a perpendicular arrangement, which can further stabilize radical intermediates.^[^ ^9^ ^]^

**Table 1:** Structures of investigated 5‐deazaalloxazines and their photophysical and electrochemical properties and catalytic efficiency.

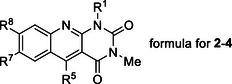 [for general formula for **1**, see Figure 1D]						Photophysical and electrochemical properties, and catalytic efficiency							
No	R^5^	R^7^	R^8^	R^1^		*λ*_abs_ [nm] /log *ε*	*λ*_F_ [nm]	*Φ*_F_	*τ*_F_ [ns] ^[^ ^b^ ^]^	*Φ*_Δ_	*τ*_T_ [µs]	*E*_1/2_[V]^[^ ^c^ ^]^	Catalytic efficiency^[^ ^d^ ^]^
**1(*o*‐MePh)** ^[^ ^a^ ^]^	*o*‐MePh^[^ ^a^ ^]^	OMe^[^ ^a^ ^]^	OMe^[^ ^a^ ^]^	‐ ^]^		418/4.25	470	0.58	7.75	0.026	n.d. ^[^ ^e^ ^]^	−1.33	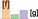
**2a**	H	OMe	OMe	Me		366/4.13	415	0.19	2.25	0.13	13	−1.59	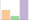
**2a‐H**	H	OMe	OMe	H		366/3.69	412	0.22	2.48	0.12	n.d. ^[^ ^e^ ^]^	−1.58	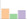
**2b‐H**	H	OMe	H	H		380/3.97	441	0.48	10.54	0.15	n.d. ^[^ ^e^ ^]^	−1.43	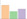
**2c‐H**	H	H	OMe	H		347/4.40	392	0.18	2.73	0.22	n.d. ^[^ ^e^ ^]^	−1.51	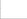
**2d‐H**	H	Me	Me	H		360/3.98	412	0.21	3.83	0.24	20	−1.48	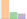
**2e**	H	H	H	Me		357/3.94	408	0.14	2.47	0.32	20	n.d. ^[^ ^e^ ^]^	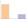
**3a(Ph)**	Ph	OMe	OMe	Me		367/3.92	432	0.14	1.64	0.16	41	−1.61	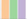
**3a(*o*‐BrPh)**	*o*‐BrPh	OMe	OMe	Me		370/3.97	427	0.030	0.31	0.22	n.d. ^[^ ^e^ ^]^	−1.59	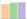
**3a(*o*‐BrPh)‐H**	*o*‐BrPh	OMe	OMe	H		370/4.04	426	0.031	0.33	0.22	42	−1.55	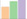
**3a(*p*‐BrPh)**	*p*‐BrPh	OMe	OMe	Me		369/4.05	437	0.13	1.52	0.17	n.d. ^[^ ^e^ ^]^	n.d. ^[^ ^e^ ^]^	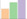
**3a(*o*‐MePh)**	*o*‐MePh	OMe	OMe	Me		367/3.97	423	0.15	1.59	0.13	28	−1.65	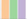
**3a(*o*‐MePh)‐H**	*o*‐MePh	OMe	OMe	H		368/4.09	422	0.20	2.08	0.096	33	−1.61	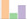
**3a(napht‐1‐yl)**	napht‐1‐yl	OMe	OMe	Me		370/3.98	428	0.014	0.13	0.038	n.d. ^[^ ^e^ ^]^	n.d. ^[^ ^e^ ^]^	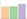
3a(pyridin‐4‐yl)	pyridin‐3‐yl	OMe	OMe	Me		372/3.95	449	0.23	2.45	0.16	n.d. ^[^ ^e^ ^]^	n.d. ^[^ ^e^ ^]^	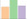
3a(pyridin‐2‐yl)	pyridin‐2‐yl	OMe	OMe	Me		371/4.05	472	0.17	2.34	0.23	n.d. ^[^ ^e^ ^]^	n.d. ^[^ ^e^ ^]^	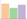
3a(thiophen‐3‐yl)	thiophen‐3‐yl	OMe	OMe	Me		372/3.91	433	0.020	0.41	0.036	0 ^[^ ^h^ ^]^	−1.61	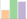
3b(Ph)	Ph	OMe	H	Me		385/3.33	459	0.47	10.63	0.15	n.d. ^[^ ^e^ ^]^	−1.48	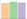
3b(*o*‐BrPh)	*o*‐BrPh	OMe	H	Me		387/4.05	455	0.13	5.55	0.25	75	n.d. ^[^ ^e^ ^]^	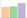
3b(*o*‐MePh)	*o*‐MePh	OMe	H	Me		385/3.60	451	0.43	10.27	0.12	69	−1.50	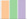
3b(napht‐1‐yl)	napht‐1‐yl	OMe	H	Me		386/3.60	456	0.049	1.54	0.063	n.d. ^[^ ^e^ ^]^	n.d. ^[^ ^e^ ^]^	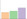
3b(pyridin‐3‐yl)	pyridin‐3‐yl	OMe	H	Me		387/3.76	471	0.55	12.91	0.11	n.d. ^[^ ^e^ ^]^	n.d. ^[^ ^e^ ^]^	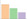
3b(pyridin‐4‐yl)	pyridin‐2‐yl	OMe	H	Me		386/3.77	490	0.46	11.77	0.10	n.d. ^[^ ^e^ ^]^	n.d. ^[^ ^e^ ^]^	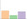
3b(thiophen‐3‐yl)	thiophen‐3‐yl	OMe	H	Me		389/3.85	463	0.020	0.49	0.0083	n.d. ^[^ ^e^ ^]^	n.d. ^[^ ^e^ ^]^	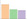
3c(Ph)	Ph	H	OMe	Me		350/4.10	405	0.014	0.45	0.047	n.d. ^[^ ^e^ ^]^	−1.58	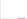
3c(*o*‐BrPh)	*o*‐BrPh	H	OMe	Me		353/4.19	402	0.017	0.28	0.295	n.d. ^[^ ^e^ ^]^	n.d. ^[^ ^e^ ^]^	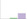
3c(*o*‐MePh)	*o*‐MePh	H	OMe	Me		350/4.26	395	0.009	0.54	0.029	0 ^[^ ^h^ ^]^	−1.61	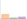
3d(Ph)	Ph	Me	Me	Me		363/3.69	428	0.074	1.34	0.13	41	−1.56	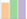
3d(*o*‐BrPh)	*o*‐BrPh	Me	Me	Me		367/3.88	425	0.025	0.45	0.26	32	n.d. ^[^ ^e^ ^]^	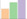
3d(*o*‐MePh)	*o*‐MePh	Me	Me	Me		363/3.67	422	0.043	2.24	0.066	37	−1.57	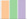
4a	CF_3_	OMe	OMe	Me		396/4.10	476	0.15	2.18	0.34	21	−1.17	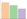
4b	CF_3_	OMe	H	Me		417/3.90	514	0.50	14.97	0.15	20	n.d. ^[^ ^e^ ^]^	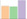

^[a]^The substitution is related to general formula **1** in Figure 1D; *i*Bu is in the position 10.

^[b]^1^st^ Exp. decay (few derivatives show biexponential fluorescence decay, so we report single representative value by calculating the *amplitude‐weighted average* lifetime. *τ*_avg_ = (A_1_τ_1_+A_2_τ_2_)/A_1_+A_2_) Note: We kept the raw biexponential parameters in the Supporting Information S5.1.

^[c]^Values versus SCE; for cyclic voltammograms, see Supporting Information S4.

^[d]^Relative efficiency of the catalysts in reductive model reactions expressed as substrate to product conversion within 4 hours relative to the value for the best catalyst in the deazaalloxazine series. Left column: dehalogenation of **5a** (the best absolute 32 %), middle column: desulfonylation of tosylamide **7a** (the best absolute 86 %), right column: desulfonylation of triflylamide **9a** (the best absolute 100 %); see Supporting Information S9.1 for absolute values of conversions for 4 hours.

^[e]^Not determined.

^[f]^The value should be 1.5 times higher than that for the best deazaalloxazine.

^[g]^The reactions performed in acetonitrile, the best solvent for catalysis with deazaflavins.^[^ ^55^ ^]^

^[h]^Analyzed but no signal detected.

**Scheme 1:**
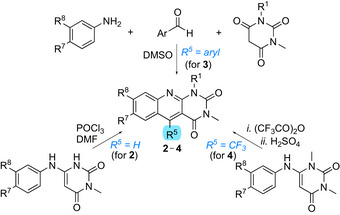
Overview of approaches taken to synthesize 5‐deazaalloxazines **2**–**4**.

Regarding synthesis, three different approaches were used, depending on the substitution at position 5 (see Scheme 1). Unsubstituted derivatives **2** were prepared from the corresponding anilinouracils under the condition of Vilsmeier–Haack formylation.^[^ ^65^ ^]^ The 5‐arylderivatives **3** were prepared using a three‐component reaction developed by our group.^[^ ^66^ ^]^ This reaction starts with an aniline, an aromatic aldehyde and barbituric acid, all of which are mostly commercially available. The 5‐trifluoromethyl‐5‐deazaalloxazines **4** were prepared by trifluoroacetylation of anilinouracil, followed by a condensation reaction using a procedure adapted from the literature (see Supporting Information S2 for synthesis details).^[^ ^67^ ^]^

### 2.2 Photophysical and Electrochemical Properties

As mentioned above, studies investigating the photophysical properties of 5‐deazaalloxazines remain scarce, with only limited data available for a few derivatives in the literature.^[^ ^64^, ^68^, ^69^, ^70^ ^]^ In this study, we conducted a comprehensive investigation of the photophysical and electrochemical behaviour of 5‐deazaalloxazine derivatives in *N*,*N*‐dimethylformamide (DMF), focusing mainly on the characteristics important from the point of view of photoredox catalysis. Thus, we measured UV‐Vis and fluorescence spectra, fluorescence quantum yields, fluorescence lifetimes and cyclic voltammograms (see end of this section for discussion of electrochemical data). The energy of the triplet excited states of flavin derivatives is sufficiently high to be involved in the generation of singlet oxygen. Therefore, we measured the quantum yield of singlet oxygen production. This value also provided evidence regarding the efficiency of the triplet state formation. In selected cases, we measured the triplet state lifetimes (see Table 1 for data and Supporting Information S5.1 for spectra/traces). For comparison, we have also presented data for the isomeric 5‐deazaflavin **1(*o*‐MePh)** (for its structure, see Figure 1D, R = *o*‐Me, R^7^ = R^8^ = OMe, R^10^ = *i*Bu).

**Figure 2:**
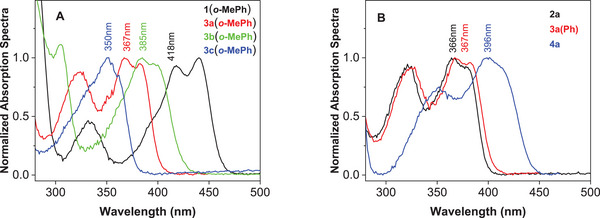
Normalized UV‐Vis spectra of selected 5‐deazaalloxazines in DMF. A) Effect of substitution at positions 7 and 8 and comparison with 5‐deazaflavin **1(*o*‐MePh**). B) Effect of substitution at position 5.

The absorption spectra of 5‐aryl‐5‐deazaflavins **1** display a characteristic band at longer wavelengths, with maxima around 418 nm, as documented for 5‐*o*‐tolyl‐7,8‐dimethoxy‐1,10‐dimethyldeazaisoalloxazine **1(*o*‐MePh)**. In contrast, 5‐aryl‐5‐deazaalloxazines **3** show absorption maxima shifted toward shorter wavelengths (blue shift). This shift is less pronounced for the 7‐methoxy derivatives **3b** (385–389 nm) and more significant for the 8‐methoxy derivatives **3c** (350–353 nm). This difference is caused by the fact, that positions 7 and 8 are not equivalent. An effect of substitution at positions 7 and 8 is documented in Figure 2A for 5‐(*o*‐tolyl) derivatives **3a(*o*‐MePh)**, **3b(*o*‐MePh),** and **3c(*o*‐MePh)** in comparison with the deazaflavin **1(*o*‐MePh)**. Analogously, substitution at positions 7 and 8 influences the absorption spectra of unsubstituted derivative **2**. Substitution with a trifluoromethyl group at position 5 causes a significant red shift, whereas the shift is less pronounced after introducing an aryl group [cf. **2a**, **4a,** and **3a(Ph)**, see Figure 2B]. A noticeable effect of substitution was observed on the molar absorptivity (log ε) of 5‐aryl‐5‐deazaalloxazines **3**, which was within the range of 3.92–4.09 for dimethoxy derivatives **3a** and slightly increased for 8‐methoxy derivatives **3c** (log ε = 4.10–4.26) but decreased for most of the 7‐methoxy‐ **3b** and 7,8‐dimethyl derivatives **3d** (see Table 1). The effect of 5‐substitution on log ε was negligible. Our results by DFT calculations indicate (see Supporting Information S5.2) that in 5‐deazaalloxazines, similarly as in 5‐deazaflavins,^[^ ^70^ ^]^ the lowest‐energy band has significant contribution from pure π,π* transitions.

The fluorescence maxima of 5‐aryl‐5‐deazaalloxazines exhibit a hypsochromic shift compared to the deazaflavin analogue **1(*o*‐MePh)** (see Table 1). The effect of substitution on the fluorescence maxima follows the trends observed in the absorption spectra, with most showing red‐shifted maxima for 7‐methoxy derivatives **3b** and most blue‐shifted maxima for 8‐methoxy derivatives **3c**. Regarding the substitution at position 5, a trifluoromethyl group (in **4a**) causes a significant bathochromic shift of the fluorescence maxima (cf. values for **4a** and **2a**), even surpassing the value 470 nm observed for deazaflavin **1(*o*‐MePh)**. We observed a high fluorescence quantum yield for the 7‐methoxy derivatives **3b**, with values reaching as high as 0.55 for **3b(pyridin‐4‐yl)**. In contrast, very low Φ_F_ values were recorded for derivatives with 5‐*o*‐bromophenyl, 5‐naphthalen‐1‐yl and 5‐thiophen‐3‐yl substitutions. The remaining derivatives exhibited Φ_F_ values in the range of 0.074–0.23. Overall, the Φ_F_ values were lower for all 5‐deazaalloxazines **3** when compared to deazaflavin **1(*o*‐MePh)**. Exceptionally, some 7‐methoxyderivatives **3b** showed longer fluorescence lifetimes (higher τ_F_ values) than the 5‐aryl‐5‐deazaflavins represented by **1(*o*‐MePh)**. The fluorescence decay profiles were well fitted by single‐exponential functions in most cases, whereas a few derivatives with aryl and heteroaryl substituents required a biexponential model. Despite these differences in decay behaviour, the fluorescence spectra were unaffected by changes in the excitation wavelength. Notably, the fluorescence excitation and absorption spectra showed excellent agreement (see Supporting Information S5.1).

Regarding the singlet oxygen quantum yields (*Φ*_Δ_) higher values were observed with *o*‐bromophenyl derivatives, most probably reflecting a heavy atom effect causing higher efficiency of intersystem crossing (ISC). Conversely, naphthalene‐1‐yl and thiophene‐3‐yl derivatives showed small fluorescence quantum yields and were also characterised by a smaller efficiency in terms of singlet oxygen sensitisation. Transient UV–Vis absorption spectra were recorded following pulsed laser excitation of selected 5‐deazaalloxazine derivatives in DMF (see Supporting Information S5.1). The triplet excited states exhibited lifetimes in the microsecond time range and, as expected, they were sensitive to the presence of oxygen. Therefore, the values reported in the table were determined in an argon‐saturated solution.

**Figure 3:**
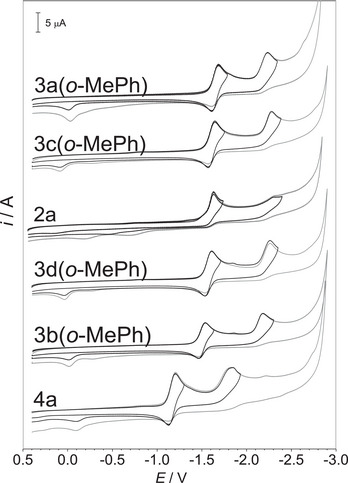
Comparison of cyclic voltammograms in DMF on a glassy carbon electrode for selected 5‐deazaalloxazines. Values of *E* are relative to SCE. For details about the measurements and other data, see Supporting Information S4.

For all of the studied 5‐deazaalloxazines, the typical reduction occurs in two well‐developed one‐electron diffusion‐controlled reduction steps (see Figure 3 for illustration). Cyclic voltammetry confirmed that the first electron transfer is always reversible (the difference between the backward and forward peak potentials is around 70–80 mV). Due to our focus here on radical anion formation as related to photoredox processes, we discuss the first reduction potential in detail (see Table 1).

The reduction potential is affected not only by the nature of substituent (electron donating/withdrawing), but also by the position of substitution. According to expectation the trifluoromethyl group at position 5 causes strong shift of the potential to the less negative values (by 420 mV), as evident by comparing the values for **4a** and **2a**. In contrast, introduction of a 5‐aryl group (c.f. compounds **2a** with **3a**), regardless of its type (c.f. values for derivatives **3a** with phenyl, *o*‐bromophenyl, *o*‐tolyl and thiophen‐3‐yl group), has only small influence on the reduction potential of 5‐deazaalloxazines. As for the substitution in the position 1, we can compare two sets of 5‐aryl‐5‐deazaflavins: **3a(*o*‐BrPh)** versus **3a(*o*‐BrPh)‐H,** and **3a(*o*‐MePh)** versus **3a(*o*‐MePh)‐H**. In both cases methyl in position 1 shifts the reduction potential to more negative values by 40 mV compared to the unsubstituted derivatives. Analogously, but less significantly, 1‐methyl derivative **2a** has more negative potential by 10 mV compared to compound **2a‐H**.

Effect of substitution at positions 7 and 8 methoxy group(s) is interesting and can be easily documented on 5‐aryl‐5‐deazaalloxazines **3**. As expected, the reduction of 7,8‐dimethoxyderivatives **3a** was the most difficult as reflected by the most negative potentials [e. g. −1.65 V for **3a(*o*‐MePh)**]. The easiest reduction, characterized by less negative reduction potentials, was observed for the 7‐methoxyderivatives **3b** [e. g. −1.50 V for **3b(*o*‐MePh)**]. Interestingly, the values for the reduction potentials for 8‐methoxyderivatives **3c** were more negative and much closer to dimethoxy derivative **3a**, as evident from the value −1.61 V for **3c(*o*‐MePh)**. This fact indicates stronger effect of methoxy group in position 8 compared to 7. The same effect of methoxy group(s) in positions 7 and 8 was observed in 5‐unsubstituted series **2a‐H, 2b‐H,** and **2c‐H**.

### 2.3 Screening of the Catalytic Activity

**Scheme 2:**
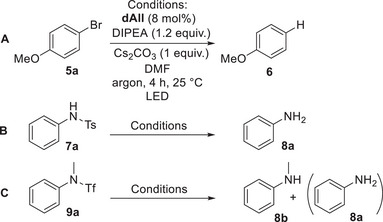
Model reactions for screening of the catalytic activity of deazaalloxazines.

All of the prepared 5‐deazaalloxazines were tested as photocatalysts in the following model reactions, which were chosen with regard to possible practical applications with “inert” substrates: i) dehalogenation of *p*‐bromoanisole (**5a**) to anisole (**6**), ii) desulfonylation of *N‐*tosylaniline (**7a**) to aniline **8a** and iii) desulfonylation of *N*‐methyl‐*N*‐phenyltriflamide (**9a**) resulting in *N*‐methylaniline (**8b**) and in exceptional cases, in aniline (**8a**) formed by subsequent dehalogenation/dealkylation (Scheme 2). In all cases, ethyl diisopropylamine (DIPEA) was used as a sacrificial reducing agent and cesium carbonate served as a base. Reaction mixtures were irradiated using a 400 nm LED as we wanted to use light with the lowest possible photon energy and most catalysts have significant absorption in this region. Nevertheless, with a catalyst that did not absorb or showed only poor absorbance at 400 nm, we also used either 385 nm or 365 nm diodes. In all cases, we evaluated the conversion of the starting material to the product after 4 h of irradiation. Table 1 shows the relative efficiency of the 5‐deazaalloxazines (i.e., the conversion values ​​related to the value for the best deazaalloxazine derivative). The results for **1(*o*‐MePh)** are given for comparison. Absolute values of conversions are presented in Supporting Information S9.1.

The 5‐unsubstituted derivatives **2** and 5‐trifluoromethyl derivatives **4** generally showed low activity in all the model photoreductions. The exception was the 5‐trifluoromethyl derivative **4a**, which was active in the dehalogenation of **5a** but showed only low‐ to‐ moderate activity in desulfonylation reactions. On the contrary, 7,8‐dimethoxy derivatives with 5‐phenyl, 5‐*o*‐bromophenyl and 5‐*o*‐tolyl substituents showed high efficiency in all types of reactions. The exception was the 1‐unsubstituted derivative **3a(*o*‐MePh)‐H**, which was effective only in dehalogenation. The 7‐methoxy derivatives **3b** and 7,8‐dimethyl derivatives **3d** were also mostly effective, with the best performance observed for the *o*‐tolyl derivatives **3b(*o*‐MePh)** and **3d(*o*‐MePh)**. Conversely, the 8‐methoxy derivatives **3c** were completely ineffective even when irradiated with LEDs with a lower wavelength. In general, we concluded that, with the exception of the 8‐methoxy derivative **3c(*o*‐MePh)**, all 5‐*o*‐tolyldeazaalloxazines showed high performance in both the model dehalogenation and desulfonylation reactions. A comparison of the best deazaalloxazines with deazaflavin **1(*o*‐MePh)** shows that deazaflavin exhibits better performance in the dehalogenation of **5a**, which is relatively easy. However, **1(*o*‐MePh)** is not effective in desulfonylations.

### 2.4 Photostability of Deazaalloxazine Catalysts

In addition to their photophysical and electrochemical properties, the photostability of photoredox catalysts is an important factor influencing their efficiency. Thus, for selected derivatives, we studied their photostability by measuring the amount of a 5‐deazaalloxazine remaining in solution after 16 hours of 400 nm LED irradiation (i.e., within the same time horizon used in photoreductions; see Table 2 and Supporting Information S8). The 7,8‐dimethoxy‐ and 7,8‐dimethyldeazaalloxazines **3a** and **3d**, which possess an aryl group at position 5, showed high stability. Interestingly, the dimethoxy derivative **2a**, with no substitution at position 5, was also highly stable. High photostability was also observed for the 8‐methoxy derivative **3c(*o*‐MePh)**, unlike the 7‐methoxy derivative **3b(*o*‐MePh)**, which was relatively unstable. The derivative **4a** with a 5‐CF_3_ group also underwent significant degradation and it was almost absent in the solution after 16 hours of irradiation. A direct comparison of **3a(*o*‐MePh)** with its deazaflavin analogue [**1(*o*‐MePh)**], revealed a greater photostability for the 5‐deazaalloxazines.

**Table 2:** Screening of the photostability of 5‐deazaalloxazines upon LED irradiation.^[^ ^a^ ^]^

5‐Deazaalloxazine	LED Wavelength [nm]	Remaining comp. upon irradiation [%]^[^ ^b^ ^]^
**2a**	400	92
**3a(Ph)**	400	89
**3a(o‐MePh)**	400	78
**3a(thiophen‐3‐yl)**	400	75
**3b(o‐MePh)**	400	37
**3c(o‐MePh)**	365	83
**3d(o‐MePh)**	400	95
**4a**	400	11
**1(*o*‐MePh)**	400	9^[^ ^c^ ^]^

^[a]^ *n*(deazaalloxazine) = 0.004 mmol,16 hours of irradiation, 50 °C, argon atmosphere, 0.6 mL DMF‐*d*_7_.

^[b]^Measured by ^1^H NMR using residual solvent as the internal standard.

^[c]^CD_3_CN.

The photostability of most studied derivatives can be said to predetermine their catalytic efficiency. This is the case for the less stable 5‐trifluoromethyl derivatives **4**, which are ineffective, and for the stable 5‐aryl‐5‐deazaalloxazines from the 7,8‐dimethoxy and 7,8‐dimethyl‐ series (**3a** and **3d**), which are efficient catalysts. Conversely, although photostable, the 5‐unsubstituted derivatives **2** are not catalytically effective. This may be explained by the expected low stability of the corresponding radicals, as previously observed for 5‐unsubstituted deazaflavins, both in nature and in artificial systems.^[^ ^56^, ^71^ ^]^ Similarly, the relatively stable 8‐methoxyderivative **3c(*o*‐MePh)** also showed very poor performance in reductive processes. This might reflect its low ISC quantum yield which can be estimated from low efficiency of singlet oxygen production. The 7‐methoxy derivative **3b(*o*‐MePh)** is a special case, as it shows low photostability. Nevertheless, despite this handicap, it showed relatively high catalytic efficiency in all model reactions. However, this derivative is expected to show little efficiency (as confirmed below) in catalysing the reductions of demanding substrates requiring long reaction times.

**Scheme 3:**
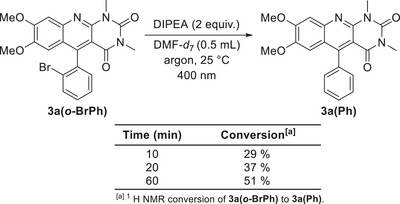
Dehalogenation of **3a(*o*‐BrPh)** and monitoring by ^1^H NMR.

In the case of *o*‐bromo derivative **3a(*o*‐BrPh)**, we observed a dehalogenation reaction upon irradiation (see Scheme 3). Monitoring the reaction mixture with ^1^H NMR spectroscopy revealed the formation of the corresponding dehalogenated compound **3a(Ph)** with a half‐time of about 1 hour (see Supporting Information S8.2 for spectra). After this finding, we inspected the reaction mixtures after reductive transformations catalysed by **3a(*o*‐BrPh)** and found the presence of **3a(Ph)** and the absence of **3a(*o*‐BrPh)** in all cases. Thus, **3a(*o*‐BrPh)** appears to act as a catalyst only at the initial stage of the reactions, with further performance provided by **3a(Ph)**. Interestingly, the debromination of **3a(*o*‐BrPh)** was also observed for the reduction performed electrochemically. Following the first reduction step, the catalytic cleavage of bromide atoms occurred, in addition to the formation of the anion radical. This was manifested by the higher current used in the first reduction step in comparison with the other compounds (see Supplementary Information S4).

### 2.5 The Catalytic Activity Using *p*‐Fluoroanisole

We selected six 5‐deazaalloxazine catalysts to be tested for the dehalogenation of *p*‐fluoroanisole (**5c**), which is one of the most demanding substrates for reductive transformations. This selection was done on the bases of efficacy in model reactions (substances that were in the first quartile in terms of efficacy in all reactions); thus, we selected 7,8‐dimethoxy derivatives **3a(Ph)**, **3a(*o*‐BrPh),** and **3a(*o*‐MePh)**, 7‐methoxyderivative **3b(*o*‐MePh)** and 7,8‐dimethyl derivative **3d(*o*‐MePh)**. For comparison, we selected also **3a(thiophen‐3‐yl)**, the best representative heterocyclic derivatives.

The dehalogenation of fluoroanisole was carried out at both 25 and 50 °C, and the conversion of fluoroanisole to anisole was monitored after 24 hours (Table 3). It should be noted that, in contrast to previous unsuccessful attempts at dehalogenation of 4‐fluoroanisole (**5c**) using 5‐deazaflavins,^[^ ^55^ ^]^ we observed the formation of a significant amount of anisole (**6**), the dehalogenation product, with all 5‐deazaalloxazines used. The most efficient derivative was derivative **3a(*o*‐MePh)**, which achieved up to 70% conversion at 50 °C. A similar efficiency was also observed for **3d(*o*‐MePh)** but only at elevated temperature. In contrast, the derivatives **3b(*o*‐MePh)**, **3a(Ph),** and **3a(thiophen‐3‐yl)** showed low efficiency even at elevated temperatures. One interesting result was achieved with the bromo derivative **3a(*o*‐BrPh)** losing bromine atom by irradiation (see above). The findings that i) **3a(Ph)** showed relatively low efficiency in **3c** dehalogenation and ii) dehalogenation with **3a(*o*‐BrPh)** after 24 hours almost reached the result attained with the best derivative **3a(*o*‐MePh)** suggest that a significant part of the **5c**‐defluorination took place with a proportion of the catalyst still containing the bromine atom in the initial stage of the reaction. The high efficiency of the bromo derivative may be related to its more efficient transition to the triplet state (which is thought to be effective for radical anion formation by PET from DIPEA) due to the heavy atom effect. For comparison, we measured the efficiency of the best 5‐deazaflavin derivative **1(*o*‐MePh)** which was low (see Table 3), probably reflecting its low photostability. Additionally, we tested the best catalyst **3a(*o*‐MePh)** in defluorination of *N‐*(4‐fluorophenyl)acetamide, the other electron‐rich substrate, yielding *N*‐phenylacetamide in 90 % conversion within 24 hours at 25 °C (not shown in Table 3).

**Table 3:** Dehalogenation of 4‐fluoroanisole (**5c**) catalyzed by selected 5‐deazaalloxazines.^[^ ^a^ ^]^

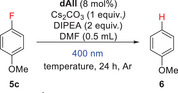		
Catalyst	Conversions [%]^[^ ^b^ ^]^	
	25 °C	50 °C
**3a(Ph)**	24	28
**3a(*o*‐BrPh)**	47	62
**3a(*o*‐MePh)**	52	70
**3a(thiophen‐3‐yl)**	18	33
**3b(*o*‐MePh)**	16	38
**3d(*o*‐MePh)**	13	67
**1(*o*‐MePh)** ^[^ ^c^ ^]^	9	11

^[a]^Reaction conditions: **5c** (0.038 mmol, ca. 73 mM), deazaalloxazine (0.003 mmol, ca. 6 mM), DIPEA (0.072 mmol; ca. 140 mM), DMF (0.5 mL), Cs_2_CO_3_ (0.038 mmol, ca. 73 mM), argon atmosphere, *λ*_exc_ = 400 nm, reaction time 24 hours. See Supporting Information S9.2 for details.

^[b]^Conversions of **5c** to **6** determined by ^1^H NMR.

^[c]^In acetonitrile.

### 2.6 Preparative Experiments – Applications

Based on the previous results, we selected **3a(*o*‐MePh)** as the most potent 5‐deazaalloxazine catalyst. It shows the best performance in fluoroanisole (**5c**) dehalogenation and it is also characterised by relatively high stability. Based on the redox potential of **3a(*o*‐MePh)**/ **[3a(*o*‐MePh)]^•–^** couple in the ground state and the spectra of the radical anion **[3a(*o*‐MePh)]^•–^**, we estimated its excited state oxidation potential to be –3.33 V (see Supporting Information S6 for details). This value characterizes the power of excited **[3a(*o*‐MePh)]^•–^** in photoreductions.

Here, we demonstrated the usefulness of the catalyst on the C─P coupling reaction between selected aryl halides **5** and trimethyl phosphite to yield the corresponding dimethyl arylphosphonates **10**. Regarding aryl halides, we focused mainly on electron‐rich and therefore difficult‐to‐reduce halogen anisoles **5**. First, we performed an initial screening of the reaction conditions (see Supporting Information S11), which showed, as expected, the importance of using an excess of trimethyl phosphite to minimise the formation of undesired dehalogenation side‐products. We also noticed a positive effect of elevated temperature on the phosphonylation yields. Note that the formation of the dehalogenation product usually accompanies arylations provided by photoredox catalysis;^[^ ^8^ ^]^ the dehalogenation product is formed by the reaction of an aryl radical with a hydrogen source (DIPEA or solvent) via hydrogen atom transfer (HAT^[^ ^72^ ^]^). We ruled out anisole formation by dephosphonylation of the product **10** by the following independent experiment: Irradiation of dimethyl 4‐methoxyphenylphosphonate (**10a**) under conditions of coupling reaction did not result in the formation of anisole.

**Scheme 4:**
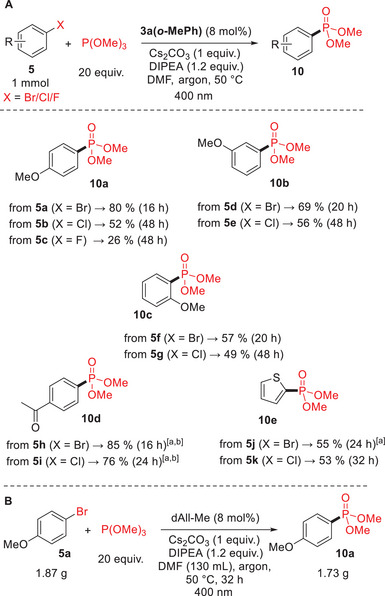
A) Preparative phosphonylation reactions with selected aryl halides at a 1 mmol scale; B) Phosphonylation at a 10 mmol scale. ^[a]^ 25 °C; ^[b]^ Yields determined by GC‐MS.

To achieve the highest yields of arylphosphonates **10**, we carried out our own preparative phosphonylations of halogen anisoles **5a**‐**k** at 50 °C with 20 equivalents of trimethyl phosphite (Scheme 4).

However, the phosphonylations of the more reactive electron‐poor acetyl derivatives **5** **h** and **5i** and 2‐bromothiophene **5j** were carried out at 25 °C, as we observed a large amount of dehalogenated product at elevated temperature (data not shown). The results demonstrate that the catalyst **3a(*o*‐MePh)** enables phosphonylation regardless of the relative position of the halogen and methoxy group and achieves good to high preparative yields. The reaction can even be carried out with aryl fluoride, as demonstrated with **5c**, albeit with a lower yield that did not increase even when the reaction time was doubled. The phosphonylation of 4‐bromoanisole (**5a**) was also performed on a large scale and yielded 1.73 g of phosphonate **10a** from 1.87 g of **5a** (see Supporting Information S11.2 for experimental details).

**Scheme 5:**
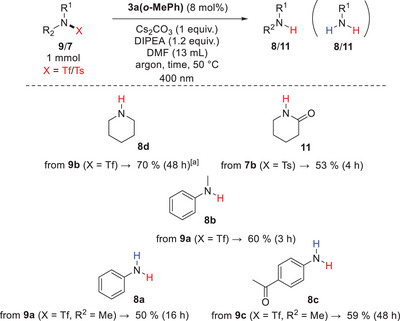
Preparative desulfonylation reactions with selected sulphonamides at a 1 mmol scale. ^[a]^ Yield determined by ^1^H NMR.

To demonstrate the effectiveness of the optimized catalyst **3a(*o*‐MePh)** in desulfonylation reactions, we also performed reactions with a few selected tosylamides and triflylamides (trifluoromethanesulfonyl amides), which had been challenging using our previously described catalyst **3a(*o*‐BrPh)**.^[^ ^63^ ^]^ An example of this type of substrate is 1‐triflylpiperidine (**9b**), which did not undergo nitrogen deprotection using **3a(*o*‐BrPh)**. On the contrary, a significant amount of product was observed for the desulfonylation of **9b** catalyzed by **3a(*o*‐MePh)**. In a preparative setup, this catalyst was able to remove the triflyl group from piperidine with a conversion of up to 70% (Scheme 5). Good yields were also achieved for the detosylation of lactam **7b** or the detriflylation of **9a** to form *N*‐methylaniline **8b**, performed within shorter reaction time (3 hours), compared to **3a(*o*‐BrPh)**. At longer reaction times, we observed dealkylation following detriflylation reaction, as already observed with the **3a(*o*‐BrPh)** catalyst. This is demonstrated for **9a**, which is converted to aniline **8a** after 16 hours of irradiation and for the transformation of **9c** to **8c** after 48 hours. Since dealkylation occurs via a coupled oxidative mechanism,^[^ ^63^ ^]^ these reactions indicate the possibility of using the optimized **3a(*o*‐MePh)** catalyst in oxidative processes as well (see Supporting Information S10 for experimental details).

## 3 Conclusion

In this work, we have presented 5‐deazaalloxazines as suitable catalysts for reductive photoredox catalysis. In contrast to isomeric 5‐deazaflavins, these substances exhibit significantly higher photostability and have a more negative reduction potential even in the ground state (by approximately 300 mV). The reduction potential depends on the substitution of the deazaalloxazine core and reaches the most negative values ​​(down to −1.65 V versus SCE) with 5‐aryl derivatives substituted with a methoxy group at both positions 7 and 8. One disadvantage of 5‐deazaalloxazines compared to deazaflavins is their blue‐shifted absorption maximum down to 350 nm. Fortunately, we showed that this shift can be tuned by substitution. In particular, the 7,8‐dimethoxyderivatives and 7‐methoxyderivatives have an absorption maximum shifted up to 372 and 385 nm, respectively, and both derivatives absorb significantly even in the visible region, thereby allowing the use of 400 nm LEDs in photoredox catalysis. The greatest spectrum shift to the red region was observed for 5‐trifluoromethyl derivatives, but they are poorly photostable and their reduction potential is also shifted to less negative values ​​(E = −1.17 V versus SCE), meaning that they are not suitable for photoreductions. Deazaalloxazines have a relatively high quantum yield of ISC, which can be estimated from the measured quantum yield of singlet oxygen sensitization, which reaches values ​​higher than 0.2. The lifetime of the triplet state, which is probably involved in the photoinduced electron transfer (PET) from the sacrificial electron donor (DIPEA), is in the tens of microseconds in deaerated solutions.

As shown in model reactions, including the dehalogenation of 4‐fluoroanisole (**5c**), the most efficient photoredox catalysts are 7,8‐dimethoxy derivatives **3a**, preferably substituted at position 5 with an *o*‐tolyl group. The 7‐methoxy‐5‐*o*‐tolyl derivative **3b(*o*‐MePh)** and the derivatives **3a(*o*‐BrPh)** with an *o*‐bromophenyl group showed high efficiency, but only in reactions that proceeded for a short time. In reactions with more demanding substrates, such as **5c**, neither **3a(*o*‐BrPh)** nor **3b(*o*‐MePh)** were effective due to their low photostability. The best catalyst turned out to be **3a(*o*‐MePh)** derivative, whose practical applicability we successfully demonstrated for the reductive arylations of trimethyl phosphite with the formation of a C─P bond in phosphonates **10** and on the desulfonylations of sulphonamides **7** and **9**. Both of these reactions are desirable transformations in organic synthesis, indicating that our catalyst represents a promising tool.

We propose that deazaalloxazines **3** participate in reductive reactions by the conPET mechanism (see Figure 1E). During the first PET, **3** in its triplet excited state accepts an electron from an electron donor (DIPEA). Following excitation, the resulting radical anion generates a strongly reductive species **3^•–^** which is characterized by its very negative potential (*E*_ox_* = −3.3 V versus SCE). However, questions remain regarding the mechanism by which the electron is subsequently transferred to the substrate. In principle, this may involve the formation of complexes with the substrate or the generation of a solvated electron, which is its own reducing agent. In the case of flavin derivatives, cases have been reported for the formation of a solvated electron involved in reductions, such as in photolyase models^[^ ^73^ ^]^ or in the regeneration of 5‐deazaflavinium salts during photooxidations.^[^ ^49^, ^74^ ^]^ This suggests that a mechanism involving a solvated electron could be involved. A detailed study of the mechanism, as well as the study of other possible applications of photoreductions driven by 5‐deazaalloxazines, is currently underway in our laboratories.

## Supporting Information

The authors have cited additional references within the Supporting Information.^[^ ^75^, ^76^, ^77^, ^78^, ^79^, ^80^, ^81^, ^82^, ^83^, ^84^ ^]^

## Conflict of Interest

The authors declare no conflict of interest.

## Supporting information



Supporting Information

## Data Availability

The data that support the findings of this study are openly available in Zenodo at 10.5281/zenodo.17140607.
