# Supplementary material for: Deazaalloxazines – Flavin Derivatives That Provide Reductive Photoredox Catalysis with Inert Substrates
Source: Chemistry. 2025 Nov 19;31(71):e02897. doi: 10.1002/chem.202502897 (PMC12734692; doi:10.1002/chem.202502897)
Supplement: Supplementary file 1 — Supporting Information [file CHEM-31-e02897-s001.pdf]

S1. General procedures 2
S2. Synthesis of deazaalloxazines and substrates 4
2.1 Synthesis of 5-H deazaalloxazines 4
2.2 Synthesis of 5-aryldeazaalloxazines 9
2.3 Synthesis of 5-trifluormethyldeazaalloxazines 14
2.4 Synthesis of substrates 16
S3. Spectroelectrochemical measurements 17
S4. Cyclic voltammograms of deazaalloxazines 19
S5. Photophysical properties 22
5.1 Experimental data 22
5.2 Theoretical spectra 56
S6. Estimation of E^*_{ox} of anion radical 59
S7. Experimental setup for Photocatalytic experiments 60
S8. Photostability of deazaalloxazines 61
8.1 Photostability of selected deazaalloxazines 61
8.2 Photocatalytic debromination of deazaalloxazine 3a(*o*-BrPh) 61
8.3 Investigation of stability of deazaalloxazine 3a(*o*-MePh) during reaction 62
S9. Initial screening of catalytic activity 63
9.1 Photocatalytic model reaction of deprotection or dehalogenation by deazaalloxazine catalysts 63
9.2 Photocatalytic reduction by selected deazaalloxazines 66
S10. Reductive desulfonylations 67
10.1 Initial screening on analytical scale 67
10.2 Reactions on preparative scale 69
S11. Photocatalytic *coupling* reaction with $P(OMe)_3$ 72
11.1 Initial screening on analytical scale 72
11.2 Preparative experiments 74
S12. NMR spectra of deazaalloxazines 78
S13. NMR spectra of isolated products of photoreductive deprotection 97
S14. NMR spectra of isolated products of photoreductive coupling 104
S15. Carthesian Coordinates 108
S16. References 114

S1. GENERAL PROCEDURES

Materials and Instrumentation: chemicals were purchased at Sigma-Aldrich and Fluorochem. The solvents were purified and dried using standard procedures. Commercially obtained reagents were used as received without further purification unless otherwise stated. Thin layer chromatography (TLC) analyses were carried out on DC Alufolien Kieselgel 60 F254 (Merck). The compounds were visualised with UV light (254 and 366 nm). Flash chromatography was carried out using Büchi Pure C-810 at Silica 40 µm irregular column at increased pressure. Compound structures were drawn and named using ChemDraw. **Nuclear magnetic resonance** (NMR) spectra were recorded on a Agilent 400-MR DDR2 (399.94 MHz for ^{1}H, 100.58 MHz for ^{13}C, 376.50 MHz for ^{19}F), or JNM-ECZL400S spectrometer (JEOL Ltd., (399.94 MHz for ^{1}H, 100.58 MHz for ^{13}C, 376.50 MHz for ^{19}F), or Bruker Avance III™ HD 400 and Bruker Avance III™ HD 400 MHz Prodigy (100.60 MHz for ^{13}C) at 298 K unless otherwise indicated. Data for ^{1}H NMR are reported as follows: chemical shift (δ ppm), multiplicity (s = singlet, d = doublet, t = triplet, q = quartet, m = multiplet, dd = doublet of doublets, dt = doublet of triplets, br = broad etc.), coupling constant (Hz), and integration. All NMR spectra were processed and assigned using MestreNova. **High-resolution mass spectra** were obtained on Q-Tof Micro (Waters), equipped with a quadrupole and time-of-flight (TOF) analyser and a multichannel plate (MCP) detector. The melting points were measured on a Boetilus melting point apparatus and are uncorrected.

UV–Vis absorption spectra were recorded using a Shimadzu UV-2550-230V spectrophotometer. Room-temperature **emission and excitation spectra** were obtained with a Horiba Jobin Yvon 221 Fluorolog-3 spectrofluorometer. Fully corrected excitation–emission matrices were acquired using the A-TEEMs™ (absorbance–transmission and fluorescence–excitation–emission matrix) method on an Aqualog-UV-800 system (Horiba Jobin Yvon). **Time-resolved fluorescence measurements** were carried out on a PicoQuant FluoTime 300 spectrofluorometer, with emission detected using a Hamamatsu microchannel plate photomultiplier tube coupled to a single-photon timing system. **Steady-state singlet oxygen emission spectra** were recorded on the FluoTime 300 system using a lamp as the excitation source. **Triplet states** were generated and analysed via laser flash photolysis using a

nanosecond Q-switched Nd: YAG laser in combination with an optical parametric oscillator (OPO) operates at selected wavelength.

Spectro-electrochemistry measurements were performed in an OTTLE Cell3 (Optically transparent thin-layer electro-chemical cell) [1], pathlength = 0.02 cm, working electrode: Pt minigrid, counter electrode: Pt: minigrid, pseudo reference electrode: Ag wire. Samples were prepared by degassing a solution via argon bubbling for several minutes. UV-Vis spectra were recorded on Agilent Cary 8454 spectrometer. **Cyclic voltammetry** (CV) was performed with Autolab-Metrohm PGSTAT101 in a 10 ml cell with a three-electrode system: working electrode - glassy carbon (Ø 3 mm); auxiliary electrode - Pt plate; reference electrode - calomel electrode (SCE), which was separated from the solution by a salt bridge; supporting electrolyte: Me4NPF6 (Sigma), in 0.1 M DMF solution, which was degassed with argon. A ferricen/ferrocen pair with a potential of 0.45 V against SCE in DMF was used as an internal standard. Absorption and emission spectra and cyclic voltamograms were processed using Microsoft Excel and Origin 2018 (OriginLab). **Photochemical Setup**: The reactions were carried out in borosilicate glass vessels using commercial LEDs as light sources: LED Engin, 1.35 W@700 mA, 400 nm; LUXEON, 1.03 W@700 mA, 448 nm; LED Engin, 1.1 W@700 mA, 385 nm; 365 nm. For the large-capacity preparative system, the following LEDs were used: novaLIGHT T-LED100 W, 405 nm.

Quantum Mechanical Calculations: Information on the electronic structure and geometry of the DAll compounds was obtained with the use of quantum chemical density functional theory (DFT) calculations. The calculations were performed using the B3LYP functional[1] along with a correlation-consistent basis set with triple-zeta (VTZ) polarization and diffused functions added, aug-cc-pVTZ[2]. Geometry optimization of the DAll molecules at this level of theory was performed. The vertical excitation energies and oscillator strengths were calculated using the time-dependent (TD) approach as implemented in the Gaussian16 program[3]. Predicted lowest-energy singlet-singlet transitions of the DAll compounds, S0 → Si, were calculated for the ground-state geometry using the TD-DFT with the B3LYP/aug-cc-pVTZ level of theory.

S2. SYNTHESIS OF DEAZAALLOXAZINES AND SUBSTRATES

The synthesis of the already published deazaalloxazines is not described in this supplementary information (Table S1).

Table S1: Deazaalloxazines, which have synthesis described in the following references.

**dAll** 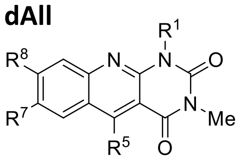	**R⁵**	**R⁷**	**R⁸**	**R¹**	**Ref.**
**3a(Ph)**	Ph	OMe	OMe	Me	4
**3a(*o*-BrPh)**	*o*-BrPh	OMe	OMe	Me	4
**3a(*o*-BrPh)-H**	*o*-BrPh	OMe	OMe	H	5
**3a(*p*-BrPh)**	*p*-BrPh	OMe	OMe	Me	4
**3a(*o*-MePh)**	*o*-MePh	OMe	OMe	Me	4
**3b(Ph)**	Ph	OMe	H	Me	4
**3b(*o*-BrPh)**	*o*-BrPh	OMe	H	Me	4
**3b(*o*-MePh)**	*o*-MePh	OMe	H	Me	4
**3c(Ph)**	Ph	H	OMe	Me	4
**3c(*o*-BrPh)**	*o*-BrPh	H	OMe	Me	4
**3c(*o*-MePh)**	*o*-MePh	H	OMe	Me	4
**3d(Ph)**	Ph	Me	Me	Me	4
**3d(*o*-BrPh)**	*o*-BrPh	Me	Me	Me	4
**3d(*o*-MePh)**	*o*-MePh	Me	Me	Me	4

2.1 Synthesis of 5-H deazaalloxazines

General procedure A1 (ref.[6])

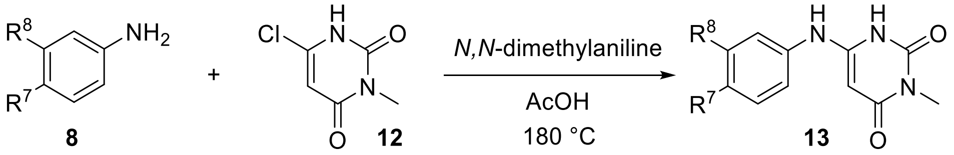

A mixture of substituted aniline **8** (1 equiv.) and 6-chloro-3-methyluracil (**12**) (1 equiv.) was dissolved in a mixture of *N,N*-dimethylaniline (5-10 mL) and acetic acid (0.25-0.5 mL). The reaction mixture was placed in a heated oil bath (180 °C) and then heated for 2 hours at 180 °C. Then the product was precipitated in ether and then vacuum filtered. The final step was the recrystallization of the resulting product from ethanol.

General procedure A2

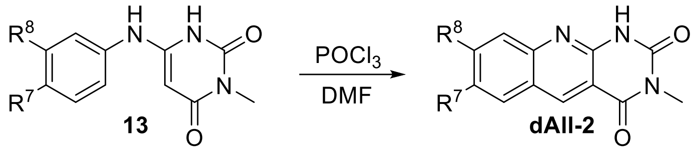

The first step of the reaction was the preparation of the formylation reagent. DMF (1 mL) was cooled in an ice bath to 0 °C and then $POCl_3$ (1.3 equiv.) was added. The mixture was left to warm to room temperature for 15 min while stirring and placed in the refrigerator for 1 h.

Subsequently, the Vilsmeyer-Haack reagent was added to the prepared anilinouracil **13** (1 equiv.) dissolved in DMF (5 mL). The reaction mixture was stirred for 1 hour at room temperature and if precipitation of the product did not occur it was subsequently heated for 1 hour at 110 °C. The resulting **dAII-2** product was then filtered and washed with 2-propanol.

*6-[(3,4-Dimethoxyphenyl)amino]-3-methylpyrimidin-2,4(1H,3H)-dione (**13a**)*

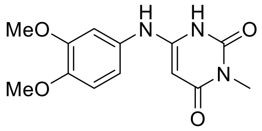

Prepared according to a **general procedure A1** from 3,4-dimethoxyaniline (**8g**) (1.50 g, 9.8 mmol) and uracil **12** (1.57 g, 9.8 mmol) in 10 mL *N,N*-dimethylaniline and 0.5 mL AcOH.

Yield: 65 % (1.76 g) grey solid.

^{1}H NMR (400 MHz, DMSO-d_6) δ 10.48 (s, 1H), 7.99 (s, 1H), 6.95 (d, *J* = 8.6 Hz, 1H), 6.81 (d, *J* = 2.4 Hz, 1H), 6.75 (dd, *J* = 8.5, 2.5 Hz, 1H), 4.65 (s, 1H), 3.75 (s, 3H), 3.73 (s, 3H), 3.05 (s, 3H).

*7,8-Dimethoxy-3-methylpyrimido[4,5-b]quinoline-2,4(1H,3H)-dione (**2a-H**)*

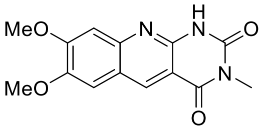

Prepared according to a **general procedure A2** from anilinouracil **13a** (0.14 g, 0.5 mmol) and $POCl_3$ (70 µL, 0.7 mmol) without heating.

Yield: 34 % (0.10 g) pale yellow crystalline powder.

Melting point: > 350 °C (from 2-propanol).
^{1}H NMR (400 MHz, DMSO-d_6) δ 11.80 (s, 1H), 8.81 (s, 1H), 7.56 (s, 1H), 7.18 (s, 1H), 3.96 (s, 3H), 3.89 (s, 3H), 3.28 (s, 3H).
^{13}C NMR (101 MHz, TFA-*d*) δ 161.2, 160.6, 151.3, 150.5, 145.9, 143.3, 137.4, 120.9, 108.1, 107.2, 98.4, 56.6, 55.8, 27.7.
HR-MS (APCI+) *m/z:* calculated for $C_{14}H_{13}N_3O_4$ [M+H$^+$]: 288.0984, **observed**: 288.0980.

*6-[(4-Methoxyphenyl)amino]-3-methylpyrimidine-2,4(1H,3H)-dione (**13b**)*

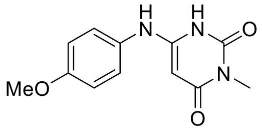

Prepared according to a **general procedure A1** from 4-methoxyaniline (**8h**) (1.00 g, 8.1 mmol) and uracil **12** (1.30 g, 8.1 mmol) in 10 mL *N,N*-dimethylaniline and 0.5 mL AcOH.

Yield: 78 % (1.55 g) grey solid.

^{1}H NMR (400 MHz, DMSO-d_6) δ 10.49 (s, 1H), 7.99 (s, 1H), 7.19 – 7.10 (d, 2H), 7.00 – 6.91 (d, 2H), 3.75 (s, 3H), 3.05 (s, 3H).

*7-Methoxy-3-methylpyrimido[4,5-b]quinoline-2,4(1H,3H)-dione (**2b-H**)*

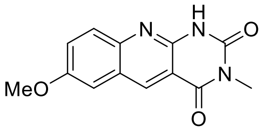

Prepared according to a **general procedure A2** from anilinouracil **13b** (0.25 g, 1.0 mmol) and $POCl_3$ (140 µL, 1.3 mmol).

Yield: 44 % (0.11 g) yellow crystalline powder.

Melting point: 281 – 283 °C (from 2-propanol).
^{1}H NMR (400 MHz, DMSO-d_6) δ 11.88 (s, 1H), 8.94 (s, 1H), 7.78 (dt, *J* = 9.3, 0.6 Hz, 1H), 7.61 (d, *J* = 2.9 Hz, 1H), 7.52 (dd, *J* = 9.2, 2.9 Hz, 1H), 3.89 (s, 3H), 3.28 (s, 3H).
^{13}C NMR (101 MHz, TFA-*d*) δ 162.6, 162.4, 152.5, 150.5, 145.5, 135.9, 134.7, 128.3, 122.3, 112.6, 110.5, 57.4, 29.9.
HR-MS (APCI+) *m/z:* calculated for $C_{13}H_{11}N_3O_3$ [M+H$^+$]: 258.0878, **observed**: 258.0877.

*6-[(3-Methoxyphenyl)amino]-3-methylpyrimidine-2,4(1H,3H)-dione (**13c**)*

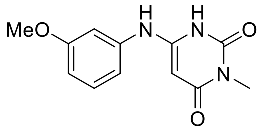

Prepared according to a **general procedure A1** from 3-methoxyaniline (**8i**) (1.00 g, 8.1 mmol) and uracil **12** (1.30 g, 8.1 mmol) in 10 mL *N,N*-dimethylaniline and 0.5 mL AcOH.

Yield: 75 % (1.50 g) white solid.

^{1}H NMR (400 MHz, DMSO-d_6) δ 10.53 (s, 1H), 8.28 (s, 1H), 7.33 – 7.24 (m, 1H), 6.82 – 6.75 (m, 1H), 6.75 – 6.70 (m, 2H), 4.89 (s, 1H), 3.75 (s, 3H), 3.06 (s, 3H).

*8-Methoxy-3-methylpyrimido[4,5-b]quinoline-2,4(1H,3H)-dione (**2c-H**)*

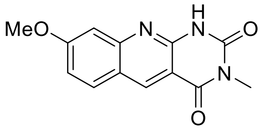

Prepared according to a **general procedure A2** from anilinouracil **13c** (0.12 g, 0.50 mmol) and $POCl_3$ (70 µL, 0.7 mmol) without heating.

Yield: 75 % (0.10 g) white crystalline powder.

Melting point: 340 – 343 °C (from 2-propanol).
^{1}H NMR (400 MHz, DMSO-*d$_6$*) δ 11.90 (s, 1H), 8.95 (s, 1H), 8.07 (d, *J* = 9.8 Hz, 1H), 7.23 – 7.15 (m, 2H), 3.94 (s, 3H), 3.28 (s, 3H).
^{13}C NMR (101 MHz, TFA-*d*) δ 170.4, 160.6, 150.5, 148.0, 145.2, 141.8, 133.1, 122.5, 120.0, 106.5, 98.4, 56.0, 27.7.
HR-MS (APCI+) *m/z:* calculated for $C_{13}H_{11}N_3O_3$ [M+H$^+$]: 258.0878, **observed**: 258.0874.

*6-[(3,4-Dimethylphenyl)amino]-3-methylpyrimidine-2,4(1H,3H)-dione (**13d**)*

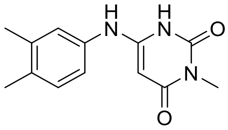

Prepared according to a **general procedure A1** from 3,4-dimethylaniline (**8j**) (0.50 g, 4.1 mmol) and uracil **12** (0.66 g, 4.1 mmol) in 5 mL *N,N*-dimethylaniline and 0.25 mL AcOH.

Yield: 57 % (0.58 g) white solid.

^{1}H NMR (400 MHz, DMSO-*d$_6$*) δ 10.43 (s, 1H), 8.06 (s, 1H), 7.14 (d, *J* = 8.0 Hz, 1H), 6.98 (d, *J* = 2.3 Hz, 1H), 6.92 (dd, *J* = 8.0, 2.4 Hz, 1H), 4.74 (s, 1H), 3.05 (s, 3H), 2.21 (s, 3H), 2.19 (s, 3H).

*3,7,8-Trimethylpyrimido[4,5-b]quinoline-2,4(1H,3H)-dione (**2d-H**)*

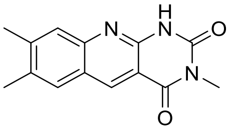

Prepared according to a **general procedure A2** from anilinouracil **13d** (0.12 g, 0.5 mmol) and $POCl_3$ (70 µL, 0.7 mmol).

Yield: 63 % (0.08 g) pale yellow crystalline powder.

Melting point: > 350 °C (from 2-propanol).
^{1}H NMR (400 MHz, DMSO-*d$_6$*) δ 11.88 (s, 1H), 8.89 (s, 1H), 7.89 (s, 1H), 7.65 (s, 1H), 3.28 (s, 3H), 2.45 (s, 3H), 2.39 (s, 3H).
^{13}C NMR (101 MHz, TFA-*d*) δ 160.5, 154.8, 150.4, 148.7, 144.5, 141.8, 137.1, 130.1, 123.2, 118.2, 108.9, 27.7, 19.8, 17.9.
HR-MS (APCI+) *m/z:* calculated for $C_{14}H_{13}N_3O_2$ [M+H$^+$]: 256.1086, **observed**: 256.1081.

*7,8-Dimethoxy-1,3-dimethylpyrimido[4,5-b]quinoline-2,4(1H,3H)-dione (**2a**)*

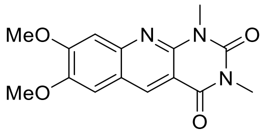

Prepared according to a **general procedure A2** from anilinouracil **17a** (0.29 g, 1.0 mmol) (preparation viz. chapter 2.3) and $POCl_3$ (140 µL, 1.3 mmol).

Yield: 17 % (0.05 g) pale yellow crystalline powder.

Melting point: 265 – 267 °C (from 2-propanol).
^{1}H NMR (400 MHz, DMSO-d_6) δ 8.89 (s, 1H), 7.60 (s, 1H), 7.32 (s, 1H), 3.99 (s, 3H), 3.91 (s, 3H), 3.66 (s, 3H), 3.34 (s, 3H).
^{13}C NMR (101 MHz, TFA-*d*) δ 163.0, 162.4, 153.4, 152.0, 148.3, 147.4, 139.8, 122.4, 110.3, 110.1, 101.1, 58.8, 57.9, 32.4, 30.9.
HR-MS (APCI+) *m/z:* calculated for $C_{15}H_{15}N_3O_4$ [M+H$^+$]: 302.1141, **observed**: 302.1139.

*1,3-Dimethylpyrimido[4,5-b]quinoline-2,4(1H,3H)-dione (**2e**)*

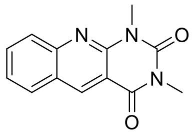

Prepared according to a **general procedure A2** from anilinouracil **17e** (0.23 g, 1.0 mmol) (preparation viz. chapter 2.3) and $POCl_3$ (140 µL, 1.3 mmol).

Yield: 68 % (0.16 g) pale yellow crystalline powder.

Melting point: 205 – 207 °C (from 2-propanol).
^{1}H NMR (400 MHz, DMSO-d_6) δ 9.12 (s, 1H), 8.21 (ddd, *J* = 8.6, 1.4, 0.6 Hz, 1H), 7,94 (dt, *J* = 1.4, 0.7 Hz, 1H), 7.89 (ddd, *J* = 8.5, 6.7, 1.5 Hz, 1H), 7.58 (ddd, *J* = 8.1, 6.7, 1.3 Hz, 1H), 3.67 (s, 3H), 3.35 (s, 3H).
^{13}C NMR (101 MHz, $CDCl_3$) δ 161.5, 151.7, 150.0, 148.5, 140.2, 133.3, 129.4, 128.2, 125.9, 124.8, 111.0, 29.7, 28.6.
HR-MS (APCI+) *m/z:* calculated for $C_{13}H_{11}N_3O_2$ [M+H$^+$]: 242.0929, **observed**: 242.0927.

2.2 Synthesis of 5-aryldeazaalloxazines

General procedure C (Ref.[4, 5])

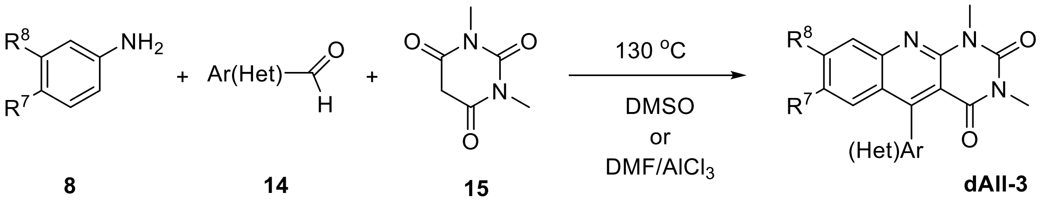

8 **14** **15** **dAll-3**

A mixture of substituted aniline **8** (1 equiv.), aromatic aldehyde **14a-e** (1 equiv.), and *N,N´*-dimethylbarbituric acid **15** (1 equiv.) was dissolved in dry solvent (6 mL). The solution was heated from 15 hours to 2 days at 130 °C. After cooling mixture to the room temperature, the precipitated product **dAll** was filtered and washed by 2-propanol**.**

7,8-Dimethoxy-3-methyl-5-(o-tolyl)pyrimido[4,5-b]quinoline-2,4(1H,3H)-dione
(3a(o-MePh)-H)

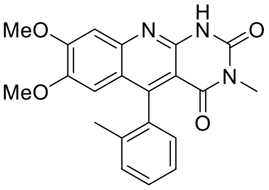

Prepared according to a **general procedure C** from 3,4-dimethoxyaniline (**8g**) (0.46 g, 3.0 mmol), 2-methylbenzaldehyde (**14a**) (0.36 g, 3.0 mmol), and *N*-methylbarbituric acid (**15-H**) (0.42 g, 3.0 mmol) in 6 mL of dry DMF with catalytic amount of $AlCl_3$.

Reaction time: 15 hours.
Yield: 45 % (0.51 g) pale yellow crystalline powder.

Melting point: 278 – 280 °C (from 2-propanol).
^{1}H NMR (400 MHz, DMSO-d_6) δ 11.82 (s, 1H), 7.37 (dd, *J* = 6.3, 1.5 Hz, 2H), 7.29 (td, *J* = 6.9, 2.2 Hz, 1H), 7.22 (s, 1H), 7.01 (dd, *J* = 7.2, 1.2 Hz, 1H), 6.31 (s, 1H), 3.99(s, 3H), 3.97 (s, 3H), 3.50 (s, 3H), 3.12 (s, 3H).
^{13}C NMR (151 MHz, TFA-*d*) δ 165.9, 162.4, 154.3, 153.7, 153.2, 146.7, 139.2, 136.8, 135.7, 133.2, 132.8, 129.0, 128.6, 124.4, 109.4, 108.1, 101.1, 59.3, 58.0, 30.4, 20.4.
HR-MS (APCI+) *m/z:* calculated for $C_{21}H_{19}N_3O_4$ [M+H$^+$]: 378.1454, **observed**: 378.1457.

7,8-dimethoxy-1,3-dimethyl-5-(naphtalene-1-yl)pyrimido[4,5-b]quinoline-2,4(1H,3H)-dione
(3a(napht-1-yl))

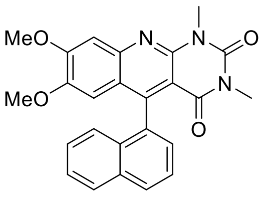

Prepared according to a **general procedure C** from 3,4-dimethoxyaniline (**8g**) (0.46 g, 3.0 mmol), 1-naphthaldehyde (**14b**) (0.47 g, 3.0 mmol), and barbituric acid **15** (0.47 g, 3.0 mmol) in 6 mL of dry DMSO.

Reaction time: 15 hours.
Yield: 62 % (0.79 g) white crystalline powder.

Melting point: 336 – 338 °C (from 2-propanol).
^{1}H NMR (400 MHz, DMSO-d_6) δ 8.04 (dd, *J* = 8.2, 5.4 Hz, 2H), 7.64 (dd, *J* = 8.3, 7.0 Hz, 1H), 7.50 (t, *J* = 7.5 Hz, 1H), 7.42 (s, 1H), 7.29 (dd, *J* = 12.9, 7.0 Hz, 2H), 7.11 (d, *J* = 8.4 Hz, 1H), 6.20 (s, 1H), 4.00 (s, 3H), 3.76 (s, 3H), 3.30 (s, 3H), 3.08 (s, 3H).
^{13}C NMR (151 MHz, DMSO-d_6) δ 162.4, 160.3, 159.1, 150.8, 149.9, 145.9, 136.5, 133.61, 131.1, 130.2, 130.0, 128.6, 127.3, 126.8, 124.9, 124.6, 123.1, 121.8, 107.4, 107.0, 98.8, 56.6, 55.1, 30.6, 28.7.
HR-MS (APCI+) *m/z:* calculated for $C_{25}H_{21}N_3O_4$ [M+H$^+$]: 428.1610, **observed**: 428.1608.

7,8-Dimethoxy-1,3-dimethyl-5-(pyridine-4-yl)pyrimido[4,5-b]quinoline-2,4(1H,3H)-dione ***(3a(pyridin-4-yl))***

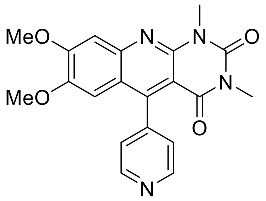

Prepared according to a **general procedure C** from 3,4-dimethoxyaniline (**8g**) (0.15 g, 1 mmol), pyridine-4-carbaldehyde (**14c**) (0.11 g, 1 mmol), and barbituric acid **15** (0.16 g, 1 mmol) in 2 mL of dry DMSO.

Reaction time: 15 hours.
Yield: 49 % (0.18 g) pale yellow crystalline powder.

Melting point: 336 – 339 °C (from 2-propanol).
^{1}H NMR (400 MHz, TFA-*d*) δ 9.05 (d, *J* = 8.1 Hz, 2H), 8.06 (d, *J* = 8.3 Hz, 2H), 7.74 (s, 1H), 6.52 (s, 1H), 4.12 (s, 3H), 3.99 (s, 3H), 3.73 (s, 3H), 3.34 (s, 3H).
^{13}C NMR (151 MHz, $CDCl_3$) δ 161.0, 155.8, 151.5, 149.9 (2C), 149.6, 148.4, 147.9, 147.3, 146.6, 122.9 (2C), 119.8, 106.9, 105.5, 104.0, 56.6, 56.0, 30.1, 28.6.
HR-MS (APCI+) *m/z:* calculated for $C_{20}H_{18}N_4O_4$ [M+H$^+$]: 379.1406, **observed**: 379.1407.

7,8-Dimethoxy-1,3-dimethyl-5-(pyridine-2-yl)pyrimido[4,5-b]quinoline-2,4(1H,3H)-dione ***(3a(pyridin-2-yl))***

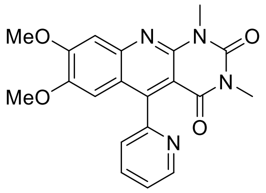

Prepared according to a **general procedure C** from 3,4-dimethoxyaniline (**8g**) (0.15 g, 1 mmol), pyridine-2-carbaldehyde (**14c**) (0.11 g, 1 mmol), and barbituric acid **15** (0.16 g, 1 mmol) in 2 mL of dry DMSO.

Reaction time: 15 hours.
Yield: 69 % (0.26 g) pale purple crystalline powder.

Melting point: 259 – 261 °C (from 2-propanol).
^{1}H NMR (400 MHz, DMSO-d_6) δ 8.04 (ddd, J = 4.9, 1.7, 0.9 Hz, 1H), 6.84 (ddd, J = 7.6, 4.9, 1.1 Hz, 1H), 6.74 (s, 1H), 6.72 (s, 2H), 5.65 (s, 1H), 3.34 (s, 3H), 3.04 (s, 3H), 2.89 (s, 3H), 2.50 (s, 3H).
^{13}C NMR (151 MHz, $CDCl_3$) δ 161.2, 156.8, 155.7, 151.6, 149.7, 149.7, 149.2, 148.0, 147.6, 136.3, 123.6, 122.9, 120.3, 106.9, 106.1, 104.24, 56.5, 55.8, 30.1, 28.5.
HR-MS (APCI+) *m/z:* calculated for $C_{20}H_{18}N_4O_4$ [M+H$^+$]: 379.1406, **observed**: 379.1409.

7,8-Dimethoxy-1,3-dimethyl-5-(thiophene-3-yl)pyrimido[4,5-b]quinoline-2,4(1H,3H)-dione ***(3a(thiophen-3-yl))***

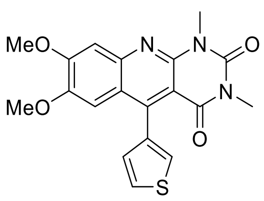

Prepared according to a **general procedure C** from 3,4-dimethoxyaniline (**8g**) (0.08 g, 0.5 mmol), thiophene-3-carbaldehyde (**14e**) (0.06 g, 0.5 mmol), and barbituric acid **15** (0.08 g, 0.5 mmol) in 1 mL of dry DMSO.

Reaction time: 15 hours.
Yield: 37 % (0.14 g) beige crystalline powder.

Melting point: 298 – 301 °C (from 2-propanol).
^{1}H NMR (400 MHz, DMSO-d_6) δ 7.70 (dd, J = 4.9, 2.9 Hz, 1H), 7.47 (dd, J = 3.0, 1.3 Hz, 1H), 7.35 (s, 1H), 7.07 (dd, J = 4.9, 1.3 Hz, 1H), 6.69 (s, 1H), 3.99 (s, 3H), 3.70 (s, 3H), 3.63 (s, 3H), 3.20 (s, 3H).
^{13}C NMR (151 MHz, $CDCl_3$) δ 160.9, 155.6, 151.7, 149.3, 148.2, 147.8, 146.9, 136.8, 128.6, 125.8, 122.6, 121.5, 106.7, 106.6, 104.9, 56.6, 55.9, 30.1, 28.6.
HR-MS (APCI+) *m/z:* calculated for $C_{19}H_{17}N_3O_4S$ [M+H$^+$]: 384.1018, **observed**: 384.1019.

7-Methoxy-1,3-dimethyl-5-(naphtalene-1-yl)pyrimido[4,5-b]quinoline-2,4(1H,3H)-dione ***(3a(napht-1-yl))***

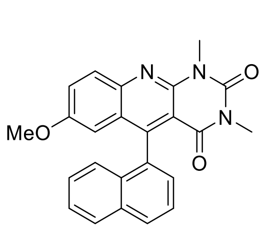

Prepared according to a **general procedure C** from 4-methoxyaniline (**8h**) (0.12 g, 1 mmol), 1-naphtaldehyde (**14b**) (0.16 g, 1 mmol), and barbituric acid **15** (0.16 g, 1 mmol) in 2 mL of dry DMSO.

Reaction time: 15 hours.
Yield: 53 % (0.20 g) yellow crystalline powder.

Melting point: 263 – 265 °C (from 2-propanol).
^{1}H NMR (400 MHz, DMSO-d_6) δ 8.05 (m, 2H), 8.00 (d, *J* = 9.2 Hz, 1H), 7.65 (dd, *J* = 8.3, 7.0 Hz, 1H), 7.58 – 7.55 (m, 1H), 7.52 – 7.46 (m, 1H), 7.34 – 7.24 (m, 2H), 7.11 (dt, *J* = 8.4, 1.0 Hz, 1H), 6.24 (d, *J* = 2.8 Hz, 1H), 3.75 (s, 3H), 3.38 (s, 3H), 3.09 (s, 3H).
^{13}C NMR (151 MHz, $CDCl_3$) δ 160.5, 157.2, 151.8, 151.7, 147.60, 145.4, 135.3, 133.4, 131.5, 129.7, 128.7, 128.5, 126.9, 126.6, 126.2, 125.9, 125.4, 125.3, 125.0, 109.3, 105.4, 55.4, 30.2, 28.6.
HR-MS (APCI+) *m/z:* calculated for $C_{24}H_{19}N_3O_3$ [M+H$^+$]: 398.1504, **observed**: 398.1497.

7-Methoxy-1,3-dimethyl-5-(pyridine-4-yl)pyrimido[4,5-b]quinoline-2,4(1H,3H)-dione ***(3b(pyridin-4-yl))***

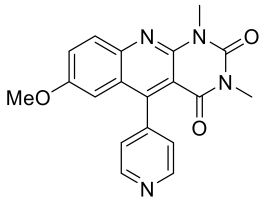

Prepared according to a **general procedure C** from 4-methoxyaniline (**8h**) (0.12 g, 1 mmol), pyridine-4-carbaldehyde (**14c**) (0.11 g, 1 mmol), and barbituric acid **15** (0.16 g, 1 mmol) in 2 mL of dry DMSO.

Reaction time: 15 hours.
Yield: 30 % (0.10 g) yellow crystalline powder.

Melting point: 303 – 305 °C (from 2-propanol).
^{1}H NMR (400 MHz, DMSO-d_6) δ 8.76 – 8.70 (m, 2H), 7.98 (d, *J* = 9.2 Hz, 1H), 7.68 – 7.54 (m, 1H), 7.34 – 7.28 (m, 2H), 6.43 (d, *J* = 2.8 Hz, 1H), 3.71 (s, 3H), 3.62 (s, 3H), 3.18 (s, 3H).
^{13}C NMR (151 MHz, $CDCl_3$) δ 160.9, 157.5, 151.4, 149.9 (2C), 149.3, 147.2, 146.4, 145.5, 129.9, 126.4, 125.4, 122.9 (2C), 107.6, 104.3, 55.6, 30.2, 28.7.
HR-MS (APCI+) *m/z:* calculated for $C_{19}H_{16}N_4O_3$ [M+H$^+$]: 349.1300, **observed**: 349.1293.

7-Methoxy-1,3-dimethyl-5-(pyridine-2-yl)pyrimido[4,5-b]quinoline-2,4(1H,3H)-dione ***(3b(pyridin-2-yl))***

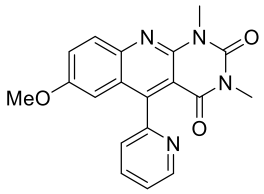

Prepared according to a **general procedure C** from 4-methoxyaniline (**8h**) (0.37 g, 1 mmol), pyridine-2-carbaldehyde (**14d**) (0.11 g, 1 mmol), and barbituric acid **15** (0.16 g, 1 mmol) in 2 mL of dry DMSO.

Reaction time: 15 hours.
Purification: column chromatography in mobile phase $CHCl_3$: EtOAc (1:1).
Yield: 45 % (0.16 g) green-yellow crystalline powder.
Melting point: 255 – 257 °C (from 2-propanol).

^{1}H NMR (400 MHz, DMSO-*d*$_6$) δ 8.71 (ddd, *J* = 5.0, 1.7, 0.9 Hz, 1H), 8.00 – 7.91 (m, 2H), 7.59 (dd, *J* = 9.2, 2.8 Hz, 1H), 7.52 (ddd, *J* = 7.6, 5.0, 1.1 Hz, 1H), 7.44 – 7.37 (m, 1H), 6.37 (d, *J* = 2.8 Hz, 1H), 3.71 (s, 3H), 3.60 (s, 3H), 3.17 (s, 3H).
^{13}C NMR (151 MHz, $CDCl_3$) δ 161.4, 157.6, 156.9, 151.9, 150.6, 150.1, 147.6, 146.1, 136.7, 130.1, 126.4, 126.1, 123.9, 123.3, 108.5, 104.9, 55.8, 30.5, 28.9.
HR-MS (APCI+) *m/z:* calculated for $C_{19}H_{16}N_4O_3$ [M+H$^+$]: 349.1300, **observed**: 349.1296.

7-Methoxy-1,3-dimethyl-5-(thiophene-3-yl)pyrimido[4,5-b]quinoline-2,4(1H,3H)-dione ***(3b(thiophen-3-yl))***

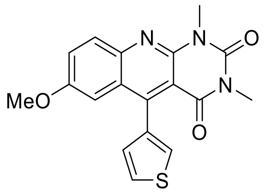

Prepared according to a **general procedure C** from 4-methoxyaniline (**8h**) (0.06 g, 0.5 mmol), thiophene-3-carbaldehyde (**14e**) (0.06 g, 0.5 mmol), and barbituric acid **15** (0.08 g, 0.5 mmol) in 1 mL of dry DMSO.

Reaction time: 15 hours.
Purification: column chromatography in mobile phase $CHCl_3$: EtOAc (50:1).
Yield: 17 % (0.06 g) yellow crystalline powder.

Melting point: 268 – 270 °C (from 2-propanol).
^{1}H NMR (400 MHz, DMSO-*d*$_6$) δ 7.91 (d, *J* = 9.2 Hz, 1H), 7.71 (ddd, *J* = 4.9, 2.9, 0.5 Hz, 1H), 7.55 (ddd, *J* = 9.1, 2.9, 0.5 Hz, 1H), 7.46 (ddd, *J* = 3.0, 1.3, 0.5 Hz, 1H), 7.06 (ddd, *J* = 4.9, 1.3, 0.5 Hz, 1H), 6.72 (d, *J* = 2.8 Hz, 1H), 3.68 (s, 3H), 3.66 (s, 3H), 3.19 (s, 3H).
^{13}C NMR (151 MHz, $CDCl_3$) δ 161.2, 157.5, 151.9, 149.1, 147.7, 145.5, 136.8, 129.9, 128.9, 127.3, 126.2, 126.1, 123.0, 109.0, 105.4, 55.8, 30.5, 29.0.
HR-MS (APCI+) *m/z:* calculated for $C_{18}H_{15}N_3O_3S$ [M+H$^+$]: 354.0912, **observed**: 354.0903.

2.3 Synthesis of 5-trifluormethyldeazaalloxazines

General procedure B1 (Ref.[7])

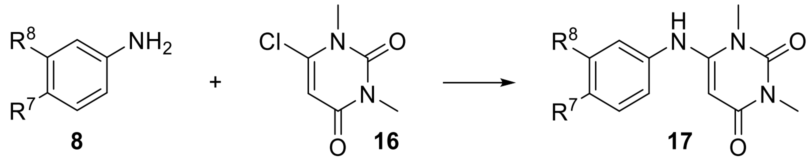

A mixture of anilines **8** (1 equiv.) and 6-chloro-1,3-dimethyluracil (**16**) (1 equiv.) was placed in a heated oil bath (180 °C) and then heated for 3 hours at 180 °C. After cooling of the reaction mixture, water was added. The product was precipitated by ultrasound and then vacuum filtered.

General procedure B2 (Ref.[7])

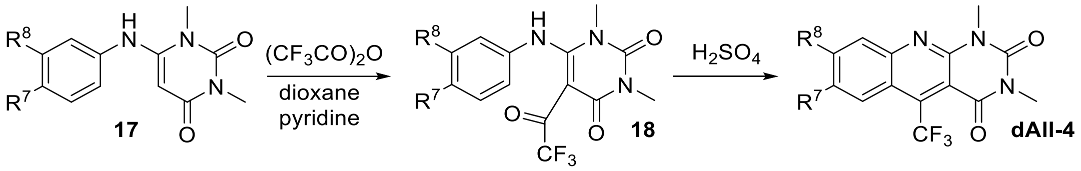

A mixture of anilinouracil **17** (1 equiv.), trifluoroacetic anhydride (2 equiv.) and pyridine (1.2 equiv.) was dissolved in dioxane (2 mL) and then stirred overnight at room temperature. Then, the solvent was evaporated on a vacuum evaporator. The product was precipitated from the water by ultrasonication and then vacuum filtered.

The filtered product **18** was dissolved in H_2SO_4 (2 ml). The mixture was stirred for 4 hours at room temperature and then poured into cold water (20 ml). The **dAll-4** product was vacuum filtered.

*6-[(3,4-Dimethoxyphenyl)amino]-1,3-dimethylpyrimidine-2,4(1H,3H)-dione (**17a**)*

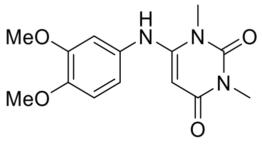

Prepared according to a **general procedure B1** from 3,4-dimethoxyaniline (**8g**) (0.30 g, 2.0 mmol) and uracil **16** (0.34 g, 2.0 mmol).

Yield: 57 % (0.32 g) grey solid.

^{1}H NMR (400 MHz, DMSO-d_6) δ 8.42 (s, 1H), 7.00 (d, *J* = 8.5 Hz, 1H), 6.85 (d, *J* = 2.4 Hz, 1H), 6.83 – 6.74 (d, 1H), 4.48 (s, 1H), 3.77 (s; 3H), 3.74 (s, 3H), 3.42 (s, 3H), 3.10 (s, 3H).

7,8-Dimethoxy-1,3-dimethyl-5-(trifluoromethyl)pyrimido[4,5-b]quinoline-2,4(1H,3H)-dione ***(4a)***

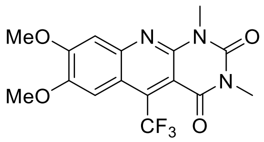

Prepared according to a **general procedure B2** from anilinouracil **17a** (0.22 g, 0.7 mmol), trifluoroacetic anhydride (210 µL, 1.4 mmol) and pyridine (73 µL, 0.9 mmol).

Separation: column chromatography in mobile phase $CHCl_3$: EtOAc (10:1).

Yield: 18 % (0.02 g) yellow crystalline powder.

Melting point: 292 – 295 °C (from 2-propanol).
^{1}H NMR (400 MHz, DMSO-d_6) δ 7.43 (s, 1H), 7.41 (s, 1H), 4.03 (s, 3H), 3.95 (s, 3H), 3.66 (s, 3H), 3.32 (s, 3H).
^{13}C NMR (151 MHz, DMSO-d_6) δ 163.3, 160.1, 154.0, 151.7, 147.4, 145.8, 141.5, 122.5 (q, J_{C-F} = 278 Hz), 120.8, 110.1, 106.9 (q, J_{C-F} = 6.9 Hz), 101.6, 59.0, 57.8, 33.0, 31.5.
^{19}F NMR (376 MHz, DMSO-d_6) δ -51.62.
HR-MS (APCI+) *m/z:* calculated for $C_{16}H_{14}F_3N_3O_4$ [M+H$^+$]: 370.1014, **observed**: 370.1010.

*6-[(4-Methoxyphenyl)amino]-1,3-dimethyldihydropyrimidine-2,4(1H,3H)-dione (**17b**)*

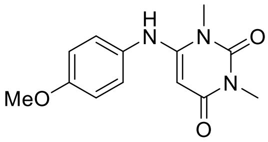

Prepared according to a **general procedure B1** from 4-methoxyaniline (**8h**) (0.30 g, 2.4 mmol) and uracil **16** (0.42 g, 2.4 mmol).

Yield: 71 % (0.45 g) brown solid.

^{1}H NMR (400 MHz, DMSO-d_6) δ 8.41 (s, 1H), 7.21 – 7.13 (d, 2H), 7.04 – 6.96 (d, 2H), 4.40 (s, 1H), 3.77 (s, 3H), 3.43 (s, 3H), 3.09 (s, 3H).

*7-Methoxy-1,3-dimethyl-5-(trifluoromethyl)pyrimido[4,5-b]quinoline-2,4(1H,3H)-dione (**4b**)*

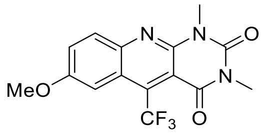

Prepared according to a **general procedure B2** from anilinouracil **17b** (0.45 g, 1.7 mmol), trifluoroacetic anhydride (480 µL, 3.4 mmol) and pyridine (166 µL, 2.1 mmol).

Yield: 19 % (0.12 g) green-yellow crystalline powder.

Melting point: 179 – 182 °C (from 2-propanol).
^{1}**H NMR** (400 MHz, DMSO-*d6*) δ 8.00 (d, *J* = 9.3 Hz, 1H), 7.69 (dd, *J* = 9.3, 2.7 Hz, 1H), 7.47 (t, *J* = 2.3 Hz, 1H), 3.94 (s, 3H), 3.66 (s, 3H), 3.33 (s, 3H).
^{13}**C NMR** (101 MHz, CDCl-*d*3) δ 159.4, 158.0, 151.4, 146.8, 146.6, 136.2, 135.9, 130.3, 127.1, 123.7 (q, $J_{C\text{-}F}$ = 278 Hz), 122.9, 102.9 (q, $J_{C\text{-}F}$ = 6.5 Hz), 55.8, 30.4, 29.3.
^{19}**F NMR** (376 MHz, $CDCl_3$) δ -51.74.
HR-MS (APCI+) *m/z:* calculated for $C_{15}H_{12}F_3N_3O_3$ [M+H$^+$]: 340.0909, **observed**: 340.0906.

*3-Methyl-6-(phenylamino)pyrimidine-2,4(1H,3H)-dione (**17e**)*

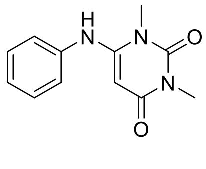

Prepared according to a **general procedure B1** from aniline (**8a**) (0.70 g, 7.5 mmol) and uracil **16** (1.20 g, 7.5 mmol).

Yield: 72 % (0.71 g) pale yellow solid.

^{1}**H NMR** (400 MHz, DMSO-*d6)* δ 8.53 (s, 1H), 7.48 – 7.39 (m, 2H), 7.29 – 7.20 (m, 3H), 4.63 (s, 1H), 3.43 (s, 3H), 3.11 (s, 3H).

2.4 Synthesis of substrates

N-(4-Fluorophenyl)acetamide

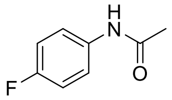

A solution of the 4-fluoroaniline (1.00 g, 9.00 mmol) and DIPEA (3.14 ml, 18.0 mmol) in CH_2Cl_2 (20.0 ml) was cooled to 0 °C and then acetic acid anhydride (1.00 ml, 10.8 mmol) was added dropwise. Reaction mixture was stirred at room temperature and after reaction completion (monitored by TLC, hexane:EtOAc, 1:1) was poured to water and extracted with CH_2Cl_2 (three times). Organic phase was washed with brine, dried over $MgSO_4$, and concentrated in vacuo, giving the product.

Yield: 73 % (1.00 g) beige solid

Melting point: 148–150 °C (hexane)

^{1}**H NMR** (400 MHz, DMSO-*D*6) δ 9.95 (s, 1H, NH), 7.61 – 7.53 (m, 2H), 7.07 (t, *J* = 9.0 Hz, 2H), 1.99 (s, 3H, CH_3).

^{13}**C NMR** (101 MHz, DMSO-*d6*): δ 168.7, 158.3 (d, $J_{C\text{-}F}$= 239.3 Hz), 136.3 (d, $J_{C\text{-}F}$ = 2.5 Hz), 121.2 (d, $J_{C\text{-}F}$ = 7.8 Hz), 115.7 (d, $J_{C\text{-}F}$ = 22.1 Hz), 24.4.

^{19}**F NMR** (376 MHz, DMSO-*d*6) δ -119.7.

S3.SPECTROELECTROCHEMICAL MEASUREMENTS

Figure S1: Sequence of UV-Vis spectra of spectroelectrochemical measurements of selected deazaalloxazines performed in an OTTLE cell after exposure to -1.4 V.

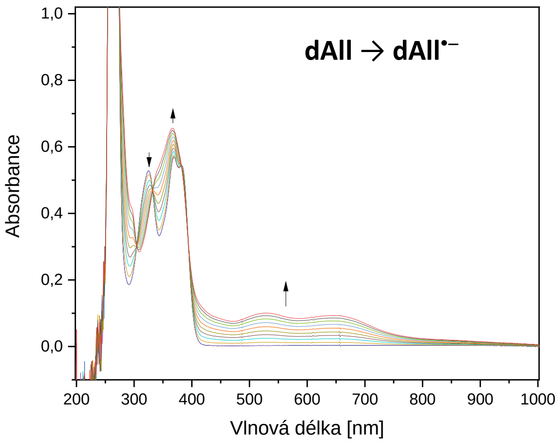

Sequence of UV-Vis absorption spectra of the forming radical anion **3a(Ph)**

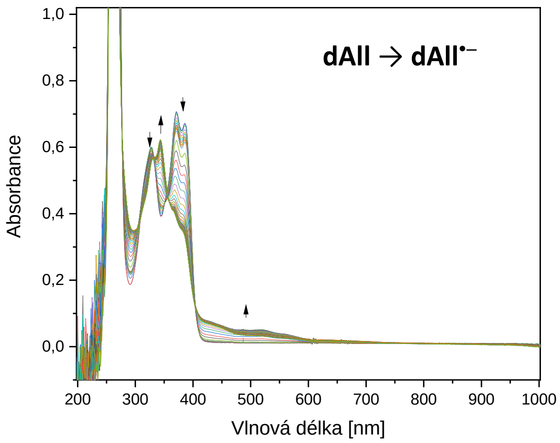

Sequence of UV-Vis absorption spectra of the forming radical anion **3a(*o*-BrPh)**

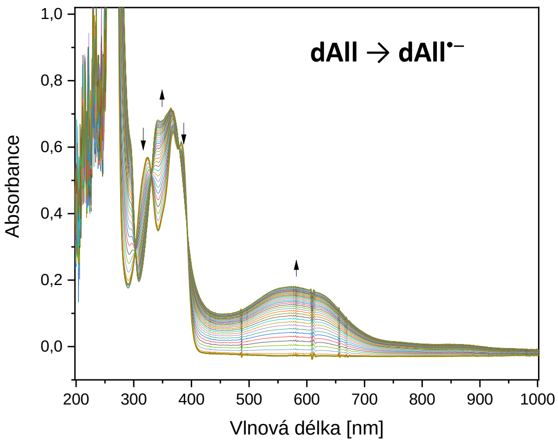

Sequence of UV-Vis absorption spectra of the forming radical anion **3a(*o*-MePh)**

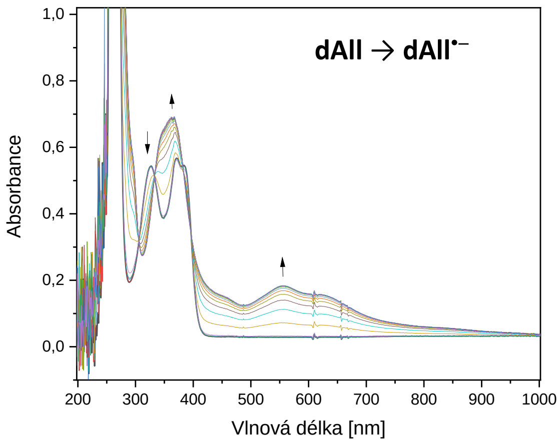

Sequence of UV-Vis absorption spectra of the forming radical anion **3a(thiophen-3-yl)**

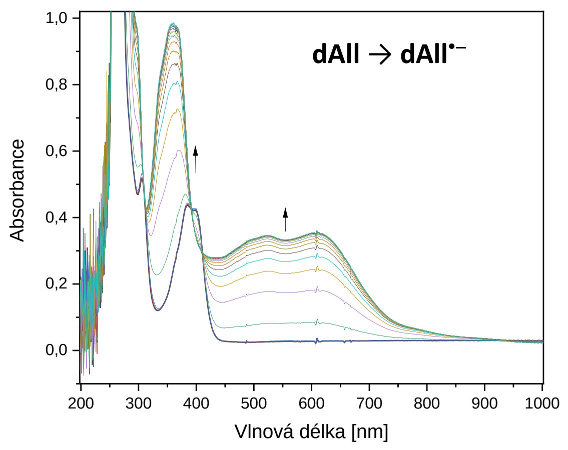

Sequence of UV-Vis absorption spectra of the forming radical anion **3b(Ph)**

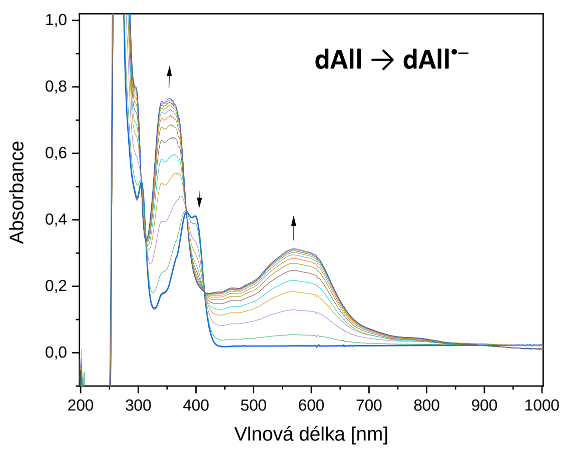

Sequence of UV-Vis absorption spectra of the forming radical anion **3b(*o*-MePh)**

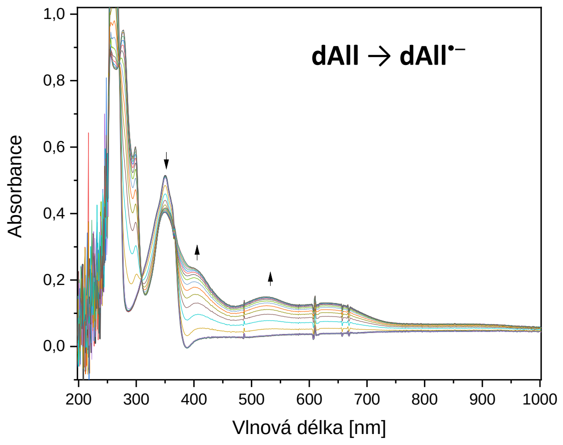

Sequence of UV-Vis absorption spectra of the forming radical anion **3c(Ph)**

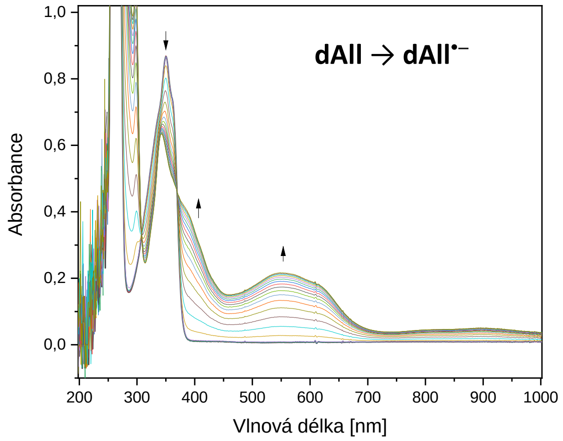

Sequence of UV-Vis absorption spectra of the forming radical anion **3c(*o*-MePh)**

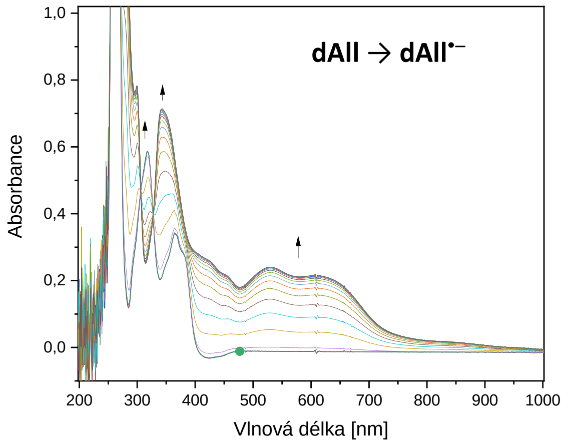

Sequence of UV-Vis absorption spectra of the forming radical anion **3d(Ph)**

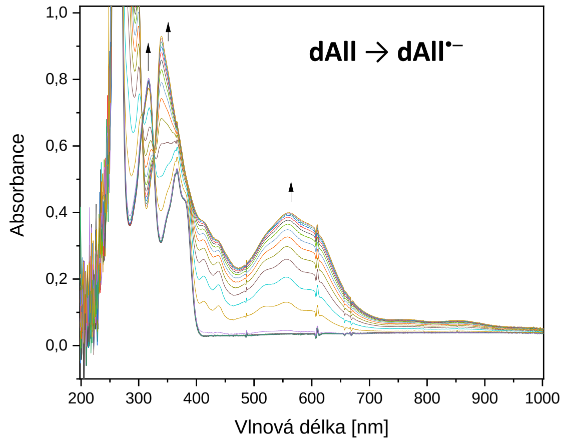

Sequence of UV-Vis absorption spectra of the forming radical anion **3d(*o*-MePh)**

S4. CYCLIC VOLTAMMOGRAMS OF DEAZAALLOXAZINES

Figure S2: Cyclic voltammetry of selected **dAll** measured in DMF + 0,1 M Me_4NPF_6 on a glassy carbon electrode versus SCE at a rate of 100 mV·s^{-1}.

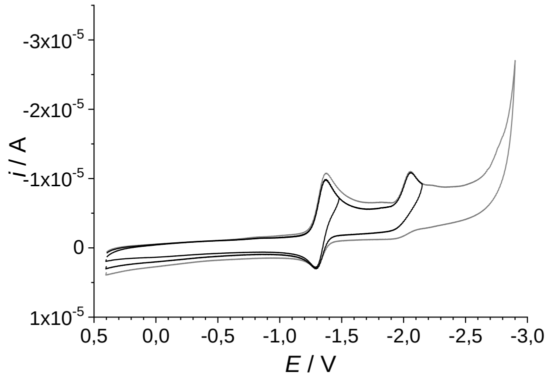

Cyclic voltammetry of **1(*o*-MePh)**

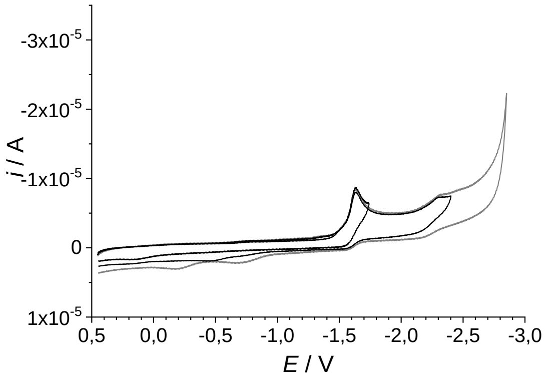

Cyclic voltammetry of **2a**

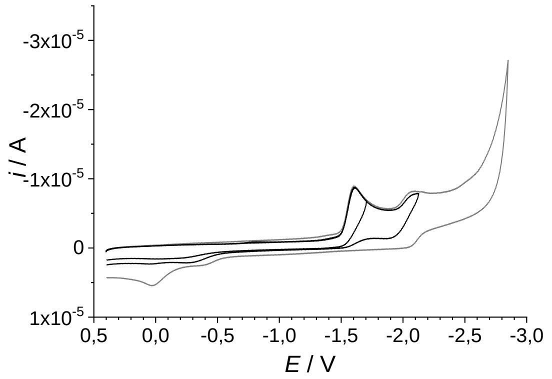

Cyclic voltammetry of **2a-H**

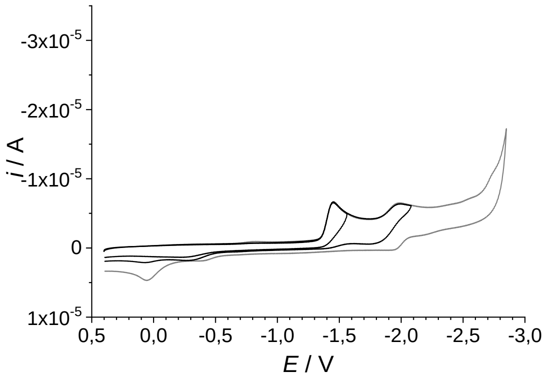

Cyclic voltammetry of **2b-H**

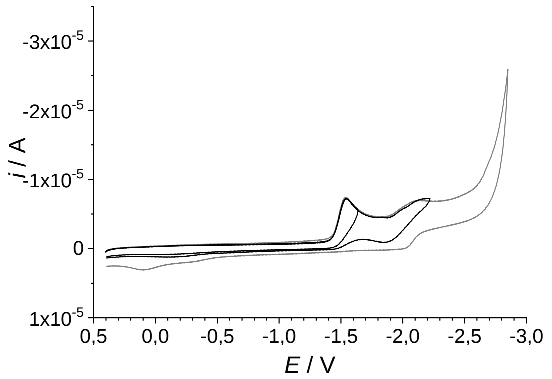

Cyclic voltammetry of **2c-H**

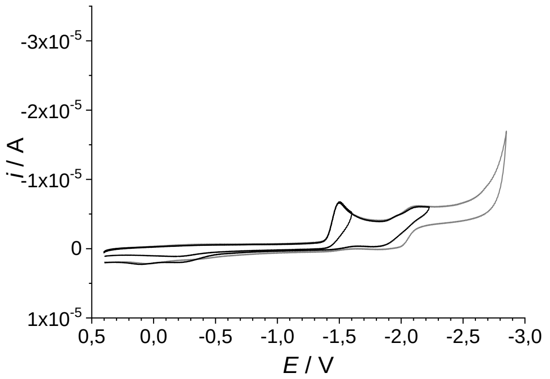

Cyclic voltammetry of **2d-H**

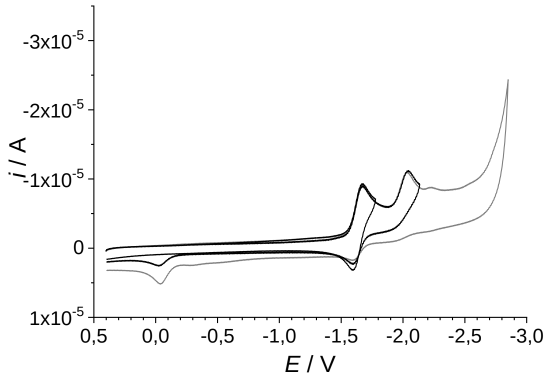

Cyclic voltammetry of **3a(Ph)**

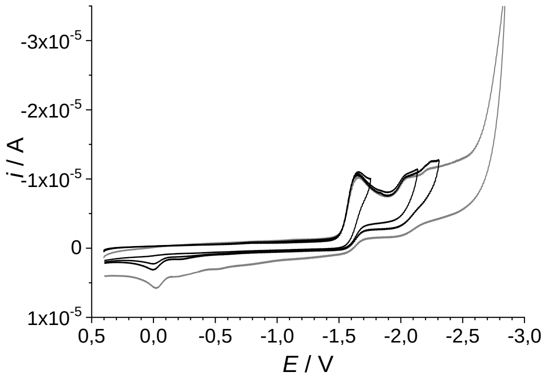

Cyclic voltammetry of **3a(*o*-BrPh)**

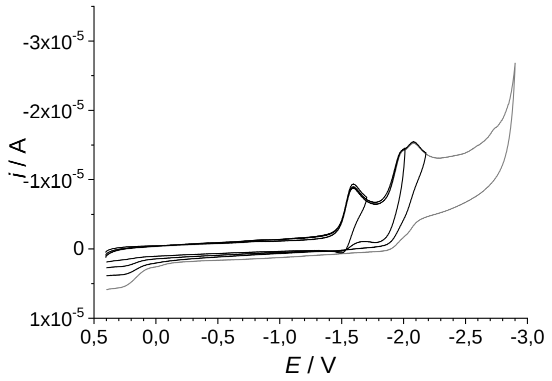

Cyclic voltammetry of **3a(*o*-BrPh)-H**

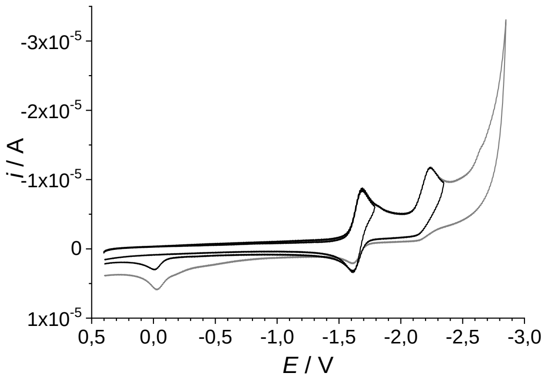

Cyclic voltammetry of **3a(*o*-MePh)**

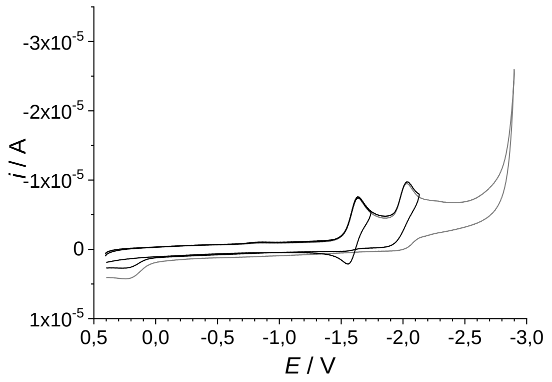

Cyclic voltammetry of **3a(*o*-MePh)-H**

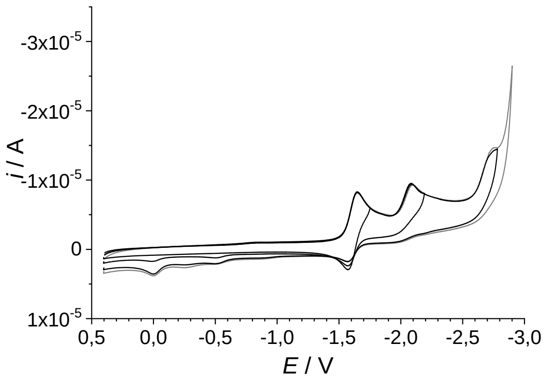

Cyclic voltammetry of **3a(thiophen-3-yl)**

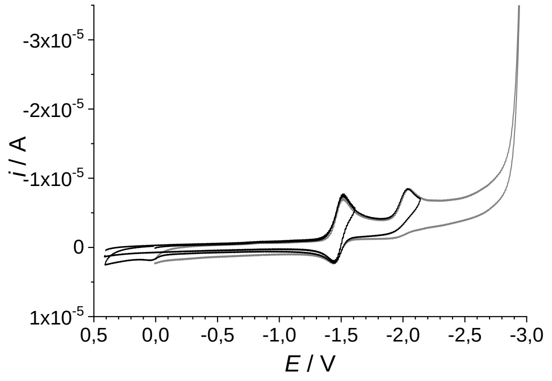

Cyclic voltammetry of **3b(Ph)**

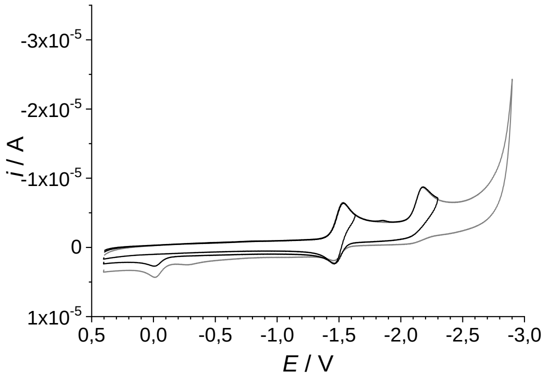

Cyclic voltammetry of **3b(*o*-MePh)**

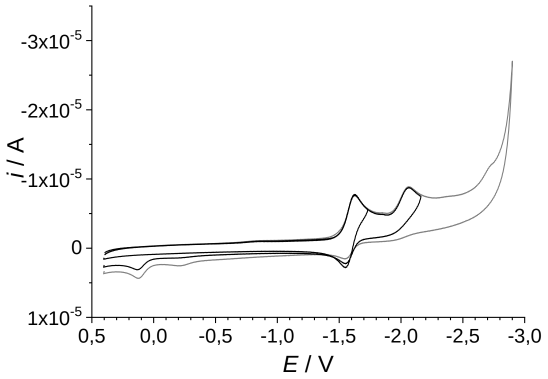

Cyclic voltammetry of **3c(Ph)**

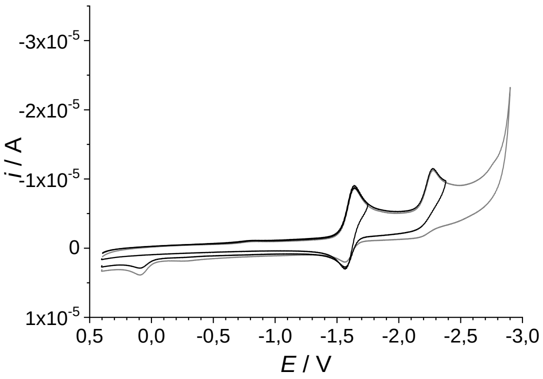

Cyclic voltammetry of **3c(*o*-MePh)**

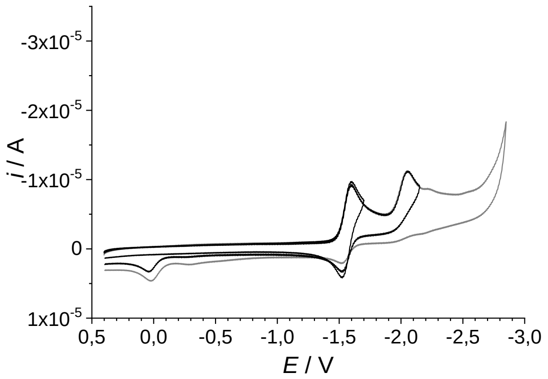

Cyclic voltammetry of **3d(Ph)**

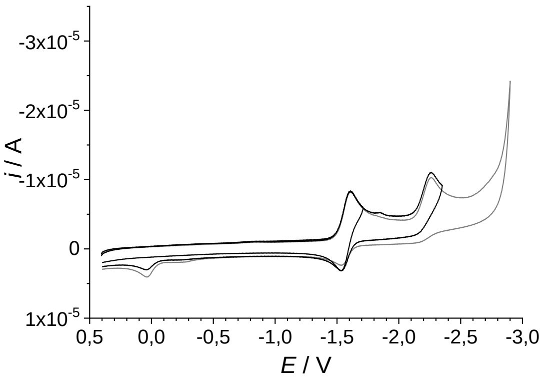

Cyclic voltammetry of **3d(*o*-MePh)**

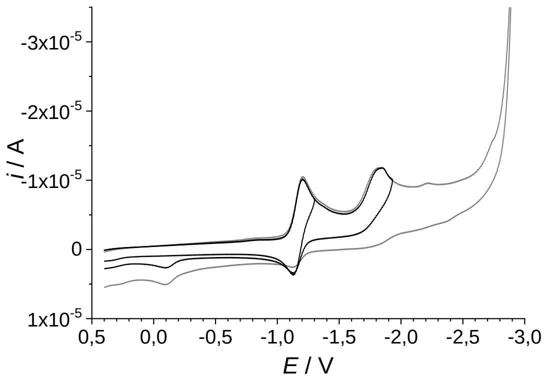

Cyclic voltammetry of **4a**

S5. PHOTOPHYSICAL PROPERTIES

5.1 Experimental data

Table S2: Biexponential fluorescence kinetics with percentage amplitude in *N*, *N*-dimethylformamide.

dAll	$\lambda_{abs.}$ (nm)/ log ε	λ_F (nm)	Φ_F	τ_F (ns) 2nd Exp decay/ Amplitude (%)	*Amplitue average lifetime*	k_r ($\times 10^8$ s^{-1})	Σk_{nr} ($\times 10^8$ s^{-1})
**2b-H**	380/ 3.97	441	0.48	13.69/59.40 5.92/40.60	10.54	0.46	0.49
**3a(*o*-MePh)**	367/ 3.97	423	0.15	1.43/62.87 1.85/37.13	1.59	0.94	5.35
**3a(napht-1-yl)**	370/ 3.98	428	0.014	0.10/96.41 1.10/2.47	0.13	1.08	75.9
**3a(pyridin-4-yl)**	372/ 3.95	449	0.23	2.53/95.47 0.74/4.53	2.45	0.94	3.14
**3a(pyridin-2-yl)**	371/ 4.05	472	0.17	2.41/95.83 0.74/4.17	2.34	0.73	3.55
**3a(thiophen-3-yl)**	372/ 3.91	433	0.020	0.30/93.24 1.93/6.76	0.41	0.49	23.9
**3b(Ph)**	385/ 3.33	459	0.47	13.0/72.61 4.36/27.39	10.6	0.44	0.50
**3b(*o*-BrPh)**	387/ 4.05	455	0.13	7.77/65.23 1.38/34.77	5.55	0.23	1.57
**3b(*o*-MePh)**	385/ 3.60	451	0.43	12.53/74.43 3.70/25.57	10.27	0.42	0.56
**3b(napht-1-yl)**	386/ 3.60	456	0.049	1.77/84.70 0.28/15.30	1.54	0.32	6.18
**3b(pyridin-4-yl)**	387/ 3.76	471	0.55	15.54/69.98 6.78/30.02	12.91	0.43	0.35
**3b(pyridin-2-yl)**	386/ 3.77	490	0.46	14.70/65.08 6.32/34.92	11.77	0.39	0.46
**3b(thiophen-3-yl)**	389/ 3.85	463	0.020	0.27/67.66 0.95/32.34	0.49	0.41	20.0
**3c(Ph)**	350/ 4.10	405	0.014	0.21/90.55 2.76/9.45	0.45	0.31	21.9
**3c(*o*-BrPh)**	353/ 4.19	402	0.017	0.24/92.32 0.70/7.68	0.28	0.61	35.1
**3c(*o*-MePh)**	350/ 4.26	395	0.009	0.12/84.11 2.74/15.89	0.54	0.17	18.4
**3d(*o*-BrPh)**	367/ 3.88	425	0.025	0.41/97.36 2.00/2.64	0.45	0.56	21.7
**3d(*o*-MePh)**	363/ 3.67	422	0.043	0.77/90.88 16.89/9.12	2.24	0.19	4.27
**4b**	417/ 3.90	514	0.50	18.73/66.72 7.43/33.28	14.97	0.33	0.33

A single representative value is reported by calculating the *amplitude-weighted average* lifetime. $\tau_{avg} =(A_1\tau_1+A_2\tau_2)/A_1+A_2)$, which is used to determine the radiative and nonradiative rate constants.

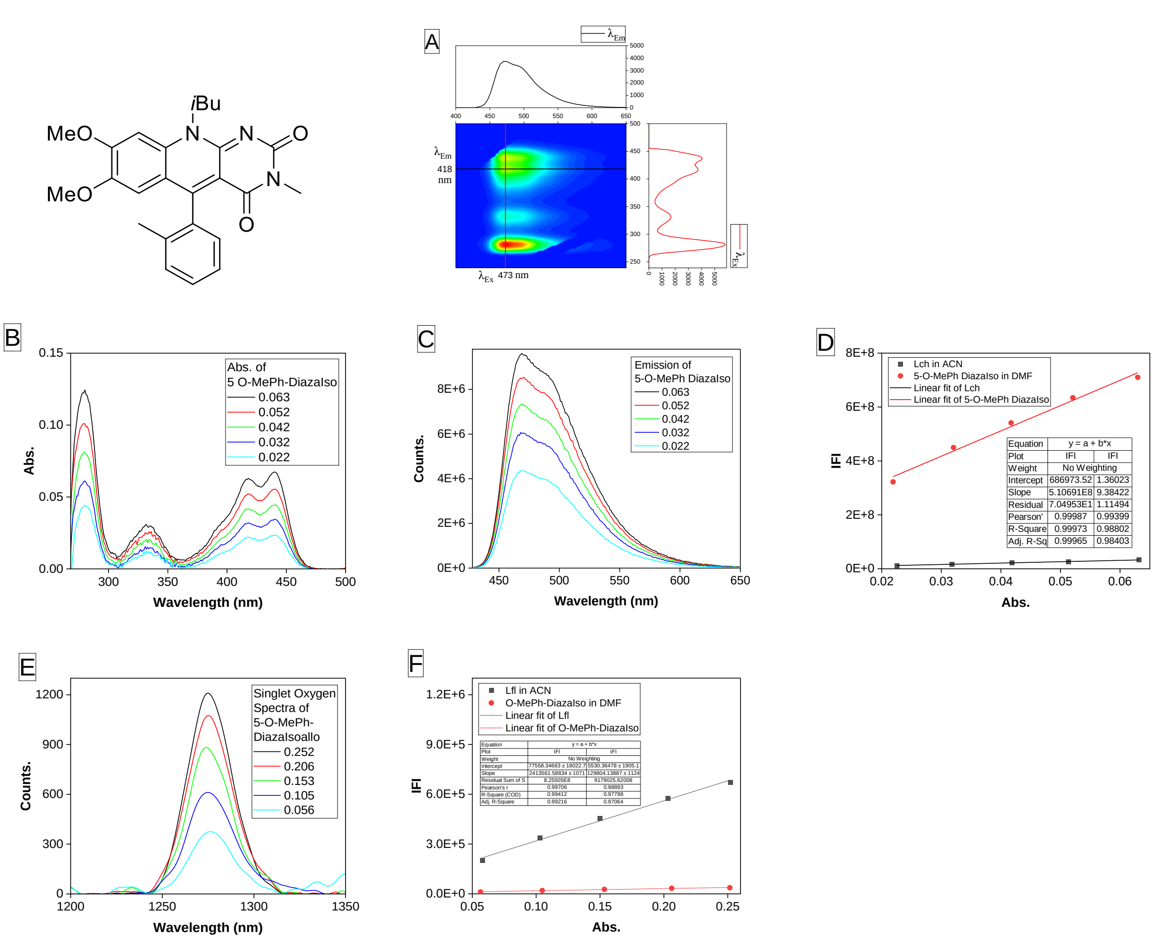

Figure S3: Photophysical data of a compound **1(*o*-MePh)**. **Panel-[A]** represents a fully corrected emission excitation matrix in DMF. **[B]** Absorption spectra of **1(*o*-MePh)** with 5 different concentrations and their **[C]** relative fluorescence spectra recorded using λ_{ex} = 418 nm. **[D]** Integrated fluorescence intensity versus absorption plot. **[E]** Phosphorescence spectra of singlet oxygen, samples excited at λ_{ex} = 443 nm and spectra collected in the NIR range between 1200 – 1350 nm. **[F]** Integrated phosphorescence intensity versus absorption plot.

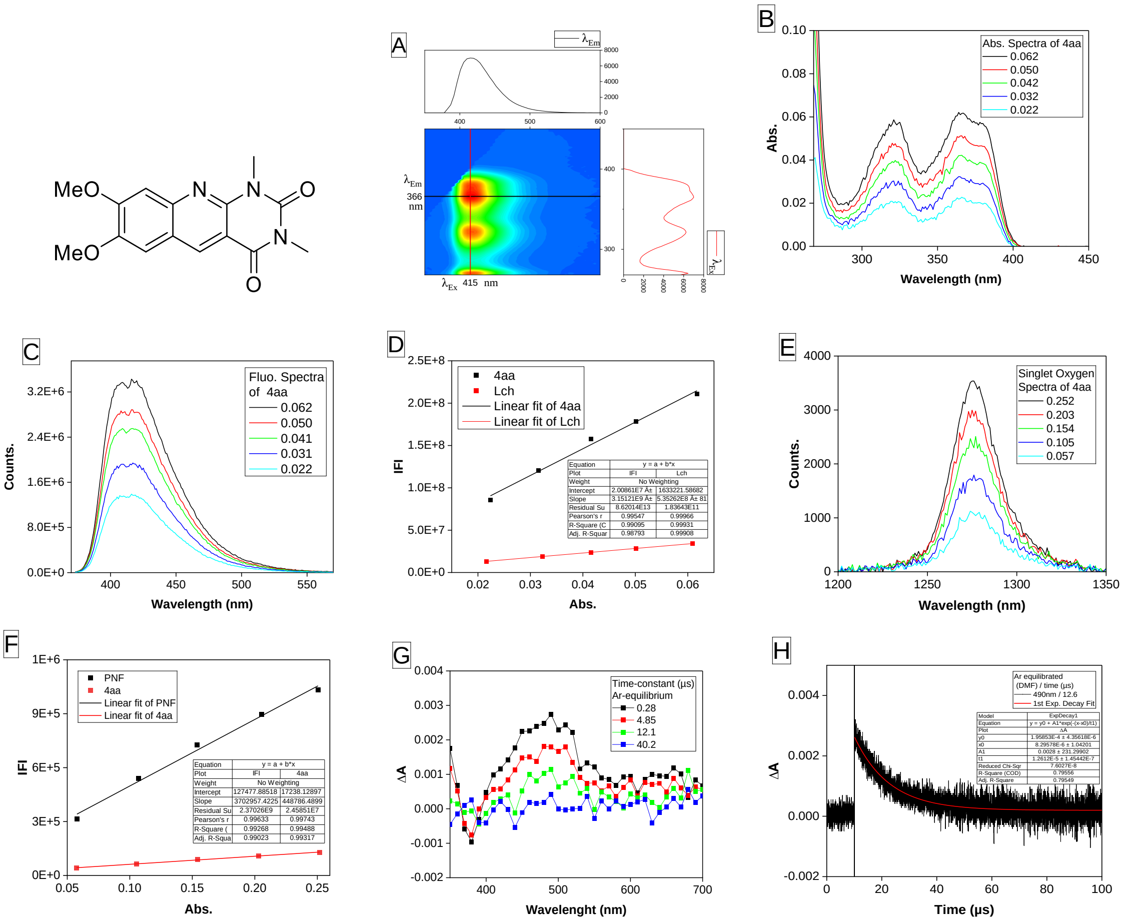

Figure S4: Photophysical data of a compound **2a**. **Panel-[A]** represents a fully corrected emission excitation matrix in DMF. **[B]** Absorption spectra of **2a** with 5 different concentrations and their **[C]** relative fluorescence spectra recorded using λ_{ex} = 367 nm. **[D]** Integrated fluorescence intensity versus absorption plot. **[E]** Phosphorescence spectra of singlet oxygen, samples excited at λ_{ex} = 365 nm and spectra collected in the NIR range between 1200 – 1350 nm. **[F]** Integrated phosphorescence intensity versus absorption plot. **[G]** Transient absorption spectra of **2a** using a laser pulse at λ_{exc} = 366 nm and energy 0.5 mJ, collected between 350 -700 nm using a 10 nm interval, (Ar-equilibrated solution). **[H]** Transient absorption kinetics (Black) and decay fit in red (Ar-equilibrated solution).

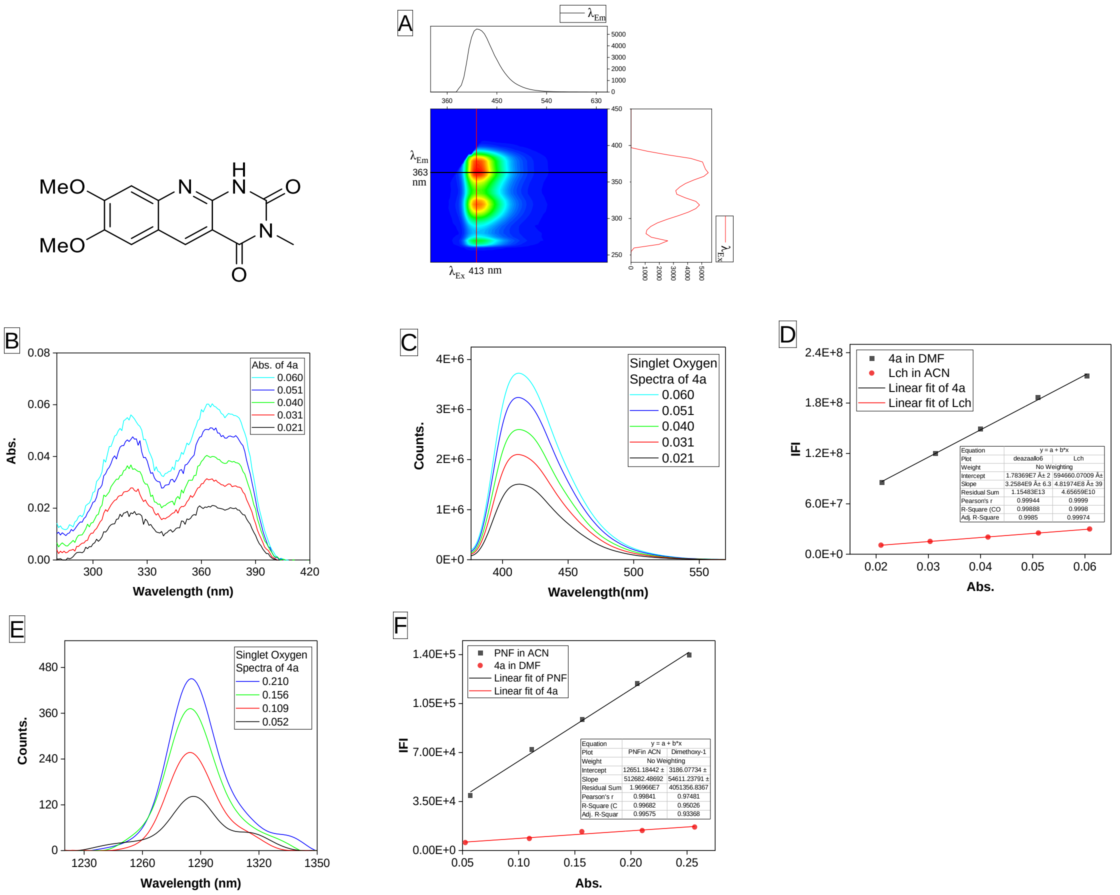

Figure S5: Photophysical data of a compound **2a-H**. **Panel-[A]** represents a fully corrected emission excitation matrix in DMF. **[B]** Absorption spectra of **2a-H** with 5 different concentrations and their **[C]** relative fluorescence spectra recorded using λ_{ex} = 359 nm. **[D]** Integrated fluorescence intensity versus absorption plot. **[E]** Phosphorescence spectra of singlet oxygen, samples excited at λ_{ex} = 375 nm and spectra collected in the NIR range between 1200 – 1350 nm. **[F]** Integrated phosphorescence intensity versus absorption plot.

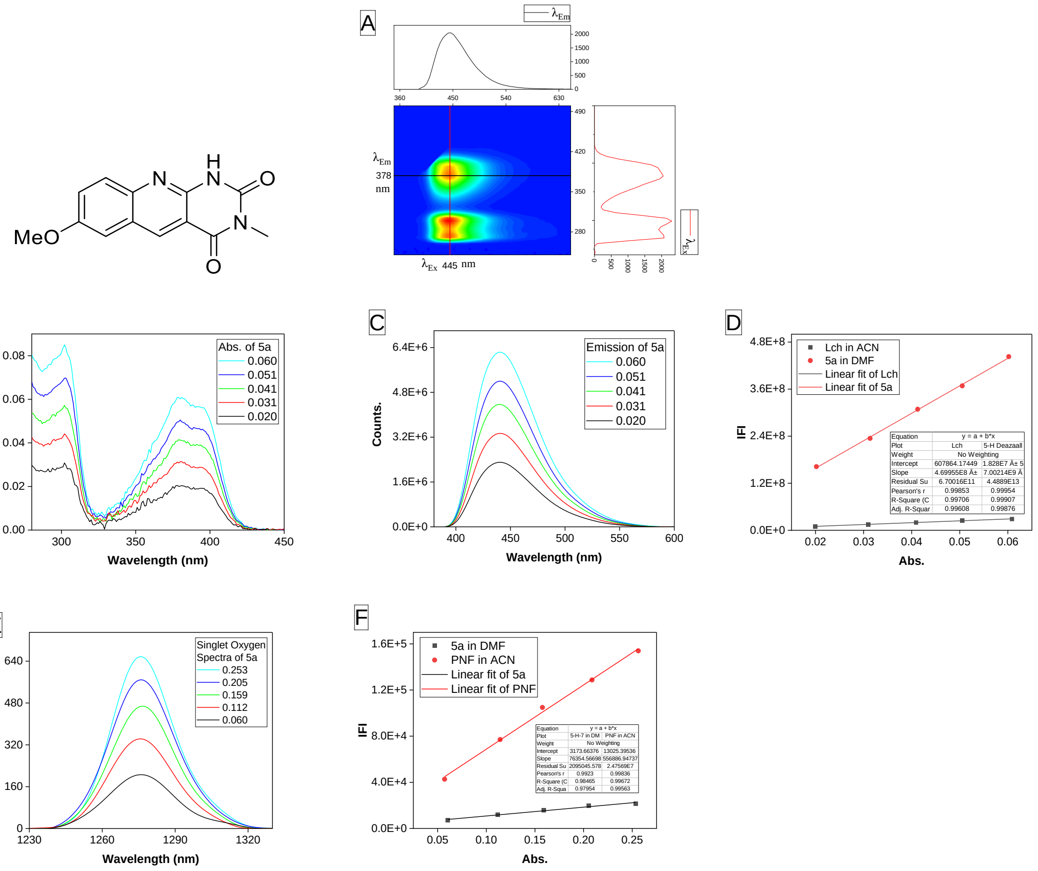

Figure S6: Photophysical data of a compound **2b-H**. **Panel-[A]** represents a fully corrected emission excitation matrix in DMF. **[B]** Absorption spectra of **2b-H** with 5 different concentrations and their **[C]** relative fluorescence spectra recorded using λ_{ex} = 380 nm. **[D]** Integrated fluorescence intensity versus absorption plot. **[E]** Phosphorescence spectra of singlet oxygen, samples excited at λ_{ex} = 375 nm and spectra collected in the NIR range between 1200 – 1350 nm. **[F]** Integrated phosphorescence intensity versus absorption plot.

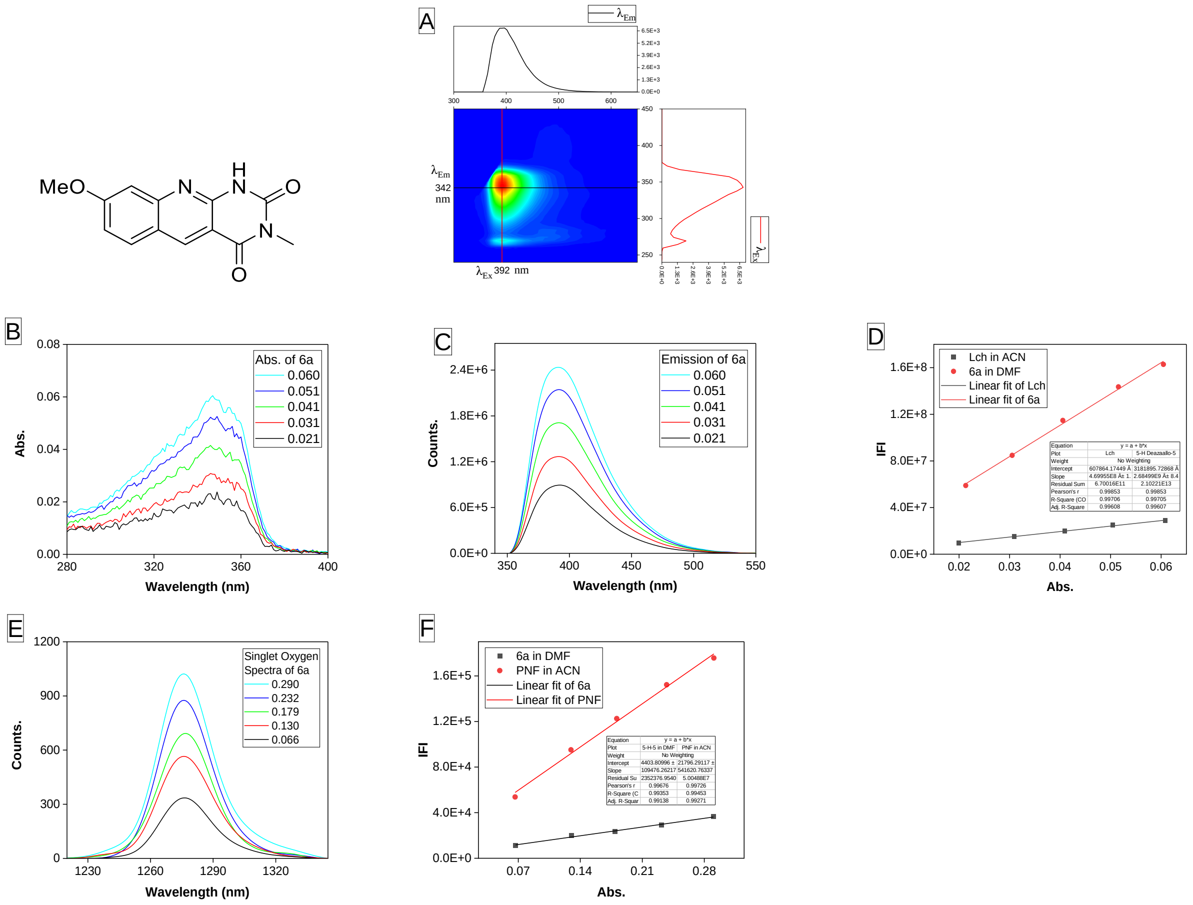

Figure S7: Photophysical data of a compound **2c-H**. **Panel-[A]** represents a fully corrected emission excitation matrix in DMF. **[B]** Absorption spectra of **2c-H** with 5 different concentrations and their **[C]** relative fluorescence spectra recorded using λ_{ex} = 347 nm. **[D]** Integrated fluorescence intensity versus absorption plot. **[E]** Phosphorescence spectra of singlet oxygen, samples excited at λ_{ex} = 357 nm and spectra collected in the NIR range between 1200 – 1350 nm. **[F]** Integrated phosphorescence intensity versus absorption plot.

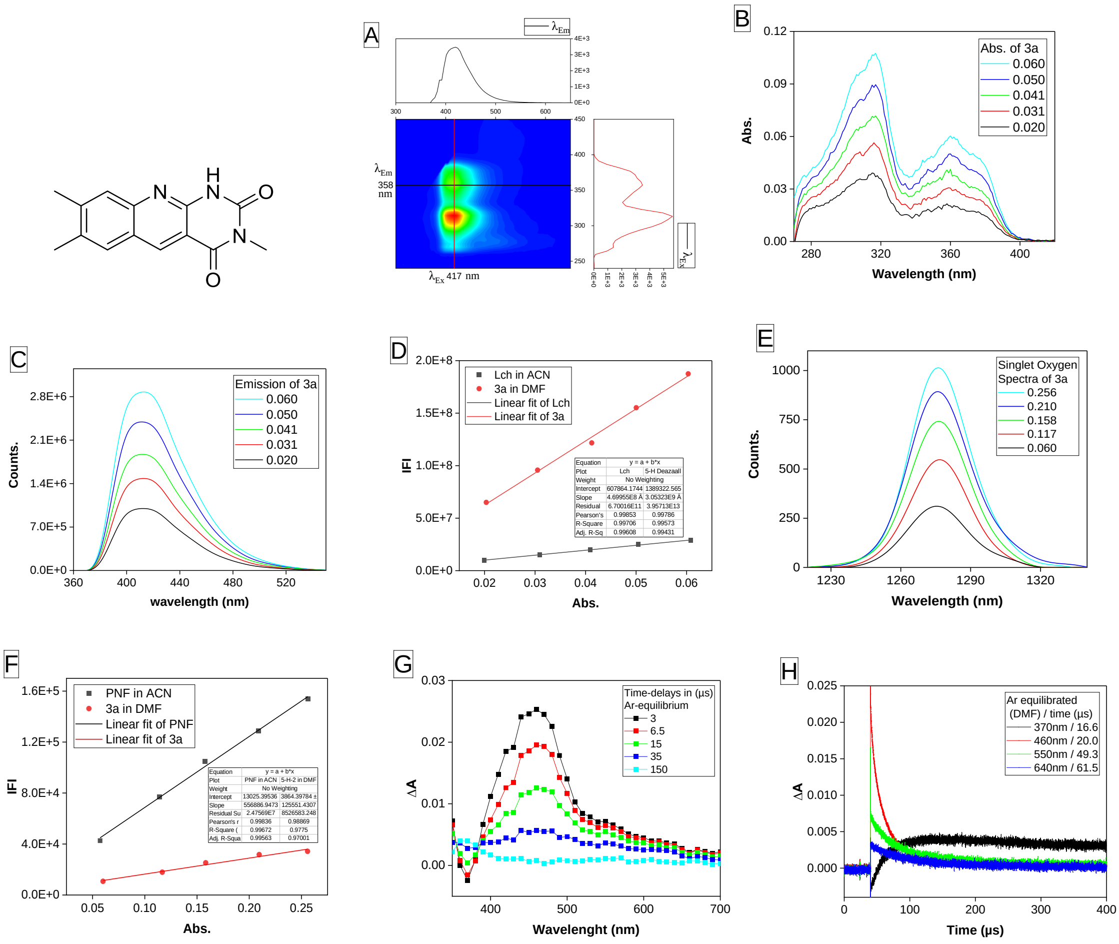

Figure S8: Photophysical data of a compound **2d-H**. **Panel-[A]** represents a fully corrected emission excitation matrix in DMF. **[B]** Absorption spectra of **2d-H** with 5 different concentrations and their **[C]** relative fluorescence spectra recorded using λ_{ex} = 360 nm. **[D]** Integrated fluorescence intensity versus absorption plot. **[E]** Phosphorescence spectra of singlet oxygen, samples excited at λ_{ex} = 375 nm and spectra collected in the NIR range between 1200 – 1350 nm. **[F]** Integrated phosphorescence intensity versus absorption plot. **[G]** Transient absorption spectra of **2d-H** using a laser pulse at λ_{exc} = 360 nm and energy 0.5 mJ, collected between 350 -700 nm using a 10 nm interval, (Ar-equilibrated solution). **[H]** Transient absorption kinetics at selective wavelength (Ar-equilibrated solution).

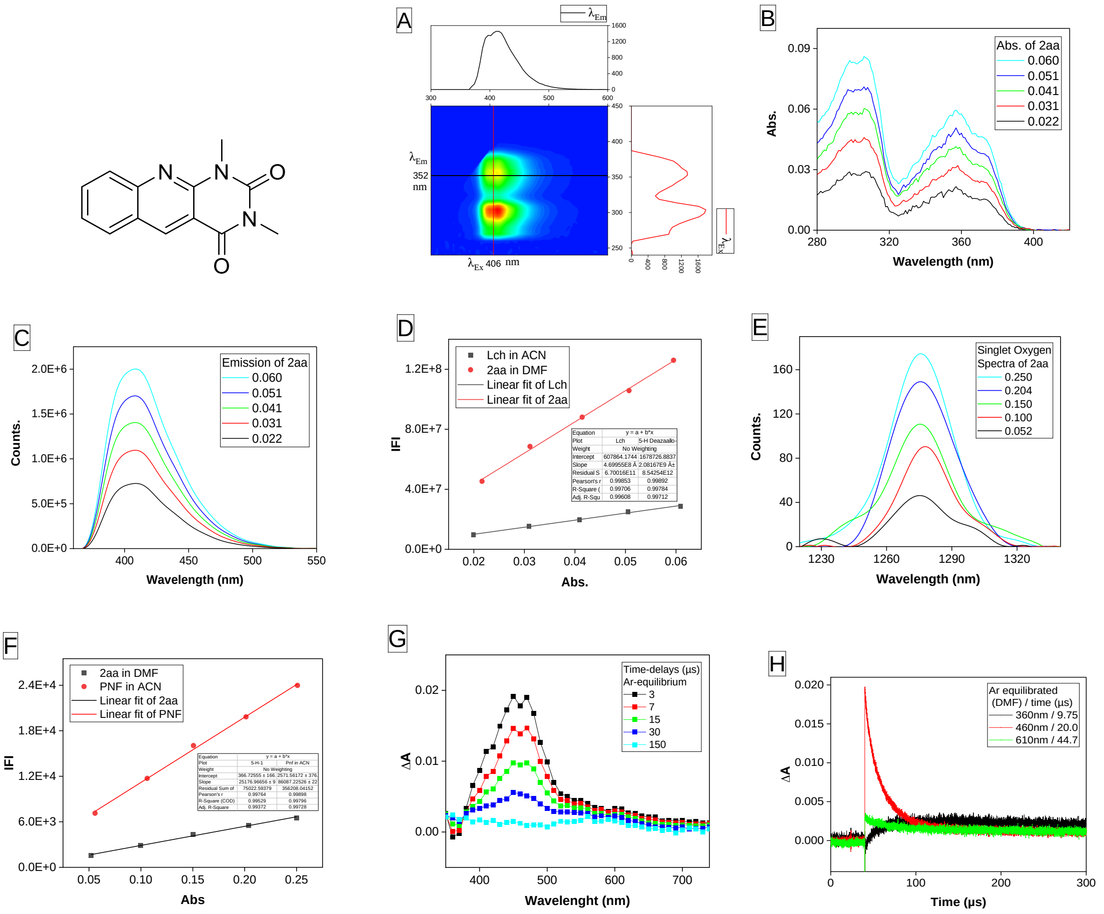

Figure S9: Photophysical data of a compound **2e**. **Panel-[A]** represents a fully corrected emission excitation matrix in DMF, **[B]** absorption spectra of **2e** with 5 different concentrations and their **[C]** relative fluorescence spectra recorded using λ_{ex} = 357 nm. **[D]** Integrated fluorescence intensity versus absorption plot. **[E]** Phosphorescence spectra of singlet oxygen, samples excited at λ_{ex} = 375 nm and spectra collected in the NIR range between 1200 – 1350 nm. **[F]** Integrated phosphorescence intensity versus absorption plot. **[G]** Transient absorption spectra of **2e** using a laser pulse at λ_{exc} = 360 nm and energy 0.5 mJ, collected between 350 -740 nm using a 10 nm interval, (Ar-equilibrated solution). **[H]** Transient absorption kinetics at selective wavelength (Ar-equilibrated solution).

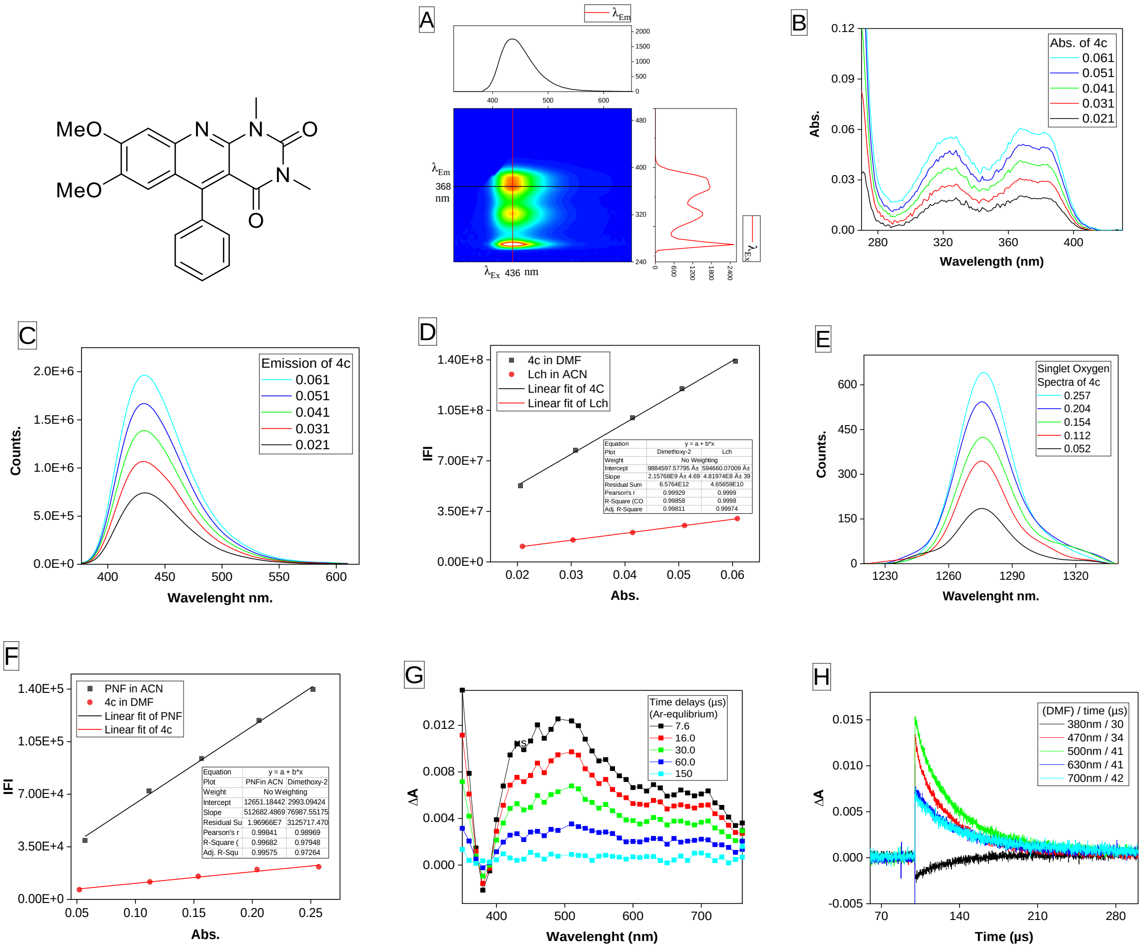

Figure S10: Photophysical data of a compound **3a(Ph)**. **Panel-[A]** represents a fully corrected emission excitation matrix in DMF. **[B]** Absorption spectra of **3a(Ph)** with 5 different concentrations and their **[C]** relative fluorescence spectra recorded using λ_{ex} = 367 nm. **[D]** Integrated fluorescence intensity versus absorption plot. **[E]** Phosphorescence spectra of singlet oxygen, samples excited at λ_{ex} = 375 nm and spectra collected in the NIR range between 1200 – 1350 nm. **[F]** Integrated phosphorescence intensity versus absorption plot. **[G]** Transient absorption spectra of **3a(Ph)** using a laser pulse at λ_{exc} = 370 nm and energy 0.5 mJ, collected between 350 -760 nm using a 10 nm interval, (Ar-equilibrated solution). **[H]** Transient absorption kinetics at selective wavelength (Ar-equilibrated solution).

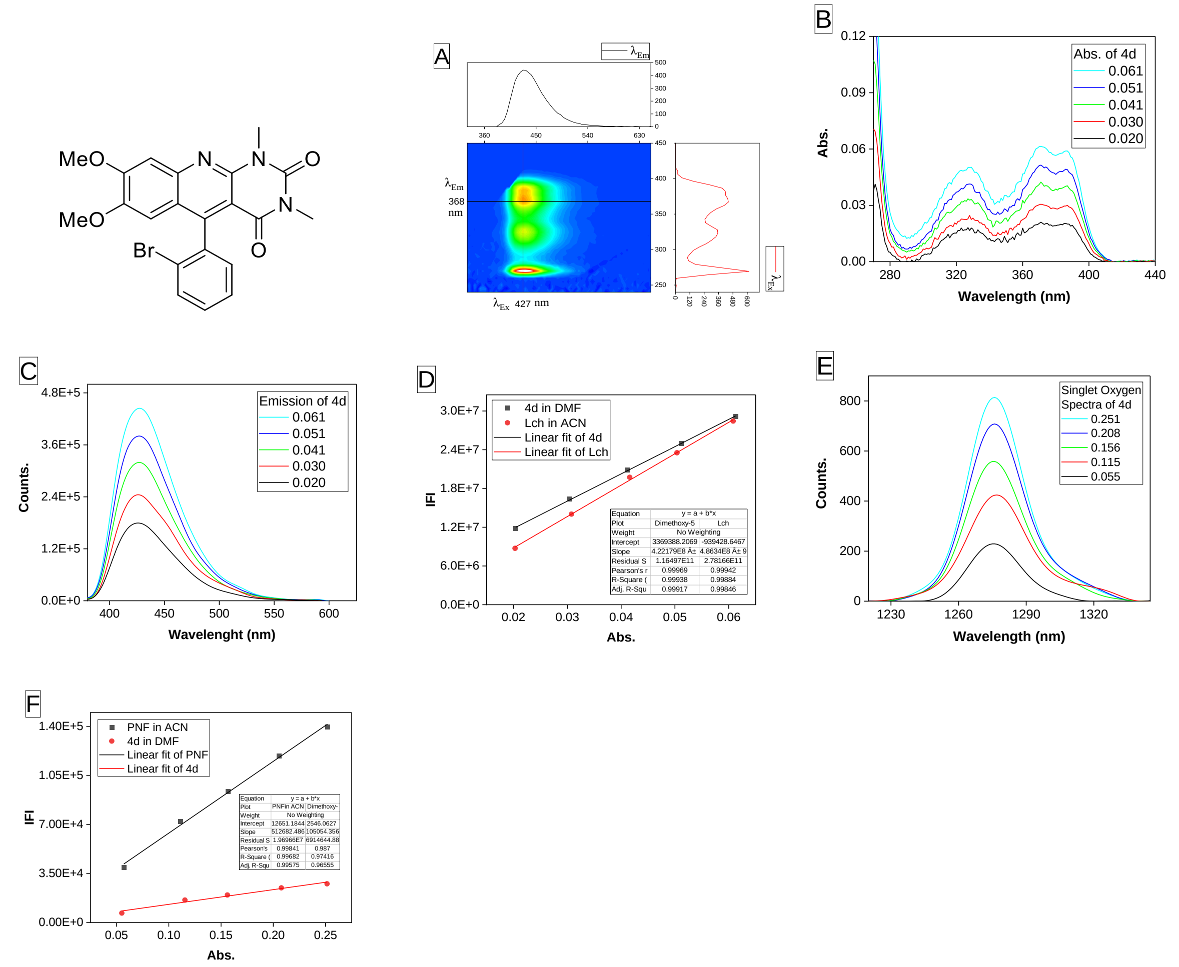

Figure S11: Photophysical data of a compound **3a(*o*-BrPh)**. **Panel-[A]** represents a fully corrected emission excitation matrix in DMF. **[B]** Absorption spectra of **3a(*o*-BrPh)** with 5 different concentrations and their **[C]** relative fluorescence spectra recorded using $\lambda_{ex} = 370$ nm. **[D]** Integrated fluorescence intensity versus absorption plot. **[E]** Phosphorescence spectra of singlet oxygen, samples excited at $\lambda_{ex} = 375$ nm and spectra collected in the NIR range between 1200 – 1350 nm. **[F]** Integrated phosphorescence intensity versus absorption plot.

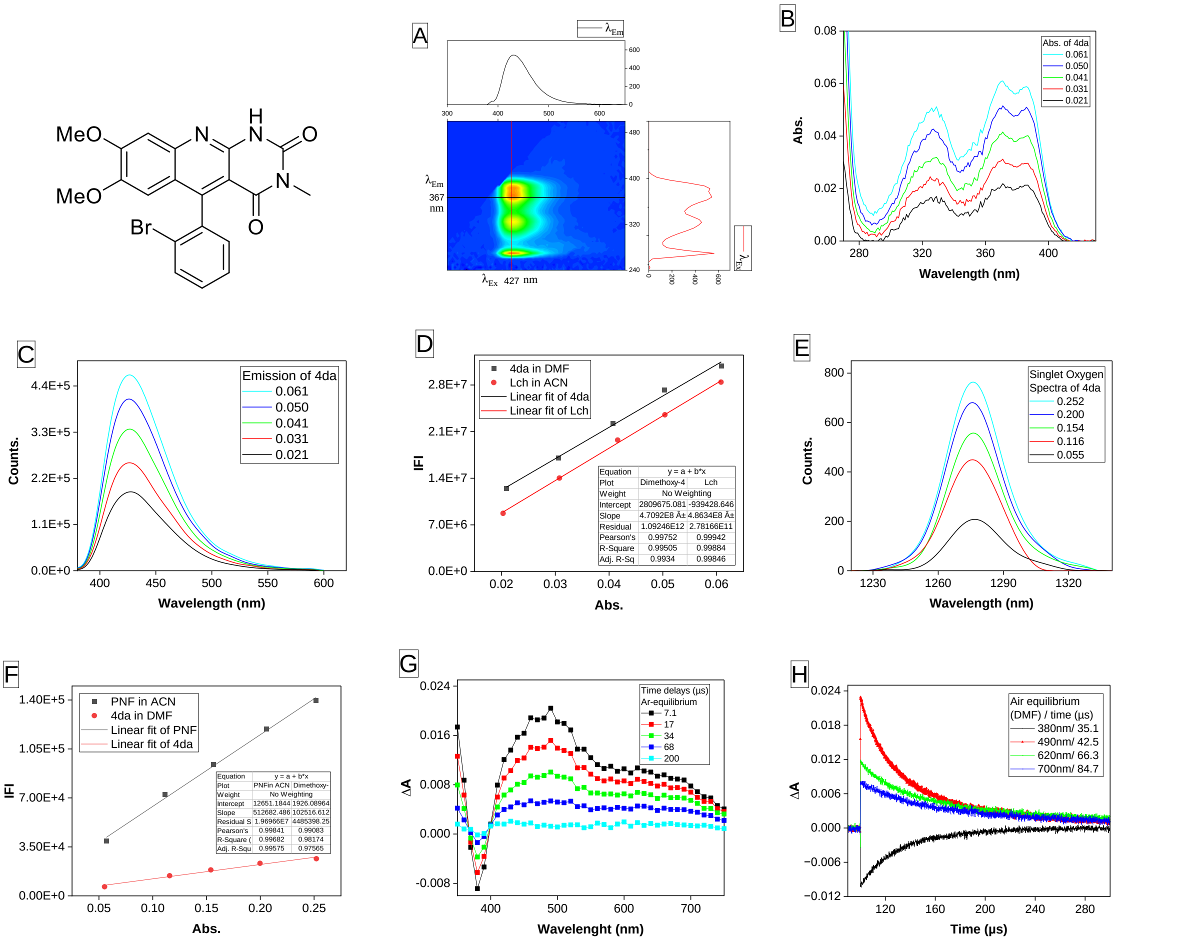

Figure S12: Photophysical data of a compound **3a(*o*-BrPh)-H**. **Panel-[A]** represents a fully corrected emission excitation matrix in DMF. **[B]** Absorption spectra of **3a(*o*-BrPh)-H** with 5 different concentrations and their **[C]** relative fluorescence spectra recorded using λ_{ex} = 370 nm. **[D]** Integrated fluorescence intensity versus absorption plot. **[E]** Phosphorescence spectra of singlet oxygen, samples excited at λ_{ex} = 375 nm and spectra collected in the NIR range between 1200 – 1350 nm. **[F]** Integrated phosphorescence intensity versus absorption plot. **[G]** Transient absorption spectra of **3a(*o*-BrPh)-H** using a laser pulse at λ_{exc} = 370 nm and energy 0.5 mJ, collected between 350 -760 nm using a 10 nm interval, (Ar-equilibrated solution). **[H]** Transient absorption kinetics at selective wavelength (Ar-equilibrated solution).

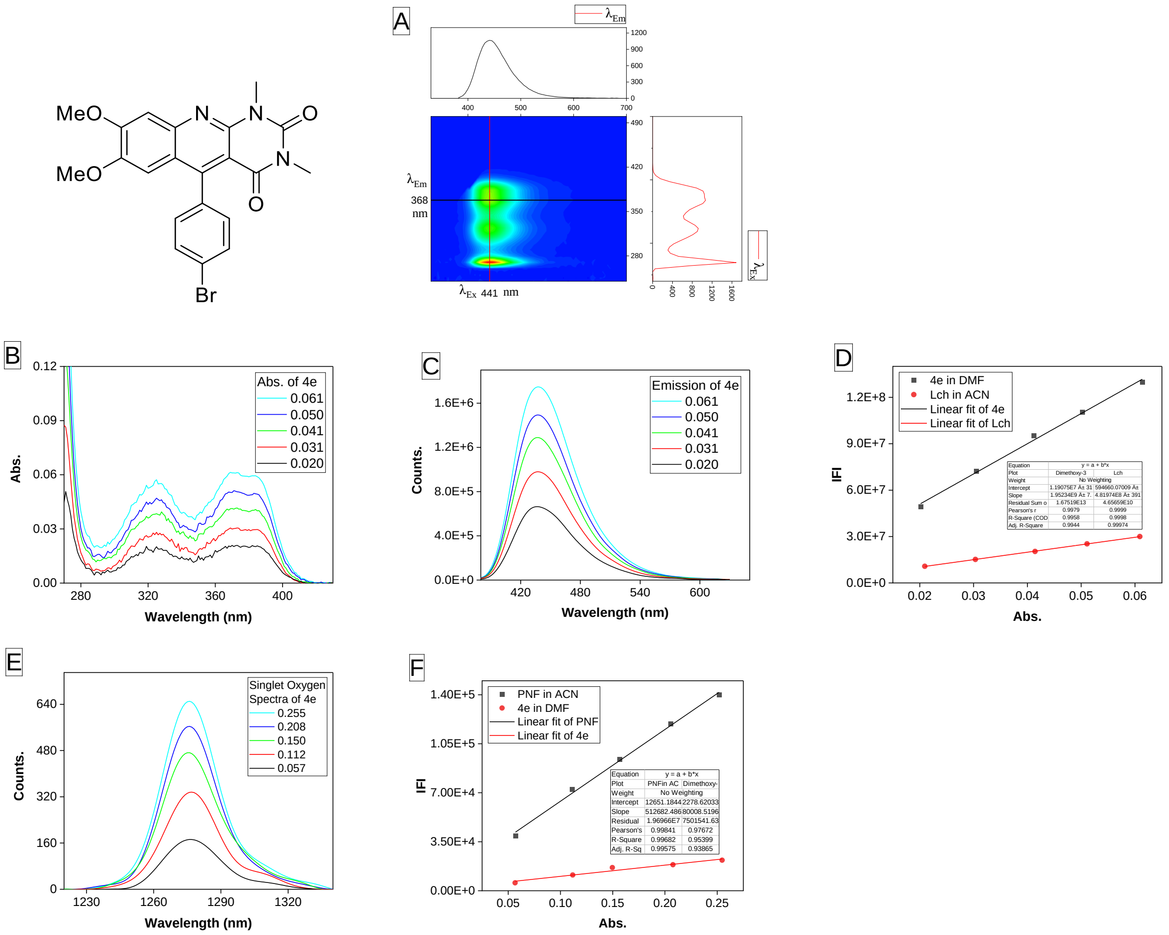

Figure S13: Photophysical data of a compound **3a(*p*-BrPh)**. **Panel-[A]** represents a fully corrected emission excitation matrix in DMF. **[B]** Absorption spectra of **3a(*p*-BrPh)** with 5 different concentrations and their **[C]** relative fluorescence spectra recorded using $\lambda_{ex} = 369$ nm. **[D]** Integrated fluorescence intensity versus absorption plot. **[E]** Phosphorescence spectra of singlet oxygen, samples excited at $\lambda_{ex} = 375$ nm and spectra collected in the NIR range between 1200 – 1350 nm. **[F]** Integrated phosphorescence intensity versus absorption plot.

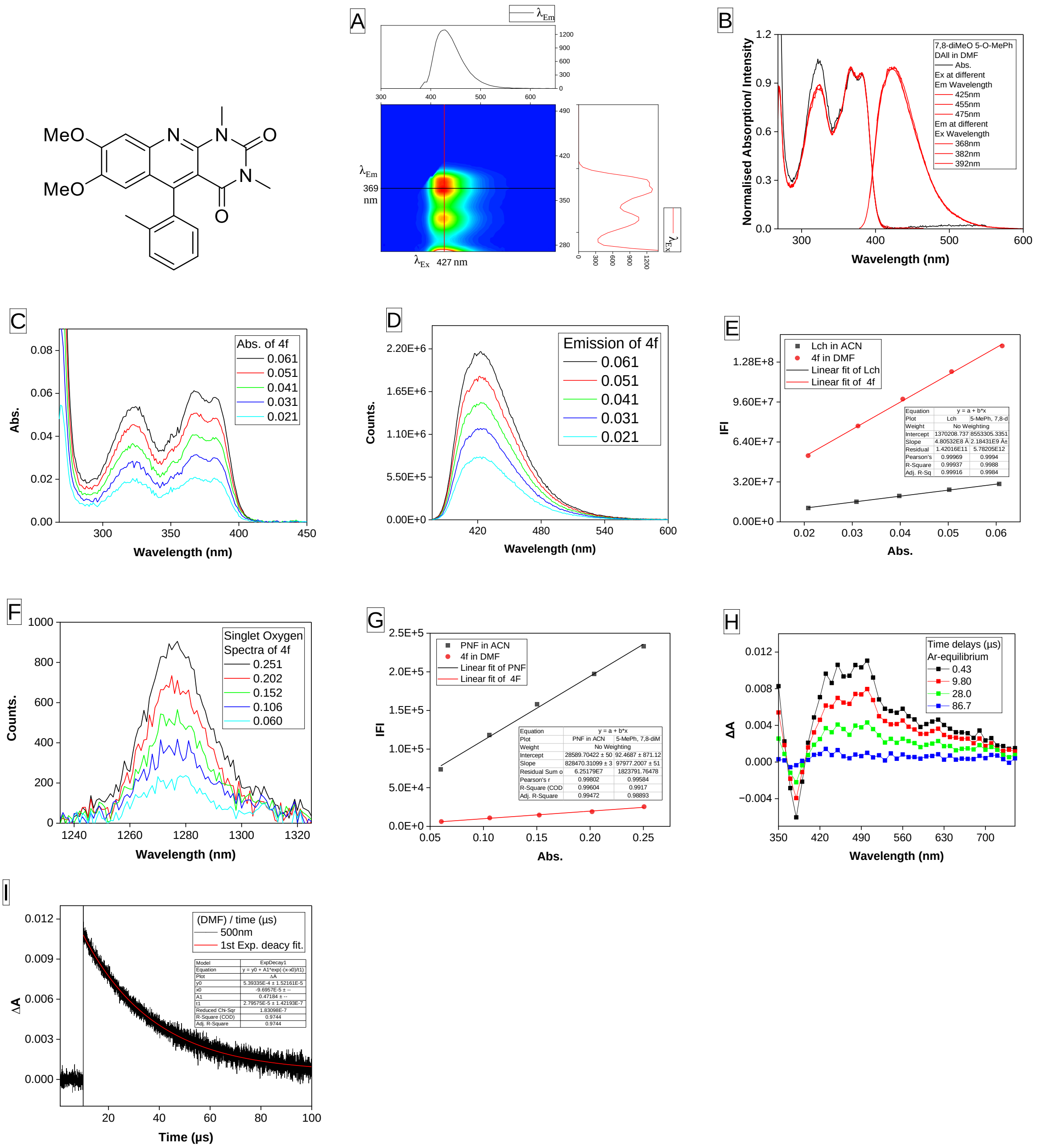

Figure S14: Photophysical data of a compound **3a(*o*-MePh)**. **Panel-[A]** represents a fully corrected emission excitation matrix in DMF. **[B]** UV-Vis absorption spectrum in DMF, steady-state emission spectra recorded at varying excitation wavelengths, excitation spectra collected at selected emission wavelengths. **[C]** Absorption spectra of **3a(*o*-MePh)** with 5 different concentrations and their **[D]** relative fluorescence spectra recorded using λ_{ex} = 367 nm. **[E]** Integrated fluorescence intensity versus absorptionplot. **[F]** Phosphorescence spectra of singlet oxygen, samples excited at λ_{ex} = 382 nm and spectra collected in NIR range between 1200 – 1350 nm. **[G]** Integrated phosphorescence intensity versus absorption plot. **[H]** Transient absorption spectra of **3a(*o*-MePh)** using a laser pulse at λ_{exc} = 385 nm and energy 0.5 mJ, collected between 350 -760 nm using a 10 nm interval, (Ar-equilibrated solution). **[I]** Transient absorption kinetic (Black) and decay fit in red (Ar-equilibrated solution).

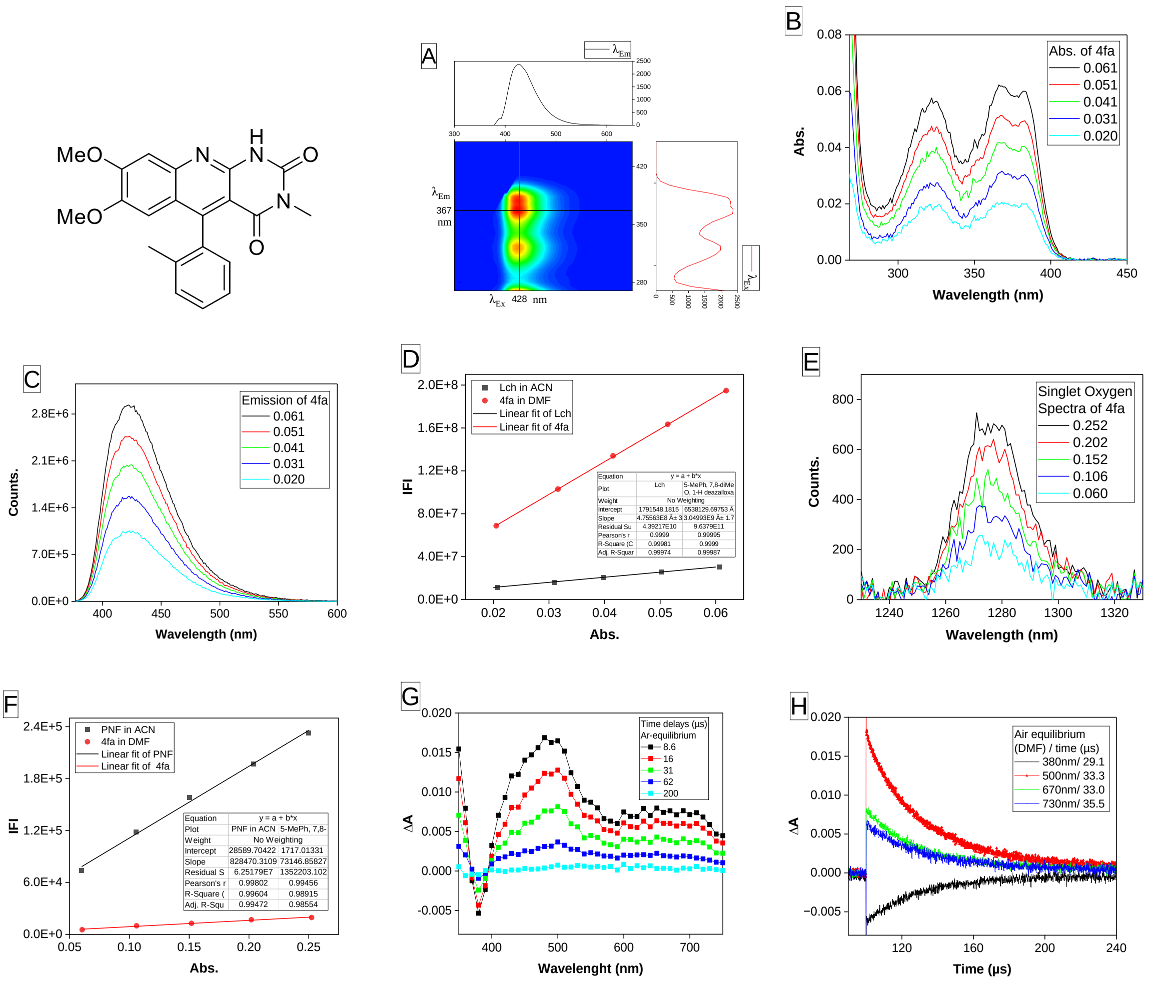

Figure S15: Photophysical data of a compound **3a(*o*-MePh)-H**. **Panel-[A]** represents a fully corrected emission excitation matrix in DMF. **[B]** Absorption spectra of **3a(*o*-MePh)-H** with 5 different concentrations and their **[C]** relative fluorescence spectra recorded using λ_{ex} = 368 nm. **[D]** Integrated fluorescence intensity versus absorption plot. **[E]** Phosphorescence spectra of singlet oxygen, samples excited at λ_{ex} = 382 nm and spectra collected in the NIR range between 1200 – 1350 nm. **[F]** Integrated phosphorescence intensity versus absorption plot. **[G]** Transient absorption spectra of **3a(*o*-MePh)-H** using a laser pulse at λ_{exc} = 370 nm and energy 0.5 mJ, collected between 350 -760 nm using a 10 nm interval, (Ar-equilibrated solution). **[H]** Transient absorption kinetics at selective wavelength (Ar-equilibrated solution).

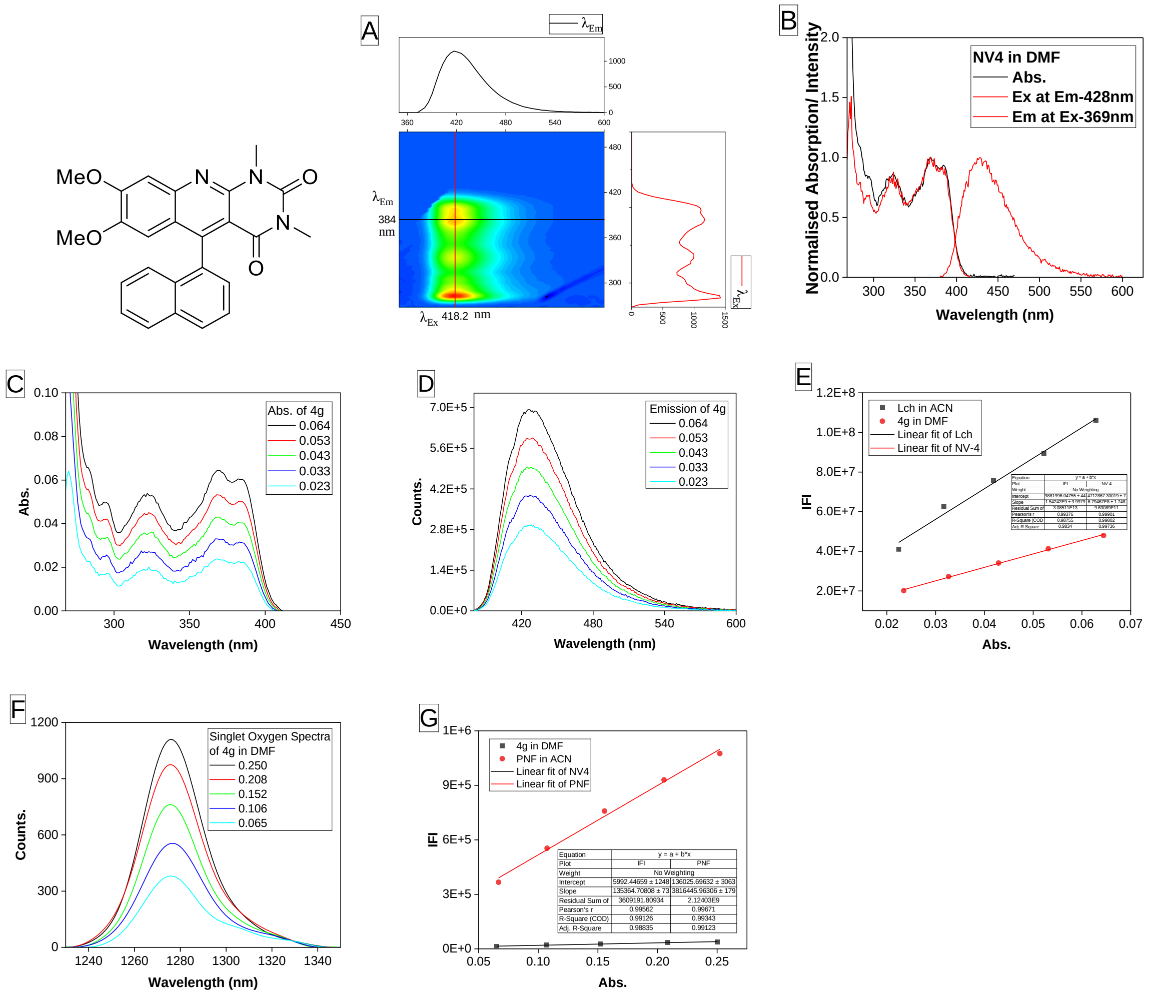

Figure S16: Photophysical data of a compound **3a(napht-1-yl)**. **Panel-[A]** represents a fully corrected emission excitation matrix in DMF. **[B]** UV-Vis absorption spectrum in DMF, steady-state emission spectra recorded at excitation wavelengths λ_{ex} = 369 nm, excitation spectra collected at emission wavelengths λ_{em} = 428 nm **[C]** Absorption spectra of **3a(napht-1-yl)** with 5 different concentrations and their **[D]** relative fluorescence spectra recorded using λ_{ex} = 370 nm. **[E]** Integrated fluorescence intensity versus absorption plot. **[F]** Phosphorescence spectra of singlet oxygen, samples excited at λ_{ex} = 370 nm and spectra collected in NIR range between 1200 – 1350 nm. **[G]** Integrated phosphorescence intensity versus absorption plot.

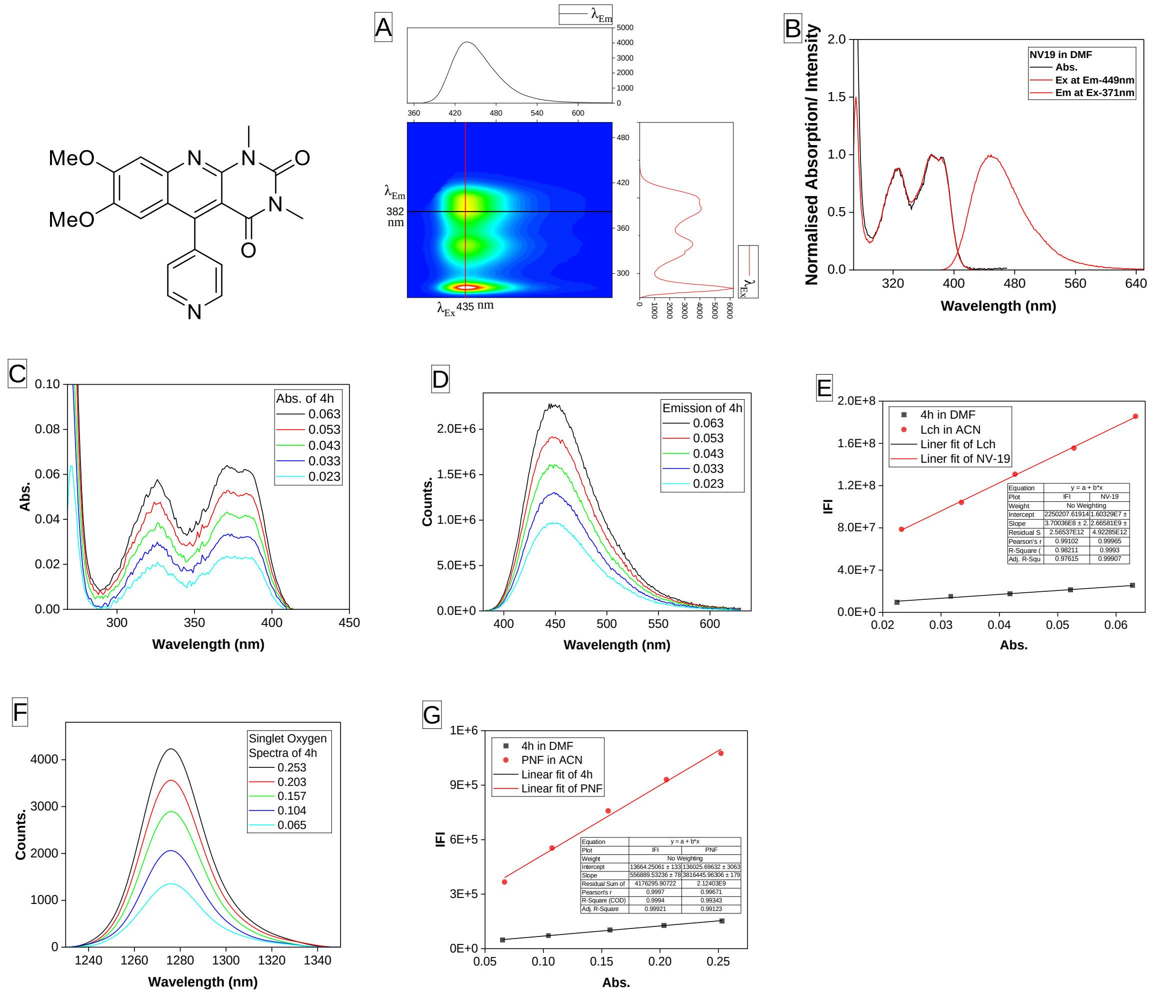

Figure S17: Photophysical data of a compound **3a(pyridine-4-yl)**. **Panel-[A]** represents a fully corrected emission excitation matrix in DMF, **[B]** UV-Vis absorption spectrum in DMF, steady-state emission spectra recorded at excitation wavelengths λ_{ex} = 371 nm, excitation spectra collected at emission wavelengths λ_{em} = 449 nm. **[C]** Absorption spectra of **3a(pyridine-4-yl)** with 5 different concentrations and their **[D]** relative fluorescence spectra recorded using λ_{ex} = 372 nm. **[E]** Integrated fluorescence intensity versus absorption plot. **[F]** Phosphorescence spectra of singlet oxygen, samples excited at λ_{ex} = 370 nm and spectra collected in NIR range between 1200 – 1350 nm. **[G]** Integrated phosphorescence intensity versus absorption plot.

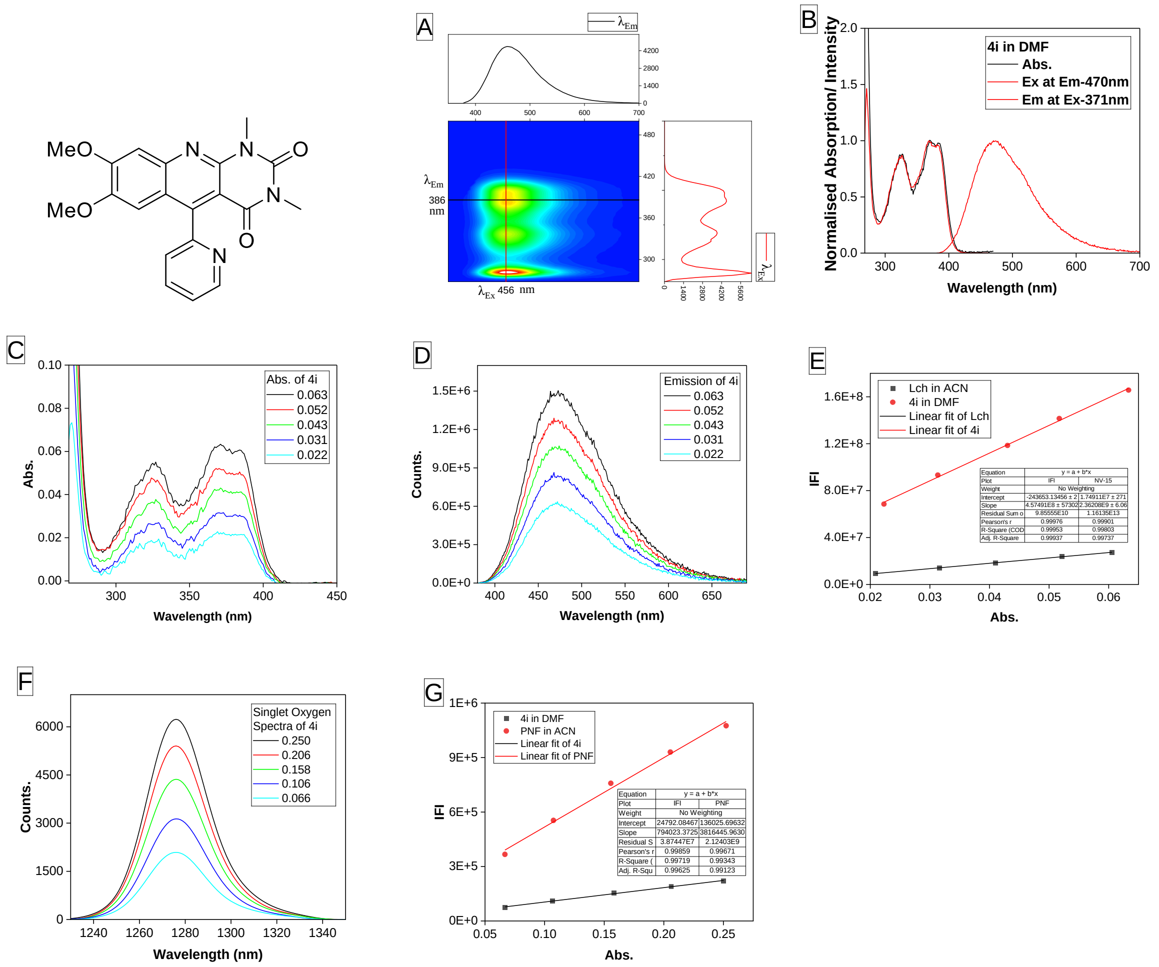

Figure S18: Photophysical data of a compound **3a(pyridine-2-yl)**. **Panel-[A]** represents a fully corrected emission excitation matrix in DMF. **[B]** UV-Vis absorption spectrum in DMF, steady-state emission spectra recorded at excitation wavelengths λ_{ex} = 371 nm, excitation spectra collected at emission wavelengths λ_{ex} = 470 nm. **[C]** Absorption spectra of **3a(pyridine-2-yl)** with 5 different concentrations and their **[D]** relative fluorescence spectra recorded using λ_{ex} = 371 nm. **[E]** Integrated fluorescence intensity versus absorption plot. **[F]** Phosphorescence spectra of singlet oxygen, samples excited at λ_{ex} = 370 nm and spectra collected in NIR range between 1200 – 1350 nm. **[G]** Integrated phosphorescence intensity versus absorption plot.

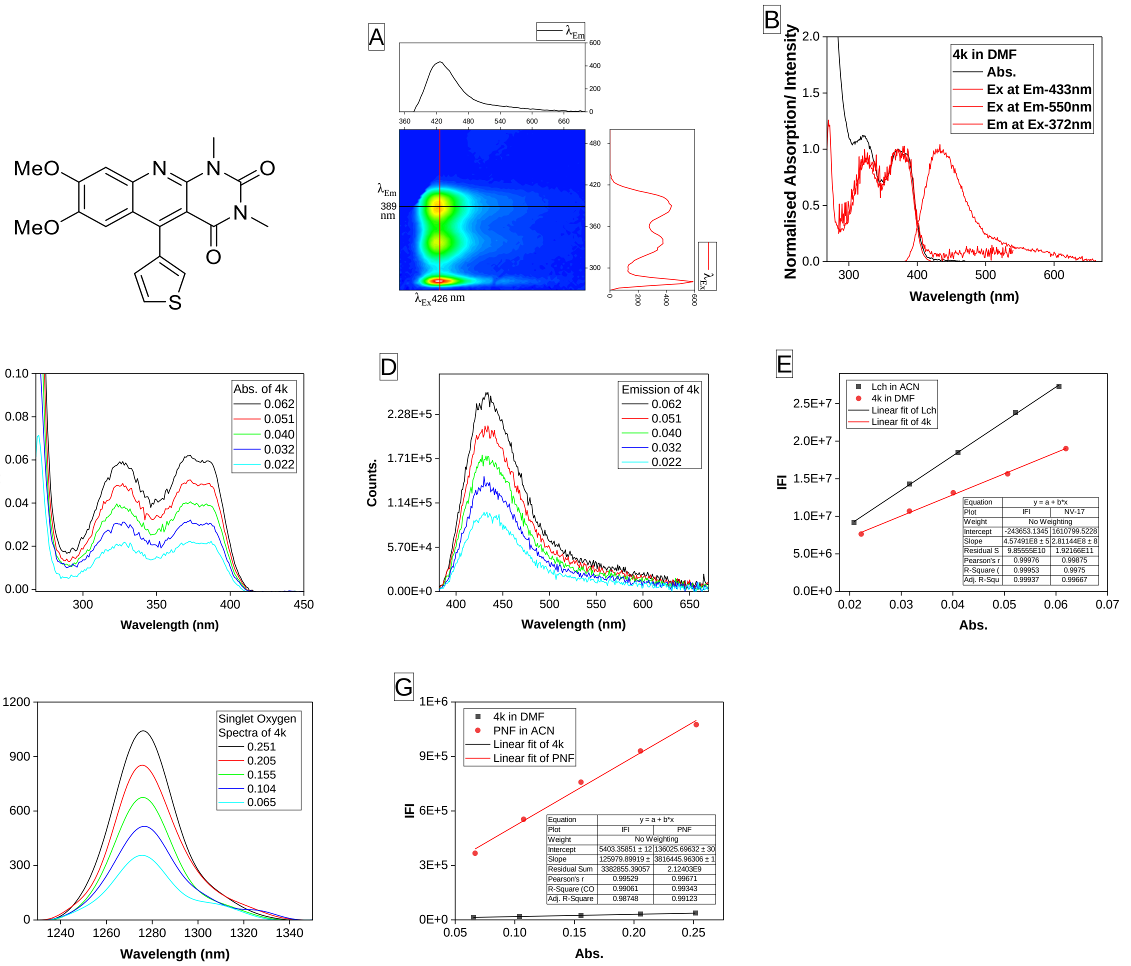

Figure S19: Photophysical data of a compound **3a(thiophen-3-yl)**. **Panel-[A]** represents a fully corrected emission excitation matrix in DMF. **[B]** UV-Vis absorption spectrum in DMF, steady-state emission spectra recorded at excitation wavelengths λ_{ex} = 372 nm, excitation spectra collected at selected emission wavelengths. **[C]** Absorption spectra of **3a(thiophen-3-yl)** with 5 different concentrations and their **[D]** relative fluorescence spectra recorded using λ_{ex} = 372 nm. **[E]** Integrated fluorescence intensity versus absorption plot. **[F]** Phosphorescence spectra of singlet oxygen, samples excited at λ_{ex} = 370 nm and spectra collected in NIR range between 1200 – 1350 nm. **[G]** Integrated phosphorescence intensity versus absorption plot.

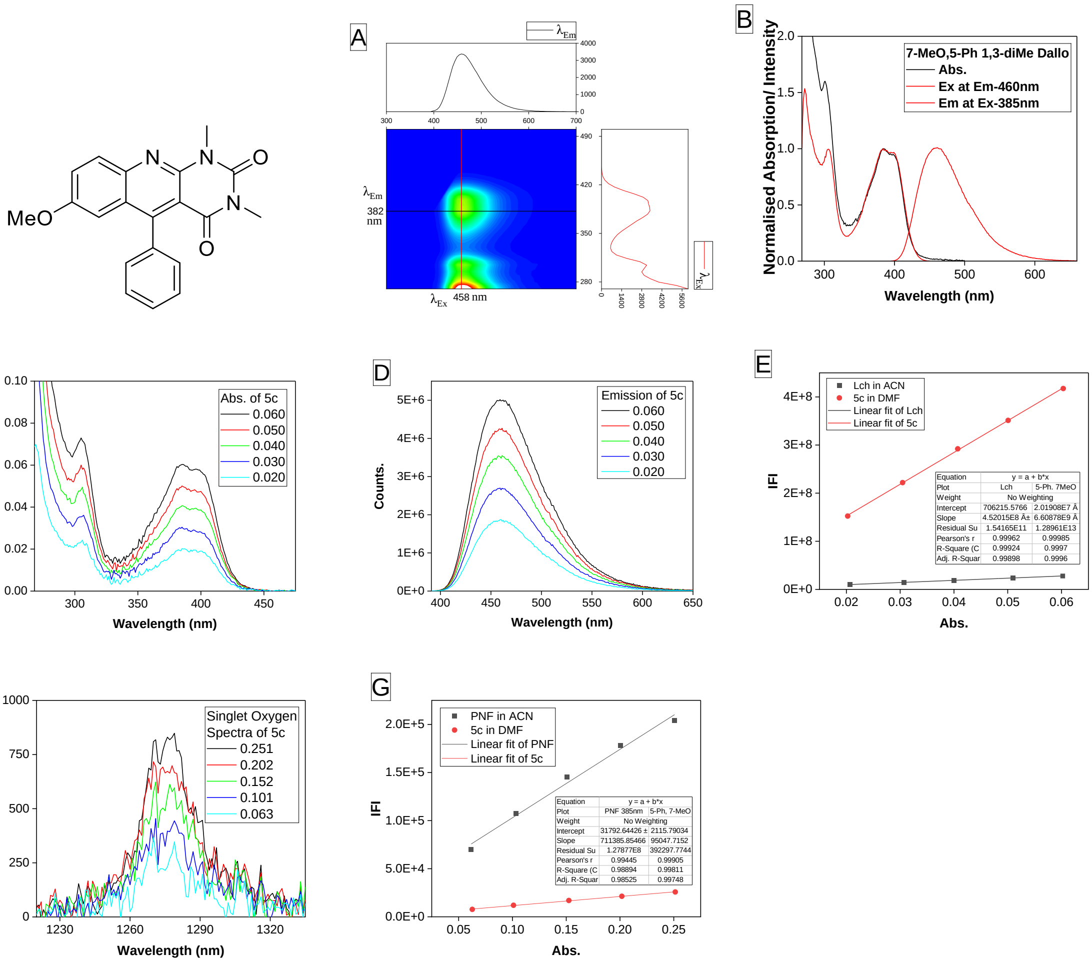

Figure S20: Photophysical data of a compound **3b(Ph)**. **Panel-[A]** represents a fully corrected emission excitation matrix in DMF. **[B]** UV-Vis absorption spectrum in DMF, steady-state emission spectra recorded at excitation wavelengths λ_{ex} = 385 nm, excitation spectra collected at emission wavelengths λ_{em} = 460 nm. **[C]** Absorption spectra of **3b(Ph)** with 5 different concentrations and their **[D]** relative fluorescence spectra recorded using λ_{ex} = 385 nm. **[E]** Integrated fluorescence intensity versus absorption plot. **[F]** Phosphorescence spectra of singlet oxygen, samples excited at λ_{ex} = 385 nm and spectra collected in NIR range between 1200 – 1350 nm. **[G]** Integrated phosphorescence intensity versus absorption plot.

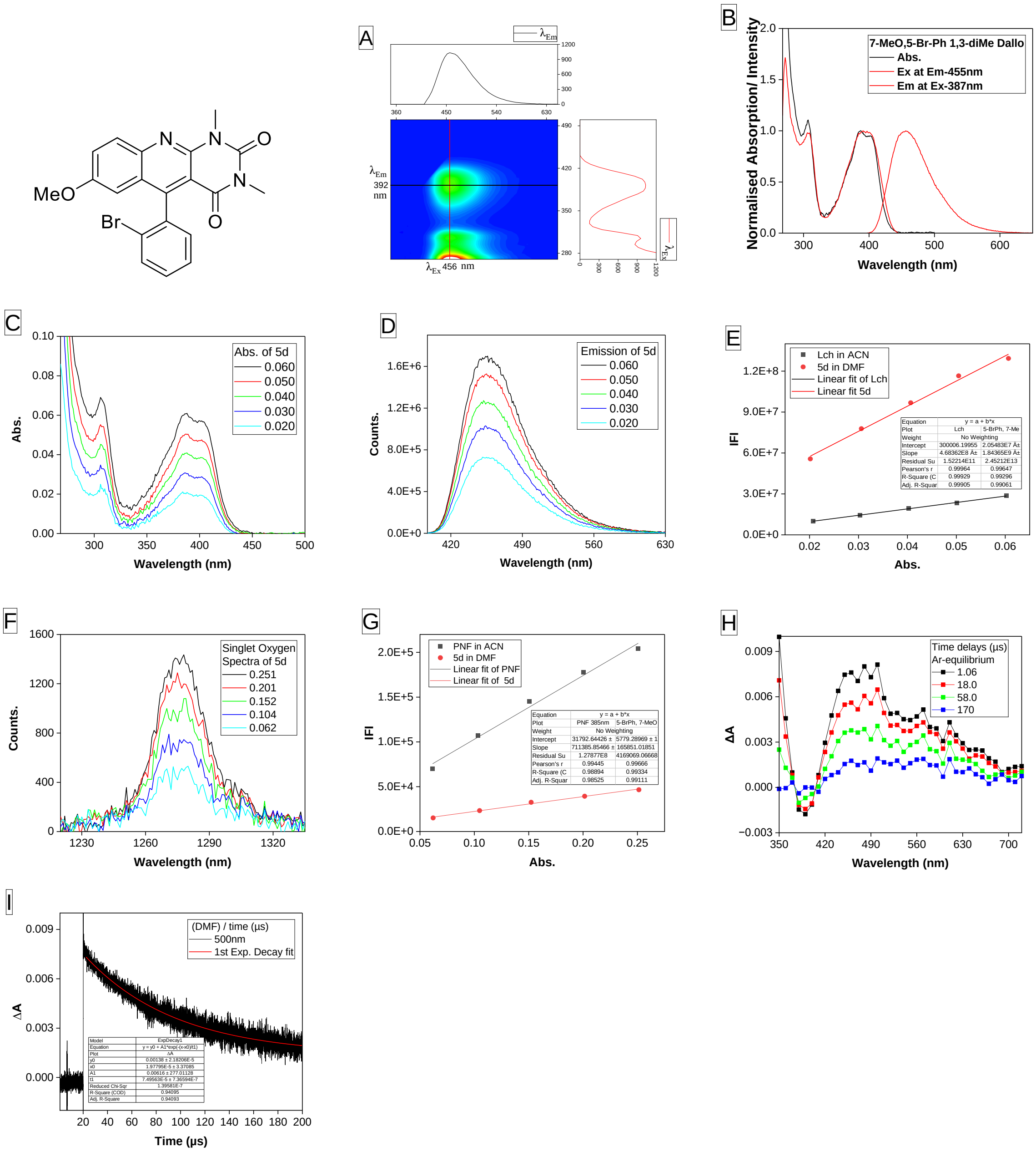

Figure S21: Photophysical data of a compound **3b(*o*-BrPh)**. **Panel-[A]** represents a fully corrected emission excitation matrix in DMF. **[B]** UV-Vis absorption spectrum in DMF, steady-state emission spectra recorded at excitation wavelengths λ_{ex} = 387 nm, excitation spectra collected at emission wavelengths λ_{em} = 455 nm. **[C]** Absorption spectra of **3b(*o*-BrPh)** with 5 different concentrations and their **[D]** relative fluorescence spectra recorded using λ_{ex} = 387 nm. **[E]** Integrated fluorescence intensity versus absorption plot. **[F]** Phosphorescence spectra of singlet oxygen, samples excited at λ_{ex} = 385 nm and spectra collected in NIR range between 1200 – 1350 nm. **[G]** Integrated phosphorescence intensity versus absorption plot. **[H]** Transient absorption spectra of **3b(*o*-BrPh)** using a laser pulse atλ_{exc} = 385 nm and energy 0.5 mJ, collected between 350 -760 nm using a 10 nm interval, (Ar-equilibrated solution). **[I]** Transient absorption kinetic (black) and fit (red) (Ar-equilibrated solution).

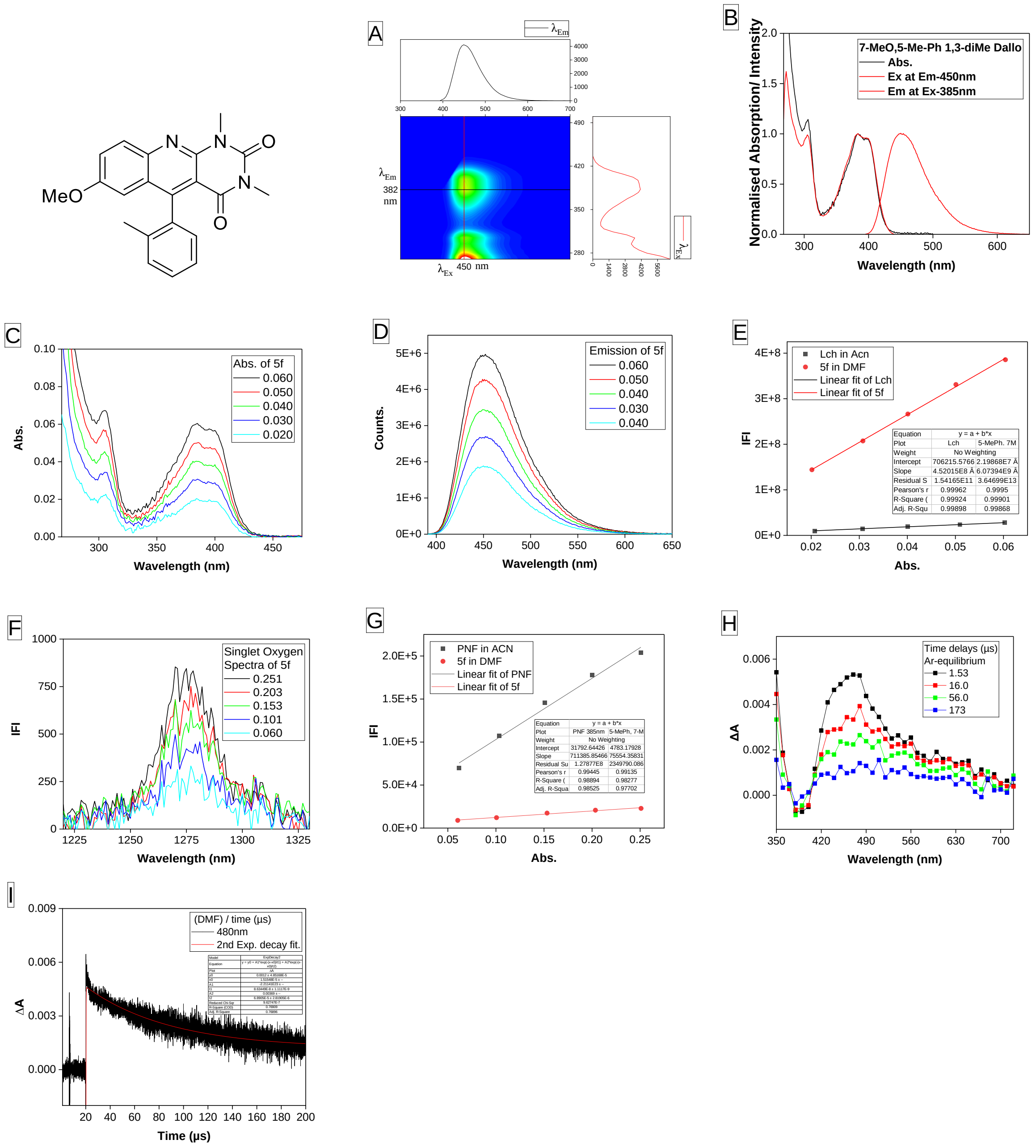

Figure S22: Photophysical data of a compound **3b(*o*-MePh)**. **Panel-[A]** represents a fully corrected emission excitation matrix in DMF. **[B]** UV-Vis absorption spectrum in DMF, steady-state emission spectra recorded at excitation wavelengths λ_{ex} = 385 nm, excitation spectra collected at emission wavelengths λ_{em} = 450 nm. **[C]** Absorption spectra of **3b(*o*-MePh)** with 5 different concentrations and their **[D]** relative fluorescence spectra recorded using λ_{ex} = 385 nm. **[E]** Integrated fluorescence intensity versus absorption plot. **[F]** Phosphorescence spectra of singlet oxygen, samples excited at λ_{ex} = 385 nm and spectra collected in NIR range between 1200 – 1350 nm. **[G]** Integrated phosphorescence intensity versus absorption plot. **[H]** Transient absorption spectra of **3b(*o*-MePh)** using a laser pulse at λ_{exc} = 385 nm and energy 0.5 mJ, collected between 350 -760 nm using a 10 nm interval, (Ar-equilibrated solution). **[I]** Transient absorption kinetic (black) and fit (red) (Ar-equilibrated solution).

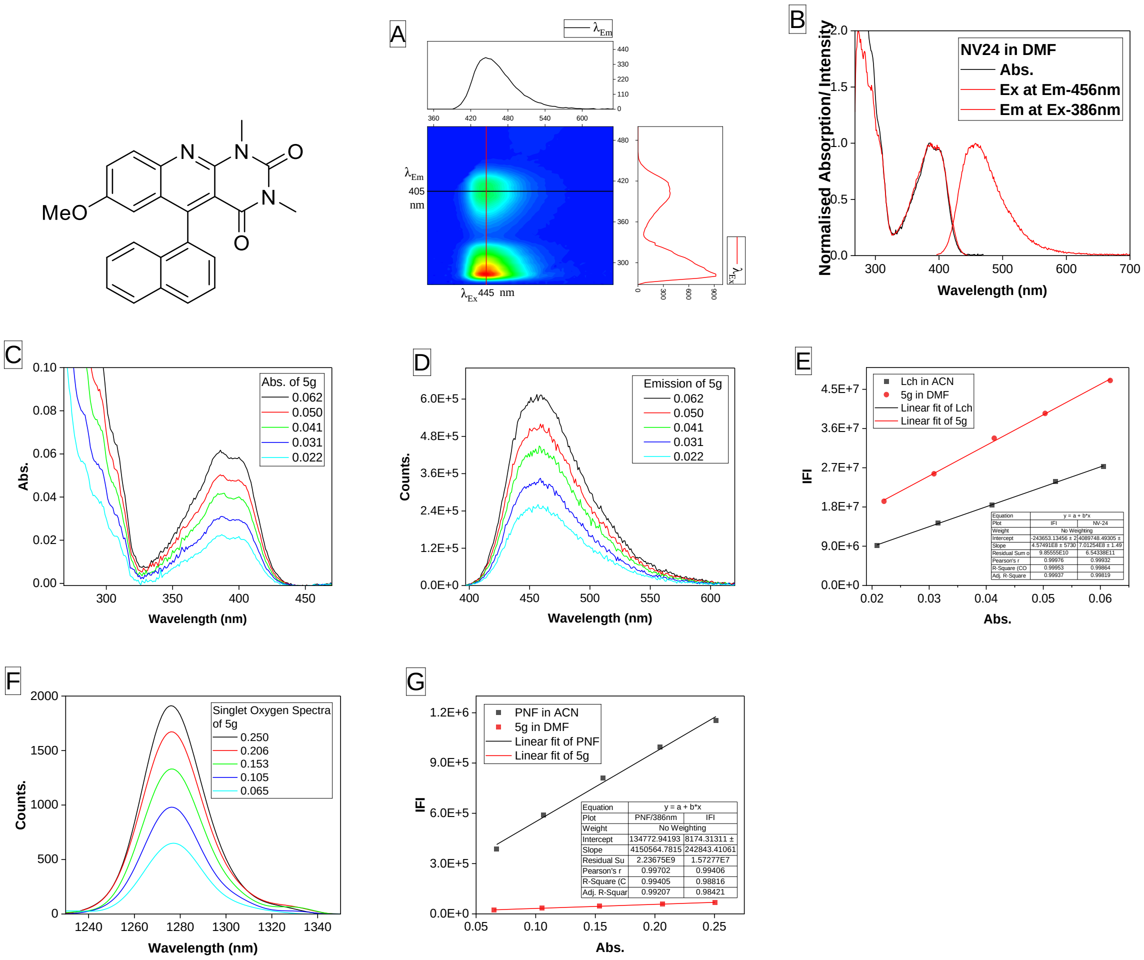

Figure S23: Photophysical data of a compound **3b(napht-1-yl)**. **Panel-[A]** represents a fully corrected emission excitation matrix in DMF. **[B]** UV-Vis absorption spectrum in DMF, steady-state emission spectra recorded at excitation wavelengths λ_{ex} = 386 nm, excitation spectra collected at emission wavelengths λ_{em} = 456 nm. **[C]** Absorption spectra of **3b(napht-1-yl)** with 5 different concentrations and their **[D]** relative fluorescence spectra recorded using λ_{ex} = 386 nm. **[E]** Integrated fluorescence intensity versus absorption plot. **[F]** Phosphorescence spectra of singlet oxygen, samples excited at λ_{ex} = 386 nm and spectra collected in NIR range between 1200 – 1350 nm. **[G]** Integrated phosphorescence intensity versus absorption plot.

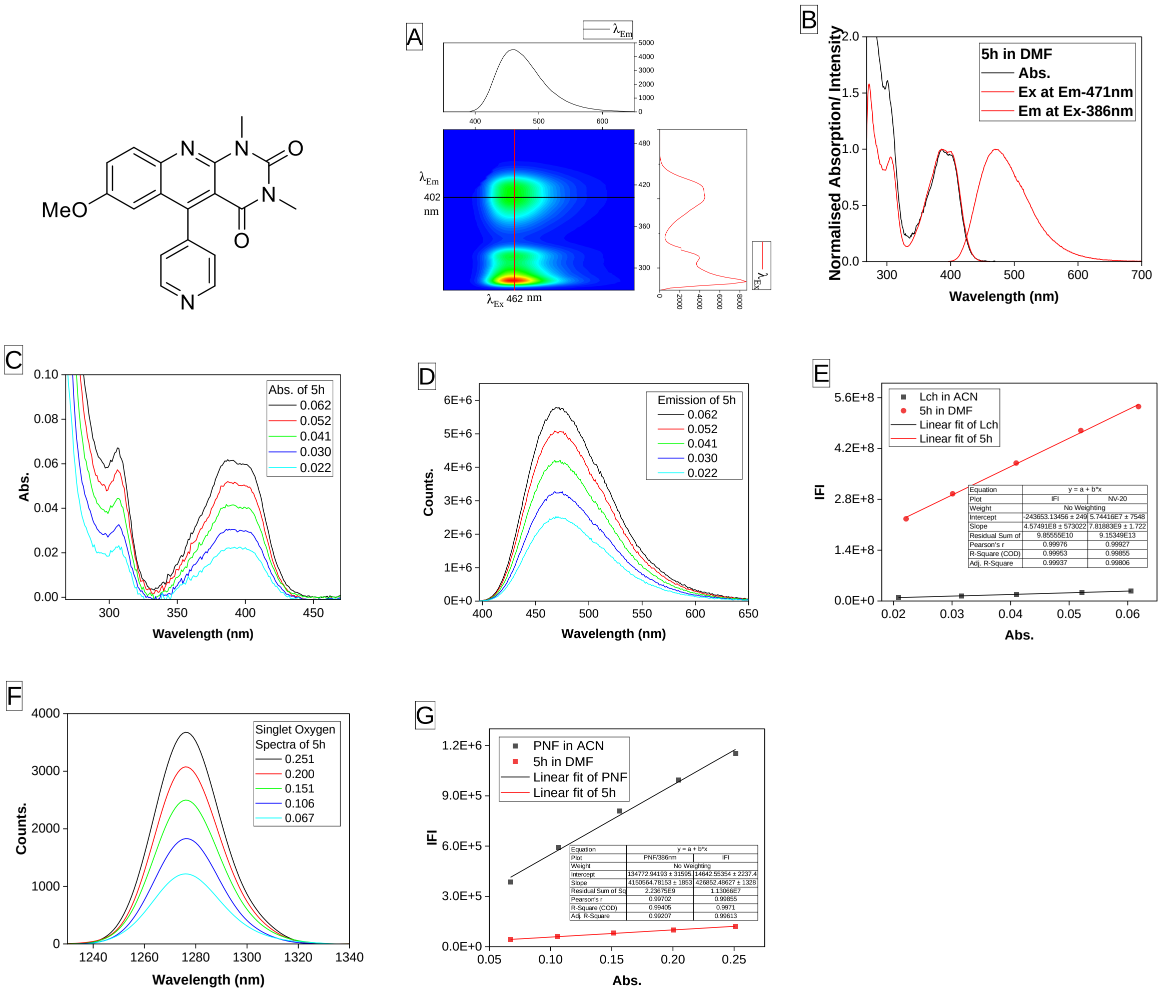

Figure S24: Photophysical data of a compound **3b(pyridin-4-yl)**. **Panel Panel-[A]** represents a fully corrected emission excitation matrix in DMF. **[B]** UV-Vis absorption spectrum in DMF, steady-state emission spectra recorded at excitation wavelengths λ_{ex} = 386 nm, excitation spectra collected at emission wavelengths λ_{em} = 471 nm. **[C]** Absorption spectra of **3b(pyridine-4-yl)** with 5 different concentrations and their **[D]** relative fluorescence spectra recorded using λ_{ex} = 387 nm. **[E]** Integrated fluorescence intensity versus absorption plot. **[F]** Phosphorescence spectra of singlet oxygen, samples excited at λ_{ex} = 386 nm and spectra collected in NIR range between 1200 – 1350 nm. **[G]** Integrated phosphorescence intensity versus absorption plot.

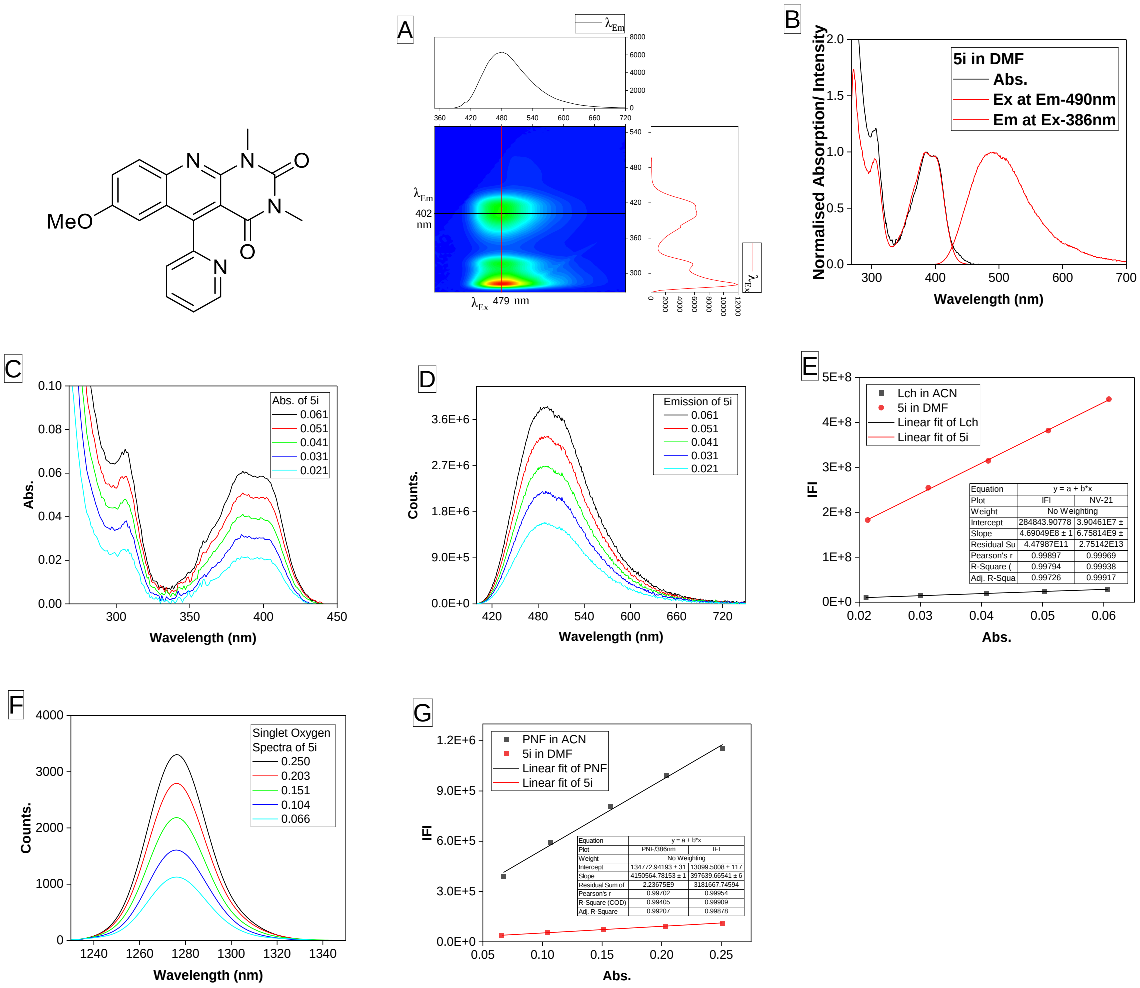

Figure S25: Photophysical data of a compound **3b(pyridin-2-yl)**. **Panel-[A]** represents a fully corrected emission excitation matrix in DMF. **[B]** UV-Vis absorption spectrum in DMF, steady-state emission spectra recorded at excitation wavelengths λ_{ex} = 386 nm, excitation spectra collected at emission wavelengths λ_{em} = 490 nm. **[C]** Absorption spectra of **3b(pyridine-2-yl)** with 5 different concentrations and their **[D]** relative fluorescence spectra recorded using λ_{ex} = 386 nm. **[E]** Integrated fluorescence intensity versus absorption plot. **[F]** Phosphorescence spectra of singlet oxygen, samples excited at λ_{ex} = 386 nm and spectra collected in NIR range between 1200 – 1350 nm. **[G]** Integrated phosphorescence intensity versus absorption plot.

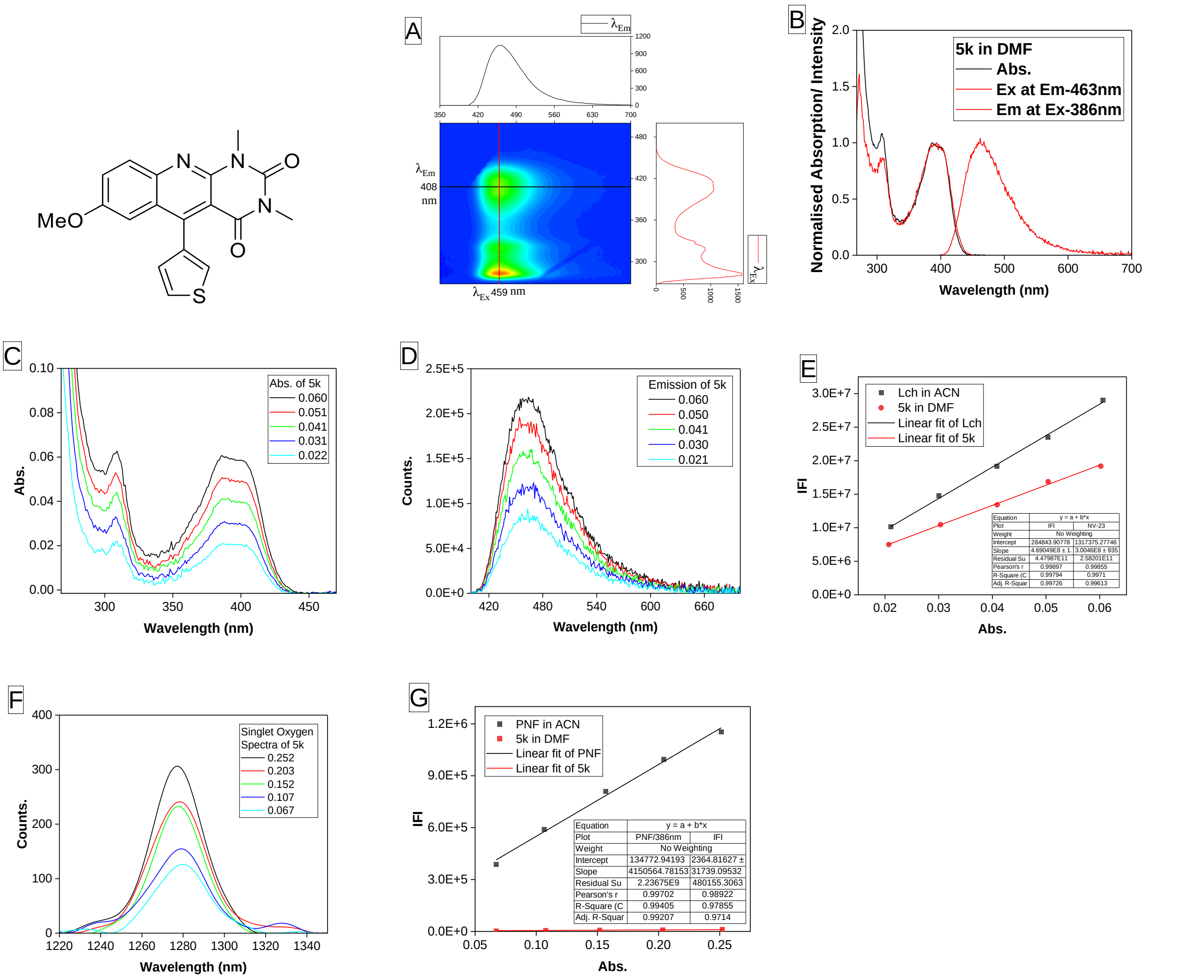

Figure S26: Photophysical data of a compound **3b(thiophen-3-yl)**. **Panel-[A]** represents a fully corrected emission excitation matrix in DMF. **[B]** UV-Vis absorption spectrum in DMF, steady-state emission spectra recorded at excitation wavelengths λ_{ex} = 386 nm, excitation spectra collected at emission wavelengths λ_{em} = 463 nm. **[C]** Absorption spectra of **3b(thiophen-3-yl)** with 5 different concentrations and their **[D]** relative fluorescence spectra recorded using λ_{ex} = 389 nm. **[E]** Integrated fluorescence intensity versus absorption plot. **[F]** Phosphorescence spectra of singlet oxygen, samples excited at λ_{ex} = 386 nm and spectra collected in NIR range between 1200 – 1350 nm. **[G]** Integrated phosphorescence intensity versus absorption plot.

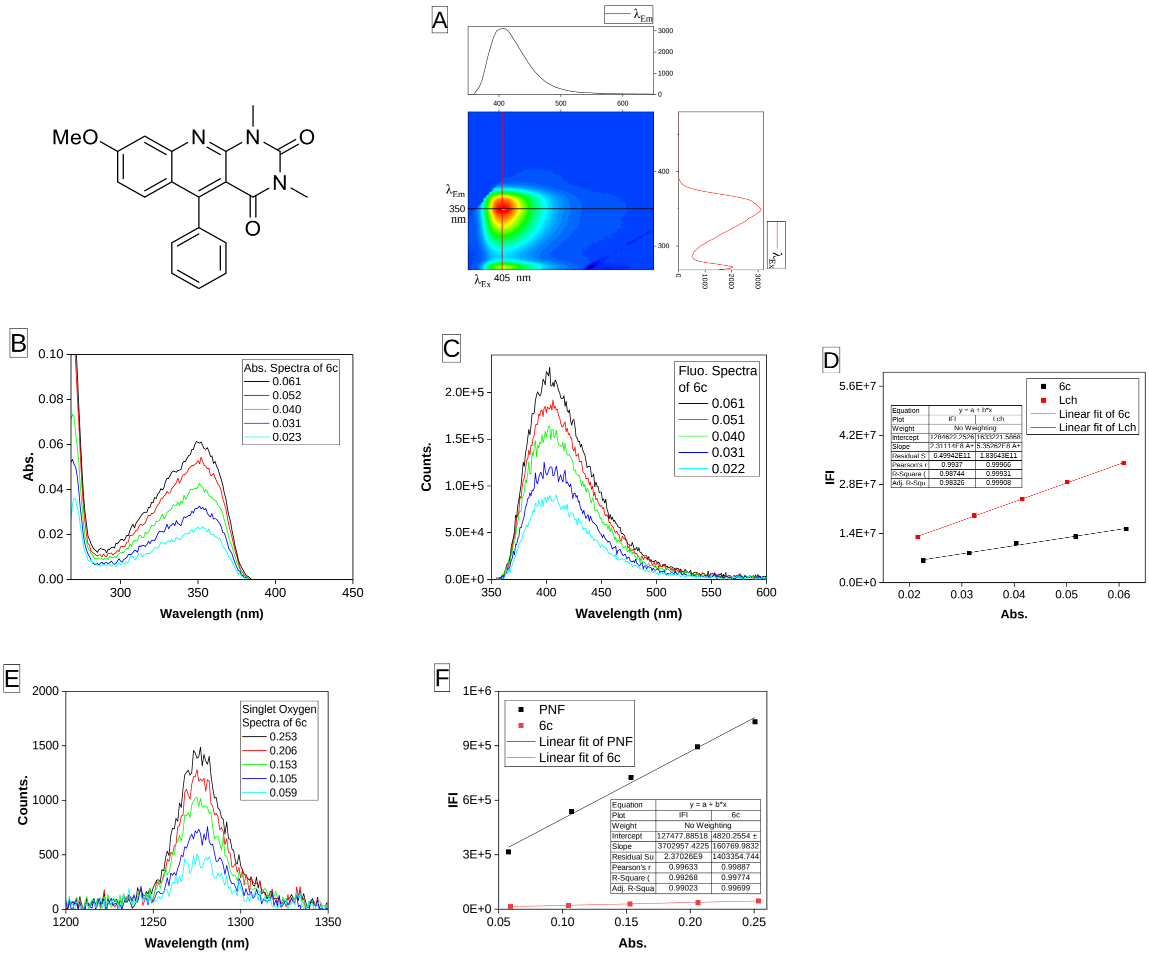

Figure S27: Photophysical data of a compound **3c(Ph)**. **Panel-[A]** represents a fully corrected emission excitation matrix in DMF. **[B]** Absorption spectra of **3c(Ph)** with 5 different concentrations and their **[C]** relative fluorescence spectra recorded using λ_{ex} = 349 nm. **[D]** Integrated fluorescence intensity versus absorption plot. **[E]** Phosphorescence spectra of singlet oxygen, samples excited at λ_{ex} = 365 nm and spectra collected in the NIR range between 1200 – 1350 nm. **[F]** Integrated phosphorescence intensity versus absorption plot.

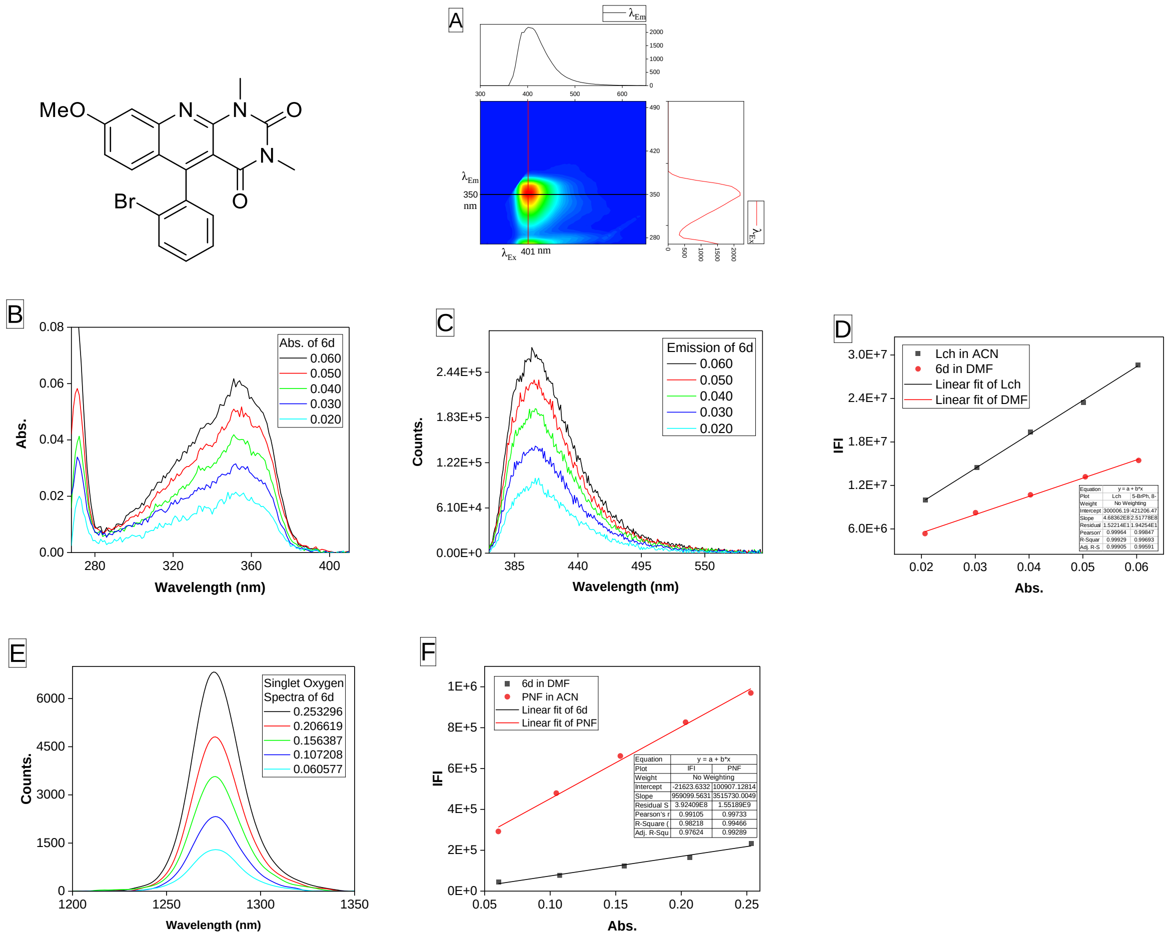

Figure S28: Photophysical data of a compound **3c(*o*-BrPh)**. **Panel-[A]** represents a fully corrected emission excitation matrix in DMF. **[B]** Absorption spectra of **3c(*o*-BrPh)** with 5 different concentrations and their **[C]** relative fluorescence spectra recorded using λ_{ex} = 353 nm. **[D]** Integrated fluorescence intensity versus absorption plot. **[E]** Phosphorescence spectra of singlet oxygen, samples excited at λ_{ex} = 357 nm and spectra collected in the NIR range between 1200 – 1350 nm. **[F]** Integrated phosphorescence intensity versus absorption plot.

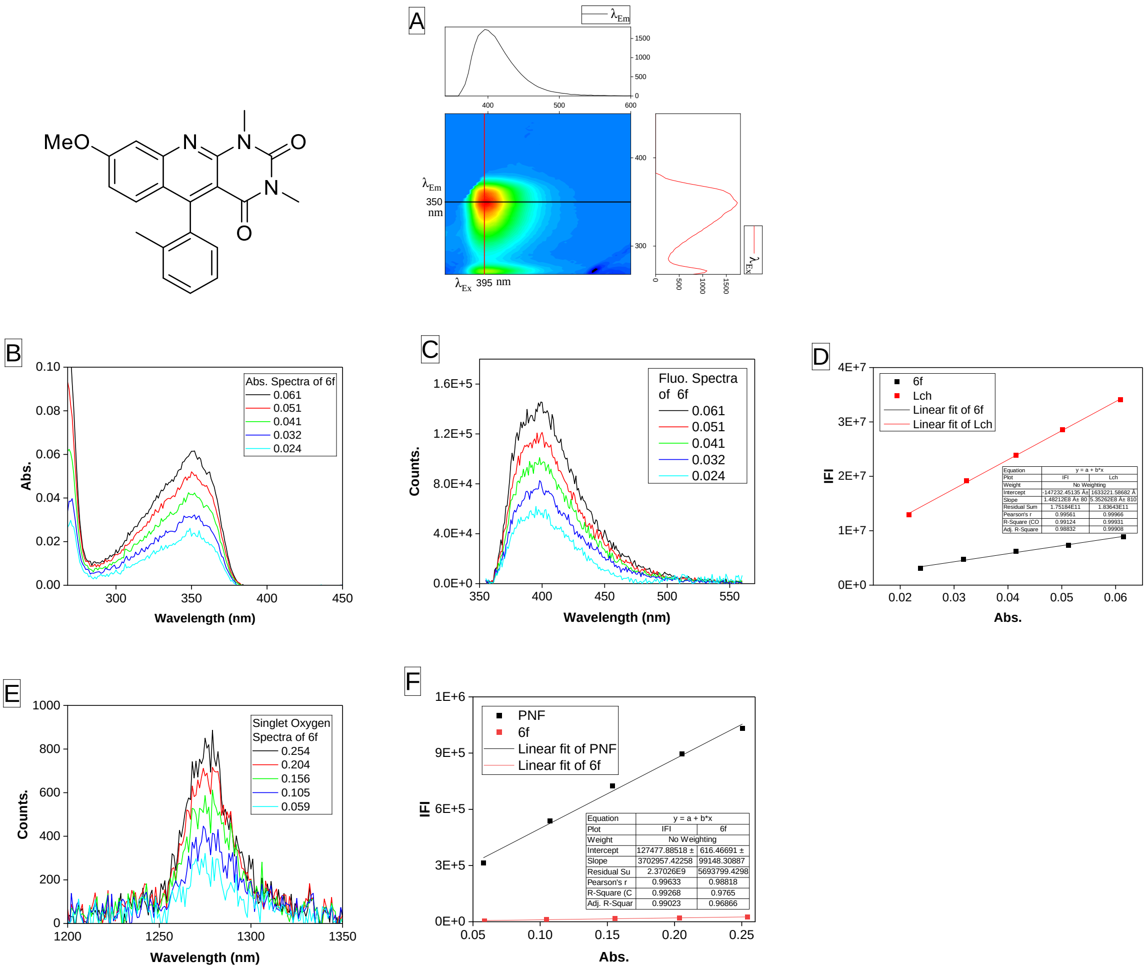

Figure S29: Photophysical data of a compound **3c(*o*-MePh)**. **Panel-[A]** represents a fully corrected emission excitation matrix in DMF. **[B]** Absorption spectra of **3c(*o*-MePh)** with 5 different concentrations and their **[C]** relative fluorescence spectra recorded using λ_{ex} = 351 nm. **[D]** Integrated fluorescence intensity versus absorption plot. **[E]** Phosphorescence spectra of singlet oxygen, samples excited at λ_{ex} = 365 nm and spectra collected in the NIR range between 1200 – 1350 nm. **[F]** Integrated phosphorescence intensity versus absorption plot.

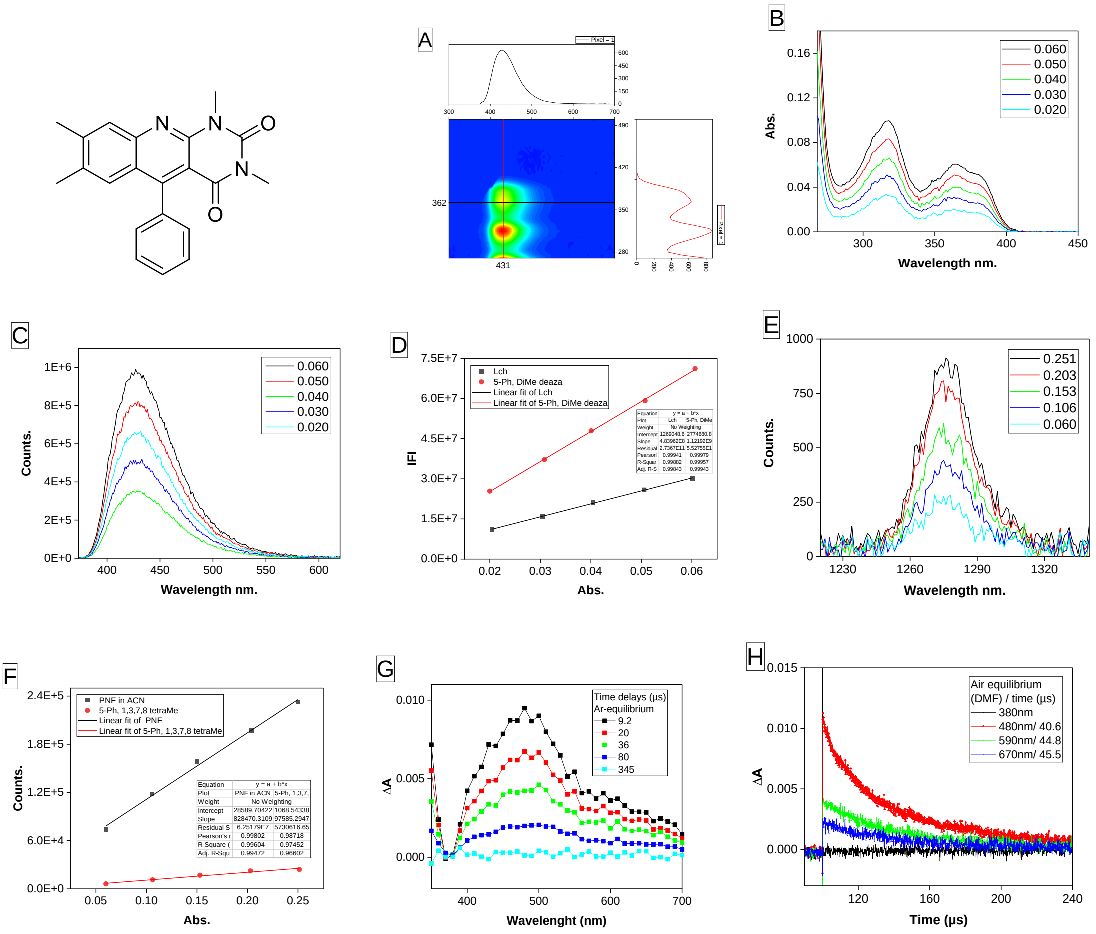

Figure S30: Photophysical data of a compound **3d(Ph)**. **Panel-[A]** represents a fully corrected emission excitation matrix in DMF. **[B]** Absorption spectra of **3d(Ph)** with 5 different concentrations and their **[C]** relative fluorescence spectra recorded using λ_{ex} = 363 nm. **[D]** Integrated fluorescence intensity versus absorption plot. **[E]** Phosphorescence spectra of singlet oxygen, samples excited at λ_{ex} = 382 nm and spectra collected in the NIR range between 1200 – 1350 nm. **[F]** Integrated phosphorescence intensity versus absorption plot. **[G]** Transient absorption spectra of **3d(Ph)** using a laser pulse at λ_{exc} = 365 nm and energy 0.5 mJ, collected between 350 -760 nm using a 10 nm interval, (Ar-equilibrated solution). **[H]** Transient absorption kinetics at selective wavelength (Ar-equilibrated solution).

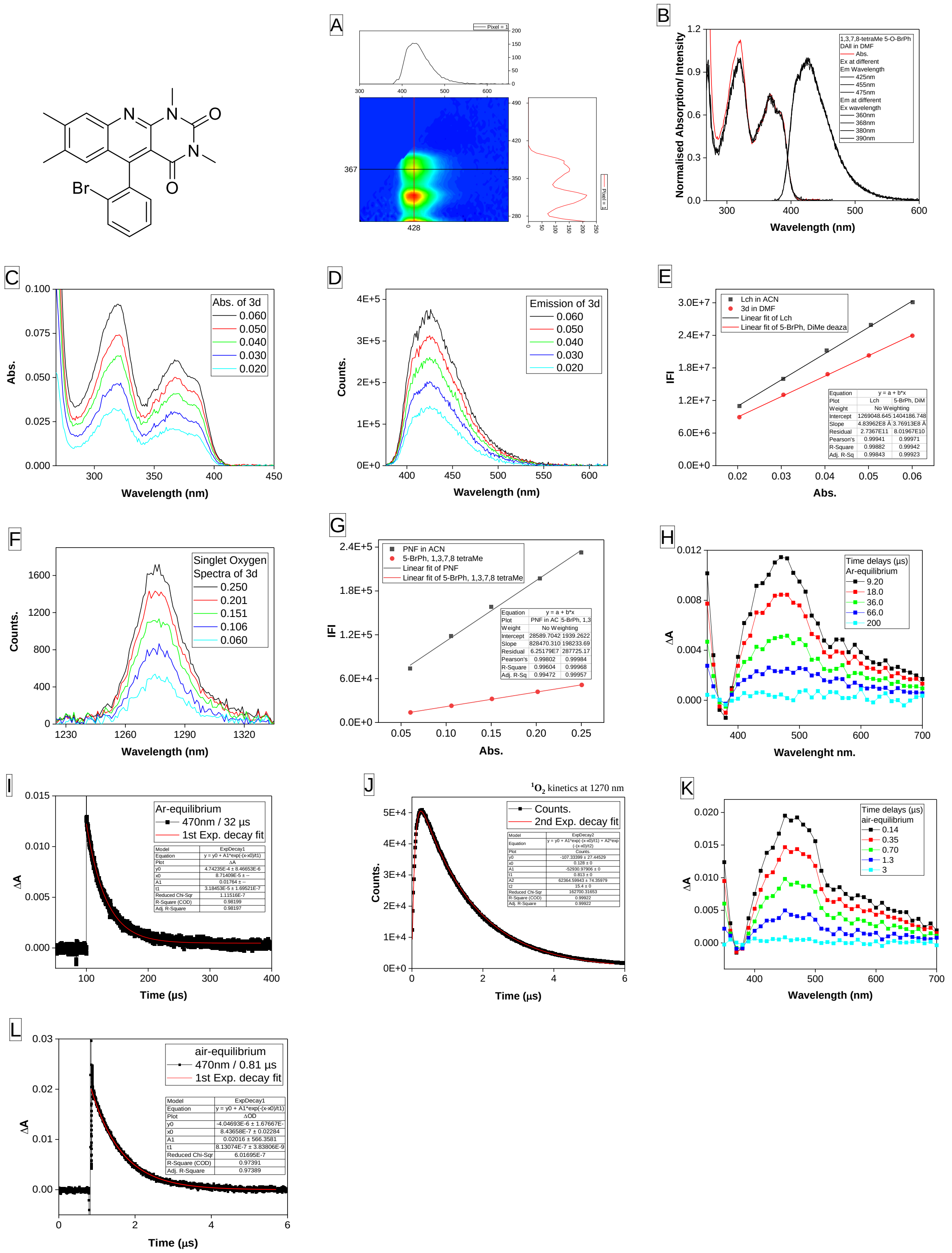

Figure S31: Photophysical data of a compound **3d(*o*-BrPh)**. **Panel-[A]** represents a fully corrected emission excitation matrix in DMF. **[B]** UV-Vis absorption spectrum in DMF, steady-state emission spectra recorded at varying excitation wavelengths, excitation spectra collected at selected emission wavelengths. **[C]** Absorption spectra of **3d(*o*-BrPh)** with 5 different concentrations and their **[D]** relative fluorescence spectra recorded using λ_{ex} = 367 nm. **[E]** Integrated fluorescence intensity versus

absorption plot. **[F]** Phosphorescence spectra of singlet oxygen, samples excited at λ_{ex} = 382 nm and spectra collected in NIR range between 1200 – 1350 nm. **[G]** Integrated phosphorescence intensity versus absorption plot. **[H]** Transient absorption spectra of **3d(*o*-BrPh)** using a laser pulse at λ_{exc} = 365 nm and energy 0.5 mJ, collected between 350 -700 nm using a 10 nm interval, (Ar-equilibrated solution) and **[I]** transient absorption kinetics (Black) and decay fit in red (Ar-equilibrated solution). **[J]** Phosphorescence ($^1O_2$) decay (black) and fitted curve (red). **[K]** Transient absorption spectra of **3d(*o*-BrPh)** using a laser pulse at λ_{exc} = 365 nm and energy 0.5 mJ, collected between 350 -700 nm using a 10 nm interval, (air-equilibrated solution). **[L]** Transient absorption kinetics (Black) and decay fit in red (air-equilibrated solution).

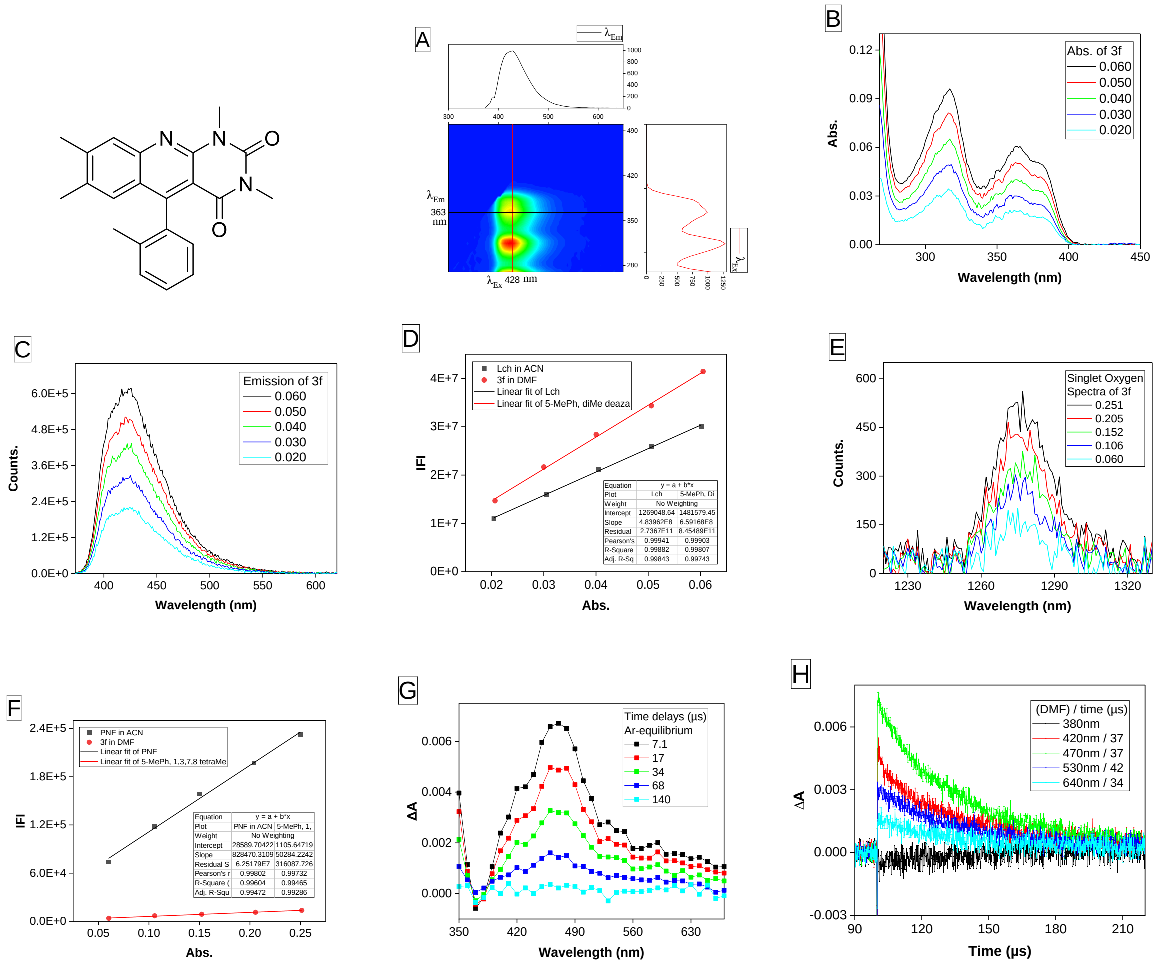

Figure S32: Photophysical data of a compound **3d(*o*-MePh)**. **Panel-[A]** represents a fully corrected emission excitation matrix in DMF. **[B]** Absorption spectra of **3d(*o*-MePh)** with 5 different concentrations and their **[C]** relative fluorescence spectra recorded using λ_{ex} = 363 nm. **[D]** Integrated fluorescence intensity versus absorption plot. **[E]** Phosphorescence spectra of singlet oxygen, samples excited at λ_{ex} = 382 nm and spectra collected in the NIR range between 1200 – 1350 nm. **[F]** Integrated phosphorescence intensity versus absorption plot. **[G]** Transient absorption spectra of **3d(*o*-MePh)** using a laser pulse at λ_{exc} = 365 nm and energy 0.5 mJ, collected between 350 -760 nm using a 10 nm interval, (Ar-equilibrated solution). **[H]** Transient absorption kinetics at selective wavelength (Ar-equilibrated solution).

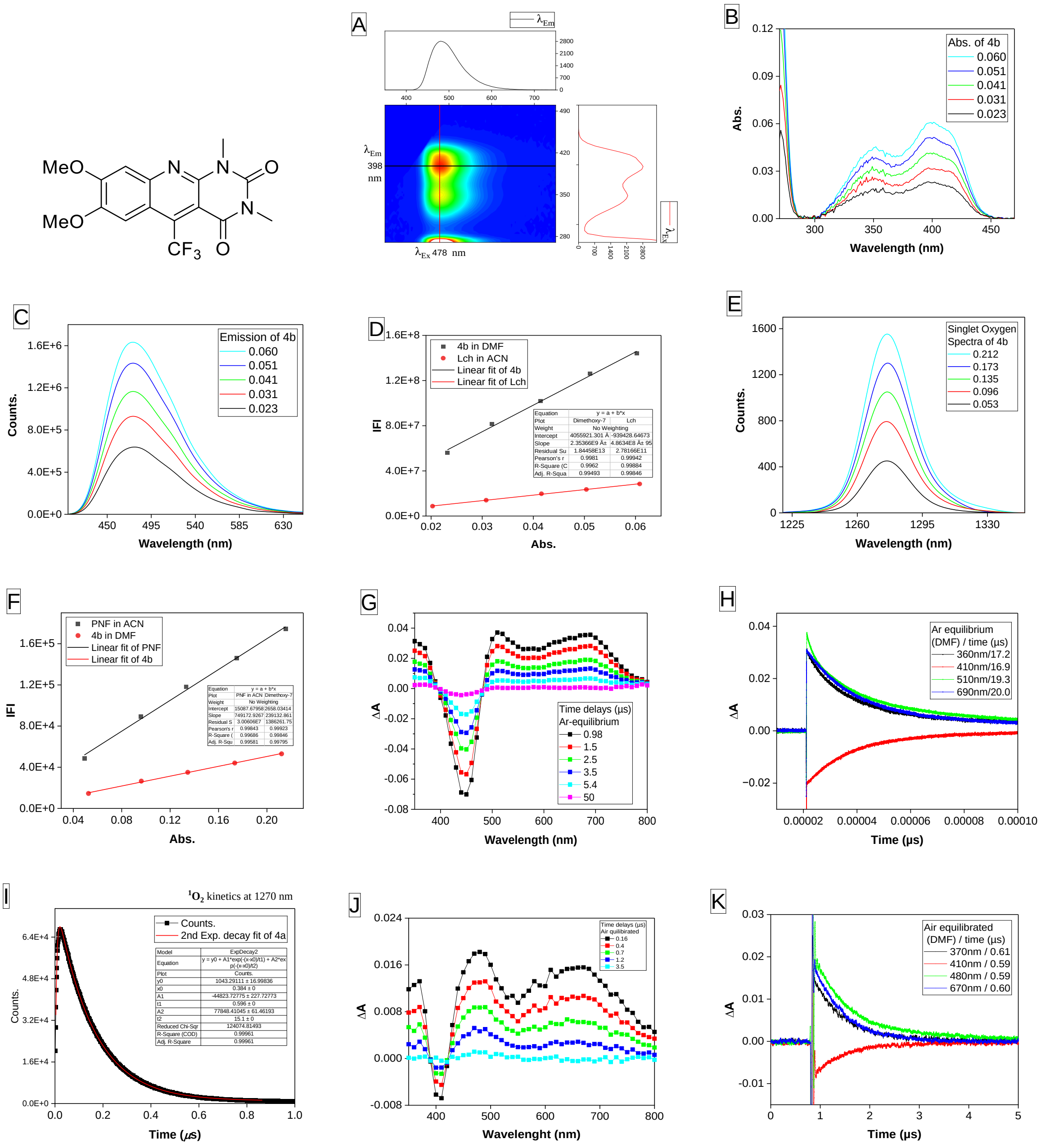

Figure S33: Photophysical data of a compound **4a**. **Panel-[A]** represents a fully corrected emission excitation matrix in DMF. **[B]** Absorption spectra of **4a** with 5 different concentrations and their **[C]** relative fluorescence spectra recorded using λ_{ex} = 398 nm. **[D]** Integrated fluorescence intensity versus absorption plot. **[E]** Phosphorescence spectra of singlet oxygen, samples excited at λ_{ex} = 399 nm and spectra collected in the NIR range between 1200 – 1350 nm. **[F]** Integrated phosphorescence intensity versus absorption plot. **[G]** Transient absorption spectra of **4a** using a laser pulse at λ_{exc} = 405 nm and energy 0.5 mJ, collected between 350 -800 nm using a 10 nm interval, (Ar-equilibrated solution) and **[H]** transient absorption kinetics at selective wavelength (Ar-equilibrated solution). **[I]** Phosphorescence ($^1O_2$) decay (black) and fitted curve (red). **[J]** Transient absorption spectra of **4a** using a laser pulse at λ_{exc} = 405 nm and energy 0.5 mJ, collected between 350 -800 nm using a 10 nm interval, (air-equilibrated solution). **[K]** Transient absorption kinetics at selective wavelength (air-equilibrated solution).

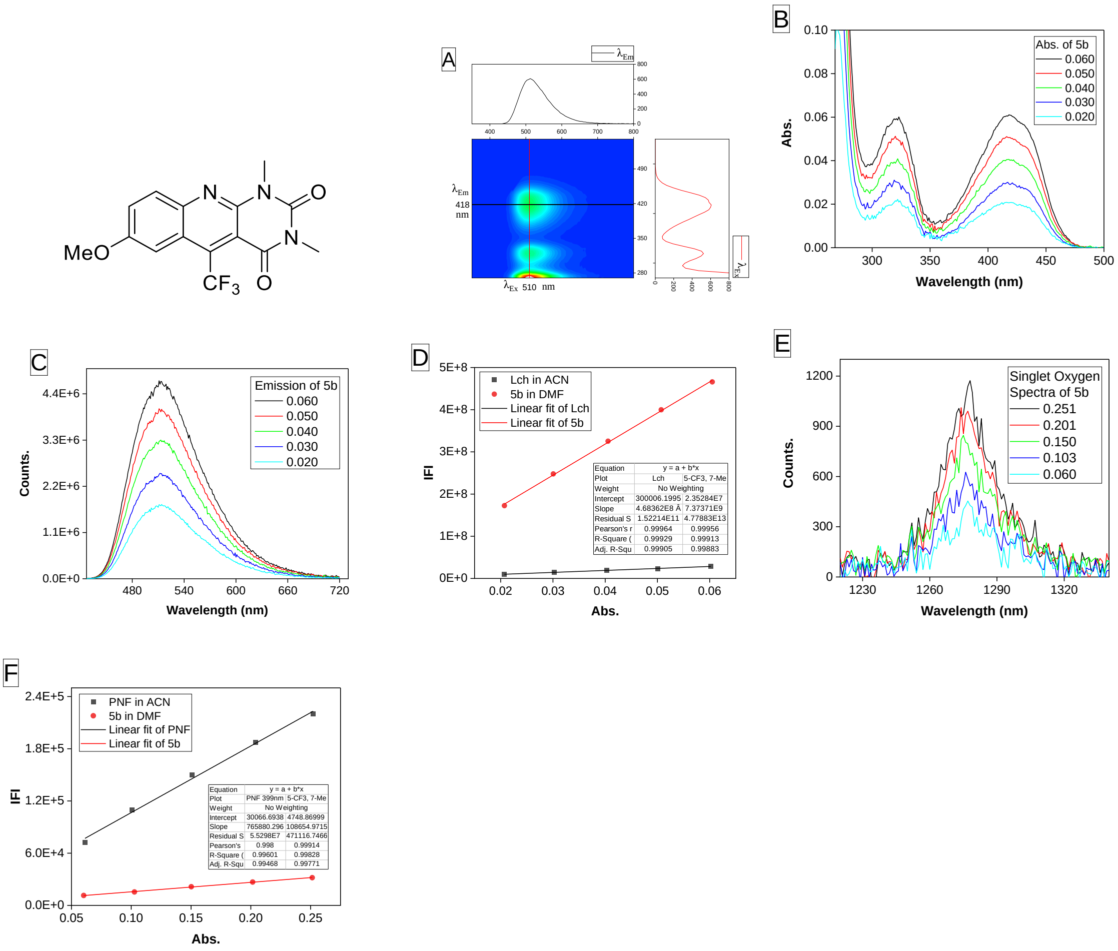

Figure S34: Photophysical data of a compound **4b**. **Panel-[A]** represents a fully corrected emission excitation matrix in DMF. **[B]** Absorption spectra of **4b** with 5 different concentrations and their **[C]** relative fluorescence spectra recorded using λ_{ex} = 417 nm. **[D]** Integrated fluorescence intensity versus absorption plot. **[E]** Phosphorescence spectra of singlet oxygen, samples excited at λ_{ex} = 399 nm and spectra collected in the NIR range between 1200 – 1350 nm. **[F]** Integrated phosphorescence intensity versus absorption plot.

5.2 Theoretical spectra

Table S3: The lowest predicted (B3LYP/aug-cc-pVTZ) $S_0 \rightarrow S_i$ excitation energies of deazaalloxazines with their corresponding oscillator strengths, *f*. No solvent included in the model.

	2a		3a(*o*-BrPh)		3a(*o*-MePh)		3b(*o*-MePh)		3b(thiophen-3-yl)		3d(*o*-MePh)	
$S_0 \rightarrow S_i$	$E\times 10^{-3}$ / cm^{-1}	*f*	$E\times 10^{-3}$ / cm^{-1}	*f*	$E\times 10^{-3}$ / cm^{-1}	*f*	$E\times 10^{-3}$ / cm^{-1}	*f*	$E\times 10^{-3}$ / cm^{-1}	*f*	$E\times 10^{-3}$ / cm^{-1}	*f*
$\rightarrow S_1$	29.0	0.059	28.4	0.121	28.4	<0.001	25.5	0.076	25.2	0.074	28.0[a]	0.001
$\rightarrow S_2$	31.9[a]	<0.001	29.3	0.002	28.7	0.117	27.1[a]	<0.001	26.8	0.008	28.3	0.069
$\rightarrow S_3$	33.5	0.113	31.4	0.152	31.4	0.106	30.4[a]	0.004	29.3	0.004	31.5[a]	0.020
$\rightarrow S_4$	36.9[a]	<0.001	32.3[a]	<0.001	32.2	0.047	32.6[a]	0.006	32.6	0.003	32.6	0.103
$\rightarrow S_5$	38.1	0.024	35.2[a]	<0.001	34.0	0.001	33.2	0.041	33.0	0.048	33.4	0.007
$\rightarrow S_6$	40.9[a]	0.0	37.9	0.124	38.0[a]	<0.001	36.9	0.072	36.6	0.070	37.5[a]	<0.001
$\rightarrow S_7$	41.2	0.333	38.4	0.010	38.2	0.137	37.1[a]	0.004	36.9[a]	0.001	38.2	0.087
$\rightarrow S_8$	41.4	0.478	38.5	0.038	38.8	0.062	39.1	0.398	38.4	0.166	38.8[a]	0.004
$\rightarrow S_9$	41.8[a]	0.0	39.3	0.054	39.0[a]	0.008	39.4	0.030	39.1	0.100	40.2	0.353
$\rightarrow S_{10}$	44.2	0.365	39.6	0.089	40.2	0.075	40.0	0.079	39.5	0.032	40.5	0.489
$\rightarrow S_{11}$	45.0	0.002	40.0	0.112	40.4	0.783	40.1[a]	0.005	40.1	0.004	41.4[a]	0.005
$\rightarrow S_{12}$	45.8	0.314	40.1	0.020	41.5	0.018	40.6	0.280	40.5	0.686	41.9[a]	0.008
$\rightarrow S_{13}$	46.7[a]	0.0	40.1	0.232	41.8	0.125	40.9[a]	0.001	40.6	0.003	42.0[a]	0.004
$\rightarrow S_{14}$	46.9[a]	0.0	40.8	0.007	41.9	0.045	41.1	0.349	40.9	0.222	42.3	0.021
$\rightarrow S_{15}$	47.4	0.070	41.1	0.399	42.1	0.045	41.6	0.097	41.8	0.035	42.7	0.198

a - n,π* state, otherwise π,π* state

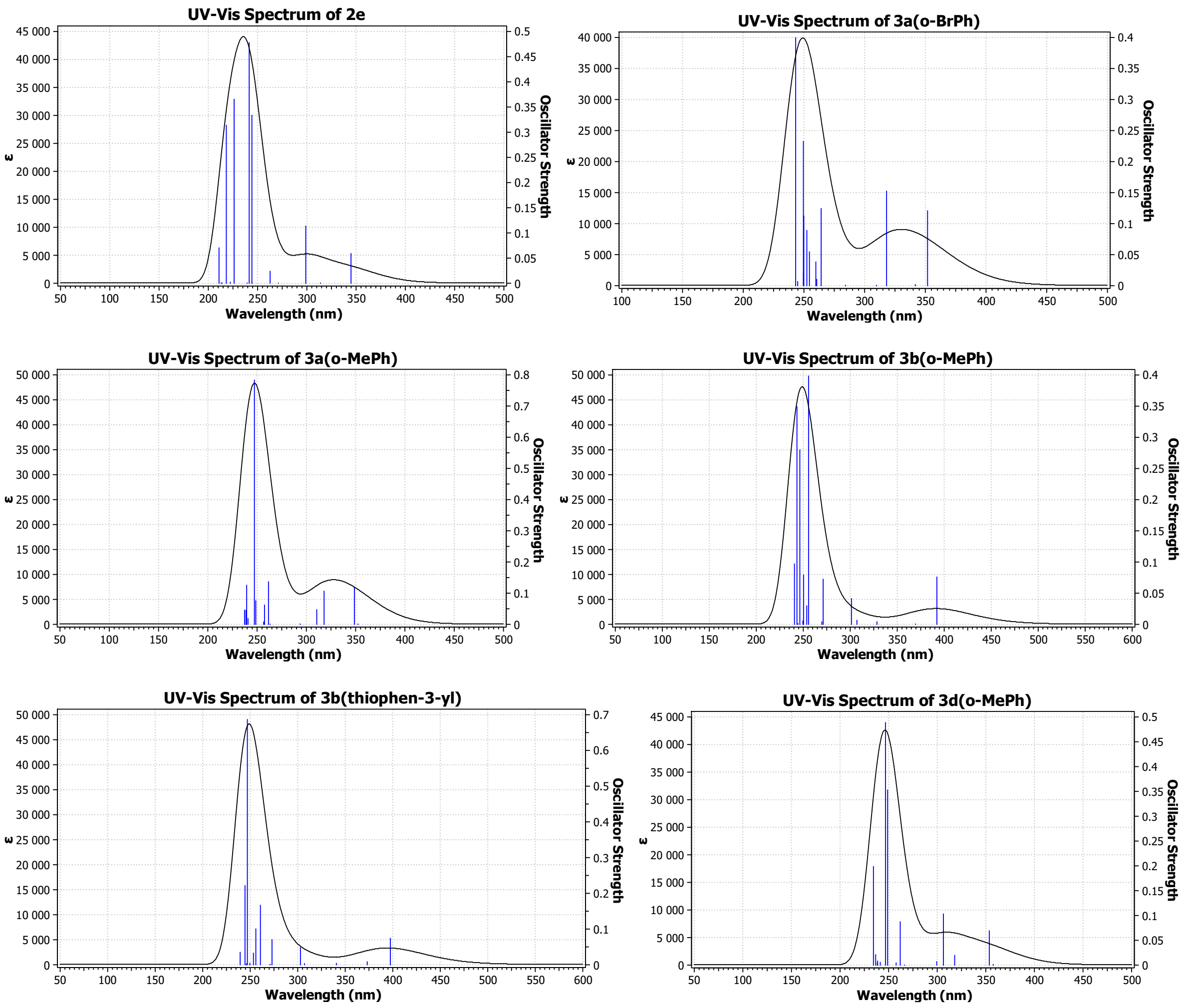

Figure S35: Theoretical absorption spectra for selected deazaalloxazines based on the lowest predicted (B3LYP/aug-cc-pVTZ) $S_0 \rightarrow S_i$ excitation energies with their corresponding oscillator strengths, f (for values, see Table S3)

**HOMO**	**->**	**LUMO - 0.67240**

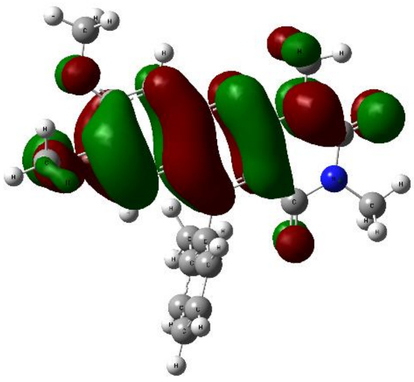

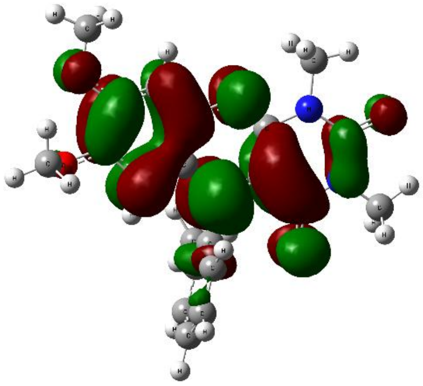

**HOMO-2**	**->**	**LUMO+1 - 0.15070**

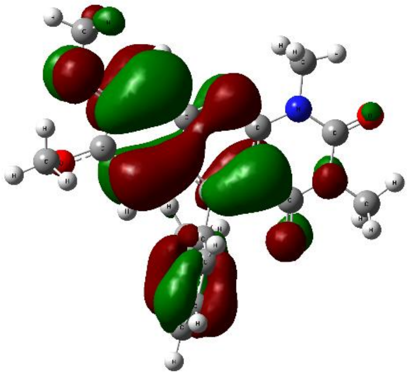

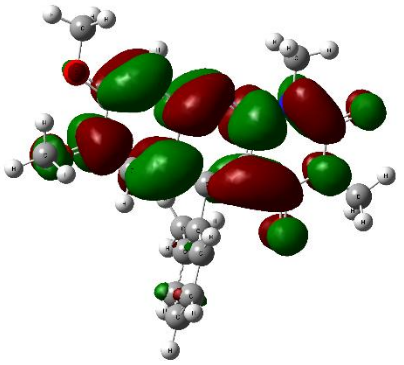

**HOMO-2**	**->**	**LUMO - -0.11650**

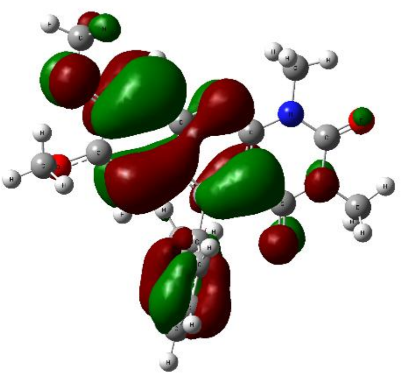

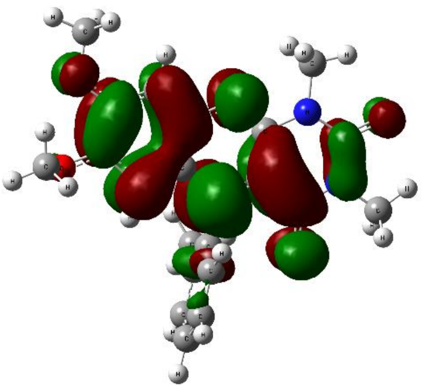

Figure S36. The shape of the molecular orbitals for the **3a(o-MePh)** molecule, involved in the S0→S2 transition. The isosurfaces correspond to the wave function value of ±0.02.

S6. ESTIMATION OF E^*_{OX} OF ANION RADICAL

E^*_{ox} of **3a(*o*-MePh)**$^{\bullet-}$ in an excited state was estimated from ground state redox potential ($E_{1/2}$ = -1.65 V vs SCE) and the value $E^{0\text{–}0}$ = 1.68 eV corresponding to photon energy of wavelength where absorption achieve 10 % of absorption maximum of the band with lower energy.[8]

$$E^*_{ox}[\mathbf{3a(}\boldsymbol{o}\mathbf{\text{-}MePh)}^{\bullet-}] = E_{1/2} - E^{0\text{–}0} = -1.65 - 1.68 = -3.33\ \text{V}$$

S7.EXPERIMENTAL SETUP FOR PHOTOCATALYTIC EXPERIMENTS

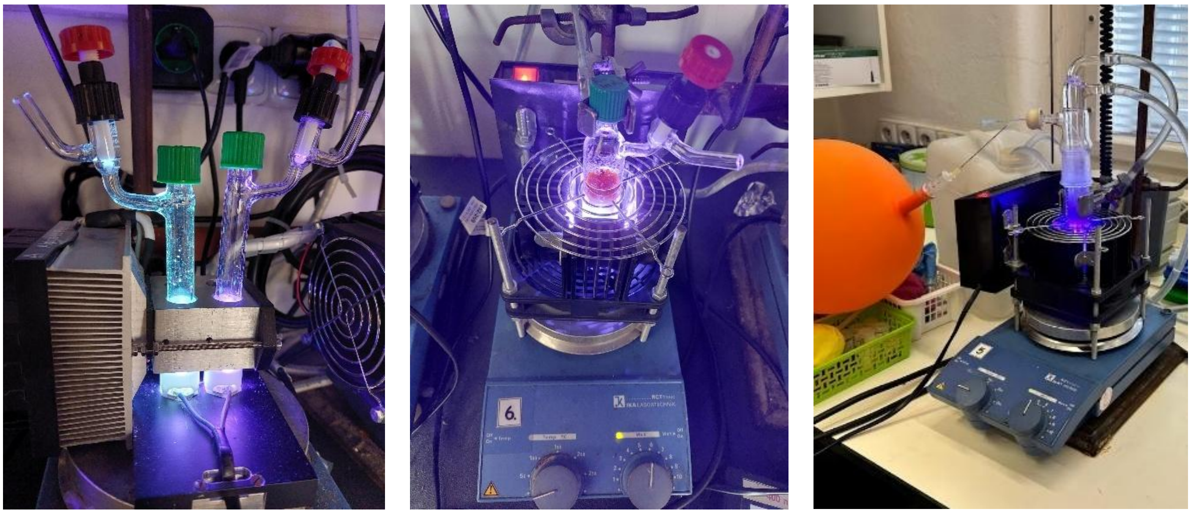

A **B** **C**

Figure S37: Experimental setup for photocatalytic reduction irradiation at analytical scale (A), preparative scale (B) and preparative scale with cooling (C).

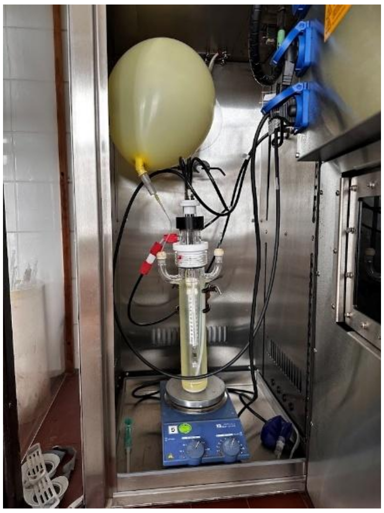

Figure S38: Experimental setup for photocatalytic reduction under preparative irradiation on the scale of 10 mmol.

S8. PHOTOSTABILITY OF DEAZAALLOXAZINES

8.1 Photostability of selected deazaalloxazines

5-Deazaalloxazine or deazaflavin (0.003 mmol) was dissolved in dry DMF-d_7 or CD_3CN (0.5 mL) and measured by ^{1}H NMR as a starting point. The mixture was then degassed using the freeze-pump-thaw technique (3 × 3 min, p = 4 mbar). The reaction mixture was irradiated at 400 nm (LED Engin, 1.35 W@700 mA) for 16 h at 50 °C with continuous stirring using the apparatus shown in **Figure S37** (see Section S7). The individual conversions were measured by ^{1}H NMR using DMF or ACN as internal standard.

8.2 Photocatalytic debromination of deazaalloxazine 3a(*o*-BrPh)

A mixture of 5-deazaalloxazine **3a(*o*-BrPh)** (0.003 mmol) and DIPEA (2 equiv.) was dissolved in dry DMF (0.5 mL) and then degassed by the freeze-pump-thaw technique (3 × 3 min, p = 4 mbar). The reaction mixture was irradiated at a wavelength of 400 nm (LED Engin, 1.35 W@700 mA) for 10-60 min at 25 °C with continuous stirring using the apparatus shown in **Figure S37** (see Section S7). Individual conversions were measured by ^{1}H NMR.

Table S4: Debromination conversion of **3a(*o*-BrPh)** in the presence of DIPEA performed in DMF-d_7

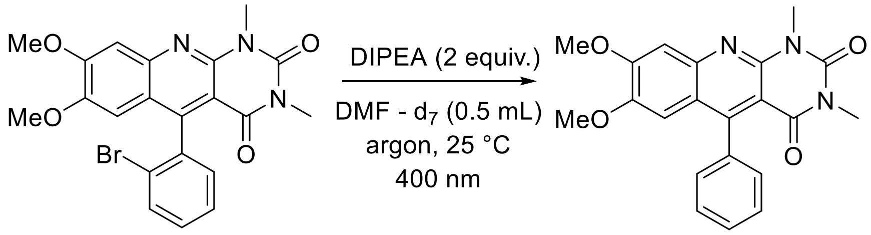

3a(*o*-BrPh) **3a(Ph)**

Time [min]	Conversion 3a(Ph) [a]
10	29 %
20	37 %
60	51 %

[a] 1H NMR conversion.

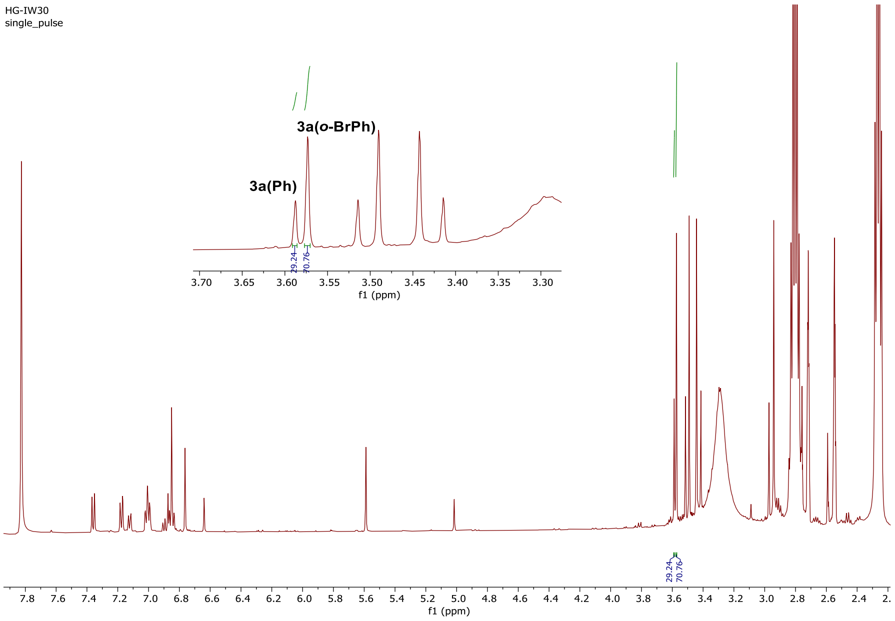

Figure S39: ^{1}H-NMR spectrum of debromination of **3a(*o*-BrPh)** in the presence of DIPEA performed in DMF-d_7 after 10 minutes of irradiation.

8.3 Investigation of stability of deazaalloxazine 3a(*o*-MePh) during reaction

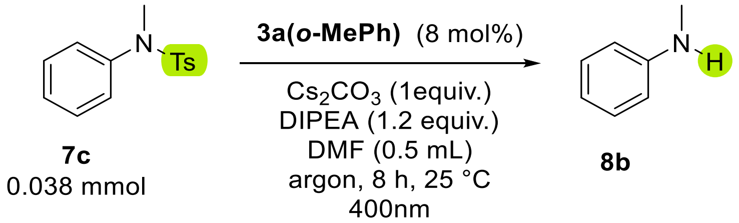

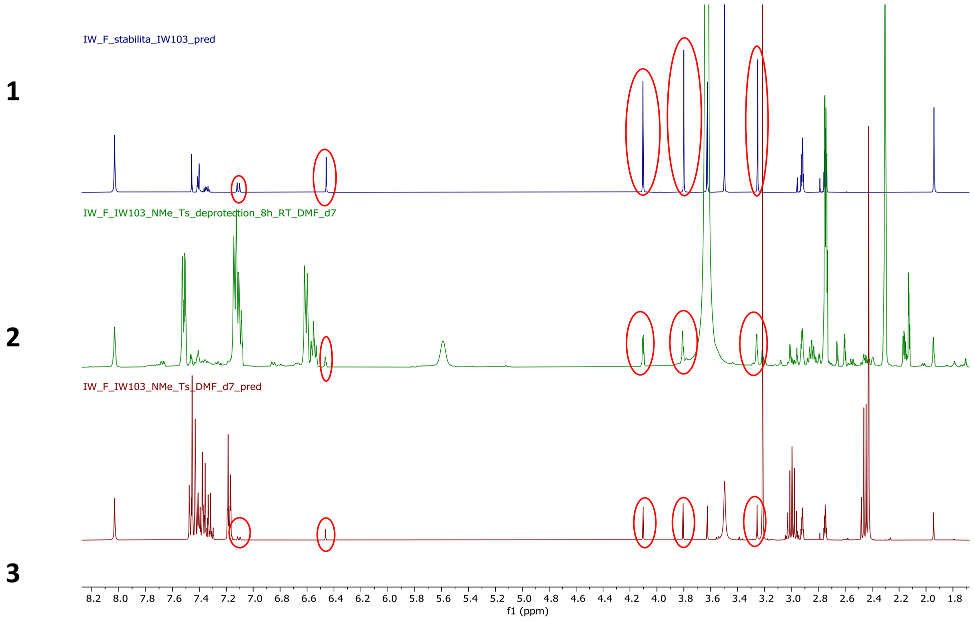

Figure S40: ^{1}H NMR spectra of deazaalloxazine **3a(*o*-MePh)** (1), in the mixture with the starting tosylamide **7c** before the start of irradiation (2), in the mixture with the product **8b** after irradiating the reaction mixture for 8 h (3). Measured in DMF-d_7.

S9. INITIAL SCREENING OF CATALYTIC ACTIVITY

9.1 Photocatalytic model reaction of deprotection or dehalogenation by deazaalloxazine catalysts

A mixture of *N*-tosylaniline (**7a**)/*N*-methylphenyltriflimide (**9a**)/*p*-bromanisole (**5a**) (0.038 mmol, 1 equiv.), deazaalloxazine or deazaflavin (8 mol%), Cs_2CO_3 (12.5 mg, 1 equiv.), and DIPEA (7.8 µL, 1.2 equiv.) were dissolved in dry DMF or ACN (0.5 mL) and then degassed by the freeze-pump-thaw technique (3 × 3 min, p = 4 mbar). The reaction mixture was irradiated at 365, 385, 400 or 450 nm for 4 h with constant stirring using the apparatus shown in **Figure S37** (see Section S7). The individual reaction conversions were measured by ^{1}H NMR.

Table S5: Summary of *N*-tosylaniline (**7a**) detosylation results for each of the deazaalloxazines

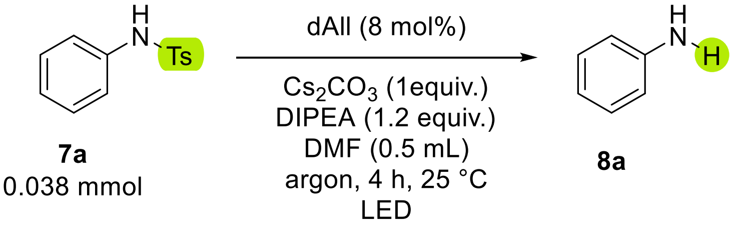

Entry	dAll	λ [nm]	Conversion [%][a]
1[b]	**1(*o*-MePh)**	400	17
2	**2a**	385	14
3	**2a-H**	385	17
		400	20
4	**2b-H**	400	30
5	**2c-H**	365	0
6	**2d-H**	385	27
7	**2e**	365	0
		385	0
8	**3a(Ph)**	400	73
9	**3a(*o*-BrPh)**	400	74
10	**3a(*o*-BrPh) -H**	400	63
11	**3a(*p*-BrPh)**	400	70
12	**3a(*o*-MePh)**	400	77
13	**3a(*o*-MePh)-H**	400	23
14	**3a(napht-1-yl)**	385	45
		400	49
15	**3a(pyridin-4-yl)**	385	43
		400	80
16	**3a(pyridin-2-yl)**	385	27

[a] ^{1}H NMR conversion. [b]Experiment in ACN.

Entry	dAll	λ [nm]	Conversion [%][a]
16	**3a(pyridin-2-yl)**	400	43
17	**3a(thiophen-3-yl)**	385	8
		400	78
18	**3b(Ph)**	400	67
19	**3b(*o*-BrPh)**	400	48
20	**3b(*o*-MePh)**	400	78
21	**3b(napht-1-yl)**	400	32
22	**3b(pyridin-4-yl)**	385	40
		400	42
23	**3b(pyridin-2-yl)**	400	15
24	**3b(thiophen-3-yl)**	400	38
25	**3c(Ph)**	365	0
26	**3c(*o*-BrPh)**	365	4
27	**3c(*o*-MePh)**	365	5
28	**3d(Ph)**	400	78
29	**3d(*o*-BrPh)**	400	65
30	**3d(*o*-MePh)**	400	86
31	**4a**	400	53
32	**4b**	400	49
		450	0

[a] ^{1}H NMR conversion.

Table S6: Summary of *N*-methylphenyltriflamide (**9a**) detriflylation results for each of the deazaalloxazines

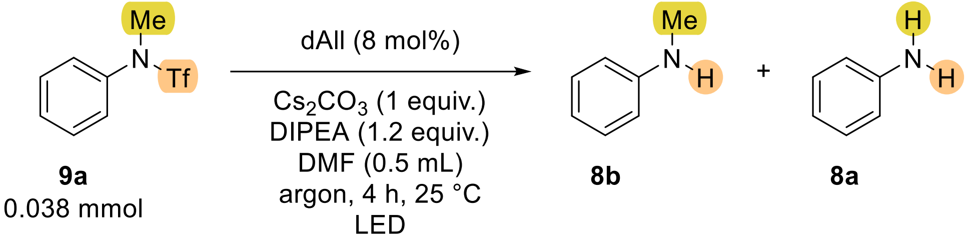

Entry	dAll	λ [nm]	Conversion 8b [%][a]
1[b]	**1(*o*-MePh)**	400	39
2	**2a**	385	90
3	**2a-H**	385	28
		400	40
4	**2b-H**	400	46
5	**2c-H**	365	0
6	**2d-H**	385	24
7	**2e**	365	2
		385	21
8	**3a(Ph)**	400	100
9	**3a(*o*-BrPh)**	400	100
10	**3a(*o*-BrPh) -H**	400	75
11	**3a(*p*-BrPh)**	400	100
12	**3a(*o*-MePh)**	400	74 + 24 **8a**
13	**3a(*o*-MePh)-H**	400	63
14	**3a(napht-1-yl)**	385	5
		400	68
15	**3a(pyridin-4-yl)**	385	39
		400	60
16	**3a(pyridin-2-yl)**	385	59
16	**3a(pyridin-2-yl)**	400	64
17	**3a(thiophen-3-yl)**	385	63
		400	79
18	**3b(Ph)**	400	74
19	**3b(*o*-BrPh)**	400	71
20	**3b(*o*-MePh)**	400	58 + 28 **8a**
21	**3b(napht-1-yl)**	400	52
22	**3b(pyridin-4-yl)**	385	20
		400	30
23	**3b(pyridin-2-yl)**	400	34
24	**3b(thiophen-3-yl)**	400	40
25	**3c(Ph)**	365	4
26	**3c(*o*-BrPh)**	365	26
27	**3c(*o*-MePh)**	365	10
28	**3d(Ph)**	400	97
29	**3d(*o*-BrPh)**	400	93
30	**3d(*o*-MePh)**	400	100
31	**4a**	400	47
32	**4b**	400	85
		450	5

[a] ^{1}H NMR conversion. [b]Experiment in ACN.

[a] ^{1}H NMR conversion.

Table S7: Summary of *p*-bromanisole (**5a**) dehalogenation results for each of the deazaalloxazines

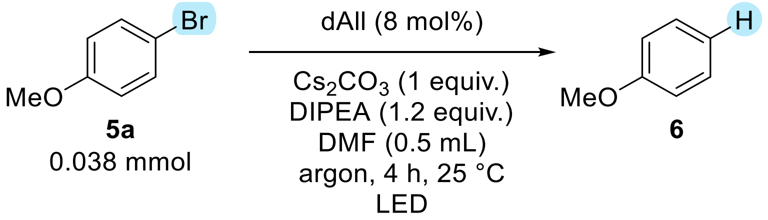

Entry	dAll	λ [nm]	Conversion [%][a]
1[b]	**1(*o*-MePh)**	400	49
2	**2a**	385	13
3	**2a-H**	385	15
		400	15
4	**2b-H**	400	20
5	**2c-H**	365	0
6	**2d-H**	385	32
7	**2e**	365	2
		385	21
8	**3a(Ph)**	400	28
9	**3a(*o*-BrPh)**	400	25
10	**3a(*o*-BrPh) -H**	400	21
11	**3a(*p*-BrPh)**	400	18
12	**3a(*o*-MePh)**	400	29
13	**3a(*o*-MePh)-H**	400	32
14	**3a(napht-1-yl)**	385	20
		400	18
15	**3a(pyridin-4-yl)**	385	18
		400	18
16	**3a(pyridin-2-yl)**	385	17

[a] ^{1}H NMR conversion. [b]Experiment in ACN.

Entry	dAll	λ [nm]	Conversion [%][a]
16	**3a(pyridin-2-yl)**	400	22
17	**3a(thiophen-3-yl)**	385	11
		400	13
18	**3b(Ph)**	400	23
19	**3b(*o*-BrPh)**	400	17
20	**3b(*o*-MePh)**	400	27
21	**3b(napht-1-yl)**	400	29
22	**3b(pyridin-4-yl)**	385	0
		400	21
23	**3b(pyridin-2-yl)**	400	12
24	**3b(thiophen-3-yl)**	400	21
25	**3c(Ph)**	365	0
26	**3c(*o*-BrPh)**	365	0
27	**3c(*o*-MePh)**	365	7
28	**3d(Ph)**	400	24
29	**3d(*o*-BrPh)**	400	15
30	**3d(*o*-MePh)**	400	28
31	**4a**	400	24
32	**4b**	400	19
16		450	0

[a] ^{1}H NMR conversion.

9.2 Photocatalytic reduction by selected deazaalloxazines

9.2.1 Photocatalytic dehalogenation *p*-fluoroanisole (5c) by selected deazaalloxazines

A mixture of *p*-fluoranisole (**5c**) (0.038 mmol, 1 equiv.), deazaalloxazine or deazaflavin (8 mol%), Cs_2CO_3 (12.5 mg, 1 equiv.), and DIPEA (7.8 µL, 1.2 equiv.) was dissolved in dry DMF or ACN (0.5 mL) and subsequently degassed by the freeze-pump-thaw technique (3 × 3 min, p = 4 mbar). The reaction mixture was irradiated at a wavelength of 400 nm (LED Engin, 1.35 W@700 mA) for 24 to 48 h with continuous stirring using the apparatus shown in **Figure S37** (see Section S7). Individual conversions were measured by ^1H NMR.

S10. REDUCTIVE DESULFONYLATIONS

10.1 Initial screening on analytical scale

Analytic experiments

A mixture of sulfonamide (0.038 mmol, 1 equiv.), 5-aryldeazaalloxazine **3a(*o*-MePh)** (8 mol%), Cs_2CO_3 (12.5 mg, 1 equiv.), and DIPEA (7.8 µL, 1.2 equiv.) was dissolved in dry DMF (0.5 mL) and subsequently degassed by the freeze-pump-thaw technique (3 × 3 min, p = 4 mbar). The reaction mixture was irradiated at a wavelength of 400 nm (LED Engin, 1.35 W@700 mA) for 2-48 h with continuous stirring using the equipment shown in **Figure S37** (see Section S7). The reaction was monitored by periodic recording of ^{1}H NMR spectra.

Table S8: Conversions of individual trifyl/tosyl substrates performed at analytical scale with **3a(*o*-MePh)** catalysis compared to previous **3a(*o*-BrPh)** results

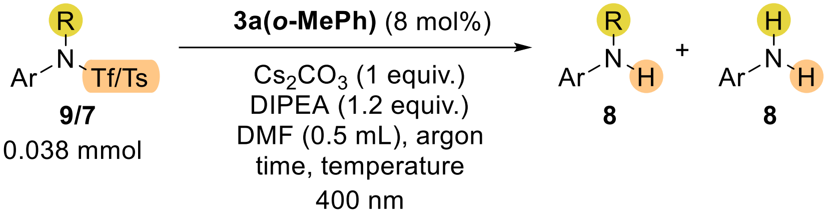

Entry	Substrate	Conversion with 3a(*o*-MePh)	Conversion with 3a(*o*-BrPh)
1	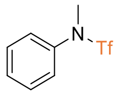 **9a**	**2 h, 25 °C:**<br>▪ 90 % NHMe<br>**4 h, 50 °C**<br>▪ 73 % NHMe<br>▪ 27 % NH_2<br>**8 h, 25 °C**<br>▪ 70 % NHMe<br>▪ 30 % NH_2	**4h, 25 °C:**<br>▪ 92 % NHMe<br>**4 h, 50 °C:**<br>▪ 100 % NHMe<br>**16 h, 50 °C:**<br>▪ 65 % NHMe<br>▪ 35 % NH_2
2	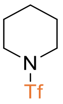 **9b**	**16 h, 50 °C:**<br>▪ 15 %<br>**24 h, 50 °C:**<br>▪ 50 %<br>**48 h, 50 °C:**<br>▪ 52 %	**16 h, 50 °C:**<br>▪ 0 %
3	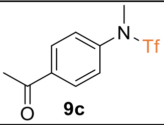 **9c**	**16 h, 50 °C**<br>▪ 76 % NHMe<br>▪ 24 % NH_2	**16 h, 50 °C:**<br>▪ 74 % NHMe<br>▪ 26 % NH_2
4	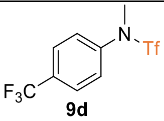 **9d**	**16 h, 50 °C:**<br>▪ 80 % NHMe<br>▪ 20 % NH_2	**16 h, 50 °C:**<br>▪ 80 % NHMe<br>▪ 20 % NH_2
5	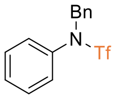 **9e**	**16 h, 50 °C**<br>▪ 100 % NHBn	**16 h, 50 °C:**<br>▪ 100 % NHBn
6	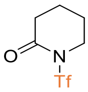 **9f**	**4 h, 50 °C:**<br>▪ Ring opening	-
7	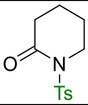 **7b**	**4 h, 25 °C:**<br>▪ 100%	-

[a] ^{1}H NMR conversion.

10.2 Reactions on preparative scale

General procedure D

A mixture of sulfonamide (1 mmol, 1 equiv.), 5-aryldeazaalloxazine **3a(*o*-MePh)** (8 mol%), Cs_2CO_3 (0.25 g, 1 equiv.), and DIPEA (208 µL, 1.2 equiv.) was dissolved in dry DMF (13 mL) and then degassed by the freeze-pump-thaw technique (3 × 3 min, p = 4 mbar). The reaction mixture was irradiated at a wavelength of 400 nm (8 × LED Engin, 1.35 W@700 mA) until complete conversion (monitored by ^{1}H NMR) with constant stirring using the apparatus shown in **Figure S37** (see Section S7). After irradiation, the reaction mixture was filtered and the DMF was evaporated. The crude product was purified by mobile phase column chromatography with a gradient of hexane → hexane:EtOAc (3:2) to give the corresponding amine.

*Aniline (**8a**)*

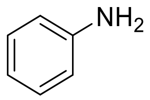

Prepared according to a **general procedure D** from triflamide **9a** (0.239 g, 1.0 mmol).
Reaction time: 16 hours.
Yield: 50 % (0.047 g) brown oil.

Prepared according to a **general procedure D** from triflamide **9e** (0.315 g, 1.0 mmol).
Reaction time: 48 hours.
Yield: 39 % (0.037 g) brown oil.

^{1}H NMR (400 MHz, DMSO-d_6) δ 7.02 – 6.94 (m, 2H) 6.56 – 6.49 (m, 2H), 6.45 (tt, *J* = 7.3, 1.2 Hz, 1H), 4.97 (br, s, 2H).
^{13}C NMR (101 MHz, DMSO-d_6) δ 149.1, 129.4, 116.2, 116.2, 114.4, 114.4.
HR-MS (ESI+) *m/z:* calculated for C_6H_8N [M+H$^+$]: 94.0653, **observed**: 94.0651.

Spectral data are in agreement with previously reported characterization data for the title compound[9].

*N-Methylaniline (**8b**)*

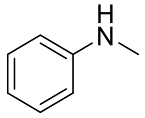

Prepared according to a **general procedure D** from triflamide **9a** (0.239 g, 1.0 mmol).
Reaction time: 3 hours.
Yield: 60 % (0.064 g) brown oil.

^{1}H NMR (400 MHz, DMSO-d_6) δ 7.09 – 7.00 (m, 2H), 6.52 – 6.46 (m, 3H), 5.56 (d, *J* = 5.2 Hz, 1H), 2.63 (d, *J* = 5.1 Hz, 3H).
^{13}C NMR (101 MHz, DMSO-d_6) δ 150.4, 129.4, 115.9, 112.1, 30.2.
HR-MS (ESI+) *m/z:* calculated for $C_7H_{10}N$ [M+H$^+$]: 108.0808, **observed**: 108.0809.

Spectral data are in agreement with previously reported characterization data for the title compound[9].

*1-(4-Aminophenyl)ethan-1-one (**8c**)*

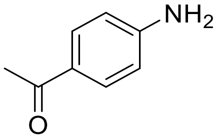

Prepared according to a **general procedure D** from triflamide **9c** (0.281 g, 1.0 mmol).
Reaction time: 48 hours.
Yield: 59 % (0.080 g) pale yellow solid.

^{1}H NMR (400 MHz, DMSO-d_6) δ 7.62 (d, *J* = 8.7 Hz, 2H), 6.61 (s, 2H), 6.52 (d, *J* = 8.7 Hz, 2H), 2.34 (s, 3H).
^{13}C NMR (101 MHz, DMSO-d_6) δ 195.5, 131.4, 131.1, 125.4, 113.3, 113.0, 112.7, 26.4.
HR-MS (ESI+) *m/z:* calculated for $C_8H_{10}NO$ [M+H$^+$]: 136.0755, **observed**: 136.0757.

Spectral data are in agreement with previously reported characterization data for the title compound[10].

*4-(Trifluoromethyl)aniline (**8e**)*

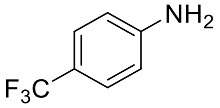

Prepared according to a **general procedure D** from triflamide **9d** (0.307 g, 1.0 mmol).
Reaction time: 48 hours.
Yield: 52 % (0.084 g) yellow oil.

^{1}H NMR (400 MHz, $CDCl_3$) δ 7.41 (d, *J* = 8.3 Hz, 2H), 6.68 (d, *J* = 8.4 Hz, 2H), 3.95 (s, 2H).
^{13}C NMR (101 MHz, $CDCl_3$) δ 149.6, 126.8 (q, *J* = 4.0 Hz), 124.9 (q, *J* = 271.7 Hz), 120.1 (q, *J* = 32.3 Hz), 114.3.
^{19}F NMR (376 MHz, $CDCl_3$) δ -61.1.
HR-MS (ESI+) *m/z:* calculated for $C_7H_7F_3N$ [M+H$^+$]: 162.0525, **observed**: 162.0525.

Spectral data are in agreement with previously reported characterization data for the title compound[11].

*N-Benzylaniline (**8f**)*

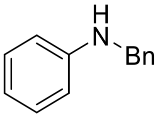

Prepared according to a **general procedure D** from triflamide **9e** (0.315 g, 1.0 mmol).
Reaction time: 48 hours.
Yield: 31 % (0.056 g) brown oil.

^{1}H NMR (400 MHz, $CDCl_3$) δ 7.36 – 7.26 (m, 4H), 7.22 – 7.19 (m, 1H), 7.03 – 6.94 (m, 2H), 6.59 – 6.50 (m, 2H), 6.48 (tt, *J* = 7.3, 1.1 Hz, 1H), 6.20 (t, *J* = 6.1 Hz, 1H), 4.23 (d, *J* = 6.1 Hz, 2H).
^{13}C NMR (101 MHz, DMSO-d_6) δ 149.2, 140.8, 129.3, 128.8, 127.7, 127.1, 116.2, 112.8, 46.9.
HR-MS (ESI+) *m/z:* calculated for $C_{13}H_{14}N$ [M+H$^+$]: 184.1118, **observed**: 184.1121.

Spectral data are in agreement with previously reported characterization data for the title compound[12].

*Piperidin-2-one (**11**)*

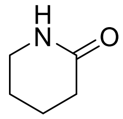

Prepared according to a **general procedure D** from tosylamide **7b** (0.253 g, 1.0 mmol).
Reaction time: 4 hours.
Purification: column chromatography in mobile phase with gradient CH_2Cl_2 → CH_2Cl_2:MeOH (10:1).
Yield: 53 % (0.052 g) pale brown solid.

^{1}H NMR (400 MHz, DMSO-d_6) δ 7.41 (s, 1H), 3.15 – 3.05 (m, 2H), 2.10 (dd, *J* = 6.9, 6.1 Hz, 2H), 1.71 – 1.55 (m, 4H).
^{13}C NMR (101 MHz, DMSO-d_6) δ 170.1, 41.3, 31.5, 22.1, 20.8.
HR-MS (ESI+) *m/z:* calculated for C_5H_9NO [M+H$^+$]: 100.0762, **observed**: 100.0757.

Spectral data are in agreement with previously reported characterization data for the title compound[13].

S11. PHOTOCATALYTIC COUPLING REACTION WITH P(OME)$_3$

11.1 Initial screening on analytical scale

Analytic experiments

A mixture of aryl halide **5** (0.038 mmol, 1 equiv.), 5-aryldeazaalloxazine **3a(*o*-MePh)** (8 mol%), Cs_2CO_3 (12.5 mg, 1 equiv.), DIPEA (7.8 µL, 1.2 equiv.), and P(OMe)$_3$ (5-20 equiv.) were dissolved in dry DMF (0.5 mL) and then degassed by the freeze-pump-thaw technique (3 × 3 min, p = 4 mbar). The reaction mixture was irradiated at a wavelength of 400 nm (LED Engin, 1.35 W@700 mA) for 16-72 h with continuous stirring using the equipment shown in **Figure S37** (see Section S7). The reaction was monitored by periodic recording of ^{1}H NMR spectra.

Table S9: Summary of the results of the coupling of $P(OMe)_3$ with various aryl halides carried out at analytical scale

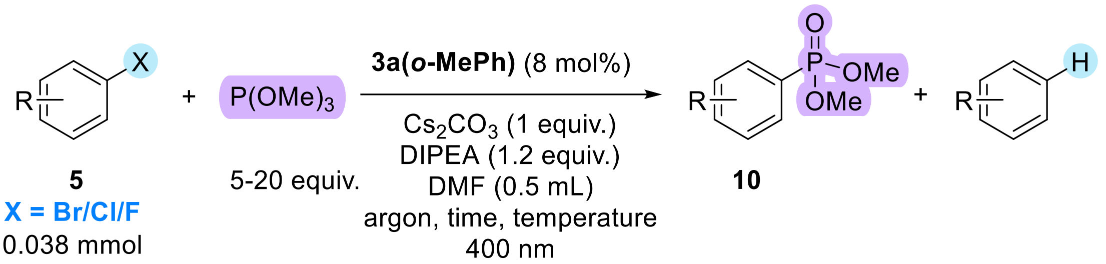

Entry	Substrate	Time	Temperature	Conversion [%][a] 5 equiv.			Conversion [%][a] 20 equiv.		
				Coup.	Deh.	Start.	Coup.	Deh.	Start.
1	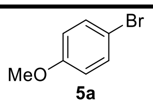 **5a**	16 h	50 °C	70	30		72	28	
2	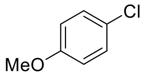 **5b**	16 h	50 °C	47	19	34	50	-	50
3		32 h	50 °C	-	-	-	68	9	23
4		48 h	50 °C	-	-	-	69	14	17
5	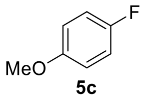 **5c**	16 h	50 °C	29	16	55	-	-	-
6		24 h	50 °C	33	21	46	-	-	-
7		48 h	50 °C	35	19	46	33	3	64
8		72 h	50 °C	-	-	-	34	10	56
9	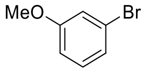 **5d**	16 h	50 °C	66	25	9	63	5	32
10	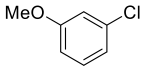 **5e**	16 h	50 °C	26	10	64	50	6	44
11		32 h	50 °C	-	-	-	51	6	43
12		48 h	50 °C	-	-	-	60	7	33
13	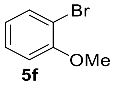 **5f**	16 h	50 °C	52	48		72	28	
14	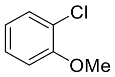 **5g**	16 h	50 °C	22	49	29	34	11	55
15		24 h	25 °C	24	40	22	-	-	-
16		32 h	50 °C	-	-	-	60	20	20
17		48 h	50 °C	-	-	-	57	21	22
18[b]	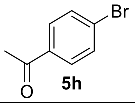 **5h**	16 h	50 °C	60	39	1	51	44	5
19[b]		16 h	25 °C	61	35	4	-	-	-
20[b]	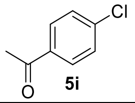 **5i**	16 h	50 °C	17	76	6	29	60	11
21[b]		24 h	25 °C	-	-	-	60	6	35
22	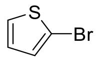 **5j**	16 h	50 °C	36	34	40	62	14	24
23		24 h	25 °C	62	38		62	38	
24	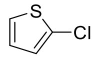 **5k**	16 h	50 °C	43	22	35	55	10	35
25		32 h	50 °C	-	-	-	65	13	22
26		48 h	50 °C	-	-	-	64	14	22

[a] ^1H NMR conversion. [b]GC-MS conversion. Coup. = product of coupling, Deh. = product of dehalogenation, Start. = starting compound

Table S10: Overview of the results of the dependence of dehalogenation/coupling of *p*-bromanisole (**5a**) on temperature and number of equivalents of $P(OMe)_3$

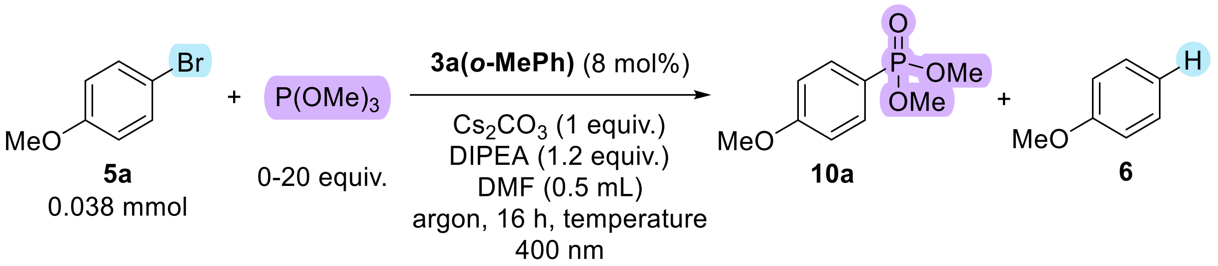

Equiv. $P(OMe)_3$	Conversion 5 °C [%][a]			Coup./ Deh.	Conversion 25 °C [%][a]			Coup./ Deh.	Conversion 50 °C [%][a]			Coup./ Deh.
	Coup.	Deh.	Start.		Coup.	Deh.	Start.		Coup.	Deh.	Start.	
0	-	45	55	-	-	46	54	-	-	57	43	-
5	44	15	41	2.9	54	18	28	3.0	70	30	-	2.3
20	55	8	37	6.9	69	12	19	5.8	72	28	-	2.6

[a] ^{1}H NMR conversion. Coup. = product of coupling, Deh. = product of dehalogenation, Start. = starting compound

Table S11: Decomposition of coupling product **10a** to form anisole (**6**) carried out under standard reaction conditions.

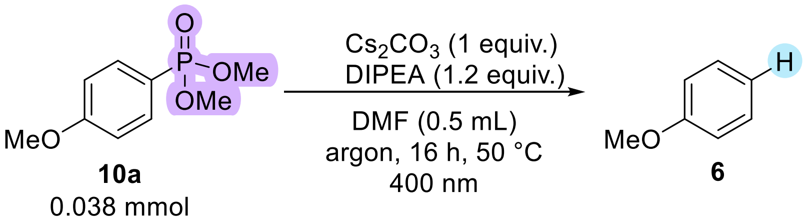

Catalyst	Conversion [%][a]
3a(*o*-MePh)	3
-	0

[a] ^{1}H NMR conversion.

11.2 Preparative experiments

General procedure F

A mixture of aryl halide **5** (1 mmol, 1 equiv.), 5-aryldeazaalloxazine **3a(*o*-MePh)** (8 mol%), Cs_2CO_3 (0.328 g, 1 equiv.), DIPEA (208 µL, 1.2 equiv.) and $P(OMe)_3$ (2.4 mL, 20 equiv.) were dissolved in dry DMF (13 mL) in a Schlenk flask and then degassed using the freeze-pump-thaw technique (3 × 3 min, p = 4 mbar). The reaction mixture was irradiated at 400 nm (8 ×

LED Engin, 1.35 W@700 mA) for 16-48 h (conversion monitored by ^{1}H NMR) with constant stirring using the apparatus shown in **Figure S37** (see Section S7). After irradiation, the reaction mixture was filtered and the DMF was evaporated. The crude product was purified by flash column chromatography with mobile phase with a hexane-acetone gradient (20-60 %) to give the corresponding product.

General procedure G: cooling system

A mixture of aryl halide **5** (1 mmol, 1 equiv.), 5-aryldeazaalloxazine **3a(*o*-MePh)** (8 mol%), Cs_2CO_3 (0.328 g, 1 equiv.), DIPEA (208 µL, 1.2 equiv.) and $P(OMe)_3$ (2.4 mL, 20 equiv.) was dissolved in dry DMF (13 mL) in a sublimator and then the mixture was bubbled with argon. The reaction mixture was irradiated at 400 nm (8 × LED Engin, 1.35 W@700 mA) for 16-24 h (conversion monitored by ^{1}H NMR) with constant stirring using the apparatus shown in **Figure S37** (see Section S7). After irradiation, the reaction mixture was filtered and the DMF was evaporated. The crude product was purified by flash column chromatography with mobile phase with a hexane-acetone gradient (20-60 %) to give the corresponding product.

General procedure H

A mixture of *p*-bromanisole (**5a**) (10 mmol, 1 equiv.), 5-aryldeazaalloxazine **3a(*o*-MePh)** (8 mol%), Cs_2CO_3 (3.28 g, 1 equiv.), DIPEA (2.08 mL, 1.2 equiv.), and $P(OMe)_3$ (24 mL, 20 equiv.) was dissolved in dry DMF (130 mL) and then the mixture was bubbled with argon. The reaction mixture was irradiated at 405 nm (novaLIGHT T-LED100 W) for 32 h (conversion monitored by ^{1}H NMR) with constant stirring using the apparatus shown in **Figure S38** (see Section S7). After irradiation, the reaction mixture was filtered and the DMF was evaporated. The crude product was purified by flash column chromatography with mobile phase with a hexane-acetone gradient (20-60 %) to give the corresponding product.

*Dimethyl (4-methoxyphenyl)phosphonate (**10a**)*

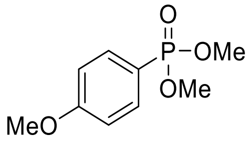

Prepared according to a **general procedure F** from 4-bromoanisole (**5a**) (0.187 g, 1.0 mmol).
Reaction time: 16 hours.
Yield: 79 % (0.171 g) yellow oil.

Prepared according to a **general procedure F** from 4-chloroanisole (**5b**) (0.142 g, 1.0 mmol).
Reaction time: 48 hours.
Yield: 52 % (0.121 g) yellow oil.

Prepared according to a **general procedure F** from 4-fluoroanisole (**5c**) (0.126 g, 1.0 mmol).
Reaction time: 48 hours.
Yield: 26 % (0.056 g) yellow oil.

Prepared according to a **general procedure H** from 4-bromoanisole (**5a**) (1.870 g, 10.0 mmol).
Reaction time: 32 hours.
Yield: 80 % (1.730 g) yellow oil.

^{1}H NMR (400 MHz, DMSO-*d*$_6$) δ 7.69 – 7.58 (m, 2H), 7.15 – 7.03 (m, 2H), 3.82 (s, 3H), 3.63 (s, 3H), 3.60 (s, 3H).
^{13}C NMR (101 MHz, DMSO-*d*$_6$) δ 162.6 (d, *J* = 3.4 Hz), 133.5 (d, *J* = 11,3 Hz; 2C), 118.0 (d, *J* = 193.3 Hz), 114.3 (d, *J* = 15.8 Hz, 2C), 55.4, 52.3 (d, *J* = 5.5 Hz, 2C).
HR-MS (ESI+) *m/z:* calculated for $C_9H_{13}O_4P$ [M+H$^+$]: 217.0629, **observed**: 217.0624.

Spectral data are in agreement with previously reported characterization data for the title compound[14].

*Dimethyl (3-methoxyphenyl)phosphonate (**10b**)*

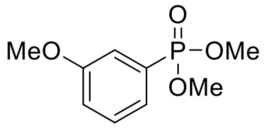

Prepared according to a **general procedure F** from 3-bromoanisole (**5d**) (0.187 g, 1.0 mmol).
Reaction time: 16 hours.
Yield: 69 % (0.148 g) yellow oil.

Prepared according to a **general procedure F** from 3-chloroanisole (**5e**) (0.142 g, 1.0 mmol).
Reaction time: 48 hours.
Yield: 56 % (0.120 g) yellow oil.

^{1}H NMR (400 MHz, DMSO-*d*$_6$) δ 7.53 – 7.44 (m, 1H), 7.27 (ddt, *J* = 12.8, 7.5, 1.2 Hz, 1H), 7.24 – 7.13 (m, 2H), 3.81 (s, 3H), 3.66 (s, 3H), 3.64 (s, 3H).
^{13}C NMR (101 MHz, DMSO-*d*$_6$) δ 159.1 (d, *J* = 18.7 Hz), 130.3 (d, *J* = 17.3 Hz), 128.3 (d, *J* = 185.4 Hz), 123.5 (d, *J* = 9.2 Hz), 118.6 (d, *J* = 3.2 Hz), 116.1 (d, *J* = 11.3 Hz), 55.3, 52.6, 52.5.
HR-MS (ESI+) *m/z:* calculated for $C_9H_{13}O_4P$ [M+H$^+$]: 217.0629, **observed**: 217.0624.

Spectral data are in agreement with previously reported characterization data for the title compound[15].

*Dimethyl (2-methoxyphenyl)phosphonate (**10c**)*

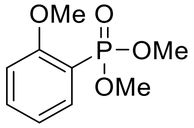

Prepared according to a **general procedure F** from 2-bromoanisole (**5f**) (0.187 g, 1.0 mmol).
Reaction time: 16 hours.
Yield: 57 % (0.123 g) yellow oil.

Prepared according to a **general procedure F** from 2-chloroanisole (**5g**) (0.142 g, 1.0 mmol).
Reaction time: 48 hours.
Yield: 49 % (0.107 g) yellow oil.

^{1}H NMR (400 MHz, DMSO-d_6) δ 7.61 (m, 2H), 7.19 – 7.11 (m, 1H), 7.06 (tdd, *J* = 7.4, 3.4, 0.9 Hz, 1H), 3.84 (s, 3H), 3.66 (s, 3H), 3.63 (s, 3H).
^{13}C NMR (101 MHz, DMSO-d_6) δ 160.9, 134.7(d, *J* = 2.0 Hz), 134.6 (d, *J* = 7.1 Hz), 120.3 (d, *J* = 14.2 Hz), 115.3 (d, *J* = 186.2 Hz), 111.9 (d, *J* = 9.2 Hz), 55.9, 52.5, 52.4.
HR-MS (ESI+) *m/z:* calculated for $C_9H_{13}O_4P$ [M+H$^+$]: 217.0629, **observed**: 217.0624.
Spectral data are in agreement with previously reported characterization data for the title compound[15].

*Dimethyl thiophen-2-ylphosphonate (**10e**)*

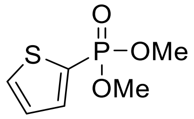

Prepared according to a **general procedure G** from 2-bromothiophene (**5j**) (0.163 g, 1.0 mmol).
Reaction time: 24 hours.
Yield: 55 % (0.105 g) yellow oil.

Prepared according to a **general procedure F** from 2-chlorothiophene (**5k**) (0.118 g, 1.0 mmol).
Reaction time: 32 hours.
Yield: 53 % (0.103 g) yellow oil.

^{1}H NMR (400 MHz, DMSO-d_6) δ 8.10 (ddd, *J* = 5.8, 4.8, 1.1 Hz, 1H), 7.66 (ddd, *J* = 7.9, 3.6, 1.1 Hz, 1H), 7.31 (dt, *J* = 4.8, 3.4 Hz, 1H), 3.68 (s, 3H), 3.65 (s, 3H).
^{13}C NMR (101 MHz, DMSO-d_6) δ 137.0 (d, *J* = 12.5 Hz), 134.9 (d, *J* = 7.0 Hz), 128.7 (d, *J* = 18.2 Hz), 125.9 (d, *J* = 208.2 Hz), 52.9, 52.9.
HR-MS (ESI+) *m/z:* calculated for $C_6H_9O_3P$ [M+H$^+$]: 193.0088, **observed**: 193.0083.

Spectral data are in agreement with previously reported characterization data for the title compound[16].

S12. NMR SPECTRA OF DEAZAALLOXAZINES

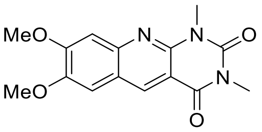

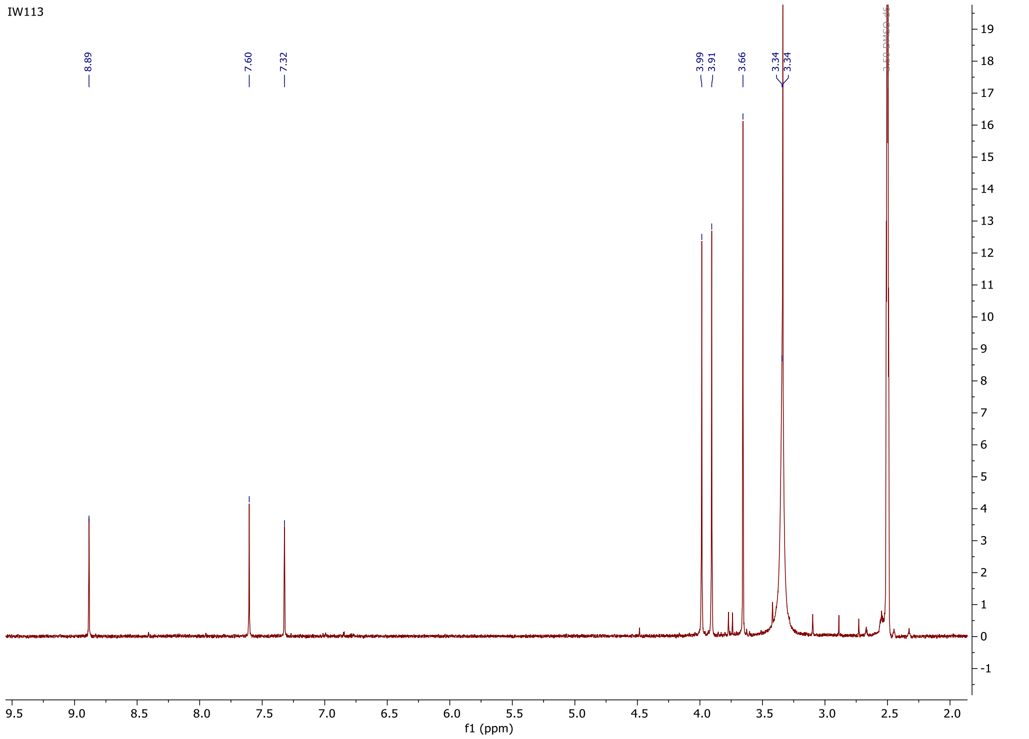

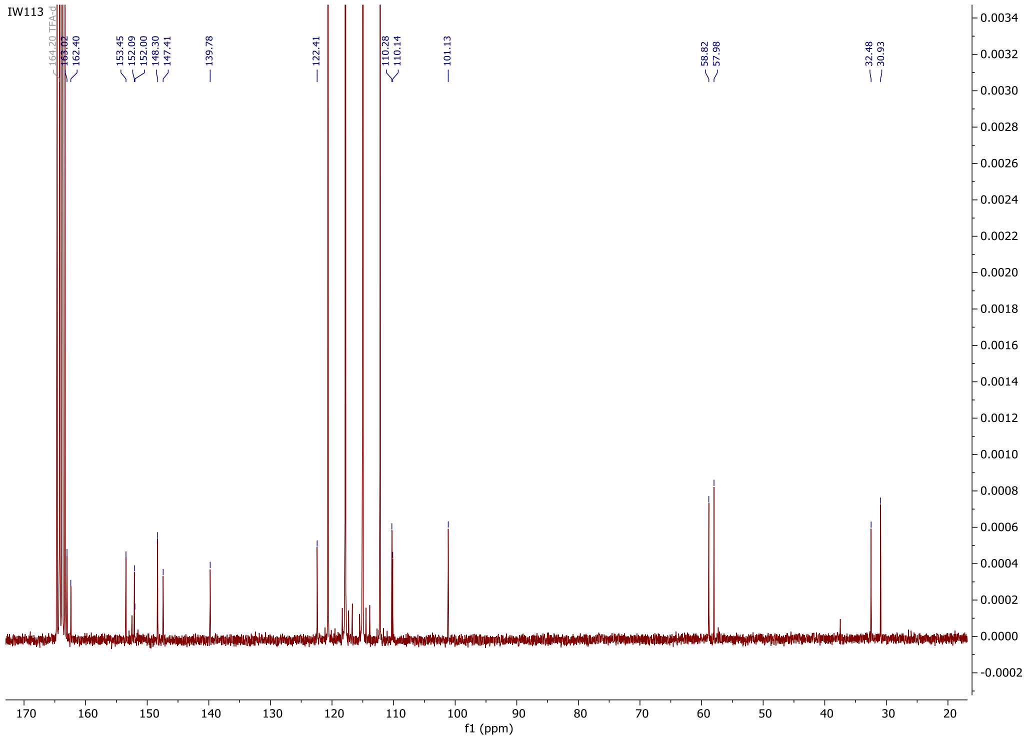

Figure S41: ^{1}H and ^{13}C NMR spectra of **2a**.

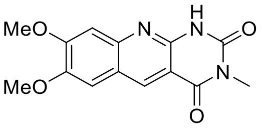

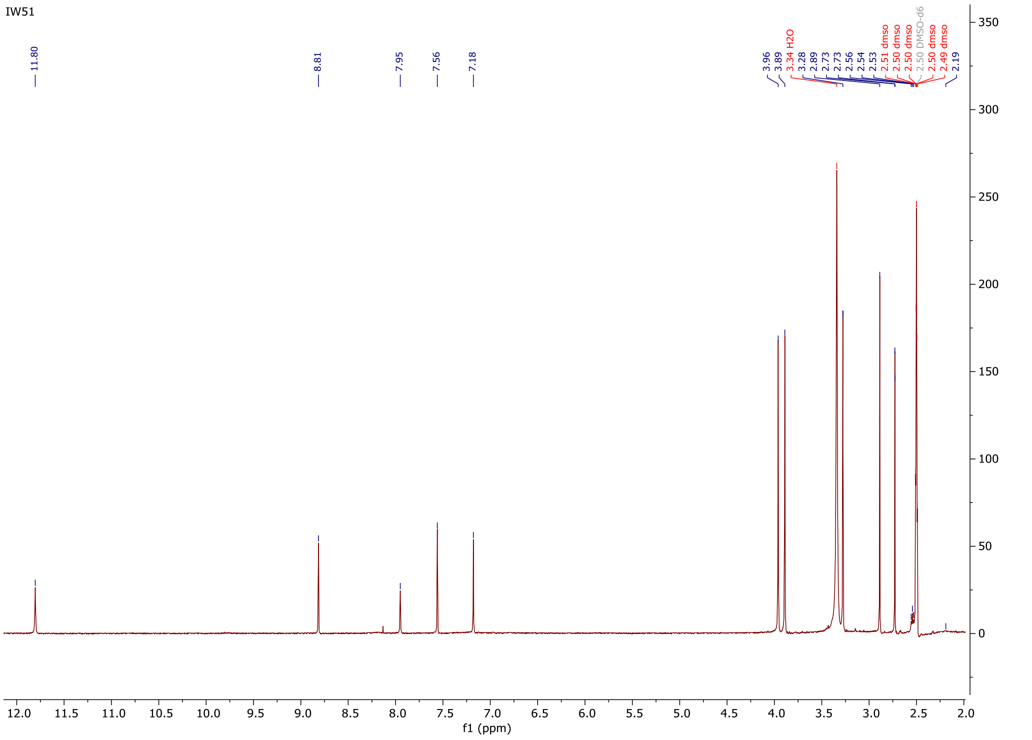

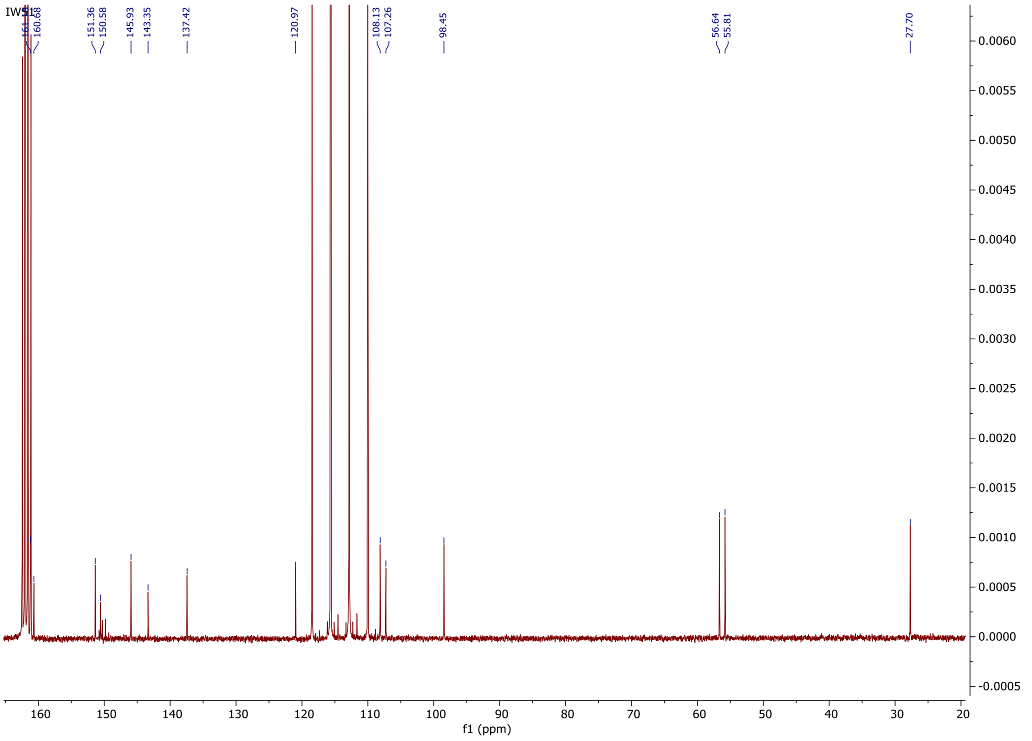

Figure S42: ^{1}H and ^{13}C NMR spectra of **2a-H**.

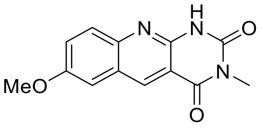

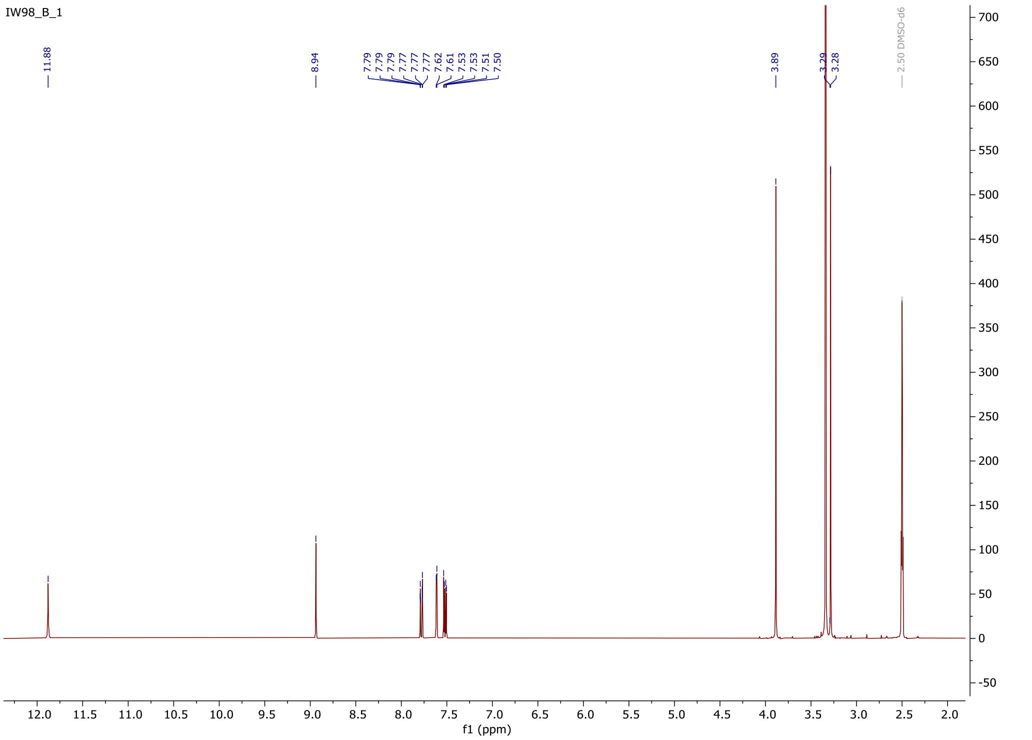

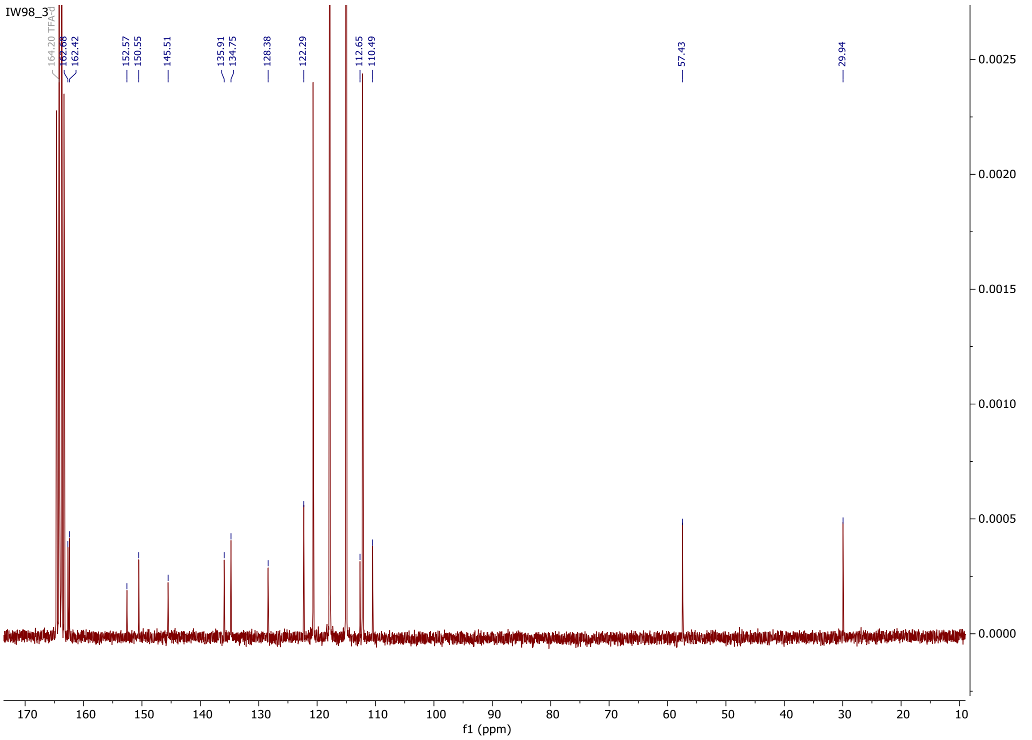

Figure S43: ^{1}H and ^{13}C NMR spectra of **2b-H**.

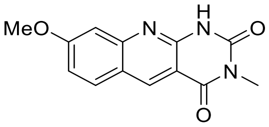

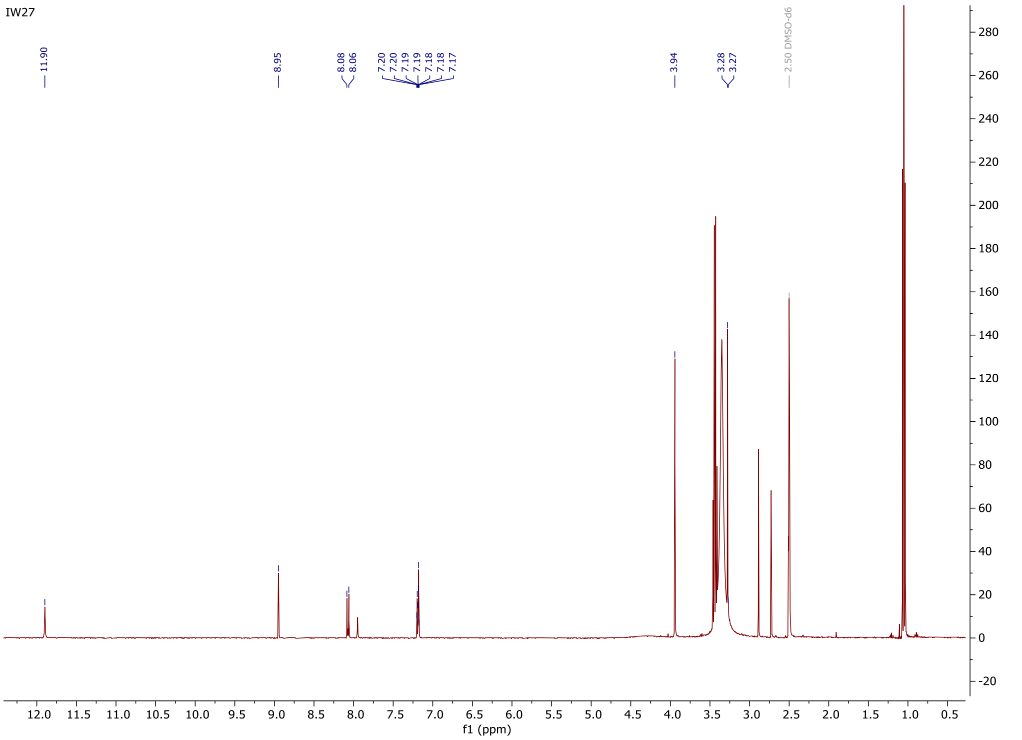

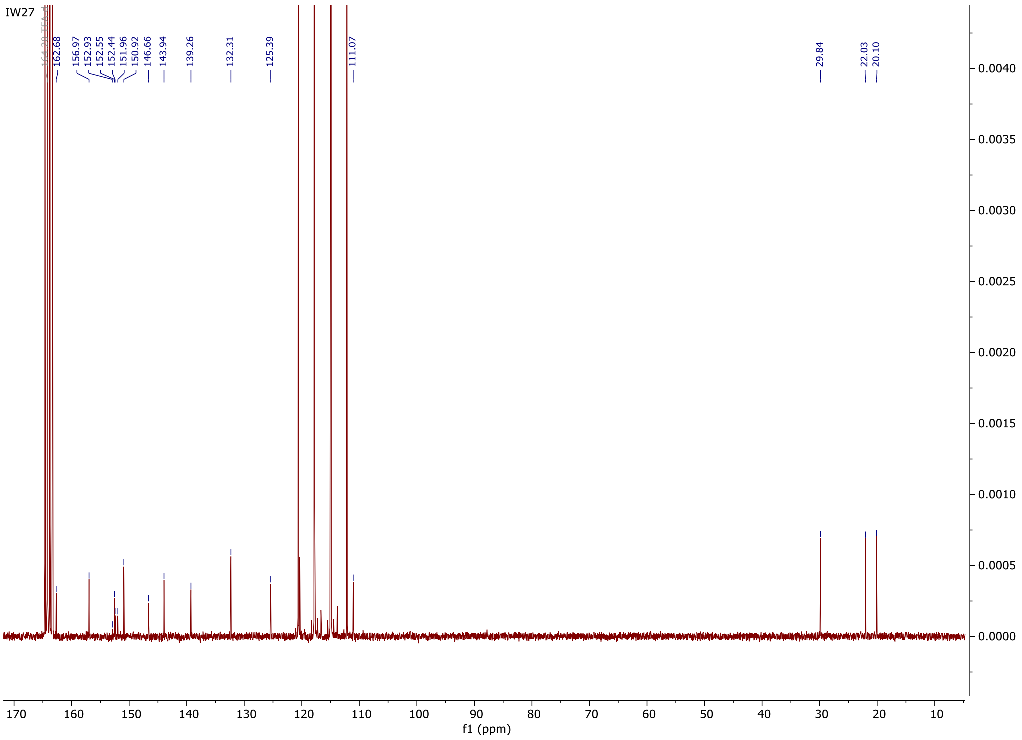

Figure S44: ^{1}H and ^{13}C NMR spectra of **2c-H**.

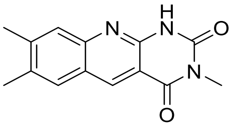

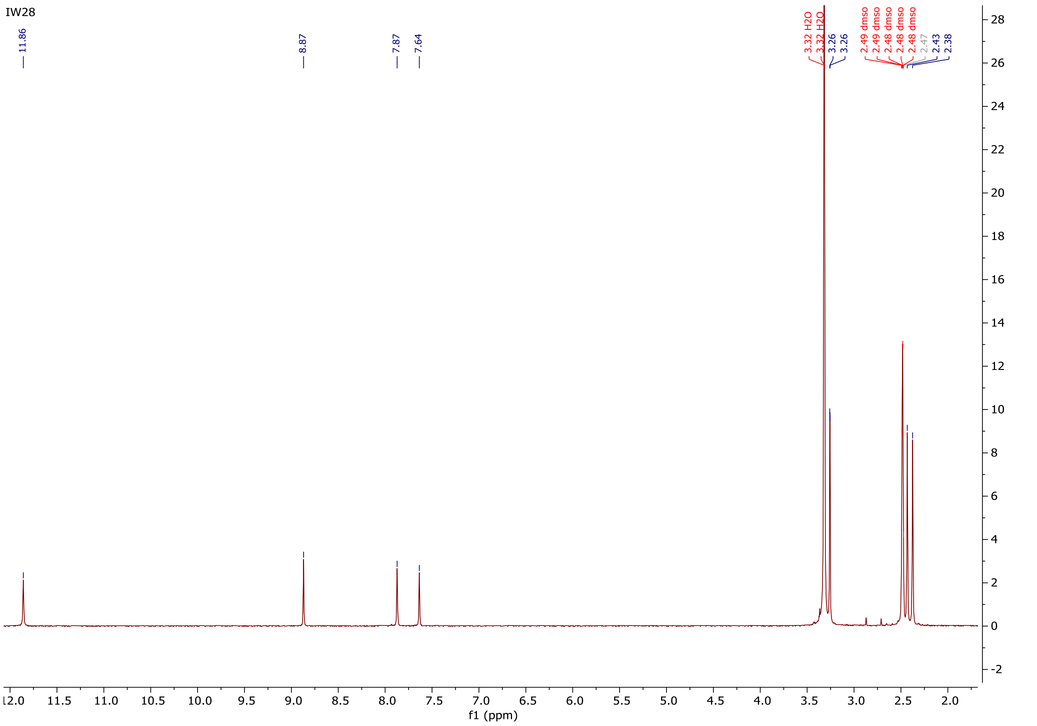

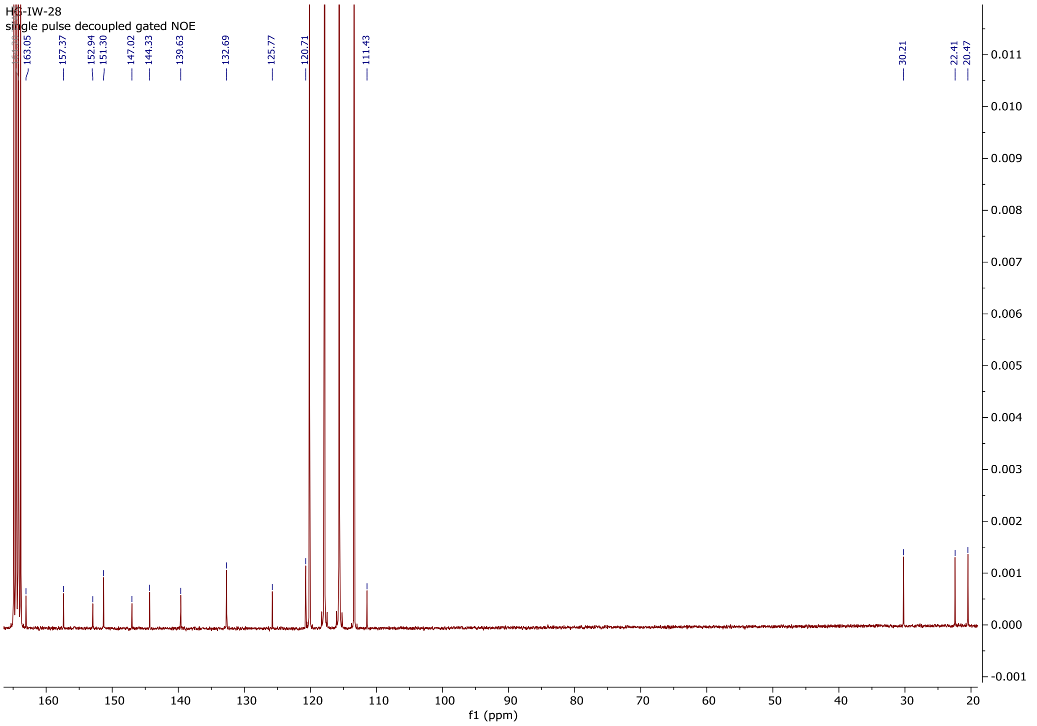

Figure S45: ^{1}H and ^{13}C NMR spectra of **2d-H**.

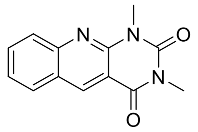

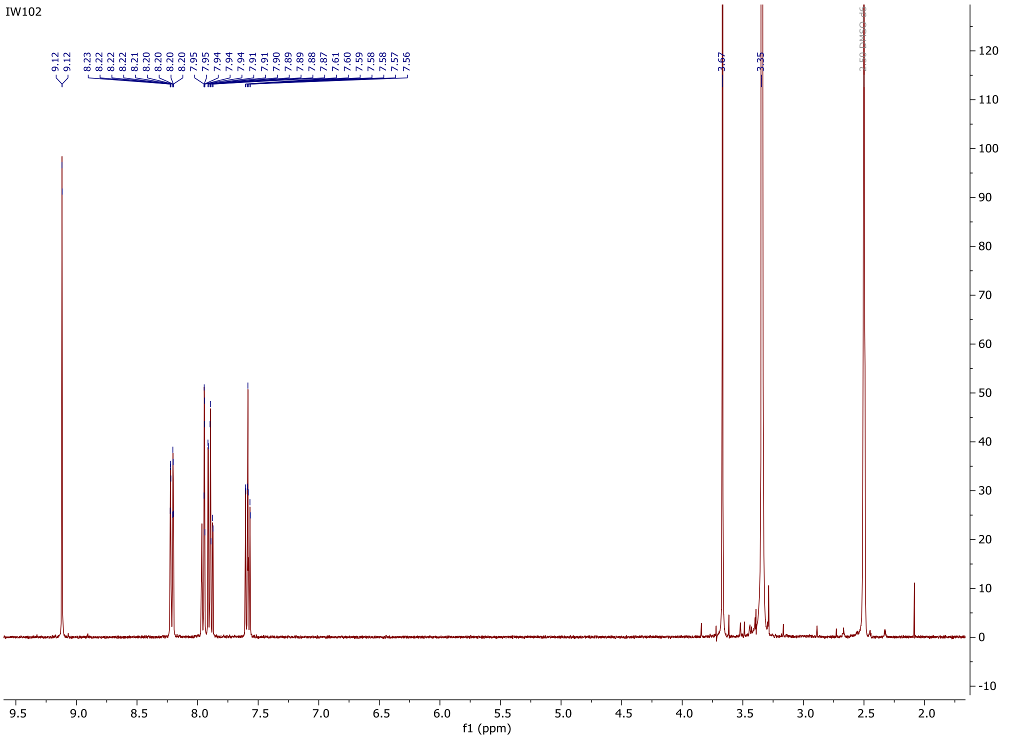

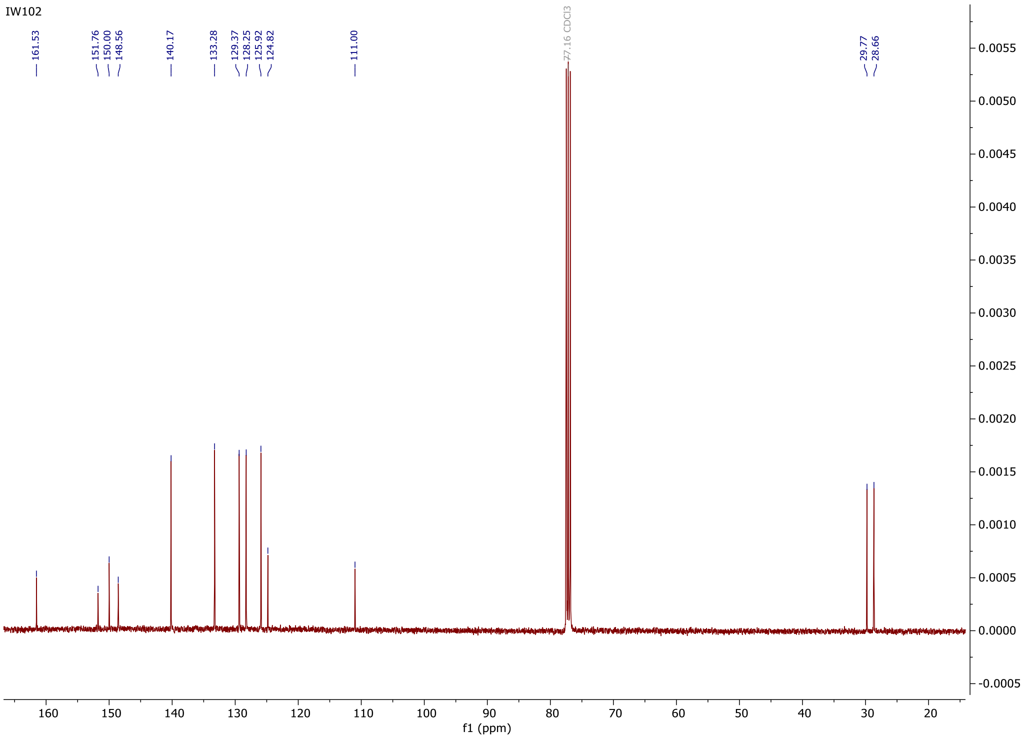

Figure S46: ^{1}H and ^{13}C NMR spectra of **2e**.

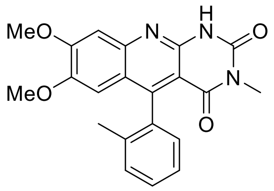

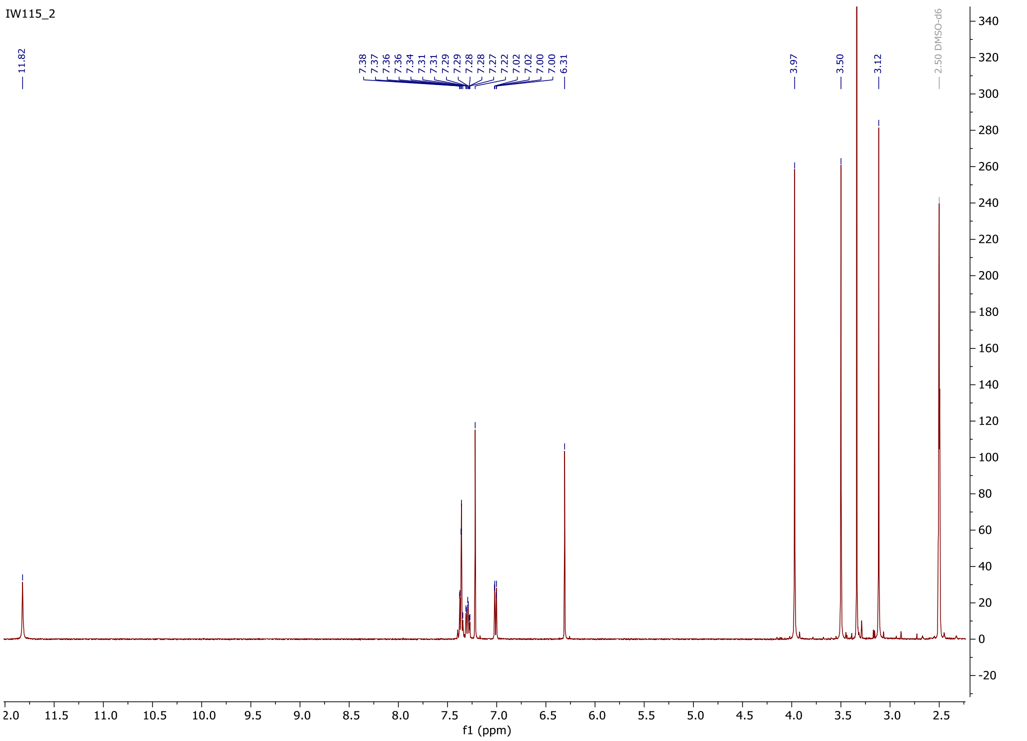

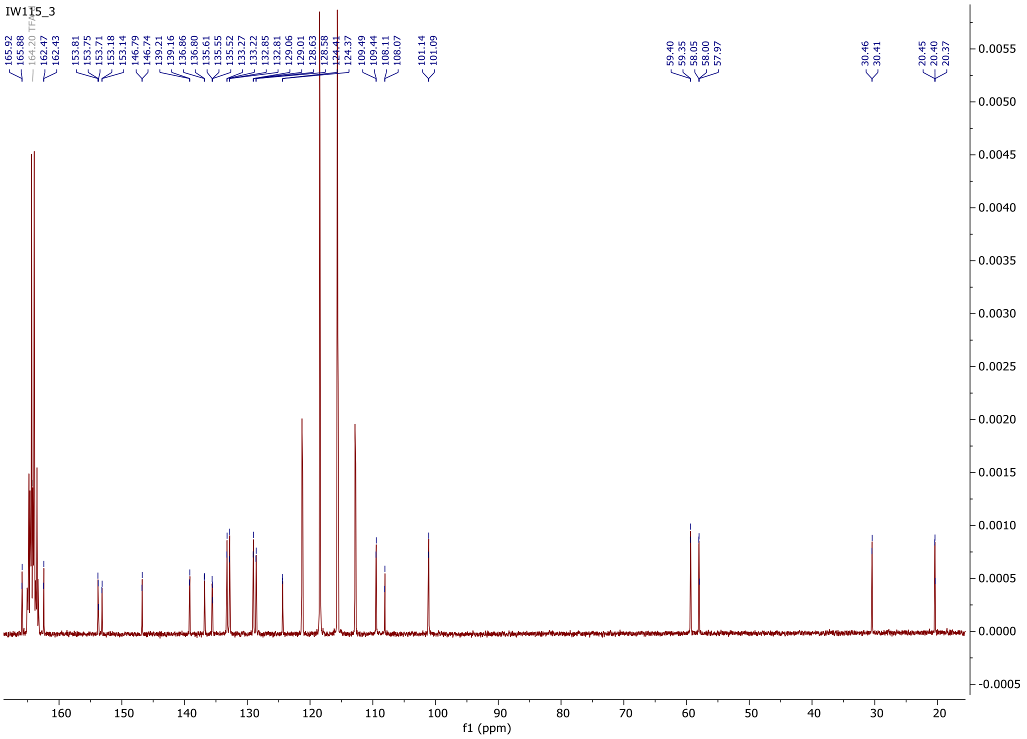

Figure S47: ^{1}H and ^{13}C NMR spectra of **3a(*o*-MePh)-H**.

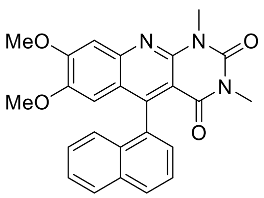

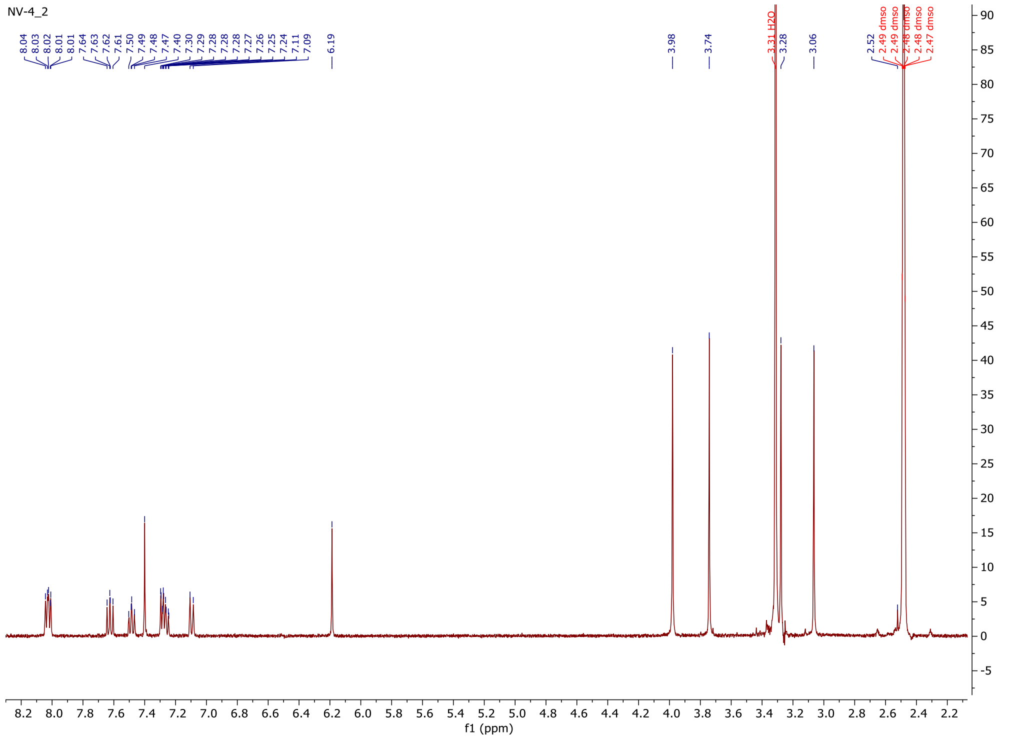

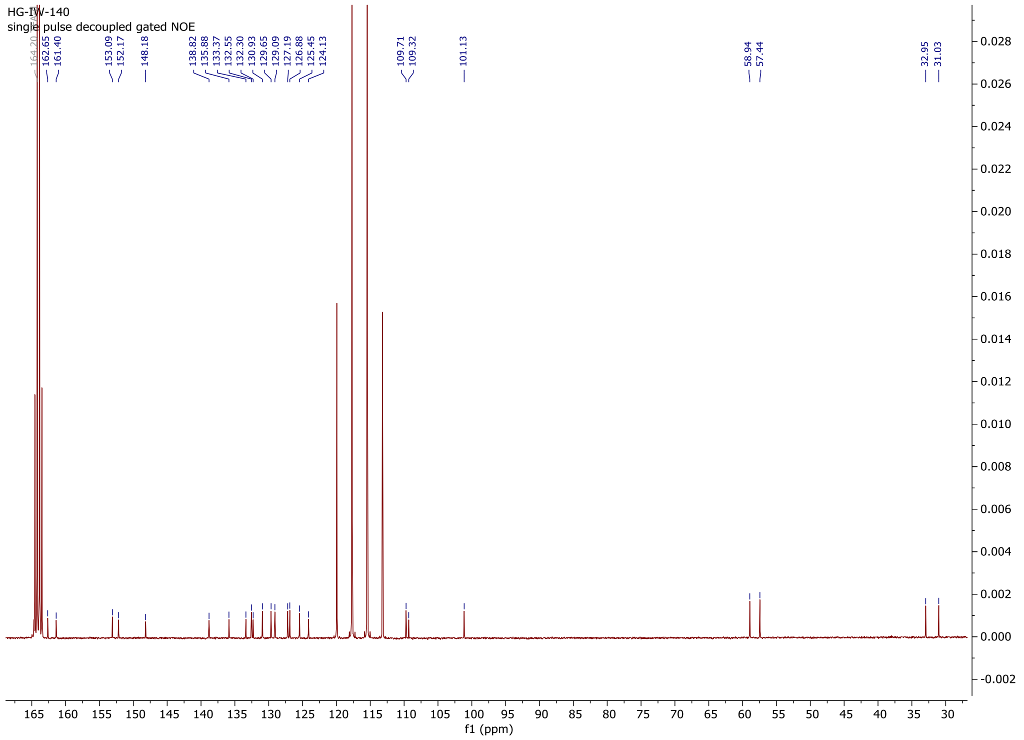

Figure S48: ^{1}H and ^{13}C NMR spectra of **3a(napht-1-yl)**.

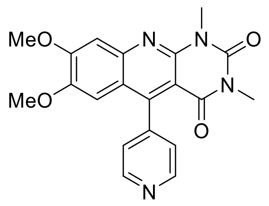

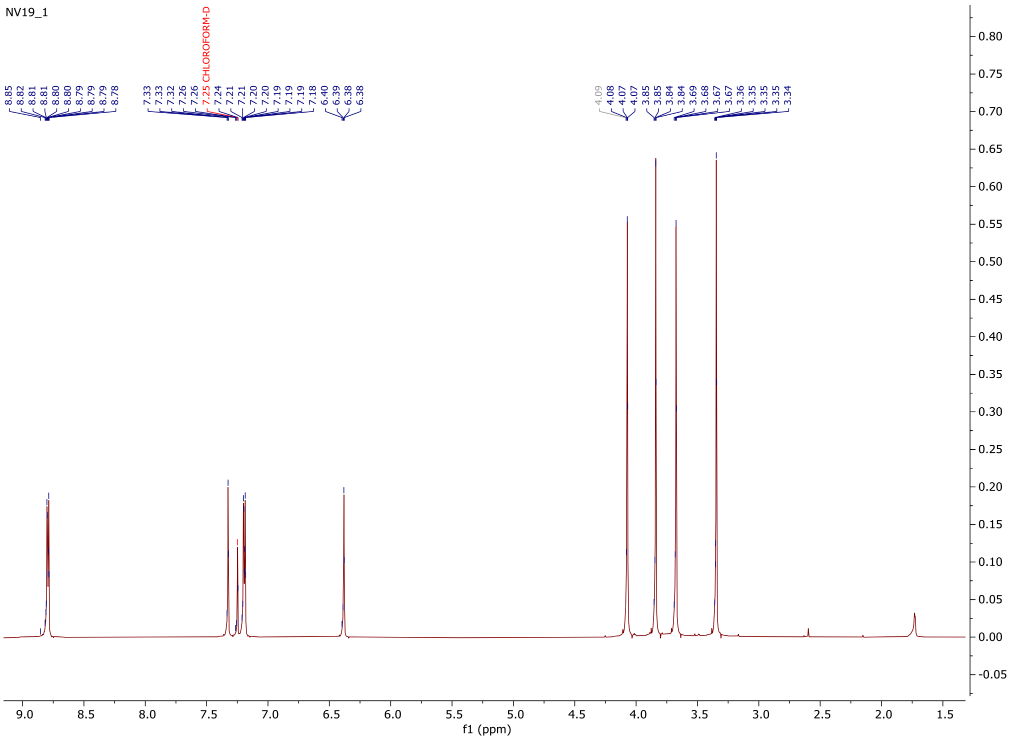

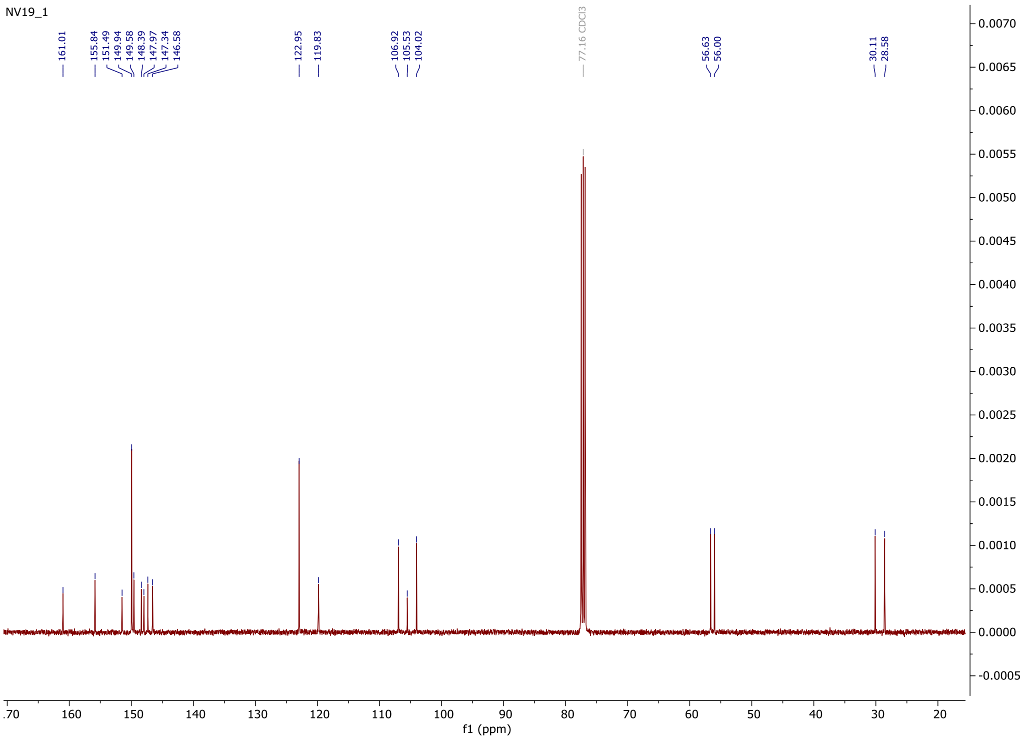

Figure S49: ^{1}H and ^{13}C NMR spectra of **3a(pyridin-4-yl)**.

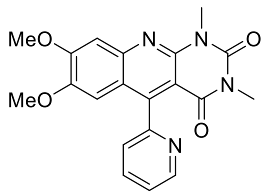

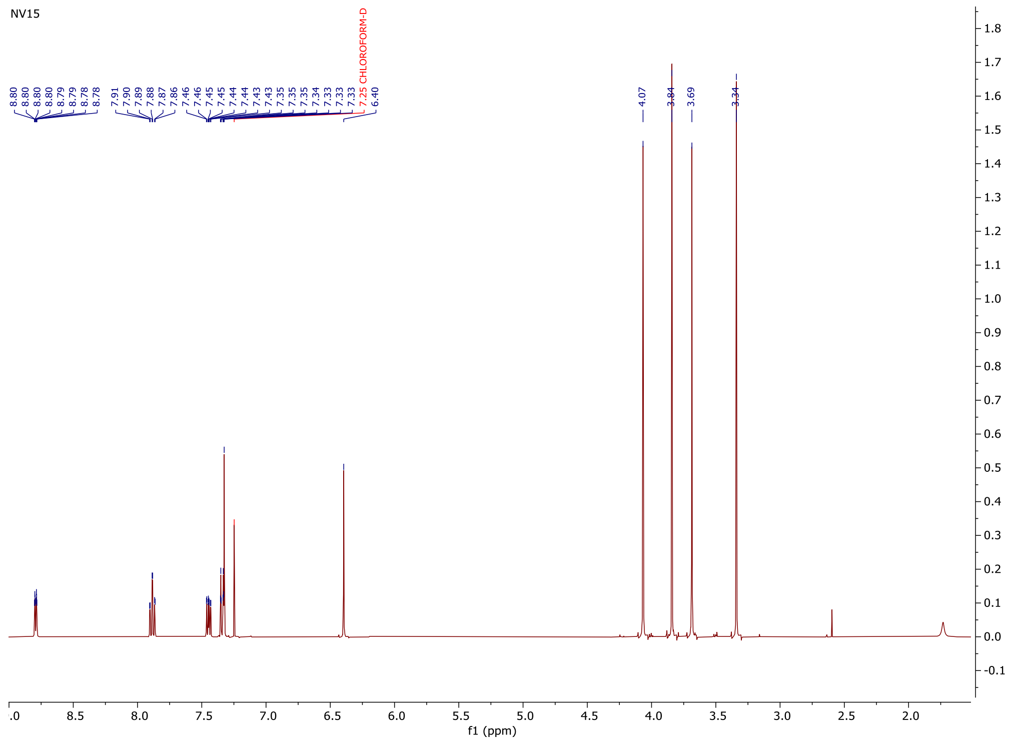

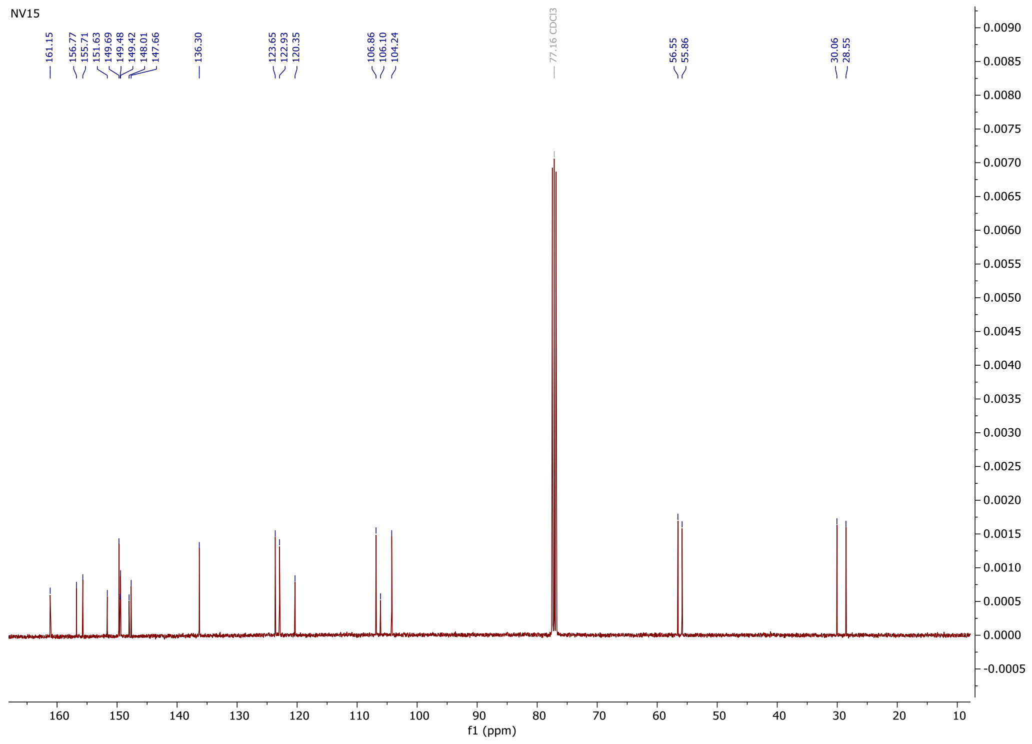

Figure S50: ^{1}H and ^{13}C NMR spectra of **3a(pyridin-2-yl)**.

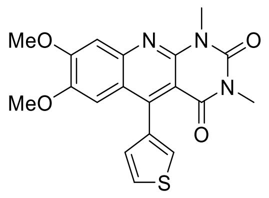

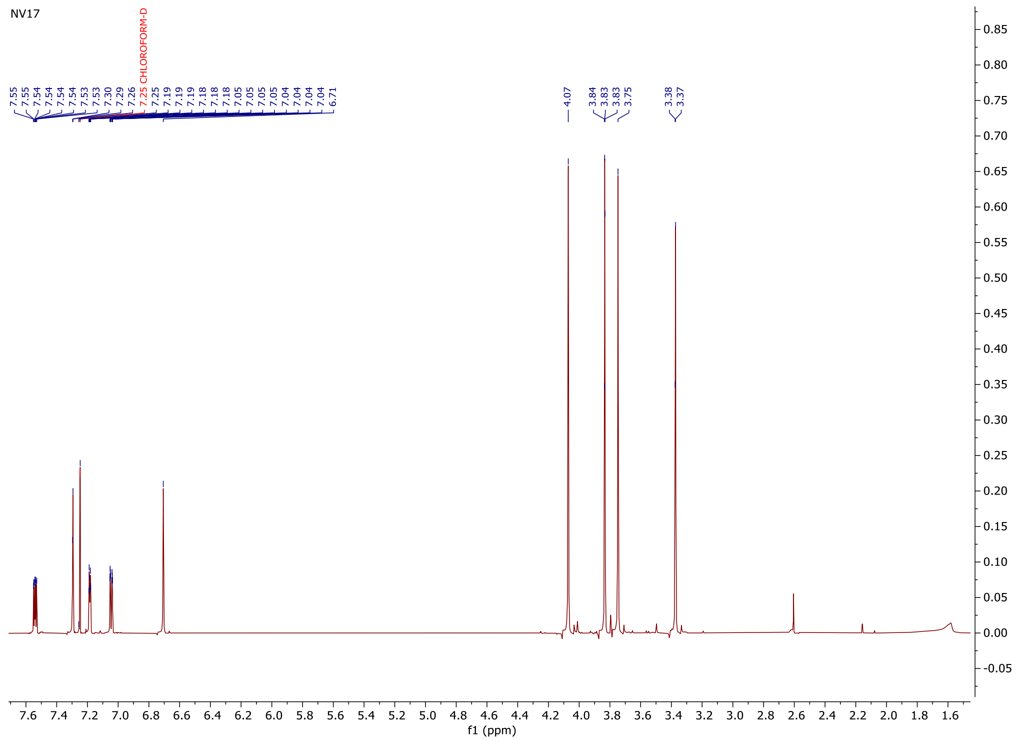

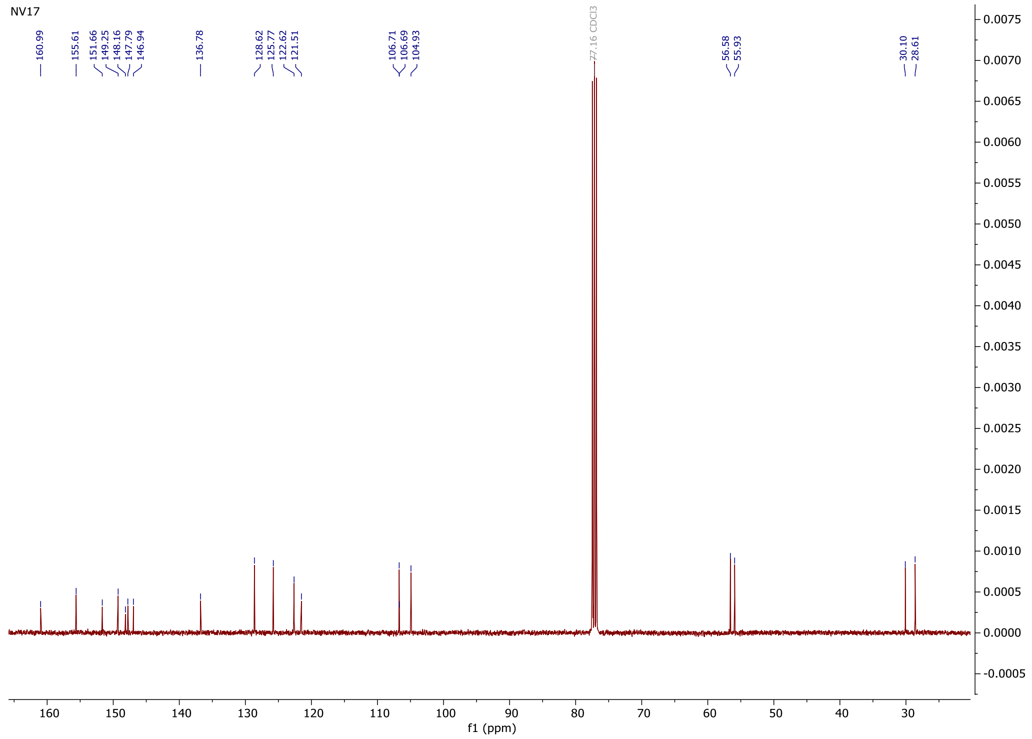

Figure S51: ^{1}H and ^{13}C NMR spectra of **3a(thiophene-3-yl)**.

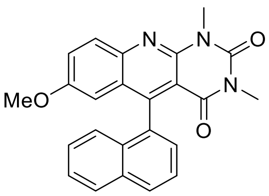

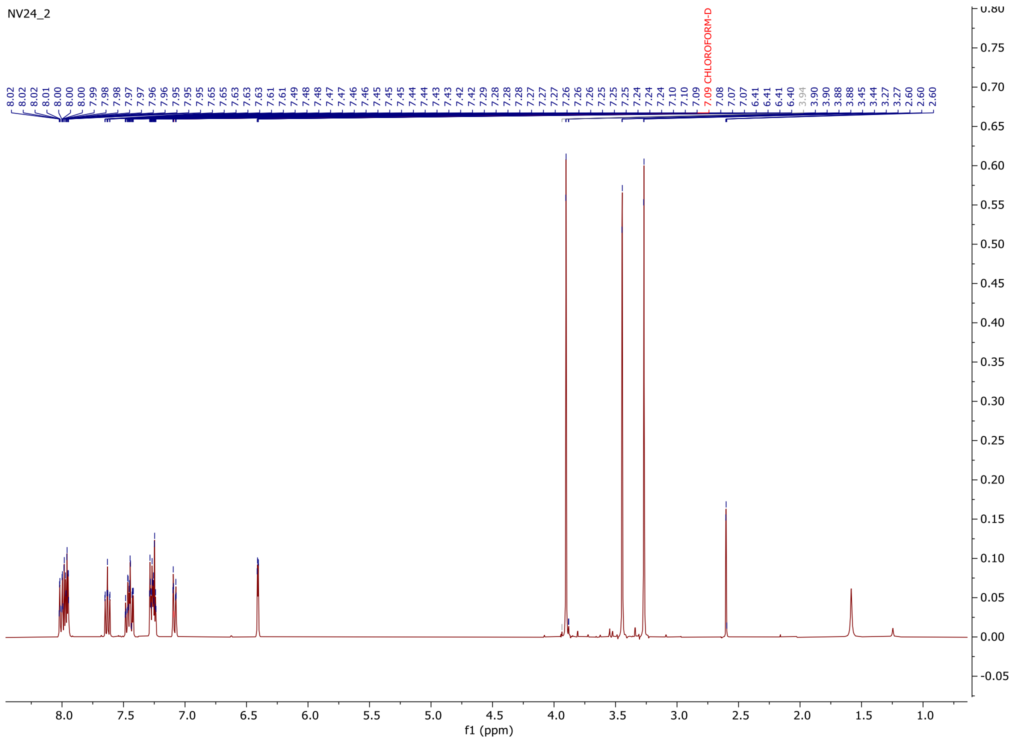

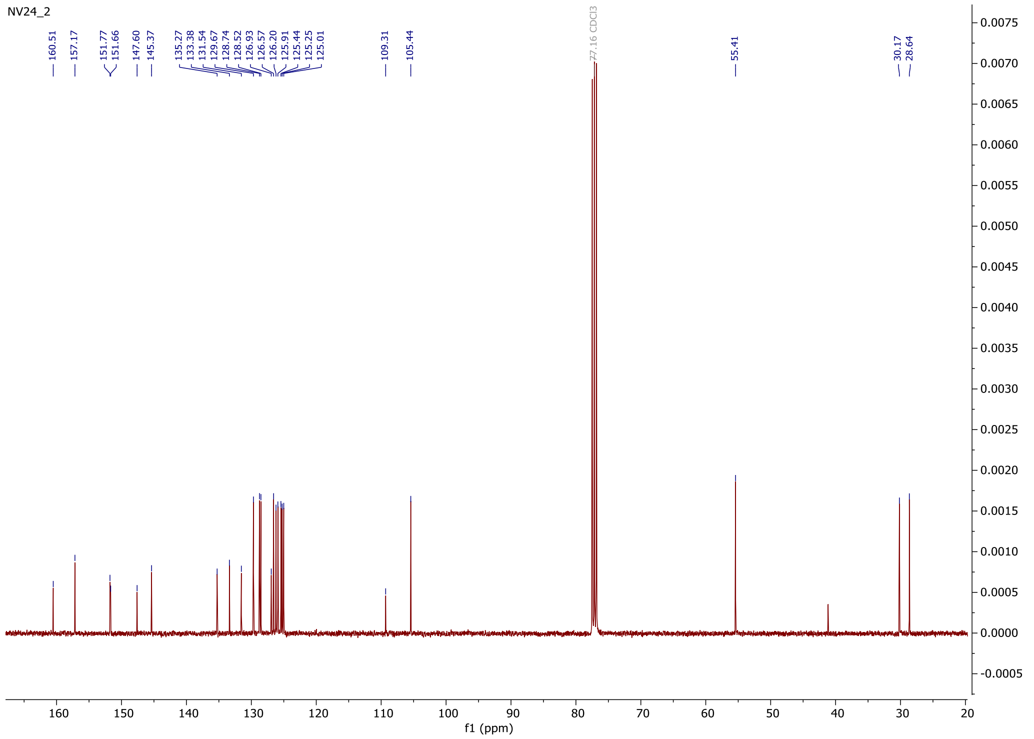

Figure S52: ^{1}H and ^{13}C NMR spectra of **3b(napht-1-yl)**.

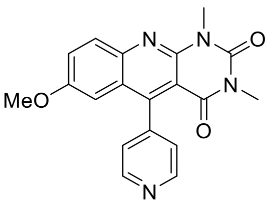

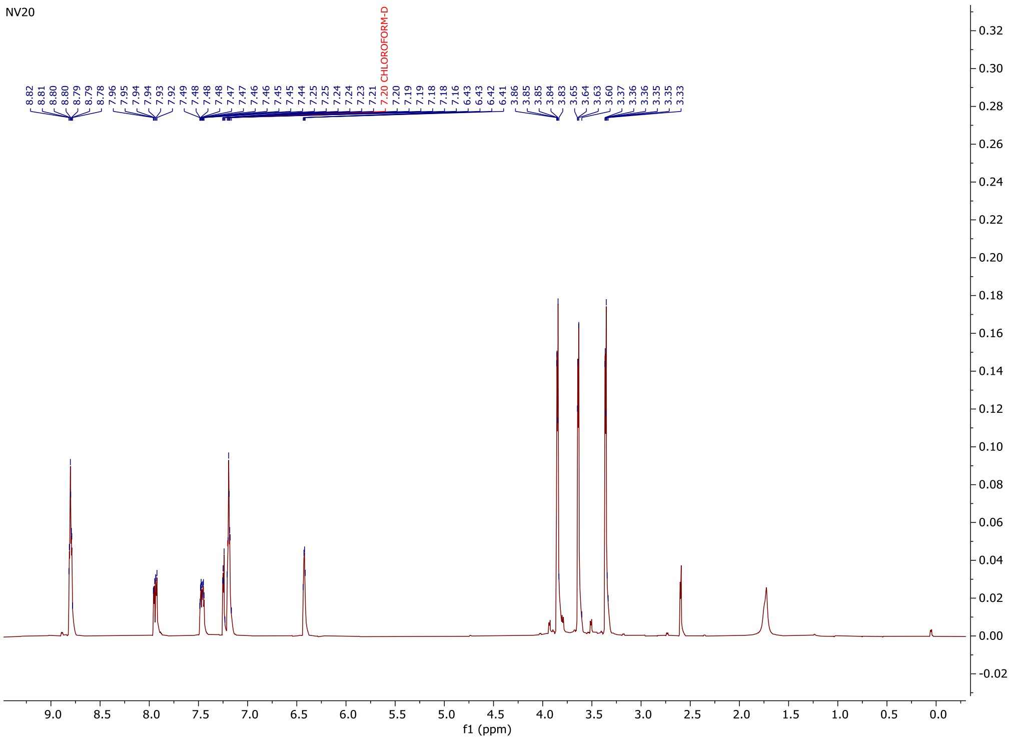

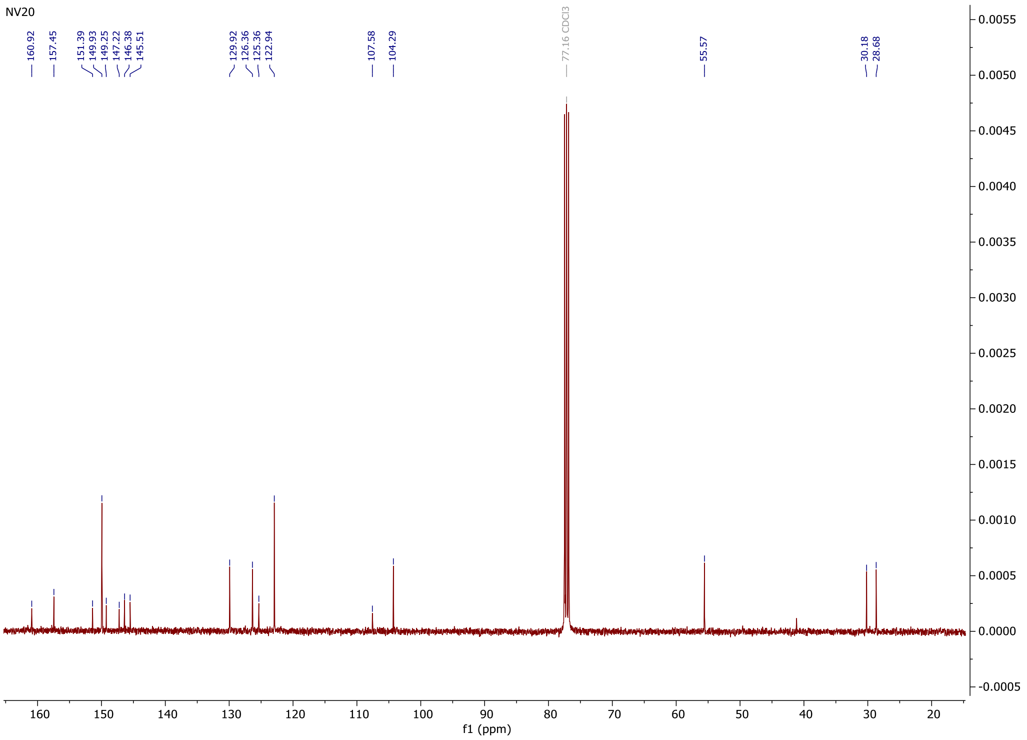

Figure S53: ^{1}H and ^{13}C NMR spectra of **3a(pyridin-4-yl)**.

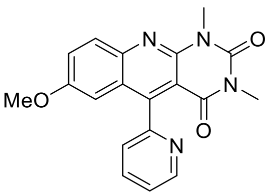

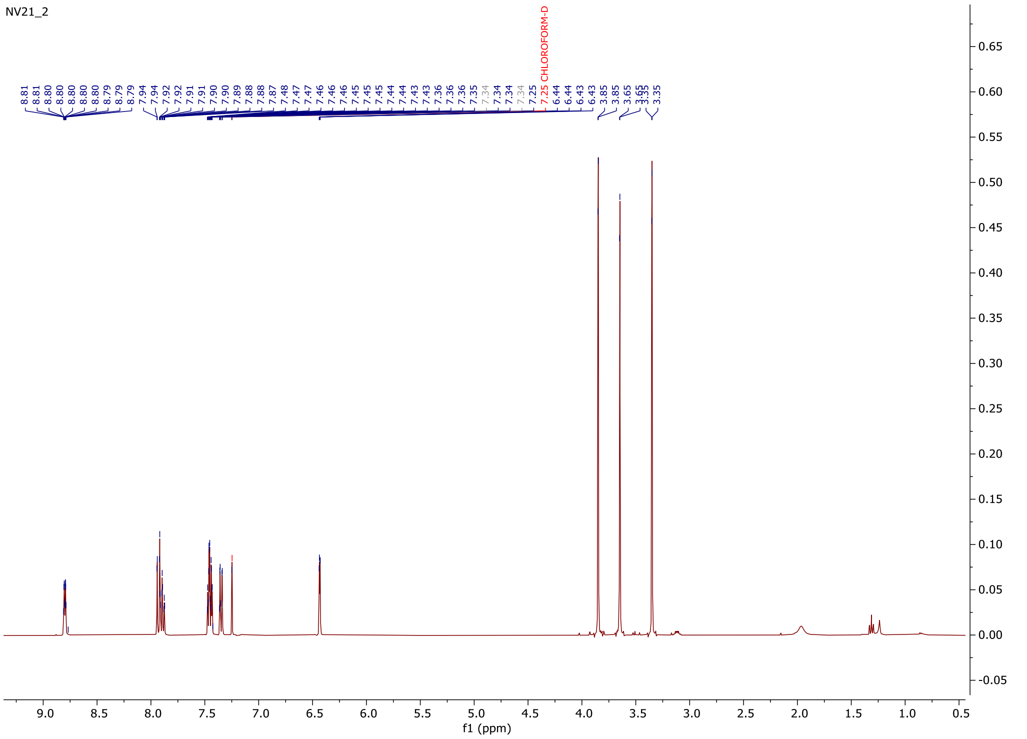

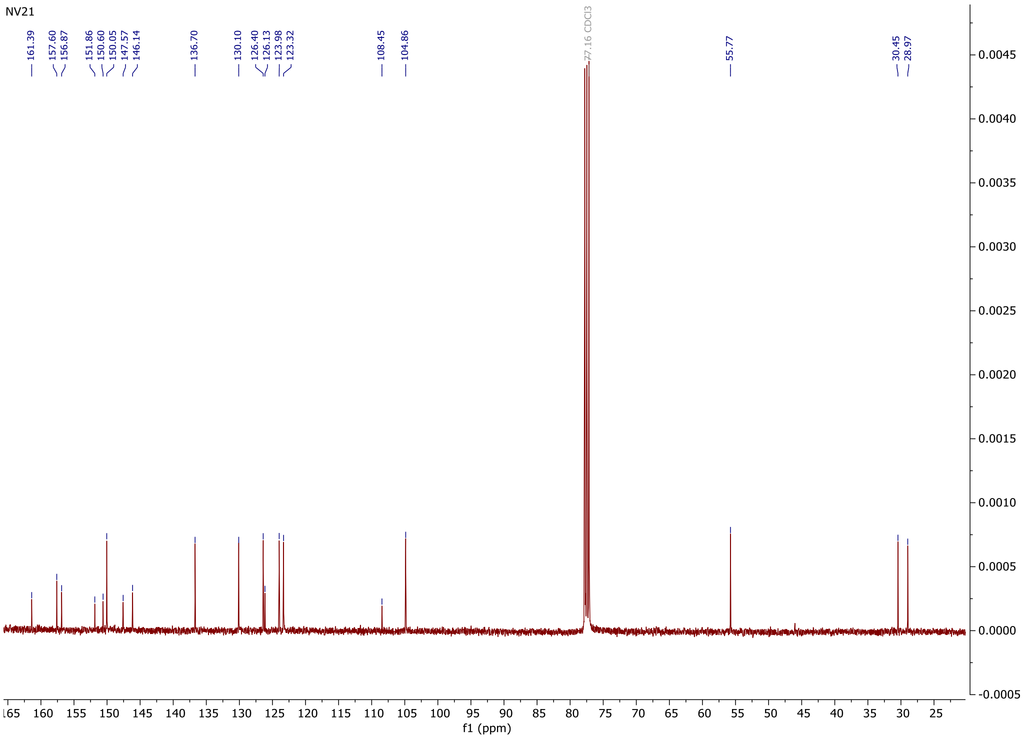

Figure S54: ^{1}H and ^{13}C NMR spectra of **3a(pyridin-2-yl)**.

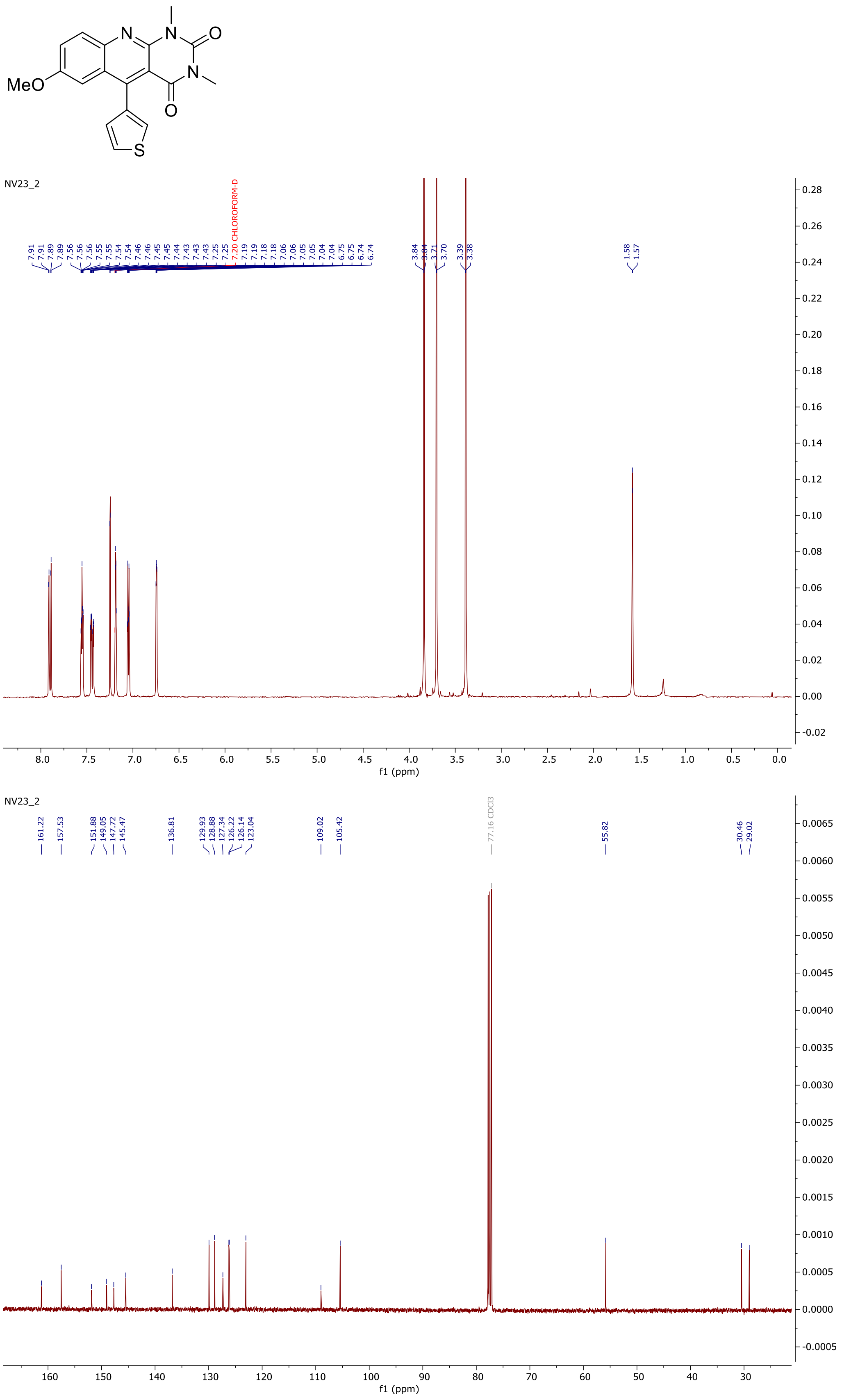

Figure S55: ^{1}H and ^{13}C NMR spectra of **3b(thiophene-3-yl)**.

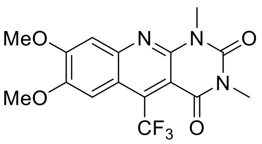
MeO
MeO
N
N
O
N
O
CF3

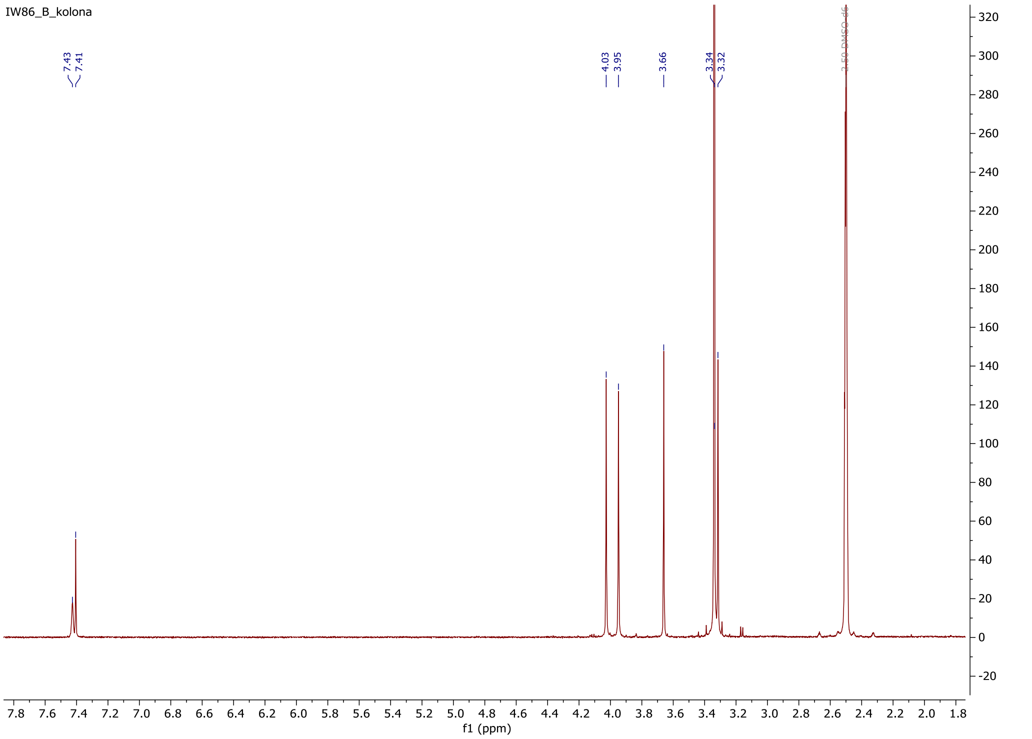
IW86_B_kolona
7.43
7.41
4.03
3.95
3.66
3.34
3.32
f1 (ppm)

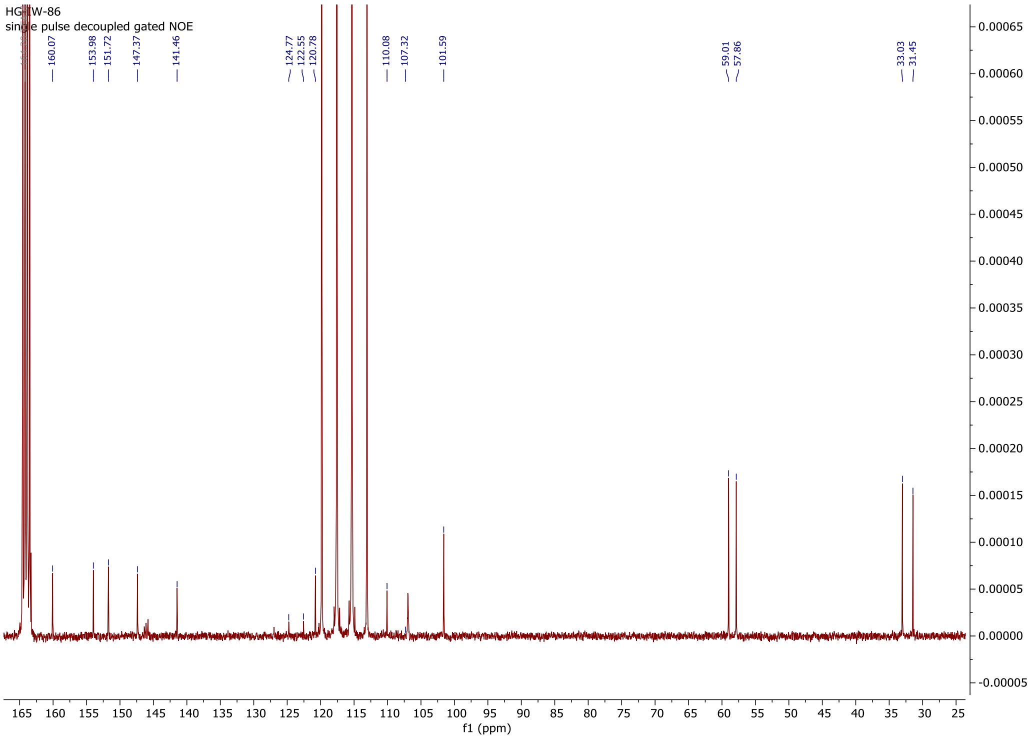
single pulse decoupled gated NOE
160.07
153.98
151.72
147.37
141.46
124.77
122.55
120.78
110.08
107.32
101.59
59.01
57.86
33.03
31.45
f1 (ppm)

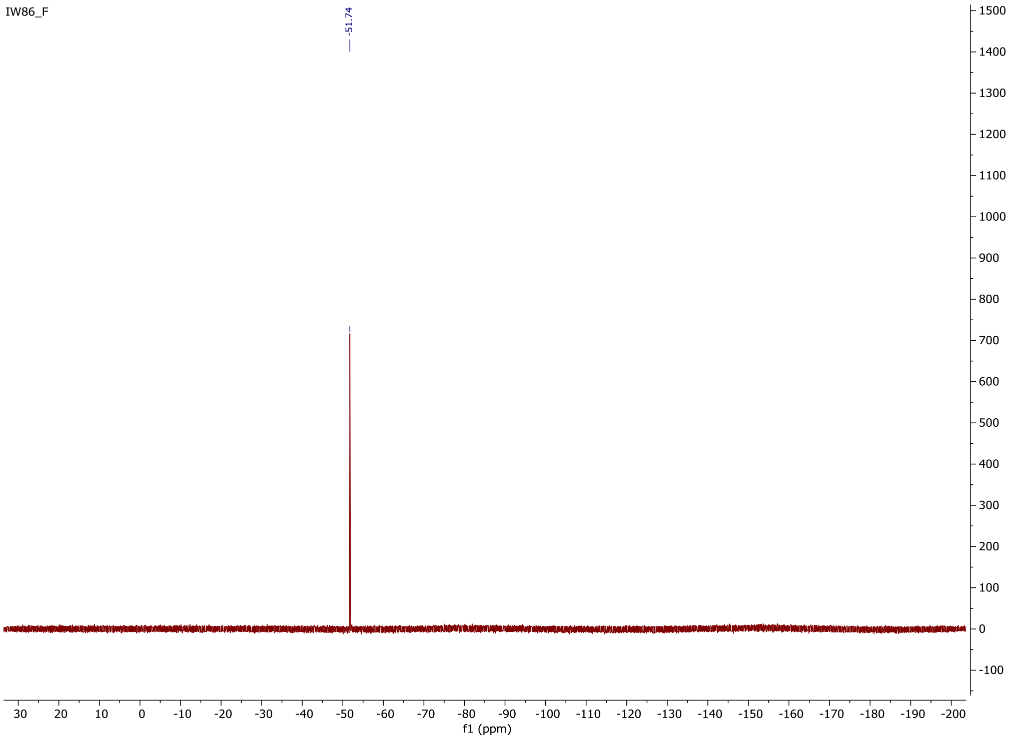

Figure S56: ^{1}H, ^{13}C NMR and ^{19}F spectra of **4a**.

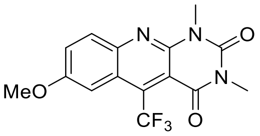

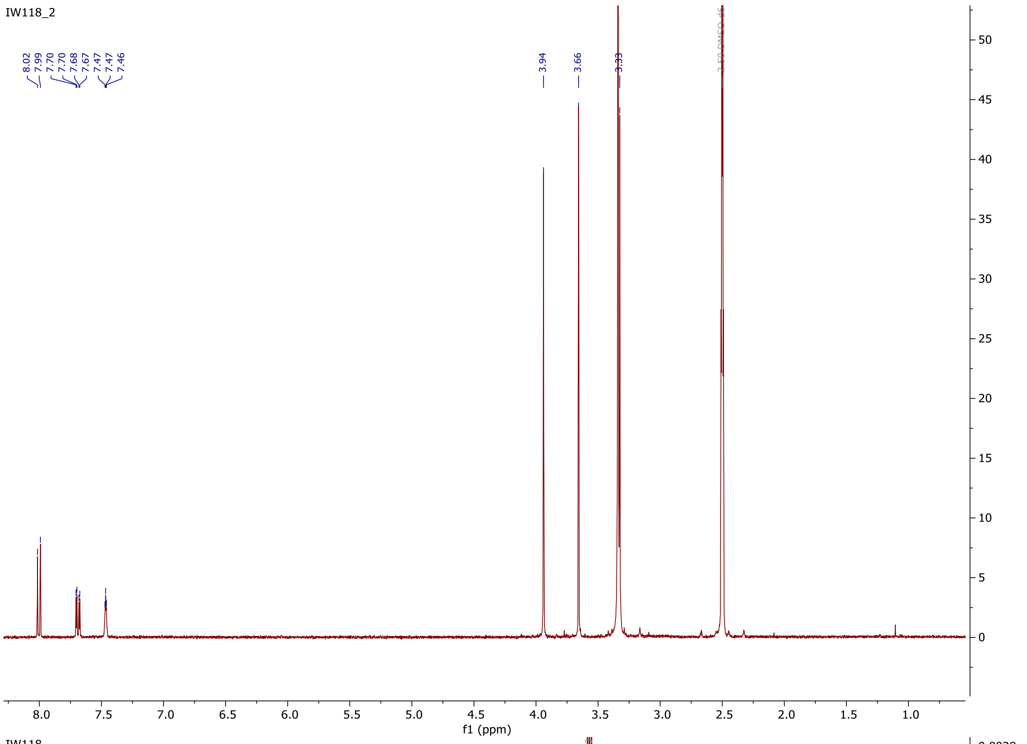
IW118_2
8.02
7.99
7.70
7.70
7.68
7.67
7.47
7.47
7.46
3.94
3.66
3.33
f1 (ppm)

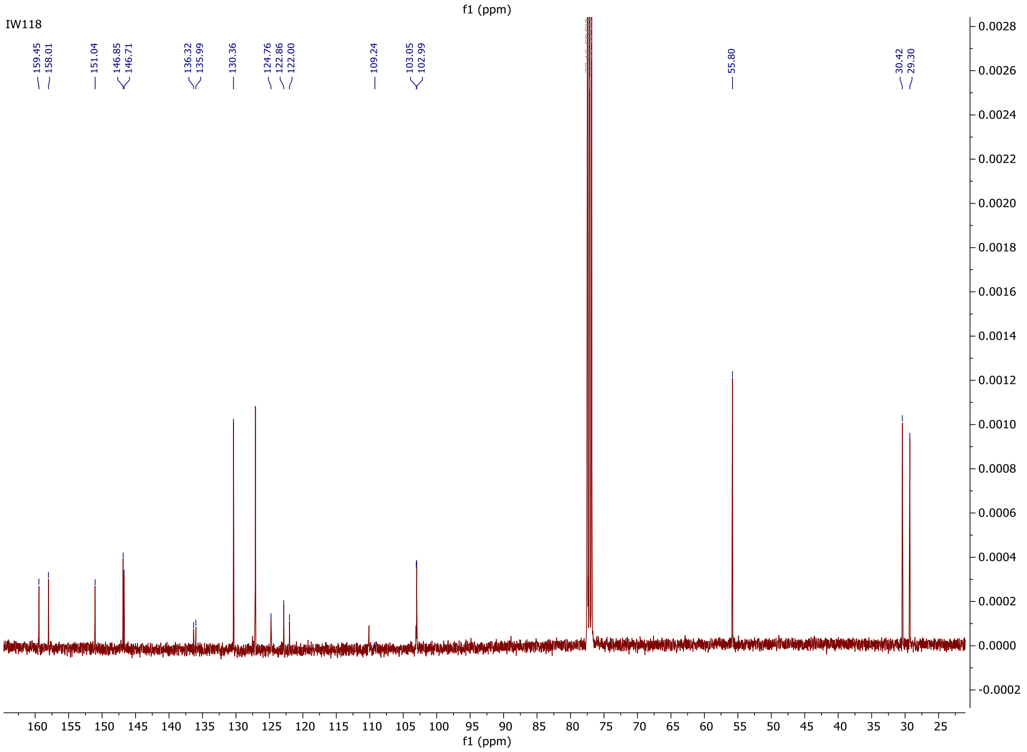
IW118
159.45
158.01
151.04
146.85
146.71
136.32
135.99
130.36
124.76
122.86
122.00
109.24
103.05
102.99
55.80
30.42
29.30
f1 (ppm)

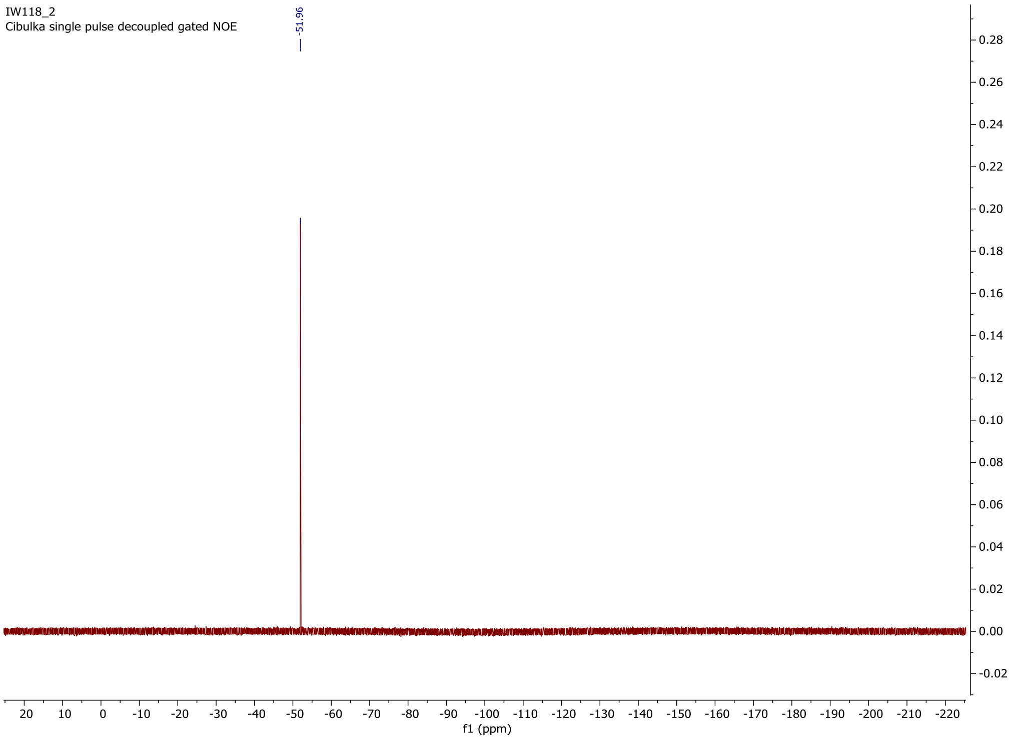

Figure S57: ^{1}H, ^{13}C NMR and ^{19}F spectra of **4b**.

S13. NMR SPECTRA OF ISOLATED PRODUCTS OF PHOTOREDUCTIVE DEPROTECTION

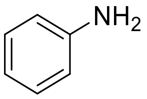

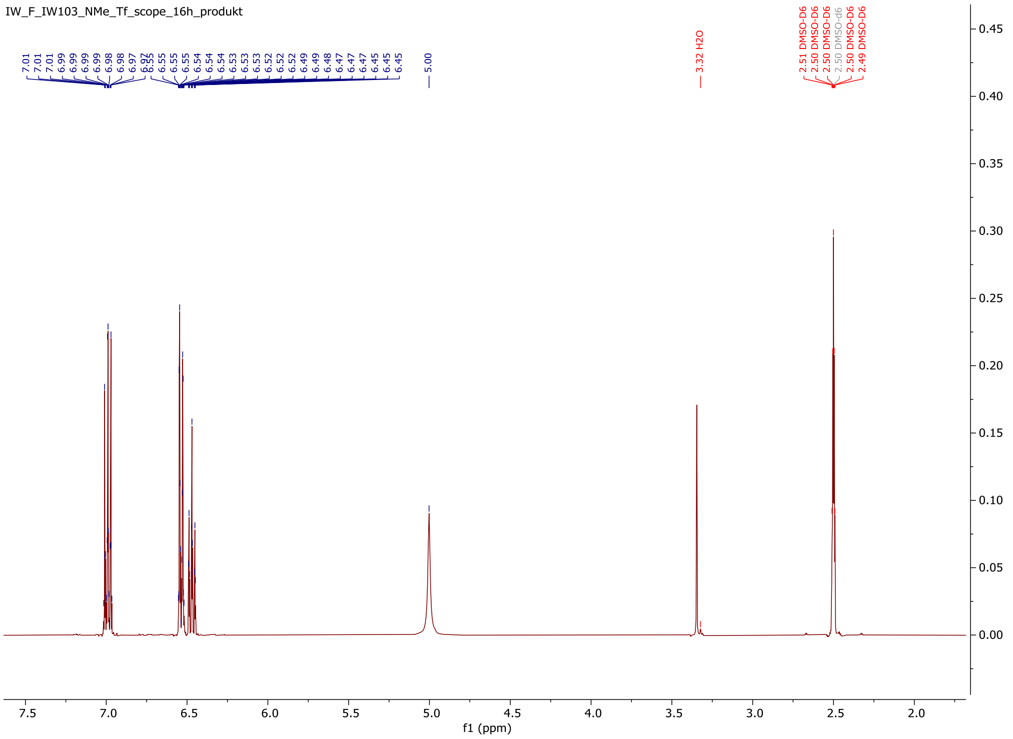

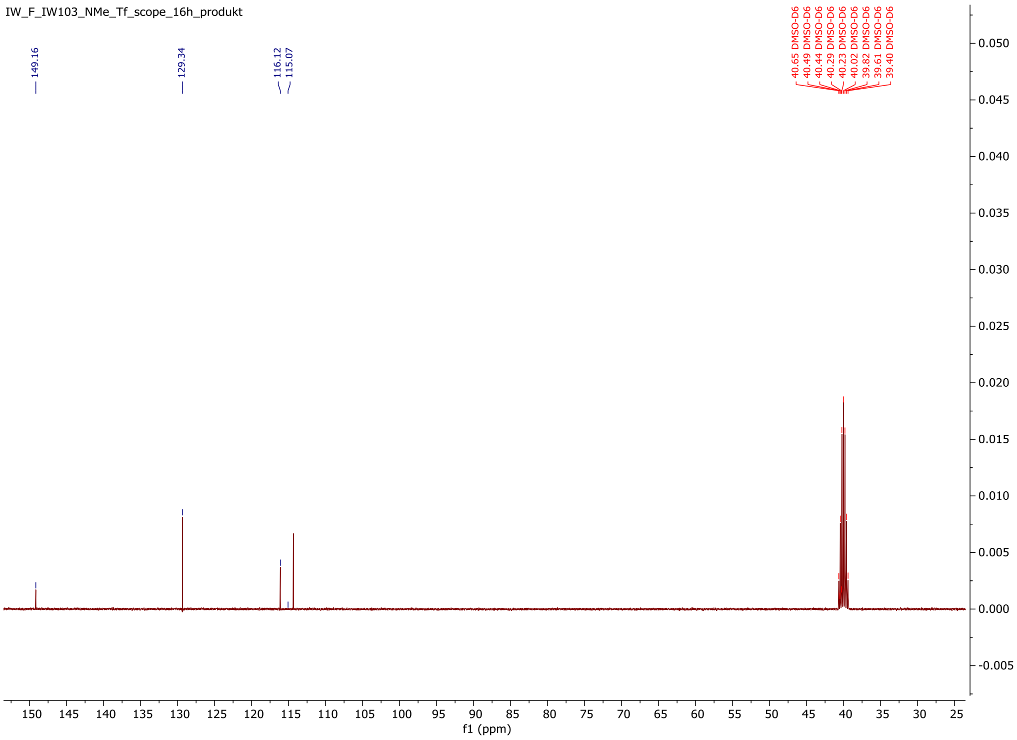

Figure S58: ^{1}H and ^{13}C NMR spectra of **8a**.

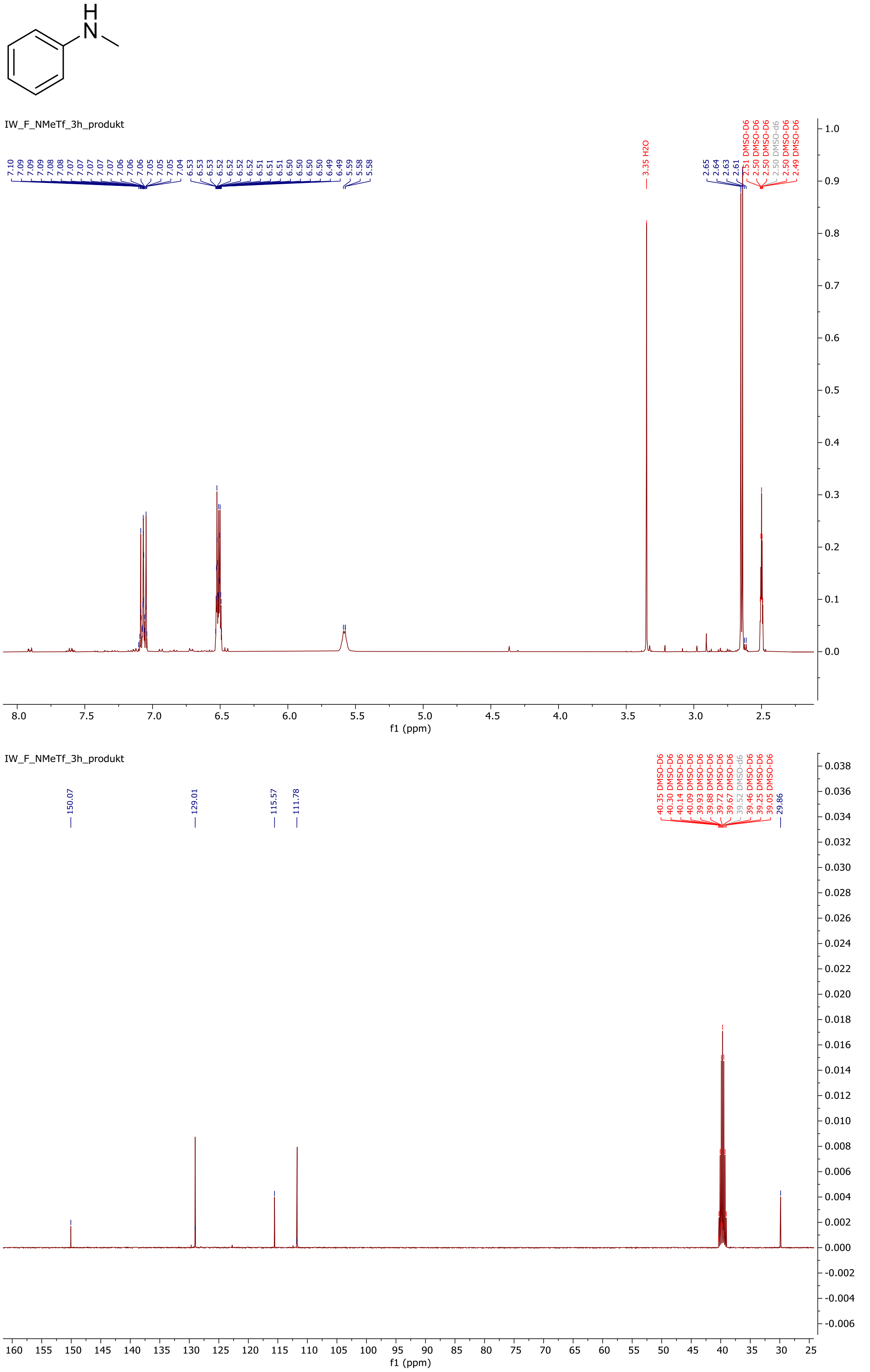

Figure S59: ^{1}H and ^{13}C NMR spectra of **8b**.

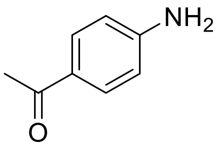

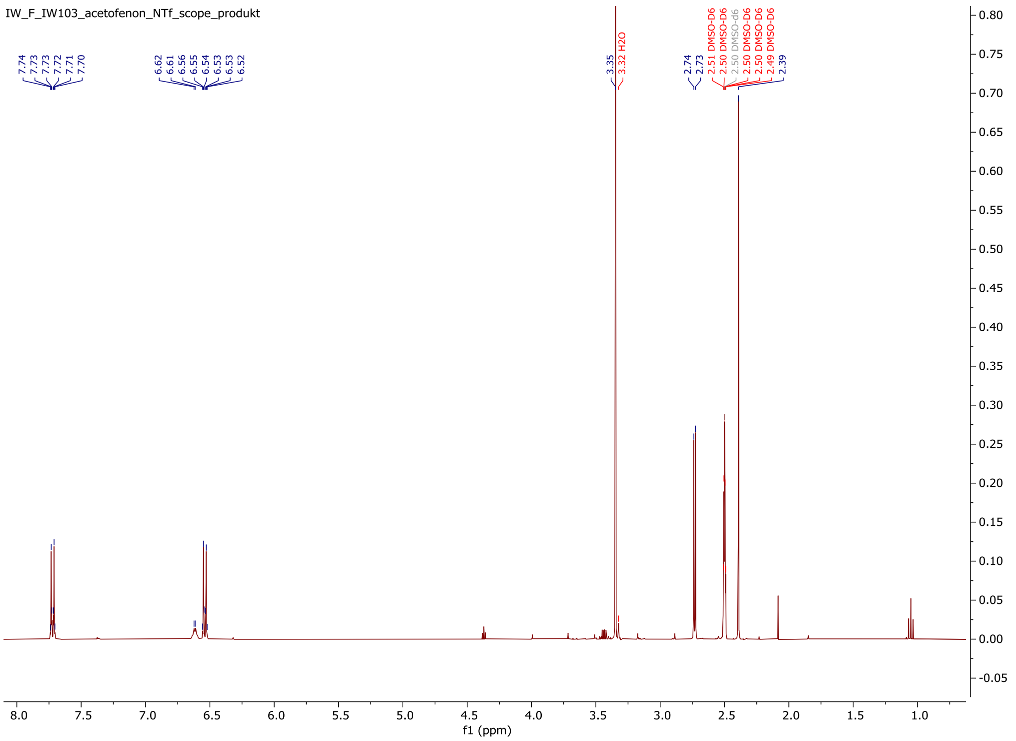

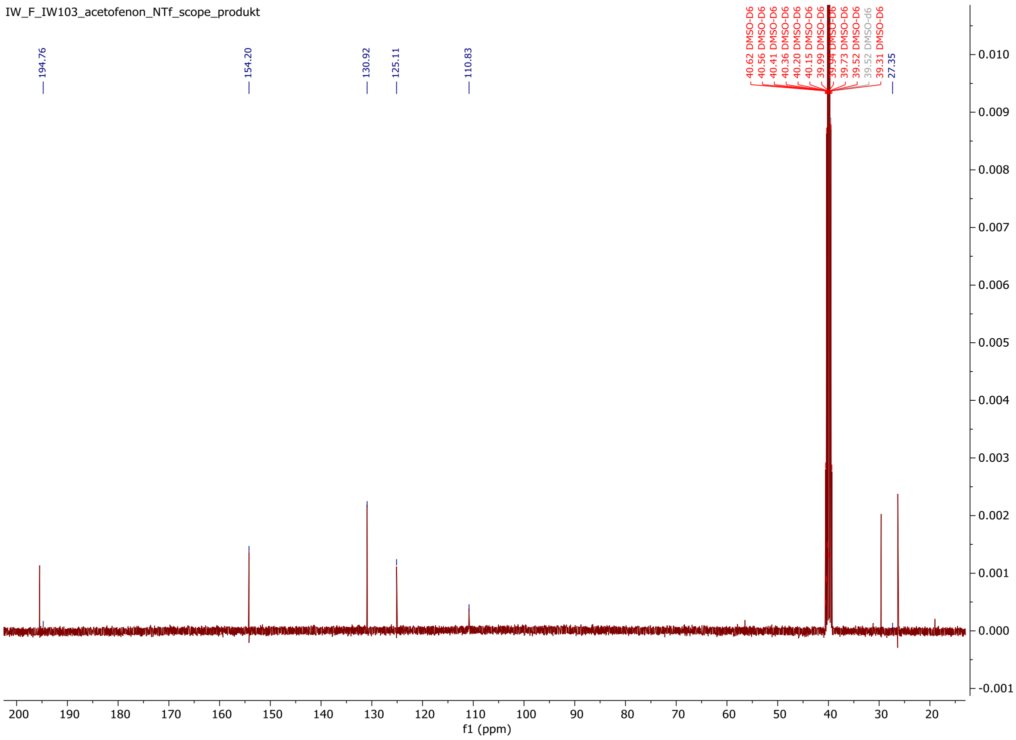

Figure S60: ^{1}H and ^{13}C NMR spectra of **8c**.

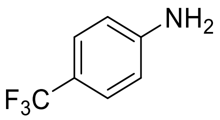
NH2
F3C

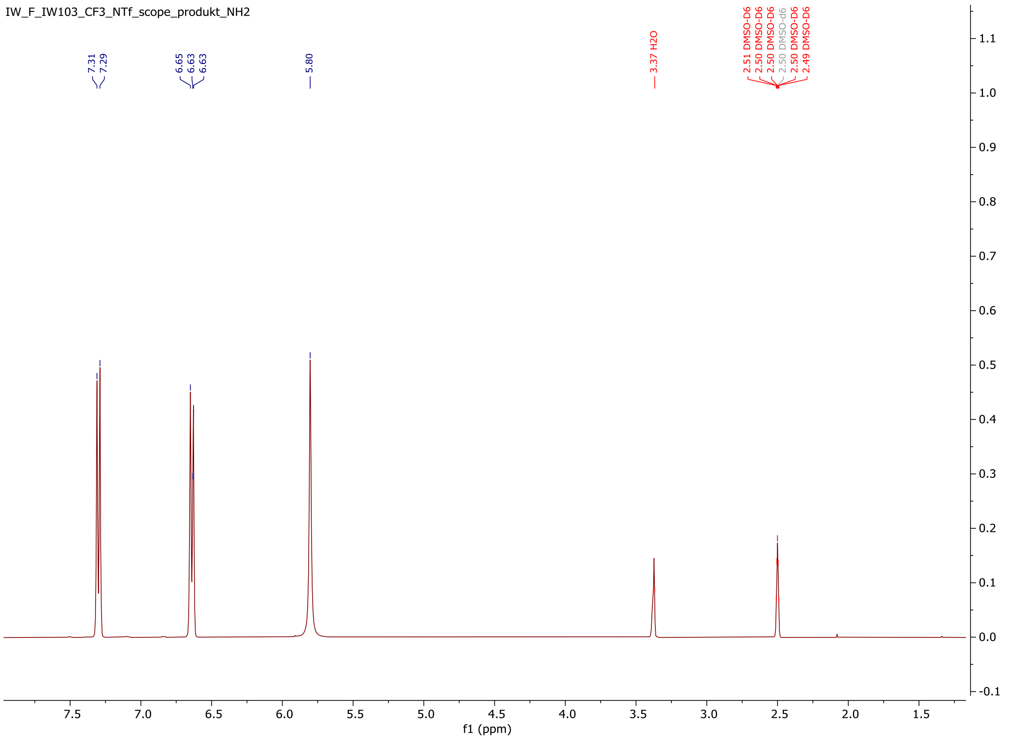
IW_F_IW103_CF3_NTf_scope_produkt_NH2
7.31
7.29
6.65
6.63
6.63
5.80
3.37 H2O
2.51 DMSO-D6
2.50 DMSO-D6
2.50 DMSO-D6
2.50 DMSO-d6
2.50 DMSO-D6
2.49 DMSO-D6
f1 (ppm)

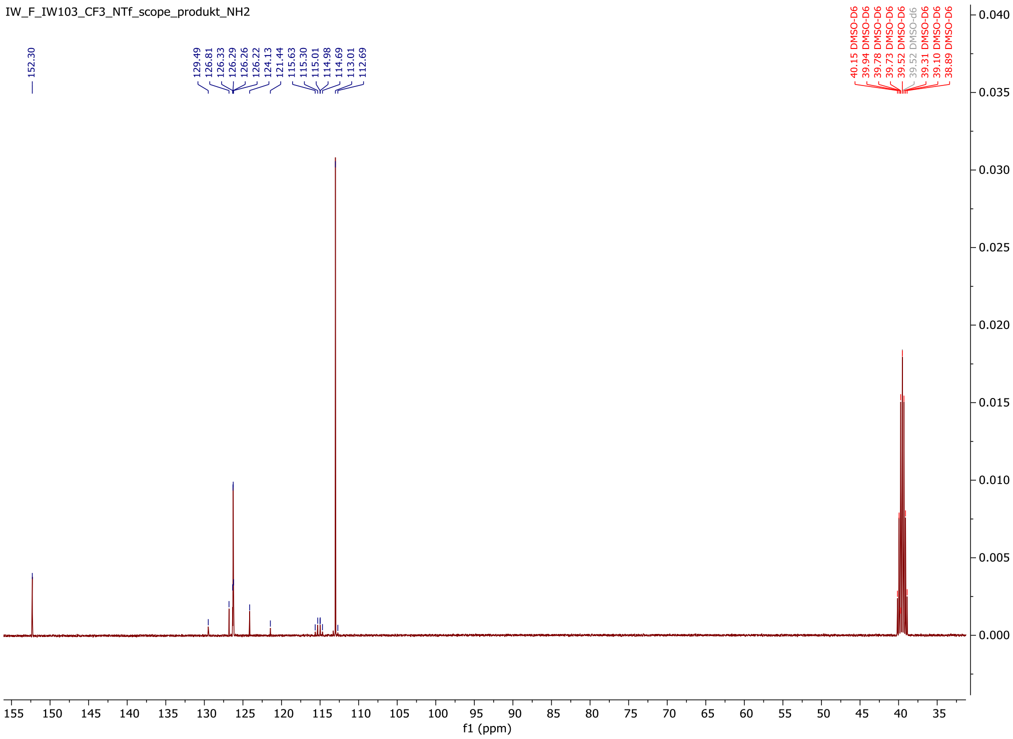
IW_F_IW103_CF3_NTf_scope_produkt_NH2
152.30
129.49
126.81
126.33
126.29
126.26
126.22
124.13
121.44
115.63
115.30
115.01
114.98
114.69
113.01
112.69
40.15 DMSO-D6
39.94 DMSO-D6
39.78 DMSO-D6
39.73 DMSO-D6
39.52 DMSO-D6
39.52 DMSO-d6
39.31 DMSO-D6
39.10 DMSO-D6
38.89 DMSO-D6
f1 (ppm)

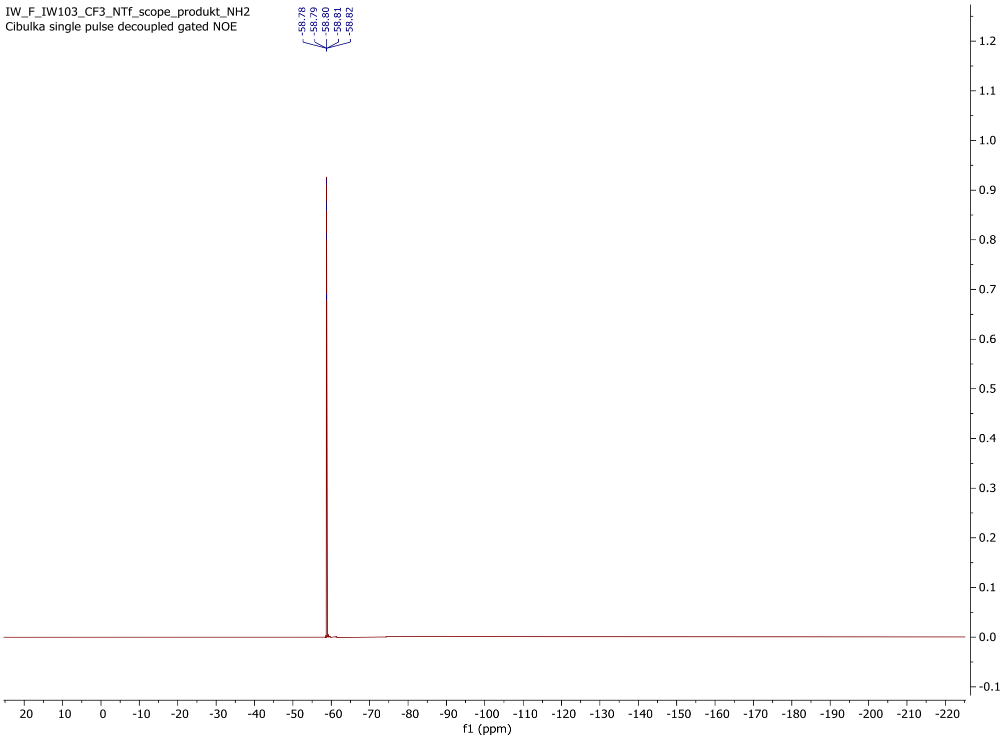

Figure S61: ^{1}H,^{13}C NMR and ^{19}F spectra of **8e**.

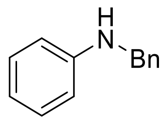

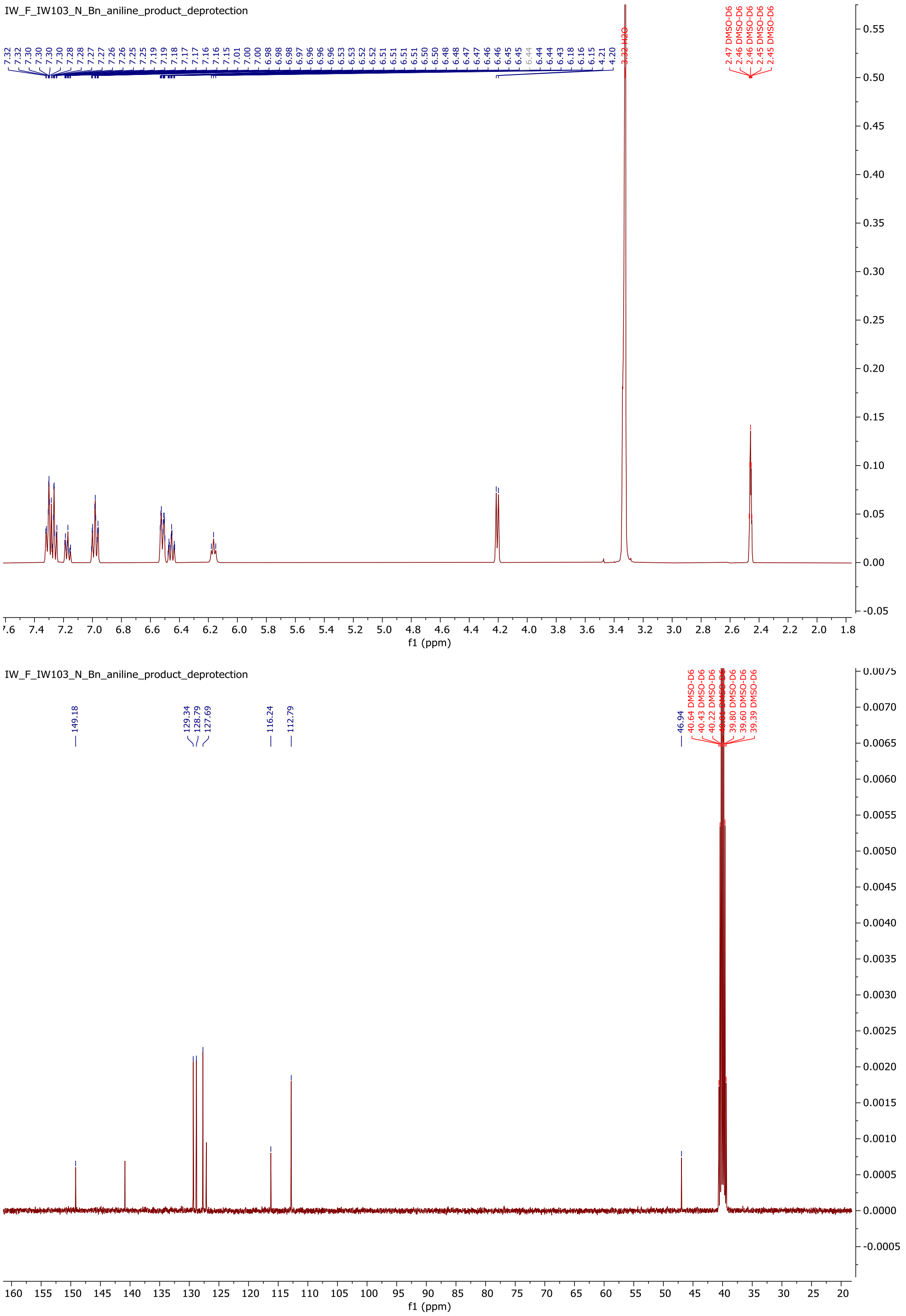

Figure S62: ^{1}H and ^{13}C NMR spectra of **8f**.

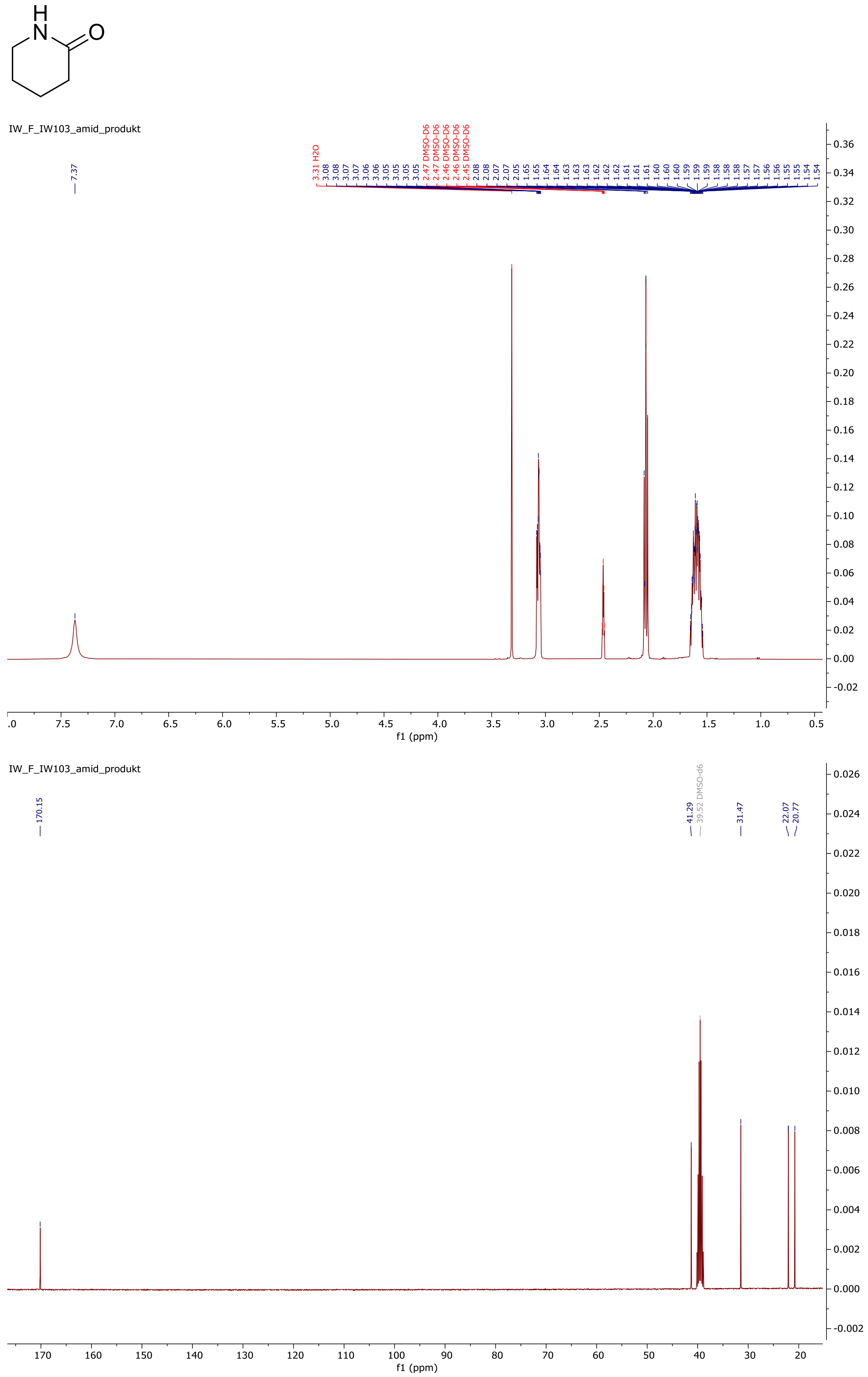

Figure S63: ^{1}H and ^{13}C NMR spectra of **11**.

S14. NMR SPECTRA OF ISOLATED PRODUCTS OF PHOTOREDUCTIVE COUPLING

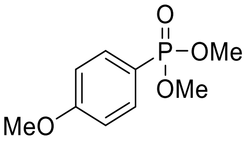

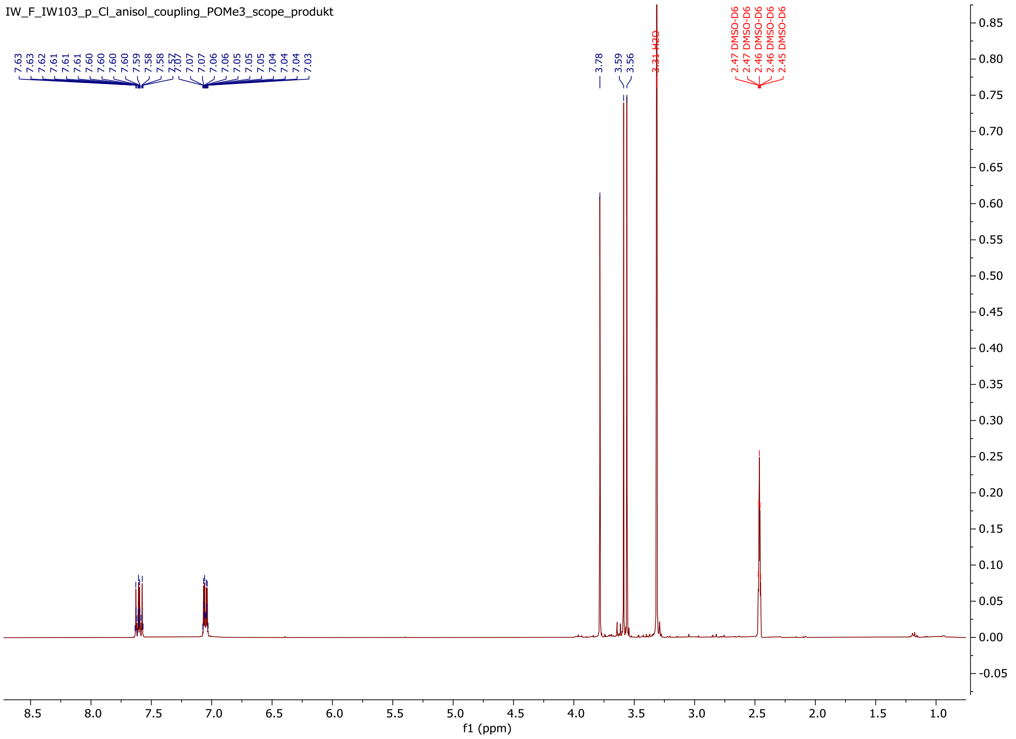

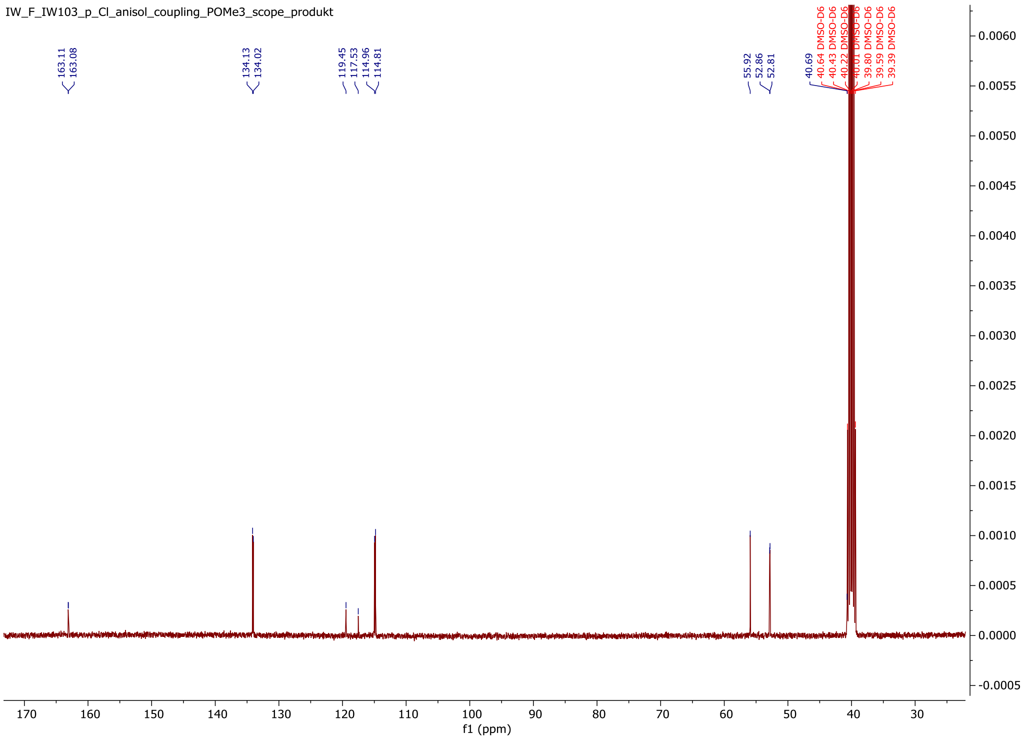

Figure S64: ^{1}H and ^{13}C NMR spectra of **10a**.

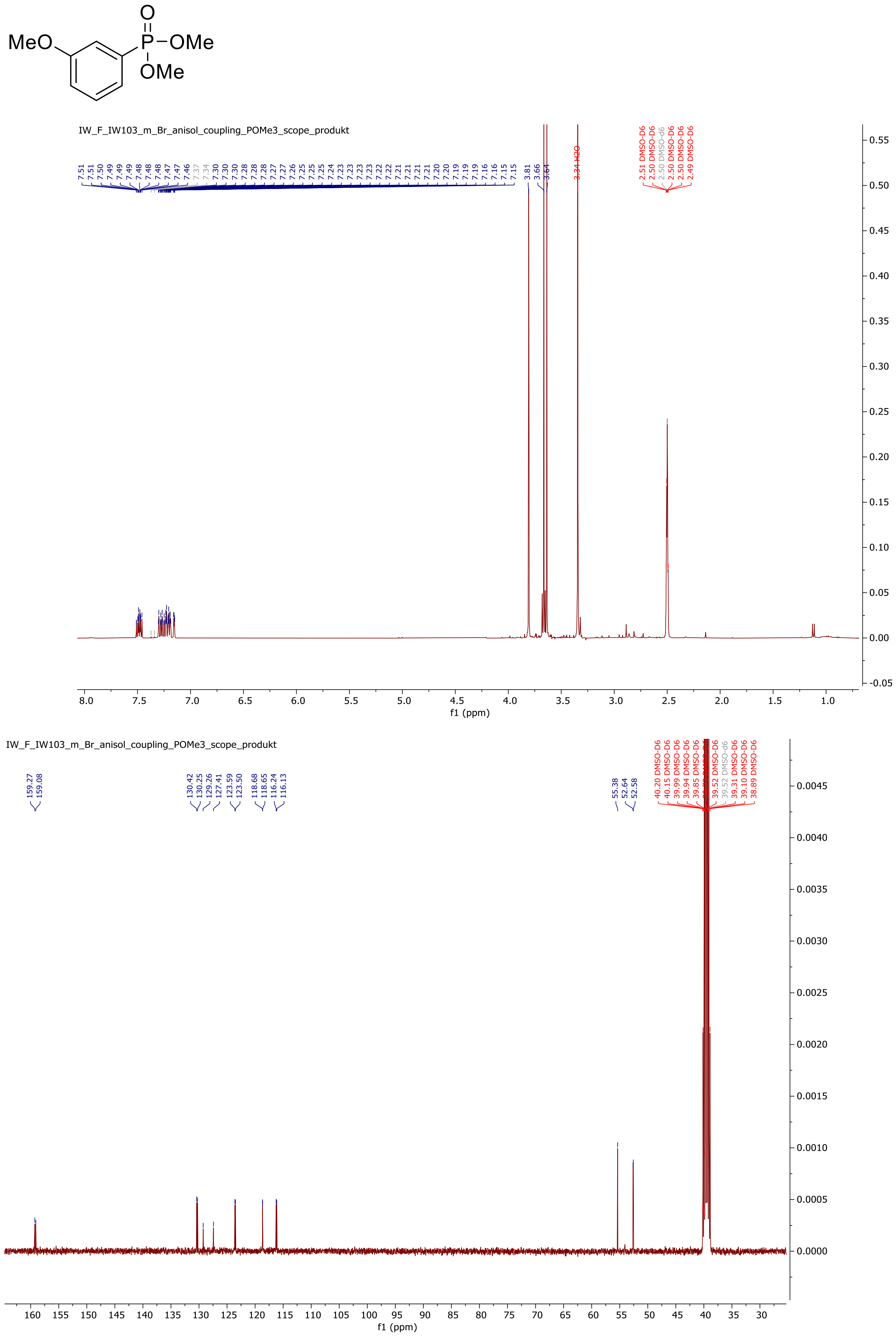

Figure S65: ^{1}H and ^{13}C NMR spectra of **10b**.

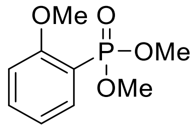

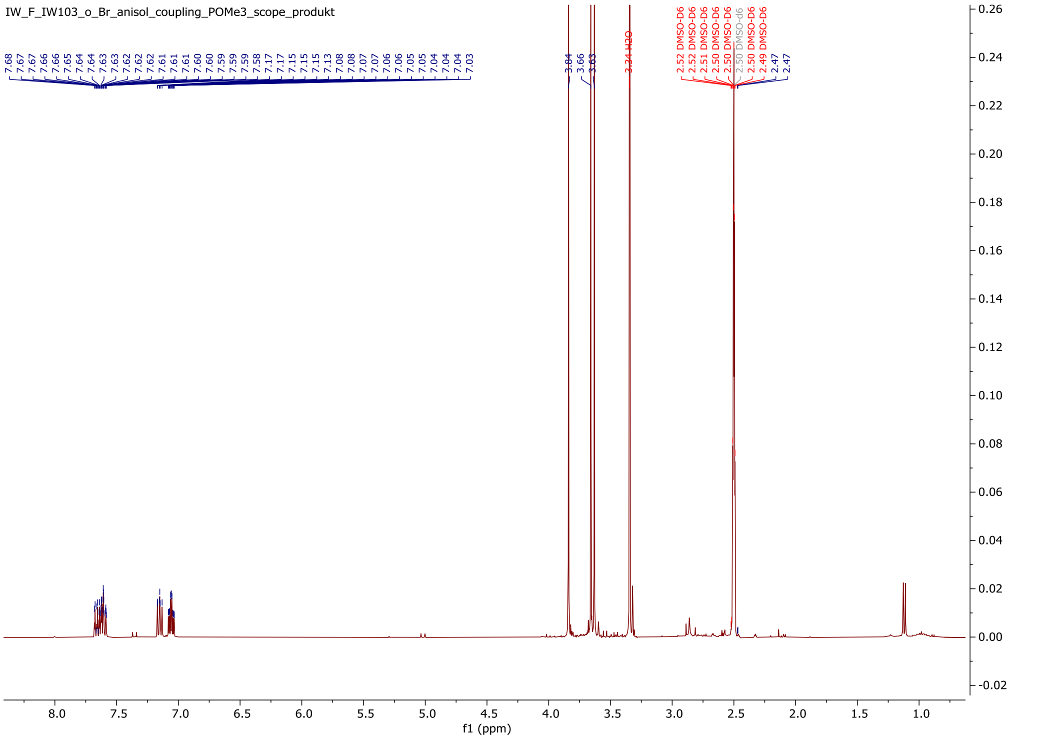

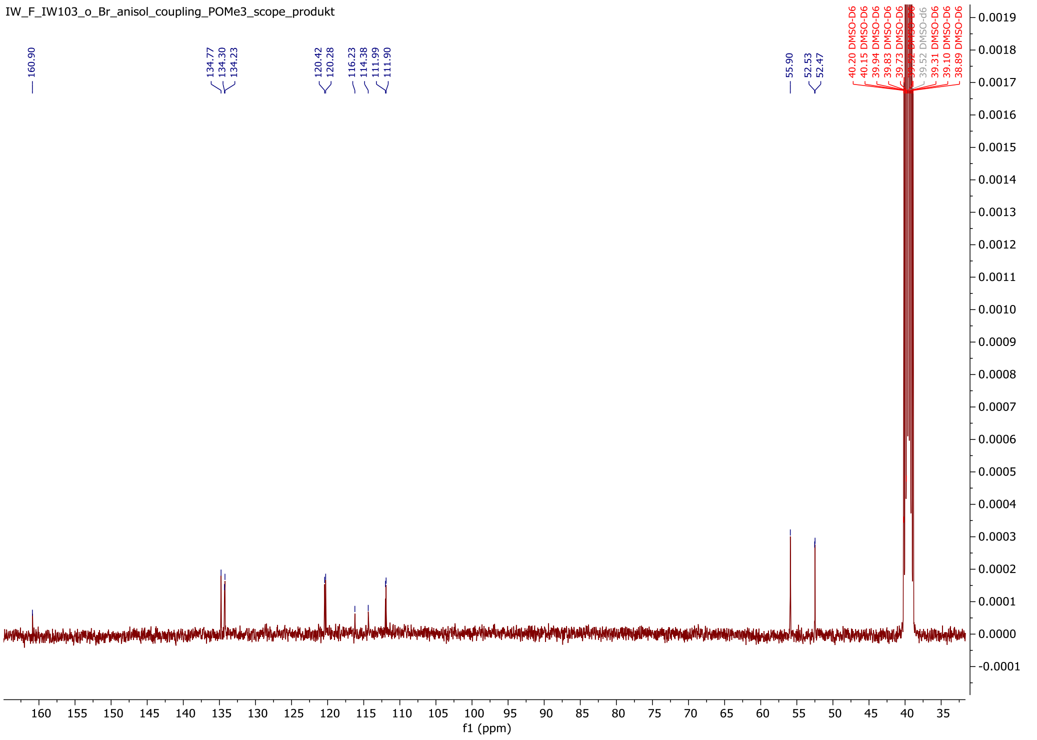

Figure S66: ^{1}H and ^{13}C NMR spectra of **10c**.

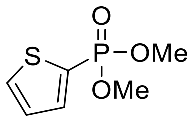

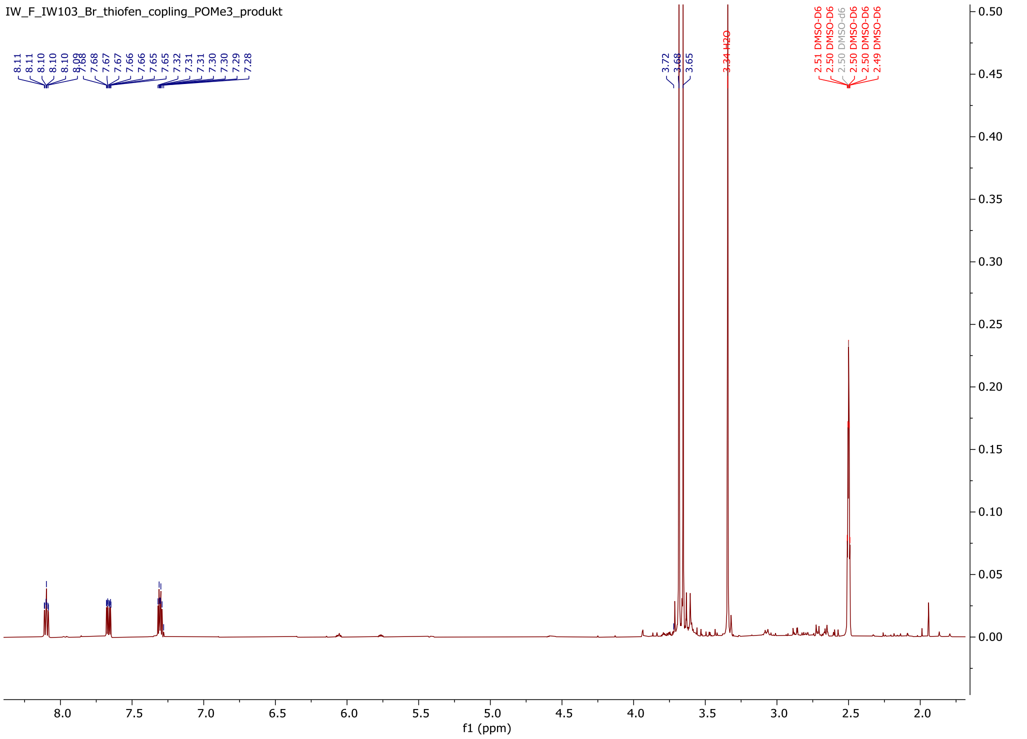

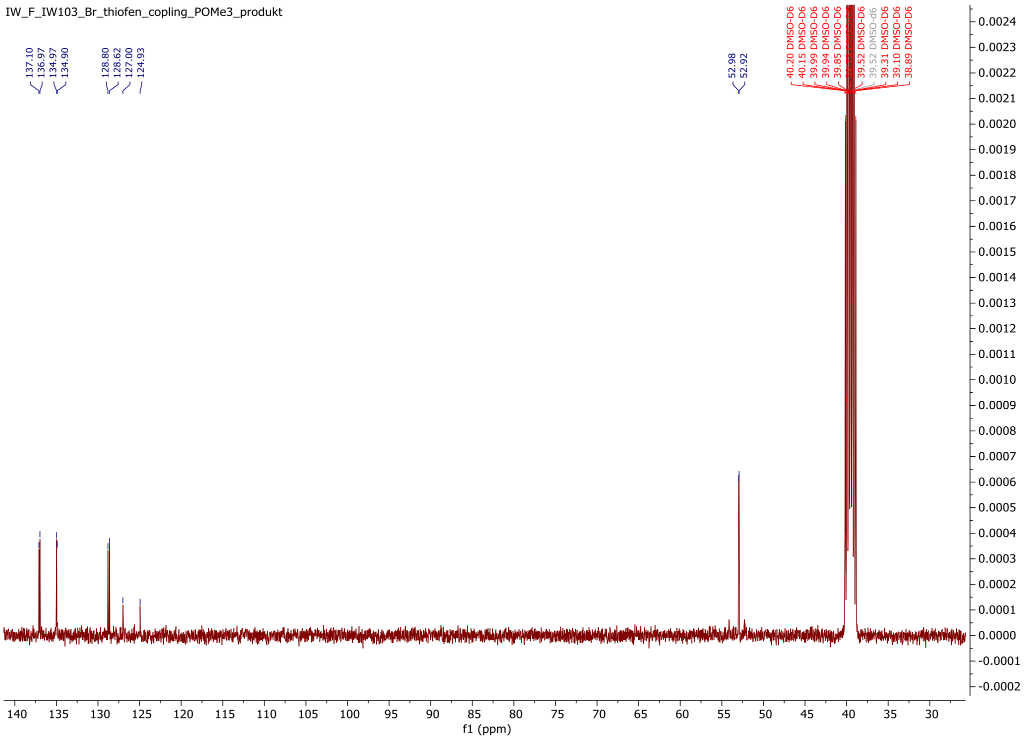

Figure S67: ^{1}H and ^{13}C NMR spectra of **10e**.

S15. CARTHESIAN COORDINATES

Caresian coordinates (in Å) of the ground state singlet equilibrium structure of **2e**.

	X	Y	Z
O	1.673502	-2.694926	0.000025
O	3.692188	1.387650	0.000034
N	2.690290	-0.648306	-0.000008
N	1.409476	1.358526	0.000039
N	-0.900388	1.318846	0.000007
C	-2.122400	-0.794955	-0.000008
C	-2.072018	0.632192	-0.000023
C	0.283171	-0.779544	0.000058
C	0.222812	0.641219	0.000046
C	-4.537845	-0.722928	-0.000070
C	-4.487754	0.688556	-0.000082
C	-3.376879	-1.450014	-0.000031
C	-3.288032	1.353410	-0.000059
C	1.574303	-1.481655	0.000124
C	2.657343	0.750873	0.000028
H	-3.239672	2.433092	-0.000067
C	4.026169	-1.253427	-0.000103
H	4.576983	-0.935663	0.881996
H	3.895748	-2.328672	-0.000593
H	4.577191	-0.934821	-0.881757
C	1.353041	2.822864	0.000043
H	0.820429	3.172364	-0.881575
H	0.820485	3.172366	0.881695
H	2.371035	3.191091	0.000013
C	-0.894722	-1.481066	0.000029
H	-3.401693	-2.532120	-0.000020
H	-5.411899	1.251004	-0.000112
H	-5.495493	-1.224658	-0.000088
H	-0.857420	-2.562394	0.000047

Caresian coordinates (in Å) of the ground state singlet equilibrium structure of **3a(*o*-BrPh)**.

	X	Y	Z
O	-3.092798	-0.940081	-0.729008
O	-4.302908	3.395402	-0.132656
N	-3.679235	1.219171	-0.424335
N	-2.075699	2.915893	-0.069923
N	0.176352	2.476247	0.008045
C	1.051820	0.222551	-0.274150
C	1.221089	1.619331	-0.053790
C	-1.326615	0.622268	-0.370414
C	-1.035628	1.997177	-0.142309
C	3.461082	-0.076848	-0.165885
C	3.624270	1.327375	0.070117
C	2.208879	-0.596473	-0.325154
C	2.521028	2.146138	0.115209
C	-2.729014	0.203379	-0.524591
C	-3.410949	2.571138	-0.202712
H	2.604496	3.206408	0.292393
C	-5.080433	0.810830	-0.568281
H	-5.233134	0.361685	-1.547366
H	-5.322871	0.067238	0.187539
H	-5.699460	1.690800	-0.452214
C	-1.752328	4.325752	0.165915
H	-1.222664	4.431884	1.109910
H	-1.109631	4.696916	-0.629138
H	-2.684345	4.875562	0.189276
C	-0.451719	-1.731937	-0.686162
C	-0.620740	-2.665445	0.334553
C	-0.391334	-2.207141	-1.997898
C	-0.745842	-4.021740	0.066358
C	-0.516083	-3.558654	-2.281399
H	-0.256047	-1.496754	-2.802250
C	-0.695312	-4.468452	-1.246882
H	-0.883434	-4.718463	0.879770
H	-0.474969	-3.898994	-3.306786
H	-0.795176	-5.524672	-1.455864
O	4.542289	-0.914149	-0.142767
C	5.426132	-0.842154	-1.265977
H	5.866723	0.149832	-1.363016
H	6.209621	-1.572181	-1.079845
H	4.895696	-1.104146	-2.185074
O	4.898549	1.740319	0.249349
C	5.134200	3.112851	0.543599
H	6.208554	3.208229	0.669891
H	4.802996	3.754728	-0.275373
H	4.629437	3.407787	1.465494
C	-0.265581	-0.273370	-0.430346
H	2.116794	-1.660816	-0.480142
Br	-0.706431	-2.100160	2.161947

Caresian coordinates (in Å) of the ground state singlet equilibrium structure of **3a(*o*-MePh).**

	X	Y	Z
O	3.128125	1.430421	-0.025129
O	4.572387	-2.868676	-0.209936
N	3.827411	-0.711789	-0.160613
N	2.321990	-2.527972	-0.087927
N	0.048039	-2.233558	-0.003431
C	-0.956874	-0.016845	-0.004703
C	-1.045698	-1.437532	0.025049
C	1.442241	-0.258748	-0.061001
C	1.230563	-1.667817	-0.048538
C	-3.382673	0.124477	0.070511
C	-3.464481	-1.305260	0.115168
C	-2.161649	0.732715	0.014967
C	-2.313966	-2.057273	0.085205
C	2.822263	0.253647	-0.079179
C	3.635511	-2.093965	-0.158203
H	-2.335835	-3.134856	0.116285
C	5.204525	-0.210082	-0.208884
H	5.305815	0.489314	-1.035072
H	5.439410	0.318324	0.713220
H	5.865633	-1.057171	-0.337376
C	2.078407	-3.973013	-0.072022
H	1.532646	-4.246240	0.828045
H	1.479138	-4.259073	-0.933593
H	3.041008	-4.467195	-0.098861
C	0.421201	2.068095	-0.122930
C	0.502214	2.855691	1.034863
C	0.352426	2.675653	-1.375136
C	0.518942	4.241845	0.889908
C	0.378175	4.058354	-1.499786
H	0.285084	2.057045	-2.260534
C	0.460623	4.845494	-0.359925
H	0.583747	4.858479	1.777487
H	0.331619	4.513424	-2.479808
H	0.478507	5.924049	-0.440470
O	-4.515295	0.885549	0.170815
C	-5.356242	0.924182	-0.986413
H	-5.725791	-0.068616	-1.242848
H	-6.192827	1.569079	-0.729999
H	-4.815727	1.349730	-1.835731
O	-4.714994	-1.811817	0.198207
C	-4.872246	-3.223016	0.293116
H	-5.941394	-3.397396	0.369710
H	-4.481794	-3.724445	-0.594923
H	-4.372950	-3.614069	1.181726
C	0.330178	0.577872	-0.050459
H	-2.129094	1.811653	-0.002421
C	0.607399	2.234323	2.401722
H	-0.170928	1.489214	2.573032
H	1.570340	1.733925	2.524502
H	0.524498	2.991460	3.179811

Cartesian coordinates (in Å) of the ground state singlet equilibrium structure of **3b(*o*-MePh).**

	X	Y	Z
O	-2.189945	2.053069	0.004983
O	-4.960941	-1.539264	-0.089418
N	-3.551475	0.255951	-0.089408
N	-2.720911	-1.953065	-0.026588
N	-0.472616	-2.413899	-0.005924
C	1.207138	-0.643477	-0.069157
C	0.822669	-2.015151	-0.019074
C	-1.144620	-0.093081	-0.057421
C	-1.405712	-1.498157	-0.028668
C	3.541620	-1.297649	-0.056475
C	3.159518	-2.657713	-0.006350
C	2.578494	-0.313578	-0.089309
C	1.831032	-3.000350	0.010697
C	-2.287499	0.841552	-0.044661
C	-3.820785	-1.115357	-0.071683
H	1.526230	-4.036602	0.046986
C	-4.691557	1.178418	-0.109369
H	-4.715924	1.757938	0.811603
H	-4.582770	1.869439	-0.941630
H	-5.595348	0.591941	-0.210508
C	-2.960360	-3.398630	0.004428
H	-2.511527	-3.868818	-0.867782
H	-2.506758	-3.829314	0.894041
H	-4.031599	-3.553183	0.009610
C	0.565715	1.778477	-0.185405
C	0.786319	2.556079	0.960917
C	0.784524	2.322930	-1.449350
C	1.214985	3.871594	0.793110
C	1.204820	3.638081	-1.596397
H	0.618260	1.710441	-2.325850
C	1.422054	4.416085	-0.468081
H	1.384875	4.481142	1.671666
H	1.363247	4.047079	-2.584941
H	1.752534	5.441358	-0.566402
O	4.838476	-0.879777	-0.076044
C	5.877202	-1.847074	-0.046528
H	5.835368	-2.507546	-0.916169
H	6.806279	-1.284557	-0.069920
H	5.842623	-2.445420	0.867296
H	3.906368	-3.436551	0.017796
C	0.171634	0.340174	-0.090795
H	2.890795	0.718638	-0.131438
C	0.533833	2.010274	2.340752
H	-0.534447	1.852556	2.503998
H	1.032432	1.052780	2.498961
H	0.887509	2.702868	3.102874

Caresian coordinates (in Å) of the ground state singlet equilibrium structure of **3b(thiophen-3-yl)**.

	X	Y	Z
O	-2.184169	2.091818	0.039626
O	-4.952891	-1.503162	-0.038939
N	-3.544208	0.292627	-0.006199
N	-2.712097	-1.916154	-0.023551
N	-0.464085	-2.376083	-0.029983
C	1.217933	-0.607119	0.011050
C	0.831232	-1.979146	-0.018729
C	-1.136625	-0.055556	0.019086
C	-1.397533	-1.460292	-0.010671
C	3.550863	-1.266218	0.024471
C	3.166780	-2.626136	-0.014938
C	2.589733	-0.279981	0.037313
C	1.837937	-2.966464	-0.034617
C	-2.280129	0.879741	0.019987
C	-3.813017	-1.078558	-0.023866
H	1.531073	-4.002365	-0.061398
C	-4.684289	1.215405	-0.009949
H	-4.656294	1.836300	0.882920
H	-4.623559	1.867906	-0.878135
H	-5.591700	0.626482	-0.036749
C	-2.950235	-3.362122	-0.047043
H	-2.501359	-3.798078	-0.936748
H	-2.496066	-3.826356	0.825338
H	-4.021359	-3.517539	-0.046795
O	4.848199	-0.850475	0.052939
C	5.885378	-1.819919	0.046565
H	5.860678	-2.428170	-0.861016
H	6.815169	-1.258809	0.074914
H	5.831795	-2.470693	0.922855
H	3.912350	-3.406475	-0.026822
H	2.903400	0.751852	0.069065
C	0.567120	1.810616	0.063679
C	0.995159	2.510756	-1.027149
C	0.602788	2.597267	1.254339
S	1.414470	4.133251	-0.624447
H	1.067506	2.168272	-2.045349
C	1.040623	3.866584	1.037879
H	0.301309	2.226736	2.222333
H	1.156959	4.661389	1.754547
C	0.181646	0.376031	0.026143

Cartesian coordinates (in Å) of the ground state singlet equilibrium structure of **3d(*o*-MePh)**.

	X	Y	Z
O	2.324991	1.921213	-0.003436
O	4.739304	-1.920814	-0.083210
N	3.509130	0.001541	-0.089207
N	2.469291	-2.116947	-0.027150
N	0.188245	-2.362669	-0.010670
C	-1.308605	-0.442828	-0.075136
C	-1.061970	-1.842210	-0.026018
C	1.081421	-0.115756	-0.059943
C	1.205356	-1.537407	-0.031453
C	-3.714007	-0.871999	-0.067017
C	-3.458748	-2.277967	-0.017543
C	-2.654393	0.000316	-0.096110
C	-2.161152	-2.727922	0.001067
C	2.305530	0.705299	-0.048476
C	3.645680	-1.388169	-0.069318
H	-1.944182	-3.787065	0.036736
C	-5.126616	-0.353208	-0.088721
H	-5.683828	-0.665391	0.797568
H	-5.140537	0.734387	-0.126672
H	-5.678820	-0.726117	-0.954442
C	-4.599976	-3.256639	0.012609
H	-5.241715	-3.093986	0.881443
H	-5.235820	-3.154593	-0.869681
H	-4.235992	-4.281451	0.049288
C	4.732042	0.810759	-0.109618
H	4.688238	1.510557	-0.940448
H	4.813610	1.383621	0.812298
H	5.575604	0.140872	-0.213277
C	2.569612	-3.579065	0.004545
H	2.074858	-3.964202	0.893010
H	2.080011	-4.004431	-0.868654
H	3.621278	-3.834815	0.012240
C	-0.448747	1.908086	-0.185714
C	-0.583166	2.702944	0.962190
C	-0.626091	2.471929	-1.447644
C	-0.887372	4.053199	0.797973
C	-0.920952	3.821229	-1.591413
H	-0.525687	1.846973	-2.325368
C	-1.053649	4.615730	-0.461429
H	-0.989923	4.675755	1.677893
H	-1.047565	4.244218	-2.578699
H	-1.285680	5.667941	-0.557026
C	-0.192044	0.438326	-0.094016
H	-2.846650	1.062859	-0.136505
C	-0.368816	2.135727	2.339844
H	-0.946411	1.224334	2.501356
H	0.683135	1.886099	2.494194
H	-0.654975	2.855524	3.105160

S16. REFERENCES

(1) Becke, A. D.; *J. Chem. Phys.,* **1993**, 98 (7), 5648-5652.

(2) Kendall, R. A.; Dunning Jr, T. H.; Harrison, R. J.; *J. Chem. Phys.,* **1992**, 96 (9), 6796-6806.

(3) Gaussian 16 Rev. C.01; Wallingford, CT, **2016**.

(4) Weisheitelová, I.; Cibulka, R.; Sikorski, M.; Pavlovska, T.; *Beilstein J. Org. Chem.*, **2024**, 20 (1), 1831-1838.

(5) Pavlovska, T.; Weisheitelová, I.; Pramthaisong, C.; Sikorski, M.; Jahn, U.; Cibulka, R.; *Adv. Synth. Catal.,* **2023**, 365 (24), 4662-4671.

(6) Cowden, W. B.; Halladay, P. K.; Cunningham, R. B.; Hunt, N. H.; Clark, I. A.; *J. Med. Chem.*, **1991**, 34 (6), 1818-1822.

(7) Dudkin, S.; Iaroshenko, V. O.; Sosnovskikh, V. Y.; Tolmachev, A. A.; Villinger, A.; Langer, P.; *Org. Biomol. Chem.,* **2013**, 11 (32), 5351-5361.

(8) Pfund, B. r.; Wenger, O. S.; *J. Am. Chem. Soc.*, **2025**, 5 (2), 426-447.

(9) vom Stein, T.; Meuresch, M.; Limper, D.; Schmitz, M.; Hölscher, M.; Coetzee, J.; Cole-Hamilton, D. J.; Klankermayer, J. r.; Leitner, W.; *J. Am. Chem. Soc.,* **2014**, 136 (38), 13217-13225.

(10) Sharma, U.; Kumar, P.; Kumar, N.; Kumar, V.; Singh, B.; *Adv. Synth. Catal.,* **2010**, 352 (11-12), 1834-1840.

(11) Goldschmid, S. L.; Soon Tay, N. E.; Joe, C. L.; Lainhart, B. C.; Sherwood, T. C.; Simmons, E. M.; Sezen-Edmonds, M.; Rovis, T; *J. Am. Chem. Soc.*, **2022**, 144 (49), 22409-22415.

(12) Wei, D.; Bruneau-Voisine, A.; Valyaev, D. A.; Lugan, N.; Sortais, J.-B.; *Chem. Comm.*, **2018**, 54 (34), 4302-4305.

(13) Kiely-Collins, H. J.; Sechi, I.; Brennan, P. E.; McLaughlin, M. G; *Chem. Comm.,* **2018**, 54 (6), 654-657.

(14) Xu, J.; Cao, J.; Wu, X.; Wang, H.; Yang, X.; Tang, X.; Toh, R. W.; Zhou, R.; Yeow, E. K.; Wu, J.; *J. Am. Chem. Soc.,* **2021**, 143 (33), 13266-13273.

(15) Wu, S.; Schiel, F.; Melchiorre, P.; *Angew. Chem*., **2023**, 135 (32).

(16) Rasheed, S.; Rao, D. S.; Subramanyam, C.; Basha, S. T.; Raju, C. N.; *Synth. Commun.,* **2014**, 44 (20), 2988-2998.
